# Multiomics analysis provides insights into musk secretion in muskrat and musk deer

**DOI:** 10.1093/gigascience/giaf006

**Published:** 2025-02-26

**Authors:** Tao Wang, Maosen Yang, Xin Shi, Shilin Tian, Yan Li, Wenqian Xie, Zhengting Zou, Dong Leng, Ming Zhang, Chengli Zheng, Chungang Feng, Bo Zeng, Xiaolan Fan, Huimin Qiu, Jing Li, Guijun Zhao, Zhengrong Yuan, Diyan Li, Hang Jie

**Affiliations:** School of Basic Medical Sciences, Chengdu University, Chengdu, 610106, China; Jinfo Mountain Forestry Ecosystem of Chongqing Observation and Research Station, Chongqing Institute of Medicinal plant cultivation, Chongqing University of Chinese Medicine, Chongqing 402760, China; School of Pharmacy, Chengdu University, Chengdu 610106, China; Sichuan Institute of Musk Deer Breeding, Sichuan Institute for Drug Control, Chengdu 611845, China; College of Life Sciences, Wuhan University, Wuhan 430072, China; Chengdu Research Base of Giant Panda Breeding, Chengdu 611081, China; College of Biological Sciences and Technology, Beijing Forestry University, Beijing 100083, China; Key Laboratory of Zoological Systematics and Evolution, Institute of Zoology, Chinese Academy of Sciences, Beijing 100101, China; College of Animal Science and Technology, Sichuan Agricultural University, Chengdu 611130, China; College of Animal Science and Technology, Sichuan Agricultural University, Chengdu 611130, China; Sichuan Institute of Musk Deer Breeding, Sichuan Institute for Drug Control, Chengdu 611845, China; College of Animal Science and Technology, Nanjing Agricultural University, Nanjing 210095, China; College of Animal Science and Technology, Sichuan Agricultural University, Chengdu 611130, China; College of Animal Science and Technology, Sichuan Agricultural University, Chengdu 611130, China; College of Agriculture, Kunming University, Kunming 650214, China; College of Agriculture, Kunming University, Kunming 650214, China; School of Pharmacy, Chengdu University, Chengdu 610106, China; College of Biological Sciences and Technology, Beijing Forestry University, Beijing 100083, China; School of Pharmacy, Chengdu University, Chengdu 610106, China; Jinfo Mountain Forestry Ecosystem of Chongqing Observation and Research Station, Chongqing Institute of Medicinal plant cultivation, Chongqing University of Chinese Medicine, Chongqing 402760, China

**Keywords:** musk deer, muskrat, musk secretion, multiomics

## Abstract

**Background:**

Musk, secreted by the musk gland of adult male musk-secreting mammals, holds significant pharmaceutical and cosmetic potential. However, understanding the molecular mechanisms of musk secretion remains limited, largely due to the lack of comprehensive multiomics analyses and available platforms for relevant species, such as muskrat (*Ondatra zibethicus* Linnaeus) and Chinese forest musk deer (*Moschus berezovskii* Flerov).

**Results:**

We generated chromosome-level genome assemblies for the 2 species of muskrat (*Ondatra zibethicus* Linnaeus) and musk deer (*Moschus berezovskii* Flerov), along with 168 transcriptomes from various muskrat tissues. Comparative analysis with 11 other vertebrate genomes revealed genes and amino acid sites with signs of adaptive convergent evolution, primarily linked to lipid metabolism, cell cycle regulation, protein binding, and immunity. Single-cell RNA sequencing in muskrat musk glands identified increased acinar/glandular epithelial cells during secretion, highlighting the role of lipometabolism in gland development and evolution. Additionally, we developed MuskDB (http://muskdb.cn/home/), a freely accessible multiomics database platform for musk-secreting mammals.

**Conclusions:**

The study concludes that the evolution of musk secretion in muskrats and musk deer is likely driven by lipid metabolism and cell specialization. This underscores the complexity of the musk gland and calls for further investigation into musk secretion–specific genetic variants.

## Introduction

Natural musk (Moschus) is mainly secreted by the musk gland located between the navel and genitals of mature male forest musk deer (*Moschus berezovskii* Flerov), an endangered artiodactyl species native to southern and central China and northernmost Vietnam [1]. Natural musk has long been an important component of traditional Chinese medicine and was used for resuscitation, blood circulation, collateral drainage, detumescence, and pain relief [2]. In addition, the muskrat (*Ondatra zibethicus* Linnaeus), a semiaquatic rodent native to North America [3] and Canada but has been introduced to Europe, Asia, South America, and Australia, has a similar musk gland and secrets musk likewise. The musk secreted by forest musk deer and muskrat produces a specific fragrance, whose chemical composition may be involved in chemical communication, potentially encoding information about sexual maturity and attraction [4]. The chemical composition analysis of musk showed that it contained active macrocyclic ketone components such as muscone and normuscone [5]. Muskrat musk also contains macrocyclic ketone compounds such as muscone and normuscone [6]. This class of substances is thought to be necessary for exerting drug effects. Our previous research indicated that musk of muskrat and musk deer has up to 272 identical metabolites, including organic compounds such as amino acids, fatty acids, ketones, aldehydes, and steroids [7].

Compared with other musk-secreting mammals (i.e., other musk deer species), there has been more captive breeding practice for the forest musk deer and the muskrat. The high-quality genome sequences of these 2 species and comparative analyses with the other mammalian genomes can potentially shed light on their genome diversity and the genetic components underlying musk secretion, which may have experienced convergent adaptation during the long process of evolution. However, there are no convergent evolutionary studies on musk-producing animals to elucidate the related mechanism of musk secretion. In the process of evolution, it is crucial to identify which genes are subject to positive and convergent evolution. Additionally, identifying the genes that are differentially expressed in different cells of the musk gland during musk secretion is critical. The regulation of the molecular process of musk secretion by these genes from chromatin conformation remains unknown.

Here, we sequenced the genomes of a male muskrat and a male musk deer. In addition, to characterize the transcriptomic variability with respect to known tissue-specific physiological activities and identify key genes underlying the musk-secreting phenotype, we sequenced 84 RNA sequencing (RNA-seq) libraries and 84 small RNA-seq libraries of 13 various muskrat organs. To accurately depict cell composition and transcriptomic changes in the musk gland of muskrat between musk secretion and nonsecretion stages, we further used a single-cell RNA (scRNA) approach to dissect the transcriptional differences. We also examined the potential chromatin architecture dynamics underlying the phenotype by sequencing 3 and 4 Hi-C libraries for the musk gland, respectively, in the secretion and nonsecretion stages (Supplementary Table S1). Integrated with these multiomics data, the study identified particular genes, such as *SMPDL3A* and *NRCAM*, that exhibit unique patterns of genetic interactions and changes in their genomic neighborhoods, suggesting they may play key roles in controlling the musk secretion process. The research concludes that the evolutionary adaptation for musk production in both the muskrat and the musk deer is likely driven by robust lipid metabolism and specialized cell functions, underscoring the musk gland’s intricate nature. This finding emphasizes the necessity for continued investigation to uncover the full functional impact of genetic variations specific to the musk secretion process.

## Results and Discussion

### Genome assembly of 2 musk-secreting mammals

**Figure 1:**
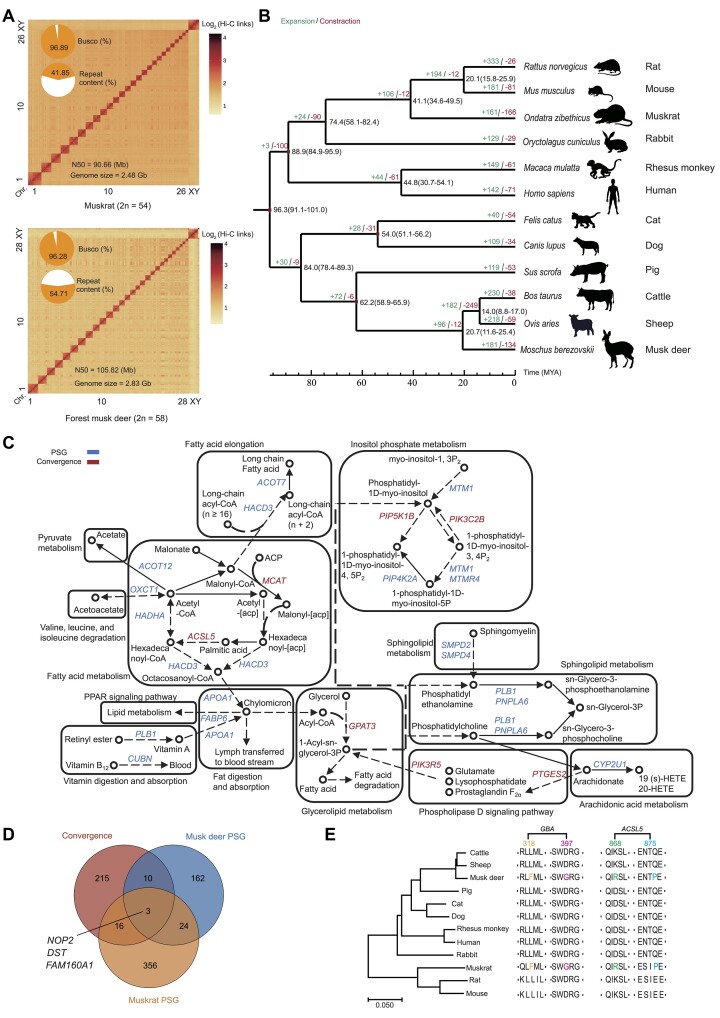
Genome assembly and gene family evolution in muskrat and musk deer. (A) Hi-C heatmaps for muskrat and forest musk deer. Pie charts represent the proportion of conserved BUSCO gene sets and repeat content. Contig N50 and assembled genome size are shown. (B) Divergence times and expansion and contraction of gene families in muskrat and musk deer genomes. Numbers on the nodes represent divergence times, with the error range shown in parentheses. The numbers of gene families that expanded (green) or contracted (red) in each lineage after speciation are shown on the corresponding branch. (C) Enrichment for PSGs and convergence genes that functions in the metabolism of lipids (only partial pathways are shown). The PSGs are shown in blue, and the convergence genes are shown in red. (D) The Venn diagram shows the number of PSGs and REGs in muskrat and musk deer. (E) The sequence alignment shows sites of the convergence gene in *GBA* and *ACSL5*.

We sequenced the genomes of 2 male musk-secreting mammals (*O. zibethicus* [muskrat] 2 years of age and *M. berezovskii* Flerov [forest musk deer] 2.5 years of age) via integration of Oxford Nanopore Technologies (ONT) long reads, high-throughput chromosome conformation capture (Hi-C) data, and BGI T7 paired-end sequences (Supplementary Table S1) to over 235.25-fold (∼583.41 Gb) and 246.02-fold (∼696.23 Gb) coverage, respectively. We assembled the 2 chromosome-level genomes by applying an improved assembly method that utilizes Hi-C interaction pairs to cluster ONT long sequences with potential linkages and avoid any erroneous overlap caused by long-distance repetitive sequences during string graph assembly [8] (see Materials and Methods). We successfully generated 2.48 Gb and 2.83 Gb for muskrat and musk deer genomes with contig N50 values of 60.53 and 69.45 Mb, which anchored onto 28 and 30 chromosomes, respectively (Supplementary Tables S1, S2; Fig. 1A; Table 1). The 28 anchored chromosomes in muskrat were confirmed by karyotype analysis (Supplementary Fig. S1a). In particular, 5 chromosome sequences have reached the gap-free level in the muskrat genome (Supplementary Table S3), and our 2 assemblies have improved the N50 length of the contig by 1,048- and 3.56-fold for the muskrat and musk deer compared to the published sequences, respectively (Supplementary Fig. S1b, c). Our assembled genomes exhibit excellent completeness, as evidenced by the coverage of >99% paired-end reads across >99% of the genome and recovery of averaged 96.95% of BUSCOs [9] in 9,226 conserved mammalian genes from the mammalia_odb10 database (Supplementary Table S4). Furthermore, we used a reference-free and *k*-mer–based approach and estimated a high assembly quality value (QV) of more than 44, exceeding the Vertebrate Genome Project (VGP) standard of QV40 [10, 11]. Subsequently, we predicted 1,013.31 Mb (40.85%) and 1,539.41 Mb (54.33%) transposable elements (TEs) for muskrat and musk deer, respectively (Supplementary Table S5). By combining homology- and *ab initio–*based methods, aided by evidence of transcription, we identified 23,260 and 24,375 protein-coding genes in the muskrat and musk deer genomes, respectively (Supplementary Table S5, Supplementary Fig. S1d, e).

**Table 1:** Global summary of 2 assemblies for muskrat and musk deer.

Genomic features	Muskrat	Musk deer
Assembled genome size (Gb)	2.48	2.83
Percentage of anchoring (%)	97.19	98.79
Contig number	561	1,173
Contig N50 (Mb)	60.53	69.45
GC content (%)	41.69	42.14
Repeat ratio (%)	40.85	54.33
Predicted number of protein-coding genes	23,260	24,375
QV	44.48	44.09
BUSCOs (%)	96.89	96.28

Next, we explored gene family expansion and contraction in 2 musk secretion species. As a result, we determined that 181 gene families underwent an expansion, and 134 underwent a contraction for the musk deer. Muskrat shows comparable numbers of gene family contraction (166) and expansion (161) events (Fig. 1B). Gene Ontology (GO) enrichment analyses of contracted genes indicated that these 2 species were both involved in pathways like the “olfactory signaling pathway,” “olfactory transduction,” “signaling by GPCR,” “B-cell receptor signaling pathway,” “tight junction immunoregulatory interactions between a lymphoid and a non-lymphoid cell,” “natural killer cell-mediated cytotoxicity,” and “autoimmune thyroid disease” (Supplementary Fig. S2a). Gene families underwent an expansion in these 2 species and were involved in biological processes like “cell cycle,” “meiotic synapsis,” “mitotic anaphase,” and “estrogen-dependent gene expression” (*P* < 0.05) (all Fisher exact test) (Supplementary Tables S7–S8).

### Positive selection and rapid evolution genes in muskrat and musk deer were mainly involved in the metabolism of lipids and epithelial regulation

To explore the function of positively selected genes (PSGs) and rapidly evolving genes (REGs) in muskrat and musk deer during evolution, we next analyzed 7,409 gene trees based on one-to-one orthologs, each constrained to the reconstructed species phylogeny. By applying branch tests and branch-site tests in PAML [12] to the corresponding branches, we identified 443 REGs and 399 PSGs for muskrats, as well as 523 REGs and 199 PSGs for musk deer, respectively (Supplementary Table S6). Interestingly, the gene *Hdac1* was in GO term “epidermal cell differentiation,” which is enriched by gene family expansion in the forest musk deer and muskrat, coinciding with the musk gland–specific gene. These respective sets of genes were mainly involved in the “metabolism of proteins” and “metabolism of lipids” (Supplementary Tables S9–S10). We also identified that PSGs like *ANAPC4, CDC16*, and *RBL2* were involved in the cell cycle pathway (Supplementary Fig. S2b). Muskrat PSGs (*HBEGF, PIGR, PLCE1, NCK2*) were enriched in the “epidermal growth factor receptor signaling pathway,” and REGs (*TGFBR3, BMP4, STRAP, ATF2, CDC73*) were enriched in “negative regulation of epithelial cell proliferation.” In addition, we deduced the convergent sites in musk secretion species (muskrat and musk deer) at each node for all the 7,409 single-copy orthologs based on the phylogenetic tree (Fig. 1B) using 2 methods (JTT-Fgene [13, 14] and CCS [15]). As a result, a total of 244 genes were identified under the JTT-Fgene model (false discovery rate [FDR] < 0.05, Poisson test), whereas 457 genes were identified by the CCS method [15]. In total, 209 genes were detected as convergent evolution for muskrat and musk deer by both methods (JTT-Fgene and CCS). We performed functional gene enrichment analyses for convergent evolution genes and found that the gene sets were also significantly enriched for “metabolism of proteins” (*P* = 0.045, Fisher exact test) and “metabolism of lipids and lipoproteins” (*P* = 0.035, Fisher exact test).

*RDH8* is a convergent evolution gene involved in “the canonical retinoid cycle in rods (twilight vision)” in muskrat and musk deer. Although olfactory receptor genes are contracted in these 2 species, several vision-related genes were PSGs or convergent evolution genes, indicating they have a sensory trade-off otherwise observed in arboreal species [16] and giraffes [17], which is consistent with the fact that both species are timid and sensitive. *TEX15*, a testis-specific protein, is required for TE silencing. A previous study indicated that *TEX15*, a new essential epigenetic regulator, may function as a nuclear effector of MILI to silence TEs by DNA methylation [18]. Our study suggested that the *TEX15* gene is an outlier with 7 unique amino acid convergent substitutions for muskrat and musk deer (Supplementary Fig. S2c). This indicated that the *TEX15* gene not only plays a role in male germ cells but might also have an essential role in forming the male characteristic organ of a musk-secreting species.

For the “metabolism of lipids and lipoproteins” pathway, we identified 10 convergent evolution genes (*SLC44A2, GBA, PTGES2, GPAT3, PIK3R5, ACSL5, MED12, ACBD6, PIP5K1B, BDH2*) in this pathway (*P* = 0.035, Fisher exact test) in muskrat and musk deer (Fig. 1C). We also checked the overlapped genes between PSGs, REGs, and convergent evolution genes and found *NOP2, DST, FAM160A1*, and *CKAP5* were shared between them (Fig. 1D, Supplementary Fig. S3). These genes have been less studied, such as FAM160A1, a member of the UPF0518 family of proteins, each containing a conserved retinoic acid–induced 16 (RAI16)–like domain with unknown biological function [19]. *CKAP5* enables the formation of persistent actin bundles on dynamic microtubules [20]. Among these convergent evolution genes, *ACSL5* and *GBA* have 2 amino acid substitutions (Fig. 1E). The protein encoded by the *ACSL5* gene is an isozyme of the long-chain fatty acid coenzyme A ligase family, which catalyzes the formation of fatty acyl-CoAs from long-chain fatty acids (C16–C20). Fatty acyl-CoAs are then used in lipid synthesis or β-oxidation–mediated pathways [21]. The *GBA1* gene encodes the lysosomal enzyme β-glucocerebrosidase (GCase) that degrades glucosylceramide and is pivotal in glycosphingolipid substrate metabolism [22]. In addition, choline is essential for synthesizing phospholipids [23], and the gene *SLC44A2* participates in the process. These results indicate a rapid evolution of lipid metabolism in these 2 species. In addition, some convergent evolution genes that also have roles in “metabolism of lipids” (*P* = 0.0002) and “cell cycle, mitotic” (*P* = 0.006) were also detected (Supplementary Fig. S2a). This might be because the male musk gland has a cyclic change; in the musk secretion stage, the musk gland is atrophic, and in the stage of musk nonsecretion, the glands become larger, accompanied by cell proliferation and differentiation.

### Tissue-specific expression of genes in the musk gland

**Figure 2:**
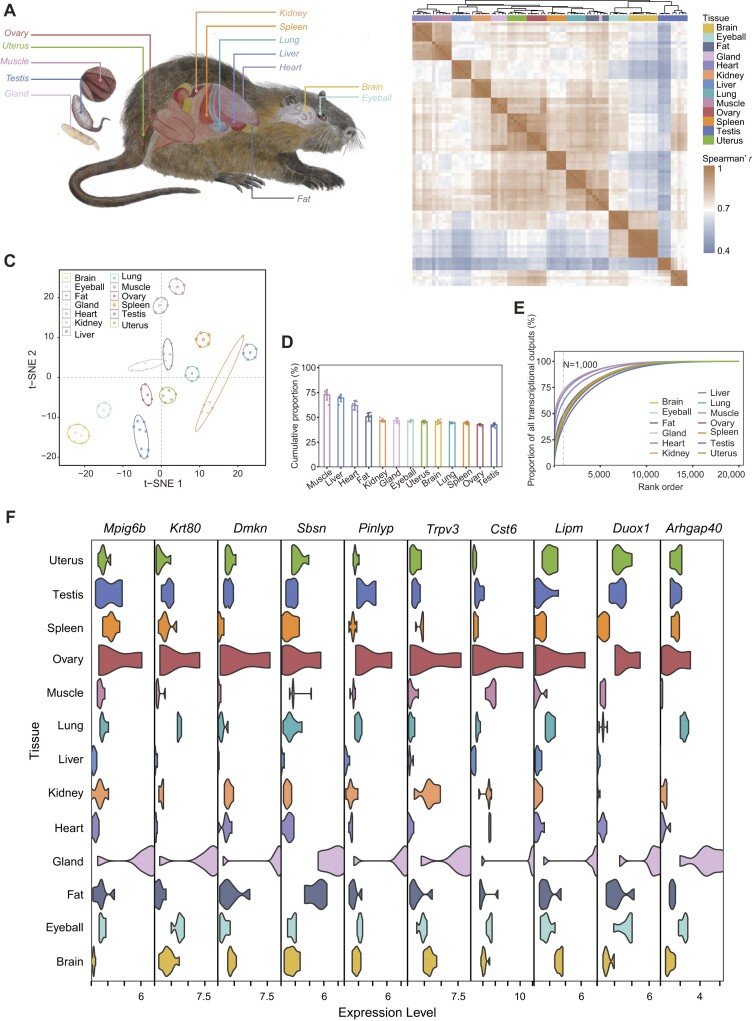
Characteristics of the muskrat BodyMap transcriptome. (A) Samples derived from 13 tissues were used for muskrat transcriptome reconstruction. (B) Hierarchical clustering and Spearman *r* heatmap of samples using expression values (TPM). (C) The t–distributed stochastic neighbor embedding (t-SNE) clustering of samples using expression values (TPM). The ellipses indicate the samples of the same tissue with similar transcriptional profiles, constructed at a probability of 0.95 (*n* = 19,800). (D) The cumulative expression proportion of the top 1,000 highly expressed genes in all samples. (E) Abundance distribution of transcripts across 13 tissues. The x-axis indicates the proportion of transcripts sorted from highest to lowest expression, with the vertical dashed line indicating the top 1,000 of the highest abundance transcripts. The y-axis indicates the accumulated fraction of transcripts relative to the total transcripts. Colored lines represent mean values across different tissues. (F) The genes specifically expressed in musk gland.

To explore the genes specifically expressed in the musk gland and their functions, we used a muskrat as a model animal to conduct further analyses, as muskrat tissue samples are accessible in contrast to the endangered musk deer. We first constructed 84 RNA-seq libraries and 84 small RNA-seq libraries to explore tissue-specific expression patterns from 13 tissues (2 from entoderm [liver and lung], 8 from mesoderm [testis, heart, spleen, kidney, muscle, fat, uterus, and ovary], and 3 from ectoderm [brain, eyeball, and musk gland]) (Fig. 2A), with at least 6 biological replicates for each stage. We then updated the annotation of distinct transcript types, including long noncoding RNAs (lncRNAs) and microRNAs (miRNAs) (Supplementary Fig. S4a), representing a core atlas dataset of *de novo* assembled transcripts. Ten and 3 gland tissue–specific expressed lncRNAs and miRNAs were detected, respectively (Supplementary Fig. S4b).

After filtering the low expression levels genes with transcripts per million (TPM) <1 in at least 50% of samples in each analyzed tissue, we evaluated the expression levels of 14,861 (63.89%) muskrat genes and mainly looked for tissue-specific expression patterns. The results showed that the transcriptional profiles of each tissue type are highly reproducible among biological replicates (Spearman *r* > 0.80) (Fig. 2B). Ovary, musk gland, and uterus also clustered into obviously separate respective groups. Meanwhile, brain and eyeball tissues clustered together (Fig. 2C). More than 60% of genes were expressed in each tissue for muskrat (Supplementary Fig. S4c), but expression levels of different genes are skewed. In most tissues, the expression of highly expressed 1,000 genes takes up an average expression of more than 50%, especially for muscle (Fig. 2D). We also observed dissimilarities between the gene expression level distribution across tissues. The most abundant transcripts (the top 1,000, as ranked by expression levels) in a tissue accounted for greater than half of the total transcribed muscle (∼71.71%), liver (∼68.76%), and heart (∼61.34%), whereas testis (∼37.70%) had a more uniform distribution (Fig. 2E, Supplementary Fig. S4d). Testis showed the highest number of differentially expressed genes compared with other tissues (Supplementary Fig. S4e).

In terms of protein-coding gene expression, 3 (uterus), 35 (testis), 27 (spleen), 6 (ovary), 26 (muscle), 9 (lung), 47 (liver), 23 (kidney), 19 (heart), 10 (gland), 8 (fat), 38 (eyeball), and 112 (brain) tissue-specific genes were detected (Supplementary Table S11). The tissue-specific genes were commonly enriched in distinct cellular functions. For example, the specifically expressed genes for the musk gland were mainly involved in “epidermis development” and those for testis in “male gamete generation” and “meiotic nuclear division” (Supplementary Fig. S5). Notably, among the genes specifically expressed in musk gland (Fig. 2F), *KRT80* and *MPIG6B* genes were also PSGs in muskrat. Keratin 80 (*KRT80*) is an intermediate filament protein that contributes to the structural integrity of epithelial cells [24]. The megakaryocyte and platelet inhibitory receptor gene G6P (*MPIG6B*) regulates platelet production, aggregation, and activation [25]. In addition, we also found that some musk gland–specific genes are related to lipid metabolism, such as *TRPV3* and *LIPM* (Fig. 2F). Transient receptor potential (TRP) channels are polymodal sensors that convert a multitude of environmental cues into cellular signaling events essential for physiology [26]. *TRPV3* is activated by warm temperatures and numerous chemicals, including plant extracts, lipid metabolites, and synthetic small molecules such as 2-aminoethoxydiphenyl borate (2-APB) [27, 28]. In mice, *LIPM* (lipase) also has a restricted tissue expression in the epidermal tissue [29], supporting the unique and active function of *LIPM* in musk gland function.

### Single-cell reconstruction of musk secretion remodeling in the muskrat adult musk gland

**Figure 3:**
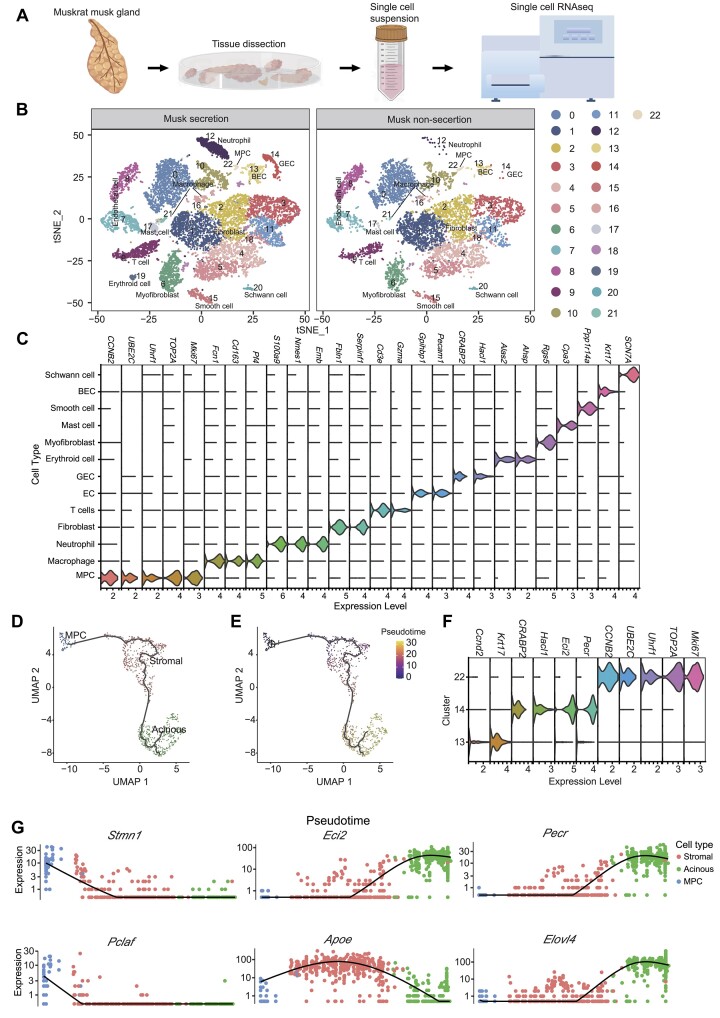
Musk gland single-cell transcriptome map of muskrats. (A) Schematic representation of muskrat musk gland tissue preparation for single-cell transcriptome analysis. (B) tSNE and UMAP cluster map revealing 23 specific clusters representing the major musk gland cell types. (C) Violin plots show the expression of representative differentially expressed genes for each cluster. (D) UMAP visualization of the muskrat mesenchymal progenitor cell (cluster 22), basal cell (cluster 13), and acinar cells/glandular epithelial cells (cluster 14). Cells are color-coded by cluster. (E) Cells are color-coded by pseudotime reconstruction traces. (F) Violin plot of genes selected as population markers for each of the 13, 14, and 22 clusters. (G) Genes that displayed divergent expression patterns during acinar cells/glandular epithelial cells’ generation in muskrat.

To depict transcriptomic changes during musk secretion at the single-cell level, we further measured the transcriptional differences between representative secretion and nonsecretion stages in the musk gland by the 10X Genomics scRNA-seq system (Fig. 3A). After quality filtering, the transcriptome profiles of 19,398 cells were available for cell-type characterization (12,128, and 7,270 cells for musk secretion and nonsecretion stages, respectively) (Supplementary Table S1). To explore the cell types of these musk glands, we performed the uniform manifold approximation and projection (UMAP) analysis and identified 23 cell clusters (Fig. 3B, C). We surveyed the expression patterns of the top 50 most variable genes (Supplementary Fig. S6a), which could cluster these cells into 13 known cell types (Supplementary Table S12).

We found that most collected cells (49.53% and 59.16%) possess characteristics typical of fibroblasts (clusters 1, 2, 3, 4, 5, 11, and 18) with higher expression of *IGFBP3* [30, 31], *DCN* [32], and *C3* [33] (Supplementary Fig. S6b). Clusters 0, 10, 16, and 21 were identified as macrophages with higher expression of *C1qa, C1qb, C1qc, Ctss, Cd14*, and *Cd68* [34]; cluster 21 had higher expression of *Coro1a* [34] and *Cd74* [35, 36]. Cluster 17 was identified as having mastocytes with expressed *Alox5, Cpa3, Kit*, and *Srgn* [37] gene markers. Cluster 22 expressed *Ccnb2, Hmgb2, Hmgb3, Mcm6, Ube2c*, and *Uhrf1* [38] mesenchymal progenitor cell (MPC) markers. In addition, a recent study [39] suggested that proliferative marker genes *Top2a, Mki67*, and *Birc5* were markers for cluster 22 in our study. Neutrophil granulocytes (cluster 12) highly expressed *Adam8, Arg2, Anxa1*, and *C5ar1* [35]. Endothelial cells (clusters 7 and 8) expressed markers of *Plvap, Cav1, Cav2, Emcn, Gpihbp1, Pecam1*, and *Tm4sf1* [34]. Myoepithelial cells (cluster 6) expressed gene markers of *Acta2, Myh11, Myl9, Mylk, Tpm*, and *De* [40, 41]. T cells (cluster 9) expressed *Rapgef6, Ltb, Rpl12, Rplp1, Rps16*, and *Rps23* [35] and *CD3* gene markers [42–45]. Basal epithelial cells (cluster 13) expressed *Ccnd2, Krt14*, and *Krt17* markers [46]. Acinar cells/glandular epithelial cells (GECs) (cluster 14) expressed *Cited4, Epcam, Crabp2*, and *phyh2* gene markers. Smooth muscle cells (cluster 15) highly expressed *Des, Myh11, Acta2*, and *Tpm2* [40, 47]. In addition, the *PPP1R14A* gene highly expressed in cluster 15 could inhibit the myosin phosphatase, lead to increased phosphorylation of myosin, and enhance smooth muscle contraction. Cluster 20 highly expressed *SOX1, S100* [48], *MPZ* [49], *NCAM* [50–52], *SCN7A*, and *CRYAB. SCN7A* is one of the many voltage-gated sodium channel proteins. *CRYAB* is highly expressed in many neurological diseases, and the protein encoded by the *S100B* gene might play a role in Ca^2+^ flux stimulation and promoting astrocyte hyperplasia. The chromosomal rearrangement or expression change of S100B is associated with neurological diseases such as Alzheimer disease, Down syndrome, and epilepsy. Thus, cluster 20 cells were defined as Schwann cells. Cluster 19 cells with highly expressed *Alas2, Bpgm*, and *Mkrn1* were identified as erythrocyte precursor cells [34].

Next, we focus on the cell clusters with increased cell abundance in the musk secretion stage (Supplementary Table S13), which included cluster 12, cluster 14, cluster 17, and cluster 21. Intriguingly, the differentially expressed genes (DEGs) in cluster 14 (Supplementary Table S14, Supplementary Fig. S6c) were involved in the “regulation of hormone levels,” “lipid biosynthetic process,” and “organic acid transport” pathways, which are related to musk secretion (Supplementary Fig. S6d). In addition, for the DEGs in cell cluster 14, we also determined that 5 genes overlapped with REGs, including those associated with carboxylic acid transport (*SLC26A2*), transcription factor activity (*ZNF317*), cell division (*NCKAP51*), and collagen-containing extracellular matrix (*GPC4* and *COL6A3*). It is worth mentioning that *NCKAP51* is also a REG of forest musk deer. Studies in closely related species have suggested that it targets mir-2425–5p to regulate the proliferation and differentiation of bovine myogenic satellite cells [53]. *GPC4* is differentially expressed in dental epithelial and mesenchymal cells [54], also expressed in renal epithelial cells to regulate epithelial branching morphogenesis [55]. *COL6A3* encodes collagen type VI and is usually expressed in tumor epithelial cells to promote invasion and metastasis [56]. Two PSGs, cyclin (*WEE1*) and integrin ligand (*NPNT*), coincided with the differential genes of GEC. *WEE1* can regulate cell division by mediating the G2/M phase progression of epithelial cells [57]. *NPNT* was found to be highly expressed in epithelial and mesenchymal cells of the tooth germ and regulated the differentiation of Sox2^+^ cells in dental epithelial cells through the EGFR-PI3K-Akt signaling pathway [58]. These genes provide evolutionary evidence for the epithelial–mesenchymal transition of the muskrat musk gland during musk secretion and nonsecretion.

### Pseudotime reconstruction traces of the origin and specification of acinar cells/glandular epithelial cells

Our scRNA results showed that acinar cells/glandular epithelial cells (cluster 14) have an increased number of cells in the musk secretion stage (with a relative abundance of 2.76% and 0.12% in musk secretion and nonsecretion stages, respectively), and this cell type is related to musk secretion. Thus, we further explored the origin and differentiation of the cells of this cluster. Acinar cells/glandular epithelial cells (cluster 14) expressed *Cited4, Epcam, Crabp2*, and *Phyh2* gene markers [34]. We used cell lineage trajectory analysis to elucidate the origin and differentiation of the acinar cells/glandular epithelial cells in muskrat. Pseudotime analysis based on transcript profiling enabled a precise reconstruction of acinar cells/glandular epithelial cells in the male muskrat gland.

The high concordance of scRNA status between cluster13 and cluster14 indicated that muskrat acinar cells/glandular epithelial cells derive directly from the basal cells, and the basal cells derive directly from the mesenchymal progenitor cell (Fig. 3D, E). This scenario on the biological origin of glandular epithelial cells was consistent with the scRNA of mouse mammary epithelial cells [59]. The markers for acinar cells/glandular epithelial cells (*CRABP2, Hacl1, Eci2*, and *Pecr*) of cluster 14, found by pseudotime reconstruction, displayed high and specific expressions compared with the other 2 clusters (Fig. 3F, G). These genes were mainly involved in lipometabolic functions. For example, *CRABP2* is involved in the metabolism and transportation of retinoic acid from the cytosol to the retinoic acid receptors (RARs) located in the nucleus [60]. *Hacl1* is an enzyme that catalyzes the hydrolysis of long-chain fatty acids [61]. Enoyl-CoA delta-isomerase 2 (*ECI2*) is a protein that catalyzes the isomerization of unsaturated fatty acid intermediates during β-oxidation, a process that breaks down fatty acids to produce energy [62]. Peroxisomal trans-2-enoyl-CoA reductase (*PECR*) is a protein that plays a role in the metabolism of fatty acids, specifically by reducing unsaturated and polyunsaturated fatty acids to their saturated forms in peroxisomes [63]. A previous study showed that muskrat musk contained fatty acids (29.32%) by gas chromatography–mass spectrometry, which are the main components of musk [64]. Combined, these results indicated that the function of lipometabolism is important in the development and evolution of the musk gland.

### Dynamic changes in compartmentalization, topologically associating domains, and promoter–enhancer interactions for musk secretion

**Figure 4:**
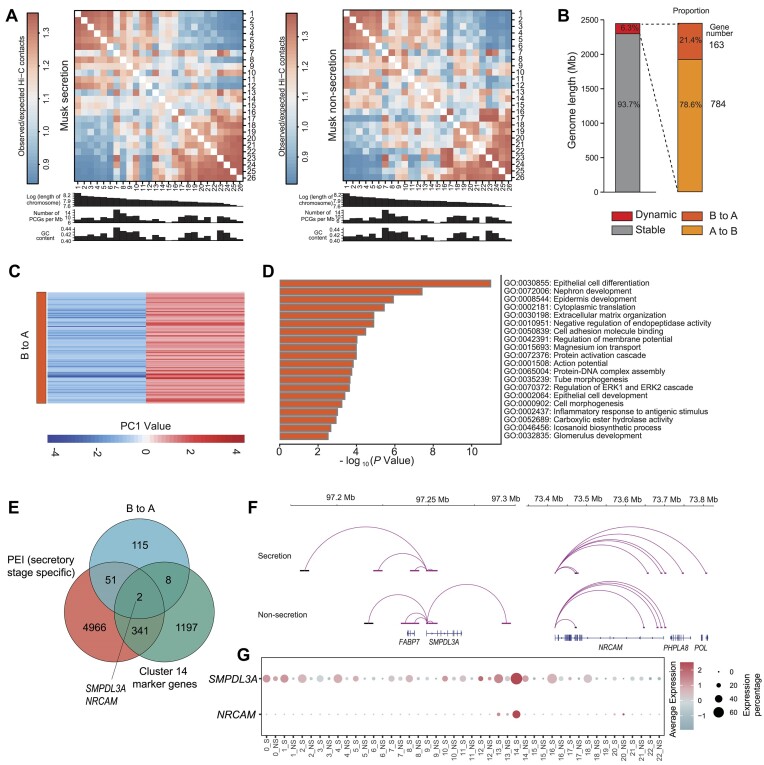
Global chromatin interaction patterns in musk gland of musk secretion and nonsecretion stages. (A) Observed/expected contact matrices between chromosome pairs. Also shown are the length, gene density, and GC content of each chromosome. (B) Genomic lengths and proportions of stable and dynamic compartments. Dynamic compartments are classified into 2 types of transitions (A to B and B to A). (C) Heatmap of the PC1 values for the compartment B to A switching regions. (D) The most enriched GO-BP terms for genes within B to A switching regions. (E) Venn diagram showing overlapping genes with compartment B to A, PEI specific to musk secretion stage, and marker genes in cluster 14. (F) PEIs rewired in the musk gland of musk secretion and nonsecretion stages of *SMPDL3*A and *NRCAM*. (G) Gene expression of *SMPDL3*A and *NRCAM* in each cluster at musk secretion and nonsecretion stages.

In the eukaryotic cell nucleus, genomic DNA is highly folded and spatially organized into a hierarchy of 3-dimensional structures, including chromosome territories, compartments, topologically associating domains (TADs), and long-range interactions [65], which play important roles in transcriptional regulation [66]. To elucidate the multiscale regulatory rewiring of chromatin architecture during musk secretion, we used an *in situ* Hi-C to map chromatin contacts for musk glands between the secretion and nonsecretion stages. We generated a total of ∼2.77 billion valid contacts (∼692.44 million [M] contacts per sample) (Supplementary Table S15) and reached a maximum resolution of 5 kb by merging the intrachromosomal contacts of the replicates at each stage (Supplementary Table S16). Most (∼54.29%) contacts occurred within chromosomes, exhibited high reproducibility among the biological replicates, and consisted dominantly (∼57.82%) of long-range interactions (≥20 kb) (Supplementary Fig. S7a–d). All samples showed a strong decrease in contact probability with increased distance between loci (Supplementary Fig. S7e). All samples showed similar A/B compartment patterns; ∼44.4% and ∼48.0% of the whole genome were compartment A bins for musk secretion and nonsecretion periods, respectively (Supplementary Fig. S7f). Compartment A was positively correlated with guanine–cytosine content (Spearman *r* > 0.60, *P* < 2.20 × 10 ^–16^) (Supplementary Fig. S7g, h) and has a high gene density (Supplementary Fig. S7i). We then constructed genome-wide interchromosomal contact maps by dividing the genome into 500-kb regions; it was revealed that the muskrat chromosomes have a similar likelihood to mutually contact each other during development: micro- and macrochromosomes tended to be self-associated, and small and gene-rich chromosomes preferentially contacted with each other more frequently (Fig. 4A). A total of 2,969 and 3,438 TADs were subsequently detected in musk secretion and nonsecretion stages in the musk gland, with a median size of ∼500 and 575 kb, respectively (Supplementary Fig. S7j). We observe that only 54% (2,256) of the positioning of TADs remains stable between the 2 stages (Supplementary Fig. S7k). We also compiled an extensive genome-wide catalog of promoter–enhancer interactions (PEIs) in musk gland tissue at a 5-kb resolution. The median sizes were ∼100 and ∼60 kb (54.67% and 63.11% PEIs existed primarily in TADs) for musk secretion and nonsecretion stages, respectively (Supplementary Fig. S8a–c). We observed that ∼87.98% of enhancers interacted with a more distant promoter instead of those closer by (Supplementary Fig. S8d). This spatial proximity data highlight the complexity of PEIs [67].

Next, we compared the 3-dimensional genome differences in the musk gland between the 2 stages. At the subchromosome level, we identified a substantial number of regions showing compartmental switching in the musk gland between 2 stages (∼153.6 Mb, or ∼6.2% of the genome) (Fig. 4B). In these regions, most switching was from A to B (120.80 Mb, embedded with 781 genes), which indicated that these regions were more closed in the musk secretion stage compared with the nonsecretion stage. The rest were transient switches, from B to A (32.8 Mb, embedded with 164 genes) (Fig. 4B, C). Because these active chromatin regions are of potential functional significance, we further checked the genes in areas subject to B-to-A switching events. They were primarily involved in “epithelial cell differentiation,” “nephron development,” “epidermis development,” “cytoplasmic translation,” “extracellular matrix organization,” “negative regulation of endopeptidase activity,” “cell morphogenesis,” “protein activation cascade,” “epithelial cell development,” “intracellular steroid hormone receptor signaling pathway,” and “regulation of membrane potential” processes (Fig. 4D).

At the TAD structure level, many changes in chromatin structure occur. We used the insulation score (IS) to evaluate the overall extent of changes in chromatin conformation between the 2 stages. For the TAD with increased IS in the musk secretion stage, the content genes were involved in the “regulation of proteolysis,” “response to radiation,” “regulation of lipid metabolic process,” and “transcription coregulator activity” pathways (Supplementary Fig. S9a), which indicated the active function of regulating lipid metabolism in the musk secretion stage. Furthermore, the PSGs or convergent evolution genes (Supplementary Fig. S9b) involved in TAD changing were also related to the metabolism of lipids and epithelial regulation pathways, such as *NAGS* catalyzing the production of N-acetylglutamate (NAG). This vital substance regulates urea synthesis [68]. *CEP250* plays a crucial role in differentiating spermatogonia and meiotic spermatocytes [69]. *CASP8AP2* plays a role in regulating cell proliferation, apoptosis, and gene expression [70].

At the PEI level, we determined that 2 genes (*SMPDL3A* and *NRCAM*) with specific PEI in the musk secretion stage also showed a compartment transition from B to A and was a marker gene in cluster 14 (Fig. 4E). There were more specific long-range interactions (>25 Kb) in the musk secretion stage for these 2 genes (Fig. 4F). The gene *SMPDL3A* showed a relatively higher expression in all clusters at the musk secretion stage (Fig. 4G). *SMPDL3A* (sphingomyelin phosphodiesterase acid-like 3A) is an enzyme induced by lipid metabolism through the liver X receptor that degrades cGAMP, modulating the cGAS-STING pathway, which is involved in immune responses and lipid sensing [71]. *NRCAM* (neuronal cell adhesion molecule) is primarily associated with neural development; its expression also promotes malignant cell transformation, cell motility, and metastatic disease [72]. In addition, *MAP3K1, NODAL*, and *Slc38a2* also showed significantly more PEIs and were contacted with more enhancers during the musk secretion stage (Supplementary Fig. S10). *MAP3K1* is a critical component of the protein kinase signal transduction cascade and plays a crucial role in cellular signaling pathways [73]. *NODAL* maintains stem cell pluripotency and promotes directed differentiation [74]. *Slc38a2* encodes an amino acid transport protein facilitating cellular uptake of amino acids [75]. These results suggest that 2 essential functions of the musk gland during the musk secretion stage are lipid metabolism and cell specialization, which indicate that synthesis and secretion activity are very active at this stage. Our results demonstrated that candidate loci can be analyzed in future studies of musk secretion mechanisms.

### The MuskDB platform

**Figure 5:**
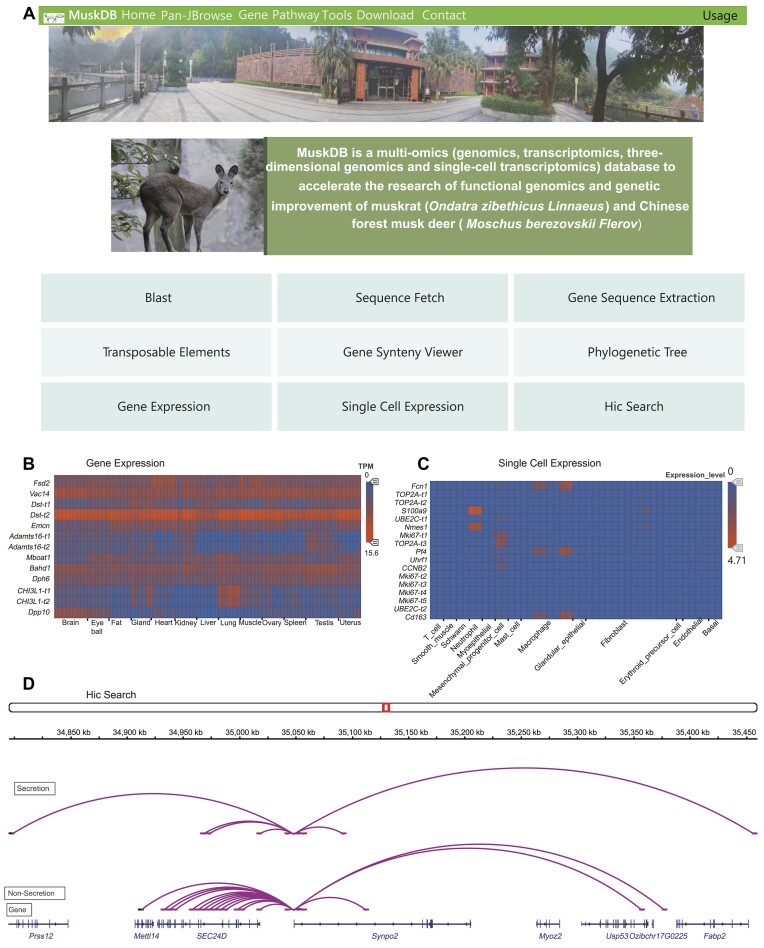
Overview of MuskDB and its application in musk secretion animals’ functional genomics. (A) MuskDB homepage contents show available online tools. (B) The expression pattern of genes in different tissues is shown by MuskDB. (C) The expression pattern of genes in various cell clusters is shown by MuskDB. (D) Hi-C contacts of the gene *Synpo2* in musk secretion and nonsecretion stage shown by MuskDB.

According to the sequencing data described above, we generated a database (MuskDB). MuskDB contains 47,635 gene entries from the genomes of 2 species, with information on 831 biological pathways, 103 bulk RNA transcriptomes, 2 single-cell transcriptomes, and 3 Hi-C datasets. The platform also holds 3 Hi-C data sets (secretion stage in July and nonsecretion stage in October for the muskrat musk gland and blood sample of musk deer), including 343,392 promoter and enhancer interactions. On the homepage, the tools include “Blast,” “Sequence Fetch,” “Gene Sequence Extraction,” “Transposable Elements,” “Gene Synteny Viewer,” “Phylogenetic Tree,” “Gene Expression,” “Single Cell Expression,” and “Hic Search” (Fig. 5A). MuskDB provides comprehensive information on muskrat and musk deer genes, including their annotation, location, and expression. The heatmaps in “Gene Expression” and “Single Cell Expression” show the expression of genes, respectively, in 13 tissues (Fig. 5B) and 13 different cell types (Fig. 5C). In “Hic Search,” users can enter a gene name or genome region to show the contact information of this gene or in this region. For example, when the gene *Synpo2* is entered, the results display the contact profile of this gene at the stages of secretion and nonsecretion (Fig. 5D).

## Conclusion

This study provided an open-source, web-accessible, user-friendly multiomics database platform of musk secretion animals, including 2 high-quality genome assemblies of musk secretion species (muskrat and musk deer), a BodyMap transcriptome of muskrat, and a comprehensive analysis of Hi-C, RNA-seq, and scRNA-seq between musk secretion and nonsecretion stages of muskrat *in vivo*. GO terms of stage-specific signature genes of musk glands were identified by scRNA-seq and emphasized significant functional differences between the musk secretion and nonsecretion stages. Because of their unique evolutionary adaptations, the muskrat and musk deer have always been a topic of interest in animal evolution and physiology. Our high-quality genome assembly provides more precise comparative insights into the genetic basis of their biological features. We identified genetic changes underlying adaptations of both species to musk secretion, notably related to the adaptation of lipid metabolism, cell cycle regulation, and sensory perception. Our single-cell RNA sequencing results may potentially facilitate the development of new strategies for the organoid culturing of the musk gland. These results show that active lipid metabolism may underlie the adaptation evolution of musk secretion. However, because of the complexity of the musk gland, more research on the functional consequences of musk secretion–specific genetic variants is needed.

## Materials and Methods

### Methods

#### Sample collection

For genome sequencing, blood from a male muskrat and a male forest musk deer was collected from the Chongqing Institute of Medicinal Plant Cultivation. To fully investigate the muskrat transcriptome, we used 84 samples from 13 tissues (3 to 6 samples from each of the 13 tissues were collected). The healthy muskrats were euthanized with isoflurane; we adjusted the isoflurane flow rate or concentration to 5% or greater and continued isoflurane exposure until 1 minute after the muskrat stopped breathing. The required tissues were then collected using standard anatomical techniques, and excess blood vessels and fat were removed in phosphate-buffered solution and then flash-frozen in liquid nitrogen and stored in a −80°C refrigerator for subsequent studies.

#### Genome sequencing

Blood high-quality DNA was extracted from 20 mL of muskrat and forest musk deer blood using a DNeasy Blood & Tissue Kit (Qiagen) according to the manufacturer’s instructions. Some of this DNA was then used to construct Nanopore libraries, which were sequenced using GridION X5 sequencers (Oxford Nanopore Technologies), and the remainder of the DNA was used to construct resequencing (NGS) libraries with an insert size of 400 bp, which were sequenced using the BGI T7 platform. The musk gland samples at musk secretion and nonsecretion stages were used to construct Hi-C libraries, which were subsequently sequenced using the Illumina NovaSeq 6000 platform (RRID:SCR_016387).

#### Genome assembly and assessment

We used an optimized 4-step genome assembly strategy [8] to generate complete assemblies of muskrat and musk deer genomes. First, we used the high-quality Nanopore sequences and applied a “correct-then-assemble” strategy using NextDenovo (v2.5.0; RRID:SCR_025033) [76] to assemble the initial contigs. To correct the initial contigs, we utilized the software NextPolish (v1.4.1; RRID:SCR_025232), employing high-quality T7 paired-end reads and Nanopore long reads, and applied the recommended algorithm modules “best.” in Nextpolish [77]. The specific parameters and pipelines used in the assembly process are available at Zenodo [78]. Thus, we yielded the ContigV1 assembly. Second, we generated unique mapped pairs by aligning the Hi-C read pairs to ContigV1 using Bowtie2 software (RRID:SCR_016368) [79] with a single-ended model. We then discarded invalid self-ligated and unligated fragments using the HiCUP pipeline (version 0.8.0; RRID:SCR_005569) [80]. We obtained the valid interaction pairs and calculated linkage frequency among all contigs using an agglomerative hierarchical clustering algorithm. The linked contigs were clustered based on the Hi-C signal density, indicating potential homologous chromosome associations. Third, we realigned the Nanopore reads to ContigV1 using the Minimap2 package (RRID:SCR_018550) [81]. Suboptimal alignment reads were removed, and mapped reads of each contig group were extracted. Local assembly was performed for each classified mapped read to avoid false overlap relationships caused by repetitive sequences during assembly [82]. All contigs were corrected again using appropriate parameters, similar to the first step. Fourth, chromosome-scale genomes were anchored using linkage information, restriction enzyme site, and string graph formulation with the ALLHiC algorithm (RRID:SCR_022750) [83]. Any placement and orientation errors displaying distinct chromatin interaction patterns were manually adjusted.

To assess the quality of genome assemblies, we used the Merqury package to evaluate assembly precision by measuring QV values [10]. BUSCO analysis (version 5.2.1; RRID:SCR_015008) [9] was used to assess the assembly completeness by searching against 9,226 conserved mammalian genes from the mammalia_odb10 database. Additionally, T7 paired-end reads were aligned to the assembled genome using BWA software (RRID:SCR_010910) [84] to calculate the alignment ratio and coverage depth for assessing the assembly completeness.

#### TE annotation

We predicted the genome TEs by combining homology searching and *ab initio* prediction methods. We performed the homology searching by applying the RepeatMasker (RRID:SCR_012954) [85] and RepeatProteinMask packages to compare the genome against the Repbase TE library. In parallel, we performed *ab initio* prediction by constructing a reference repeat library using the results from PILER (RRID:SCR_017333) [86], LTR_FINDER (RRID:SCR_015247) [87], and RepeatScout (RRID:SCR_014653) [88]. Then, we searched the genome against this library using RepeatMasker. In addition, we employed the Tandem Repeats Finder package (RRID:SCR_022065) [89] with specific parameters (“2 7 7 80 10 50 2000 -d –h”) to predict tandem repeats in the genome.

#### Protein-coding gene prediction

We predicted protein-coding gene models via integration of homology- and *ab initio–*based methods, with additional evidence from transcription data. In the homology-based approach, we used protein repertoires from model mammalian species such as *Homo sapiens* (GCA_000,001,405.28), *Mus musculus* (GCA_000,001,635.8), *Equus caballus* (GCA_002,863,925.1), *Canis lupus familiaris* (GCA_000,002,285.2), *Rattus norvegicus* (GCA_015,227,675.2), *Sus scrofa* (GCA_000,003,025.6), and *Bos taurus* (GCA_002,263,795.2) as queries. These queries were searched against the target genome using the TBLASTN algorithm (RRID:SCR_011822) [90]. The resulting BLAST hits were conjoined using the Solar (Sorting Out Local Alignment Results) to obtain a comprehensive set of alignments. Next, the gene structures within each BLAST hit were determined using the GeneWise pipeline (RRID:SCR_015054) [91], allowing us to define gene models with high accuracy and specificity. Subsequently, we obtained transcriptomic data for muskrats and musk deer from the NCBI database, with accession numbers listed in Supplementary Table S1. The RNA-seq data were aligned to the genome using the Tophat software (RRID:SCR_013035) [92], enabling the identification of potential exonic regions and splicing junctions. Gene models, represented by the Cufflinks-set, were assembled from the mapped reads using Cufflinks (RRID:SCR_014597) [93]. In the *ab initio* method, we aligned the assembled transcripts with the assembled genome using the Program to Assemble Spliced Alignment (PASA) (RRID:SCR_014656) [94]. This allowed us to assemble the transcript alignments into gene structure models used as the training set for Augustus (RRID:SCR_008417) [95], SNAP (RRID:SCR_007936) [96], and GlimmerHMM (RRID:SCR_002654) [97] pipelines. With these training sets, we conducted *ab initio* prediction of coding regions in the repeat-masked genome using Augustus, GlimmerHMM, and SNAP. Furthermore, we employed GeneID (RRID:SCR_021639) [98] and GeneScan (RRID:SCR_023400) [99] to directly generate predicted gene models in the repeat-masked genome. After completing the aforementioned methods, the generated gene models were integrated using EVidenceModeler (RRID:SCR_014659). We assigned weights to each type of evidence as follows: Homology-set > Cufflinks-set > Augustus > GeneID = SNAP = GlimmerHMM = GeneScan. Furthermore, we used PASA2 to update the gene models, incorporating untranslated regions and information on alternative splicing variations.

To annotate the protein-coding genes, we searched for functional motifs, domains, and information on the possible biological processes of the genes in established databases such as SwissProt (RRID:SCR_021164) [100], the NR database (from NCBI), and the KEGG (RRID:SCR_012773) [101].

#### Identification of one-to-one orthologous genes

In addition to the gene sets generated from our 2 assembled genomes, we also downloaded gene sets from 10 other mammalian genomes, including human, macaque, mouse, horse, cattle, sheep, pig, cat, dog, and rabbit, from the Ensembl database (RRID:SCR_002344). We used these 12 gene sets to identify orthologous genes. To accomplish this, we first selected the longest translation to represent each gene and filtered our genes with fewer than 50 amino acids. Then, we performed an all-against-all BLASTP (RRID:SCR_001010) comparison with an E-value threshold of 1e-7 to determine similarities between genes across the 12 species. We extracted alignment pairs from each pair of genomes while restricting a maximum of 5 hits per protein sequence. These alignment pairs were used as input for the MCScanX algorithm (RRID:SCR_022067) [102], which helped detect collinear blocks of coding genes and identify orthologous gene pairs with high confidence. We specifically focused on one-to-one orthologous genes between pairs of mammalian species.

After integrating a matrix of orthologous genes for the 12 mammalian species, we ensured each orthologous cluster included all the species. Subsequently, we performed multiple sequence alignment for these one-to-one orthologs using PRANK (v.170427; RRID:SCR_017228) [103]. We applied the Gblocks package (v0.91b) (RRID:SCR_015945) [104] to minimize the impact of alignment errors and divergent regions. Alignments shorter than 90 nucleotides were discarded to maintain quality. Through this process, we identified 7,409 one-to-one orthologs among the 12 species.

#### Phylogeny construction and divergence time estimation

We initially used MODELTEST [105] to analyze the codon alignments of one-to-one orthologs and determined that the general time-reversible (GTR) substitution model was the most suitable for the observed data. Therefore, we conducted a phylogenetic tree for the 12 mammals using the maximum likelihood method implemented in the RaxML package (RRID:SCR_006086) [106]. The best-fitting substitution model “GTR+GAMMAX” was utilized, and 1,000 bootstrap replicates were performed to assess the robustness of the tree topology. To estimate divergence times, we employed the MCMCTree program from the PAML package (version 4.9; RRID:SCR_014932) [12].

#### Gene family clustering, expansion, and contraction analysis

We utilized the OrthoFinder package (v2.3.1; RRID:SCR_017118) [107] to identify gene families by detecting orthogroups and paralogous genes based on the results of the all-against-all BLASTP analysis (see also the method “identification of one-to-one orthologous genes”). The expansion and contraction of gene families were evaluated by comparing cluster sizes between the ancestral species and each of the 12 mammalian species. This analysis used the Café program (RRID:SCR_005983) [108], which employs a probabilistic graphical model. Using conditional likelihoods as test statistics, we calculated *P* values for each lineage and set a threshold of *P* < 0.05 to determine gene families that exhibited significant expansion or contraction.

#### Identification of PSGs and REGs

We employed the CodeML program in the PAML package (version 4.9; RRID:SCR_014932) [12] to identify PSGs and REGs based on the 7,409 orthologous genes. For PSGs, we utilized the free-ratio branch-site mode (model = 1) as an alternative model, assuming positive selection on the foreground branch. The null model allowed sites to undergo purifying selection or evolve neutrally. For REGs, we utilized the branch model, specifically the 1-ratio model (model = 0) as the null model, assuming the same evolutionary rate for all branches, and the 2-ratio model (model = 2) as an alternative model, allowing different evolutionary rates for the foreground branch. The likelihood ratio test (LRT) method was used to detect differences between the nested models, and *P* values were computed based on χ^2^ statistics. Multiple testing was corrected using the FDR method.

#### Convergent evolution among musk secretion animals

To test for convergence among musk secretion animals (muskrat and musk deer), we used 12 mammals from Fig. 1B. Based on the phylogenetic tree, we used 2 methods to detect the convergent amino acid substitutions for each node of the 7,409 single-copy orthologs: (i) in the method by Zhang and Kumar [13] and Zou and Zhang [14], a site was assumed as a convergent site if amino acids of a focused node at that site were the same but different with their most recent ancestral amino acids. Amino acid sequences of internal nodes for all the 7,409 single-copy orthologs were reconstructed by CODEML in PAML. For each gene, the number of observed convergent site was compared with the neutral expectations derived from the JTT-fgene model, and the Poisson test was then used to evaluate the difference. (ii) With the CCS method [15], the convergent signal was identified when both musk secretion species (muskrat and musk deer) shared the same derived character at a conservative site.

#### Functional enrichment analysis

The gene set enrichment analyses, including GO, KEGG pathway, and Reactome analyses, were performed using the KOBAS 3.0 software (RRID:SCR_006350) [109, 110], with human homologs as references. The statistical significance of enrichment was assessed using the binomial distribution test, and the *P* values were adjusted for multiple testing using the Benjamini method.

#### Muskrat transcriptome reconstruction

Total RNA was extracted from each sample using RNAiso Plus reagent (TaKaRa, #9108) according to the manufacturer’s instructions. We estimated the integrity and quality of the total RNA using a Bioanalyzer 2100 system (Agilent Technologies; RRID:SCR_018043) and an RNA 6000 Nano kit. Eighty-four poly-A RNA-seq libraries were constructed. LncRNAs were then sequenced using the NOVAseq-6000 platform with a paired-end sequencing length of 150 bp (PE150) at Shenggong Bioengineering. MicroRNA was sequenced by the NEXTSEQ550 platform (RRID:SCR_016384) with a single-end sequencing length of 75 bp (SE75) at Shenggong Bioengineering. In total, we then generated a total of 1.37 Tb high-quality RNA-seq data (∼16.33 Gb sequences per sample) and 27.13 Gb high-quality miRNA data (∼0.32 Gb sequences per sample) (Supplementary Table S1).

Clean reads were obtained after quality control filtering. Using FASTX-Toolkit software (RRID:SCR_005534) to remove the clean and low-quality reads (QV <30 bases accounted for more than 20%). The lncRNA sequenced reads were aligned to our self-assembled muskrat genome by the STAR alignment tool (version 2.6.0; RRID:SCR_005622), with, on average, ∼97% (∼57.06 million) of aligned reads for each library. Read counts were quantified using featureCounts (version 2.0.1; RRID:SCR_012919). Gene-level transcript abundance was estimated as TPM.

For miRNA analysis, human, mouse, and rat miRNA and miRNA precursor sequences were downloaded from miRBase (Version 22.1; RRID:SCR_003152). The software miRdeep2 (version 0.1.2; RRID:SCR_010829) was used to first index the muskrat genome, and then the data we had initially processed were converted into the format required by the software and compared with the muskrat genome. Finally, the sequences were compared with known miRNA sequences and miRNA precursor sequences. Combined with the position of genome alignment, the matching degree was calculated to form the minimum free energy of stem-ring miRNA to identify whether it was a muskrat miRNA. The prediction of new miRNA was similar. Mature miRNAs of muskrat relatives, such as humans, mice, and rats, were selected as a reference, and mirdeep2.pl of the software was used for prediction. The screening criteria for new miRNA should meet the miRDeep2 Score25 and have a secondary structure with *P* < 0.05 as candidate miRNA. Then, the information of new miRNA predicted by all samples should be counted, and the candidate new miRNA predicted by at least 2 samples should be considered new miRNA. The number of miRNA reads between samples was counted by TPM (tags per million), and its expression was calculated.

#### Gene transcriptional profiling across tissues

We calculated the tissue specificity of gene abundance reflected by the tau score (τ) (ranging from 0 to 1, with 1 for highly tissue-specific genes and 0 for ubiquitously transcribed genes) for each gene with scaled TPM values [111]. For each tissue, we averaged all replicates and then calculated τ to account for unequal numbers of replicates among tissues. We used τ ≥ 0.75 as the cutoff for tissue-specific genes. We calculated the abundance distribution (i.e., transcriptome complexity) of distinct transcripts across tissues, reflected as the fraction of total RNAs contributed by the most highly expressed genes. Differential gene expression analysis was performed using edgeR (version 3.40.2; RRID:SCR_012802) [112], with a Benjamini and Hochberg adjusted *P* value ≤0.01 and log_2_(fold change) ≥1 as cutoffs for statistical significance.

#### Single-cell preparation

After harvesting, musk gland tissues were washed in ice-cold RPMI1640 and dissociated using Demonstrated_Protocol_Adult_Mouse_Nuclei_Isolation_RevA (10X Genomics Catalog No. CG000393 Rev A) from Miltenyi Biotec as instructions. DNase treatment was optional according to the viscosity of the homogenate. Cell count and viability were estimated using a fluorescence Cell Analyzer (Countstar Rigel S2) with AO/PI reagent after the removal of erythrocytes (Miltenyi 130–094-183). Then, debris and dead cell removal was performed or not (Miltenyi 130–109-398/130–090-101). Finally, fresh cells were washed twice in the RPMI1640 and then resuspended at 1 × 10^6^ cells per mL in 1× phosphate-buffered saline and 0.04% bovine serum albumin.

#### Single-cell RNA-seq library construction and sequencing

ScRNA libraries were prepared using Chromium Next GEM Single Cell 3ʹ Reagent Kits v3.1 (10X Genomics). Briefly, the appropriate number of cells was mixed with reverse transcription reagent and then loaded to the sample well in Chromium Next GEM Chip G. Subsequently, Gel Beads and Partitioning Oil were dispensed into corresponding wells separately in the chip. After emulsion droplet generation, reverse transcription was performed at 53°C for 45 minutes and inactivated at 85°C for 5 minutes. Next, cDNA was purified from a broken droplet and amplified in the PCR reaction. The amplified cDNA product was then cleaned, fragmented, end-repaired, A-tailed, and ligated to the sequencing adapter. Finally, the indexed PCR was performed to amplify the DNA representing the 3′ polyA part of expressing genes, which also contained a cell bar code and a unique molecular index. The indexed sequencing libraries were cleaned with SPRI beads, quantified by quantitative PCR (KAPA Biosystems KK4824), and then sequenced on an Illumina NovaSeq 6000 with PE150 read length.

#### Single-cell RNA sequencing data quality control

Fastp (v0.20.1; RRID:SCR_016962) [113] was used to trim primer sequence and low-quality bases of raw reads and collect the basic statistics. The specific parameters could be summarized as follows: (i) A 4-bp sliding window was moved from the front (5′) to tail. Once the mean quality of the bases in the window was below 10, the bases, along with the subsequent bases, were dropped, and the leading N bases were also trimmed (–cut_front –cut_front_window_size 4 –cut_front_mean_quality 10). (ii) A 1-bp sliding window was moved from tail (3′) to front. The bases in the window were dropped if their mean quality was below 3, and the trailing N bases were also trimmed, similar to the Trimmomatic TRAILING method (–cut_tail –cut_tail_window_size 1–cut_tail_mean_quality 3). (iii) The auto adapter was detected for PE data (detect_adapter_for_pe). (iv) The trimmed reads shorter than 60 bp were discarded (–length_required 60). The cleaned reads after trimming were used in the following steps.

#### Processing the single-cell RNA sequencing data

The Cell Ranger (RRID:SCR_017344) [114] Single-Cell Software Suite performed sample demultiplexing, barcode processing, and single-cell 3′ gene counting. The single-cell data from the 2 stages were analyzed jointly.

The clustering and visualization were finished by Seurat (RRID:SCR_007322) [115], with the following steps: (i) Data normalization. LogNormalize, a global-scaling normalization method, was employed to normalize the expression. The expression measurement of 1 transcript was divided by those of all the transcripts of the cell and multiplied by a scale factor (10,000 by default), and then the result was logarithmically transformed. (ii) Detection of highly variable features. FindVariableFeatures was used to get 2,000 features per dataset. (iii) Scaling. A linear transformation (“scaling”), a standard preprocessing step before dimensional reduction techniques, was applied. (iv) Dimensional reduction. PCA (principal component analysis) on the scaled data was performed, and the first 15 principal components were used in the following steps. (v) Clustering. A graph-based approach was applied to cluster the cells. (vi) tSNE/UMAP. The nonlinear dimensional reduction technique was used to visualize and explore these datasets. (vii) Cluster markers. FindAllMarkers with the default parameters except “logfc.threshold=1” was used to find markers that determined the cell clusters via the differential expression, and the top 9 markers were visualized.

#### Pseudotime analysis

In R, utilizing the Monocle3 software package (v1.3.1; RRID:SCR_018685) [ 116], pseudotime trajectories for all 13, 14, and 22 subpopulations in the musk gland were constructed. This technique sorts individual cells along their developmental paths based on how closely their gene expression patterns match those of other sequenced cells, effectively mapping out the dynamics of cellular changes [117, 118]. Furthermore, the method evaluates how genes work together in space by using Moran’s index to measure their coexpression, which helps to establish a timeline of gene expression changes.

#### Identification of DEGs in cluster 14 cells

DEGs in the cluster 14 cells of musk secretion and nonsecretion stages were identified using the FindMarkers function in Seurat. Genes meeting the thresholds (|log_2_FC| > 1, *P*-adjusted < 0.05, min.pct = 0.25) were considered DEGs. The results were visualized using dot plots.

### Hi-C data of musk gland tissue

#### Hi-C data processing

We utilized Juicer (RRID:SCR_017226), an efficient open-source tool [119], to process the Hi-C datasets. Initially, we aligned the high-quality Hi-C reads to the genome using the BWA-mem module. Subsequently, we eliminated abnormal, duplicate, and low-quality alignments (MAPQ < 30). We constructed a normalized contact matrix using the KR algorithm at different resolutions, including 5 Kb, 25 Kb, 100 Kb, 500 Kb, and 1 Mb.

#### Resolution evaluation of the Hi-C matrix

To determine the optimal resolution for our Hi-C matrix, we divided the genome into window sizes ranging from 1 Kb to 1 Mb. For each bin, we counted the number of *cis* contacts, defined as any contact where 1 read mapped within that bin, and calculated the percentage of bins with contacts greater than 1,000. We identified the minimum window size with a percentage greater than 80 as the optimal resolution for our Hi-C matrix.

#### Identification of compartment A/B at the resolution of 100 Kb and 25 Kb

At a resolution of 100 Kb, compartment A/B analysis was performed as previously described. Briefly, a Pearson correlation matrix was generated using the “cor” function in R. The first 3 principal components were obtained by applying the “prcomp” function in R to the correlation matrix. Bins at 100 Kb with a positive Spearman correlation between PC1 values and gene density were classified as compartment A, while bins with a negative correlation were classified as compartment B.

For compartment A/B identification at a resolution of 25 Kb, the A–B index value was used as previously described, representing the comparative likelihood of a sequence interacting with A or B at a resolution of 100 Kb. Bins at 25 Kb with positive values (indicating a greater association with A at 100 Kb) were identified as A compartments, while bins with negative values (indicating a greater association with B at 100 Kb) were identified as B compartments.

#### Identification of interchromosome interaction pattern

The patterns of interchromosome interactions were calculated following the previous protocol [120]. In brief, the observed number of contacts was normalized against the expected contacts in each interchromosome pair.

#### Identification of TAD

We identified TADs from the normalized contact matrix at a resolution of 25 Kb. We used the directionality index score and a hidden Markov model algorithm implemented in the TADtool software with default parameters [121] to assign TAD boundaries.

#### PEI analysis

We first combined the clean data of biological replicates and constructed the normalized contact matrix at a 5-Kb resolution. We then used PSYCHIC software to generate raw PEIs [122]. We then filtered low-confidence PEIs with interaction distances lower than 10 Kb or FDR greater than 0.001.

## Availability of Source Code and Requirements

**Project name:** Paper_scripts_muskrat_muskdeer

**Project homepage:** https://github.com/YMSen/Paper_scripts_muskrat_muskdeer

**License:** MIT license

**Softwareheritage PID:** swh:1:snp:9702a4beb9ada324e96485cc8e7b985b40319d72;origin=https://github.com/YMSen/Paper_scripts_muskrat_muskdeer [123].

## Supplementary Material

giaf006_Supplemental_Files

giaf006_GIGA-D-24-00205_Original_Submission

giaf006_GIGA-D-24-00205_Revision_1

giaf006_GIGA-D-24-00205_Revision_2

giaf006_Response_to_Reviewer_Comments_Original_Submission

giaf006_Response_to_Reviewer_Comments_Revision_1

giaf006_Reviewer_1_Report_Original_Submission Dongya Wu -- 8/14/2024

giaf006_Reviewer_1_Report_Revision_1 Dongya Wu -- 11/4/2024

giaf006_Reviewer_2_Report_Original_Submission Yu Wang -- 8/19/2024

giaf006_Reviewer_2_Report_Revision_1 Yu Wang -- 10/16/2024

giaf006_Reviewer_3_Report_Original_Submission Qingyou Liu -- 8/22/2024

## Data Availability

The sequencing data for this project have been deposited in the NCBI (National Center for Biotechnology Information) and can be accessed with BioProject Nos. PRJNA996334, PRJNA992196, PRJNA992194, PRJNA985369, PRJNA985371, PRJNA992197, PRJNA1132163, and PRJNA1197571. Musk deer transcriptomic sequencing data were reused from BioProject Nos. PRJNA289641, PRJNA928235, PRJNA790817, PRJNA291827, and PRJNA289642. All supporting data and materials are available in the *GigaScience* database, GigaDB [124–126].

## Additional Files

**Supplementary Fig. S1**. Karyotype and genome assembly of muskrats.

**Supplementary Fig. S2**. Terms for expanded and contracted genes in muskrat and musk deer.

**Supplementary Fig. S3**. Sequence alignment highlighting sites that evolved in *DST, CKAP5*, and *NoP2*.

**Supplementary Fig. S4**. Gene expression from 13 tissues in muskrat.

**Supplementary Fig. S5**. Significantly enriched GO terms in tissue-specific expressed genes for each tissue. Tissue specificity of gene abundance was reflected by the tau score (τ).

**Supplementary Fig. S6**. Transcriptome map of muskrat analyzed.

**Supplementary Fig. S7**. Hi-C data quality and global chromatin interaction patterns during 2 representative musk gland stages.

**Supplementary Fig. S8**. Basic features of PEIs.

**Supplementary Fig. S9**. PSGs and REGs involved in cell cycle and sequence alignment, highlighting sites that evolved in parallel in *Casp8ap2, NAGS*, and *Cep250*.

**Supplementary Fig. S10**. Promoter–enhancer interactions (PEIs) rewired in the musk gland of secretion and nonsecretion stages.

**Supplementary Table S1**. Sequencing data summary.

**Supplementary Table S2**. Summary for the chromosome-scale genomes of muskrat and musk.

**Supplementary Table S3**. Overview of chromosome-scale genomes of muskrat and musk deer.

**Supplementary Table S4**. Summary of assembly quality evaluation.

**Supplementary Table S5**. Summary of the TEs and statistics of gene structure in muskrat and musk deer genomes.

**Supplementary Table S6**. PSGs, REGs, and convergent evolution gene in muskrat and musk deer genomes.

**Supplementary Table S7**. The significantly enriched terms for expanded genes in muskrat and musk deer.

**Supplementary Table S8**. The significantly enriched terms for contracted genes in muskrat and musk deer.

**Supplementary Table S9**. The significantly enriched terms for rapid evolution genes in muskrat.

**Supplementary Table S10**. The significantly enriched terms for positive selected and rapid evolution genes in musk deer.

**Supplementary Table S11**. Tissue-specific genes in each tissue.

**Supplementary Table S12**. Marker genes of each cell type.

**Supplementary Table S13**. Number of cells of each cell type in 2 musk secretion stages.

**Supplementary Table S14**. DEGs in comparison between musk secretion and nonsecretion stages detected in cluster 14.

**Supplementary Table S15**. Hi-C data summary.

**Supplementary Table S16**. Hi-C matrix resolution evaluation by *cis* contacts.

## Abbreviations

2-APB: 2-aminoethoxydiphenyl borate; BUSCO: Benchmarking Universal Single-Copy Orthologs; DEG: differentially expressed gene; FDR: false discovery rate; GEC: glandular epithelial cells; GO: Gene Ontology; GTR: general time-reversible; IS: insulation score; KEGG: Kyoto Encyclopedia of Genes and Genomes; lncRNA: long noncoding RNA; LRT: likelihood ratio test; miRNA: microRNA; MPC: mesenchymal progenitor cell; NAG: N-acetylglutamate; NCBI: National Center for Biotechnology Information; ONT: Oxford Nanopore Technologies; PASA: Program to Assemble Spliced Alignment; PEI: promoter–enhancer interaction; PSG: positively selected gene; QV: quality value; RAR: retinoic acid receptor; REG: rapidly evolving gene; RNA-seq: RNA sequencing (RNA-seq); scRNA: single-cell RNA; TAD: topologically associating domain; TE: transposable element; TPM: transcripts per million; TRP: transient receptor potential; t-SNE: t–distributed stochastic neighbor embedding; UMAP: uniform manifold approximation and projection; VGP: Vertebrate Genome Project.
