# Supplementary material for: Multiomics analysis provides insights into musk secretion in muskrat and musk deer
Source: Gigascience. 2025 Feb 26;14:giaf006. doi: 10.1093/gigascience/giaf006 (PMC11878540; doi:10.1093/gigascience/giaf006)
Supplement: giaf006_Supplemental_Files [file giaf006_supplemental_files.zip › Surpporting information.docx]

**Supplementary Information**

**Supplementary Information Table of Contents**

**Supplementary Figs. 1-9**

**Supplementary Fig. 1** Karyotype and genome assembly of muskrats.

**Supplementary Fig. 2** Terms for expanded and contracted genes in muskrat and musk deer

**Supplementary Fig. 3** Sequence alignment highlighting sites that evolved in *DST*、*CKAP5*、*NoP2*.

**Supplementary Fig. 4** Gene expression from 13 tissues in muskrat.

**Supplementary Fig.5** Significantly enriched GO terms in tissue-specific expressed genes for each tissue. Tissue speciﬁcity of gene abundance was reﬂected by the tau score (τ).

**Supplementary Fig. 6** Transcriptome map of muskrat analyzed.

**Supplementary Fig. 76** Hi-C data quality and global chromatin interaction patterns during two representative musk gland stages.

**Supplementary Fig. 8** Basic features of PEIs.

**Supplementary Fig. 9** PSGs (REGs) involved in cell cycle and sequence alignment highlighting sites that evolved in parallel in *TEX15*, *Casp8ap2*, *NAGS*, and *Cep250*.

**Supplementary Fig. 10** Promoter-enhancer interactions (PEIs) rewired in the musk gland of musk secretion and non-secretion stages.


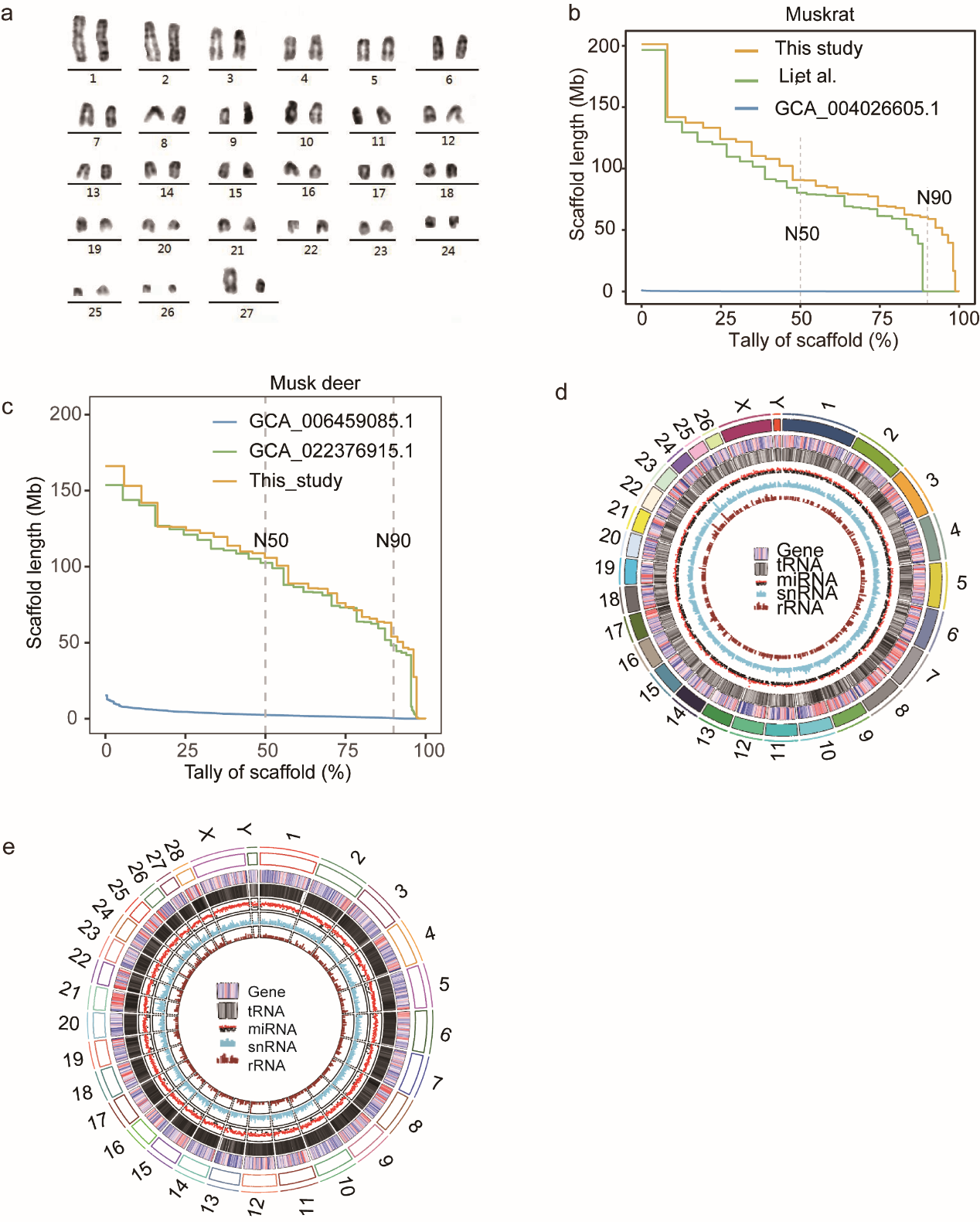


**Figure S1.** Karyotype and genome assembly of muskrats. (a) Muskrat karyotype. (b) Muskrat genome assembly in this study compared with two previous assemblies. (c) Musk deer genome assembly in this study compared with two previous assemblies. (d) Gene, tRNA, miRNA, snRNA, and rRNA distribution in the genome of muskrat. (e) Gene, tRNA, miRNA, snRNA, and rRNA distribution in the genome of muskrat.


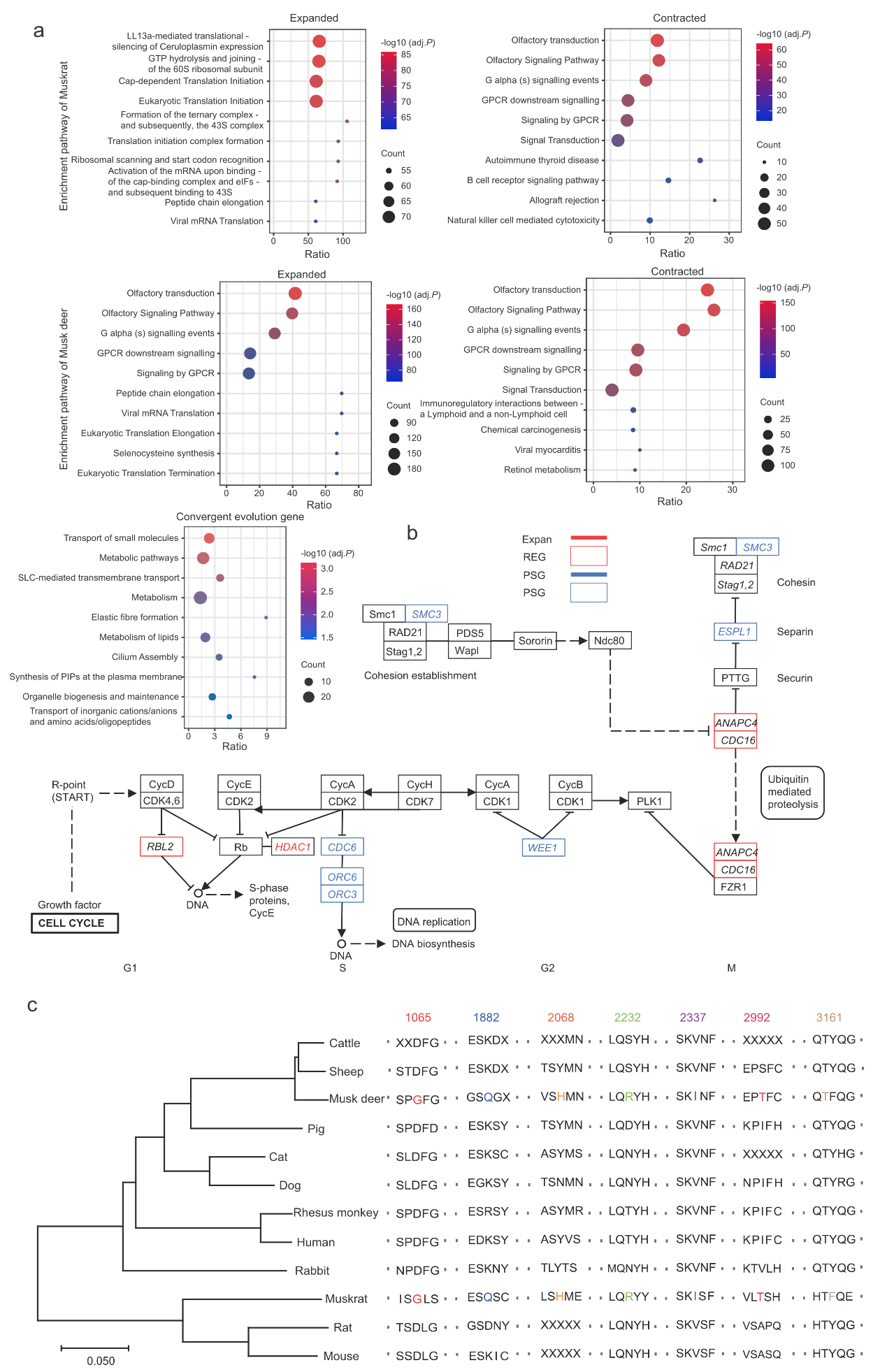
 **Figure S2.** (a) The signiﬁcantly enriched terms for expanded and contracted genes in muskrat and musk deer. PSGs (REGs) involved in cell cycle (b) and sequence alignment highlighting sites that evolved in parallel in *TEX15* (c).


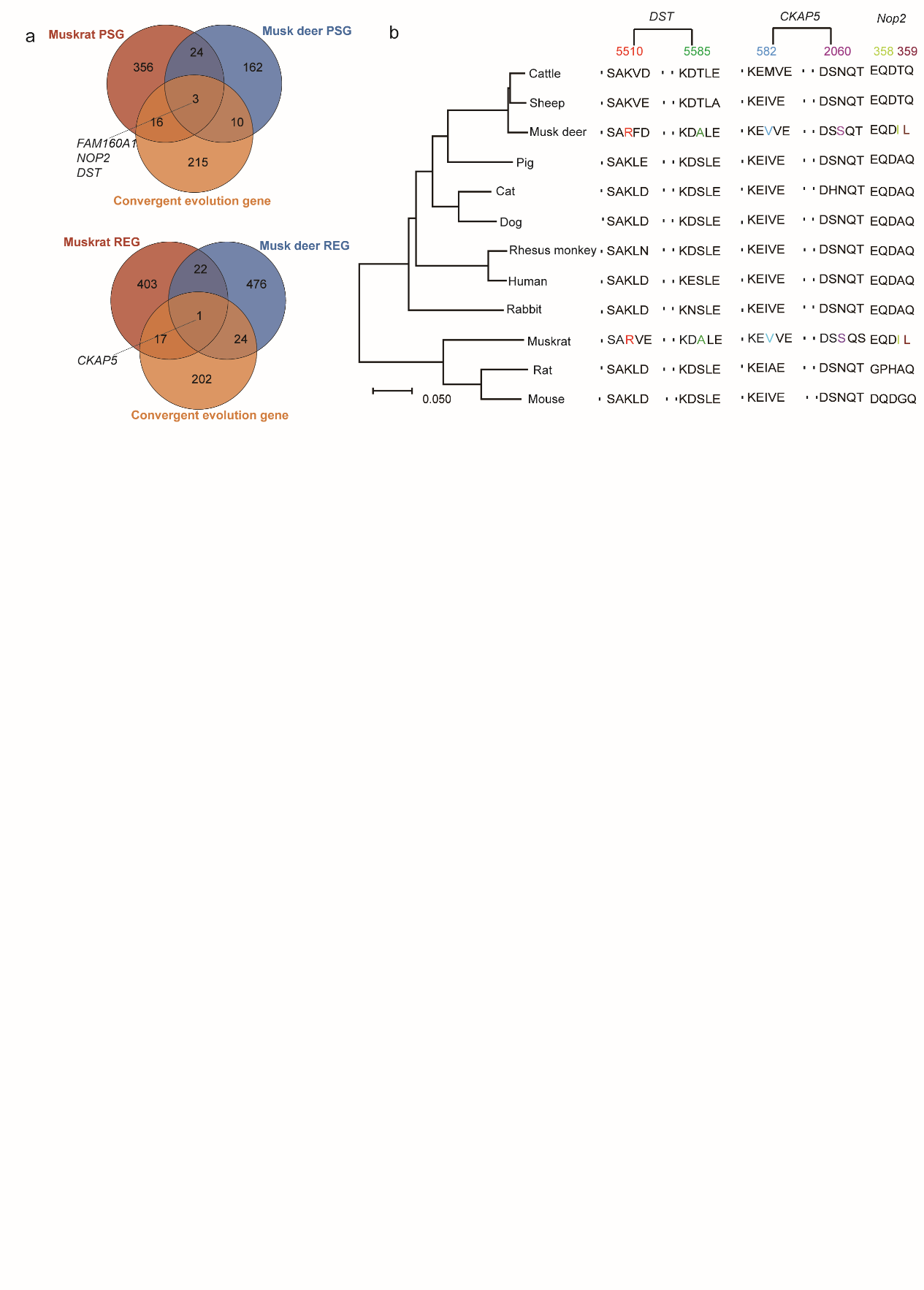


**Figure S3.** Overlapped genes between PSGs (REGs) and convergent evolution genes (a) and sequence alignment highlighting sites that evolved in these genes (b).


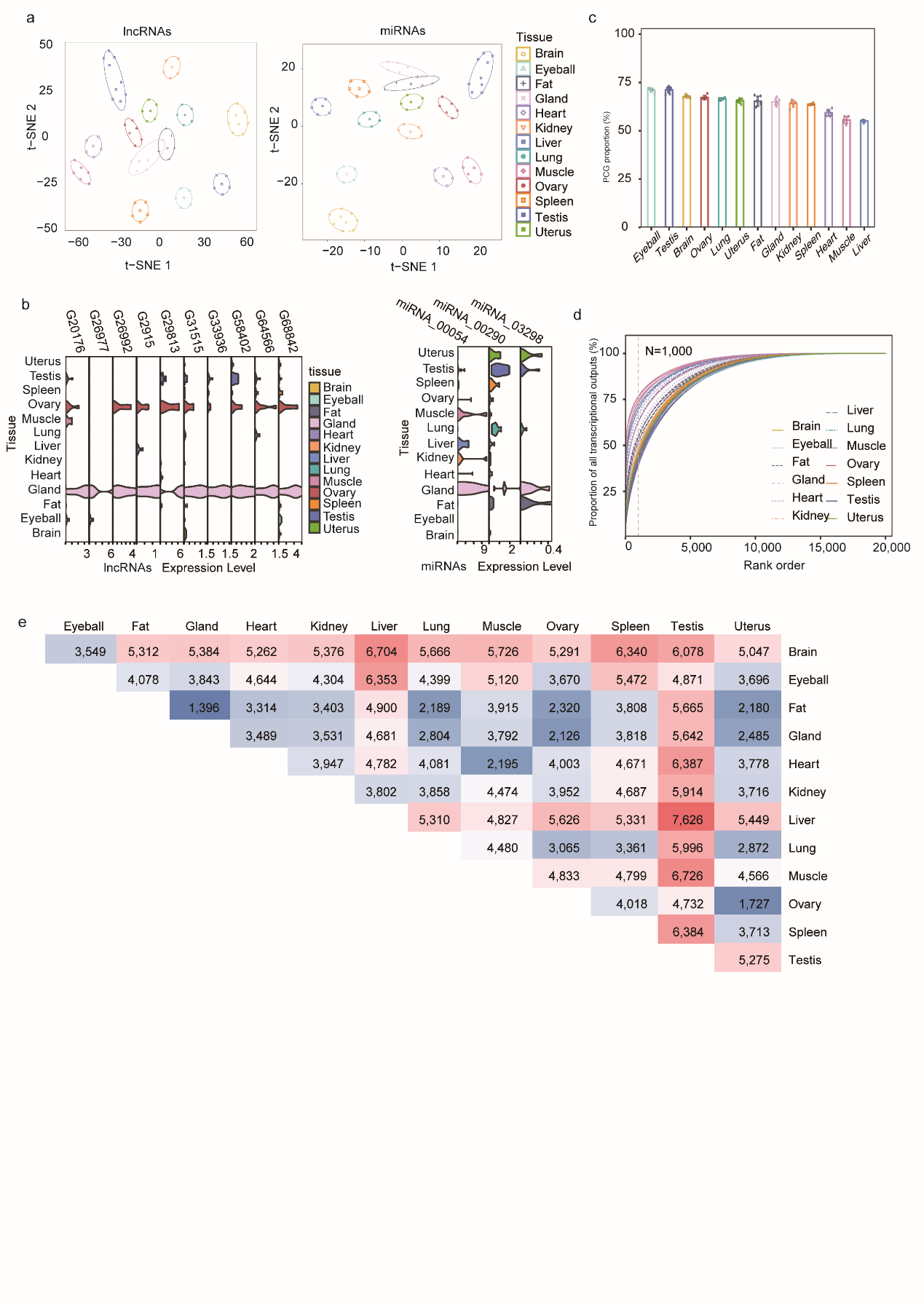


**Figure S4.** Gene expression from 13 tissues in muskrat. (a) t ‑ distributed stochastic neighbor embedding (t-SNE) clustering of samples using lncRNA (left panel) and miRNA (right panel) expression. (b) The lncRNAs (left panel) and miRNAs (right panel) specifically expressed in musk gland. (c) The proportion of expressed genes of each sample. Each dot represents a sample in every tissue. (d) Abundance distribution of transcripts across tissues. The x-axis indicates the proportion of transcripts sorted from highest to lowest abundance, with the vertical dashed line indicating the top 1,000 of highest abundance transcripts. The y-axis indicates the accumulated fraction of transcripts relative to the total transcripts. Colored lines represent mean values across tissues. (e) Heatmap of DEGs (differentially expressed genes) numbers in pairwise comparisons among the 13 tissues. DEGs were identified using the threshold of |log_2_fold change| > 1 and corrected (*P* < 0.01).


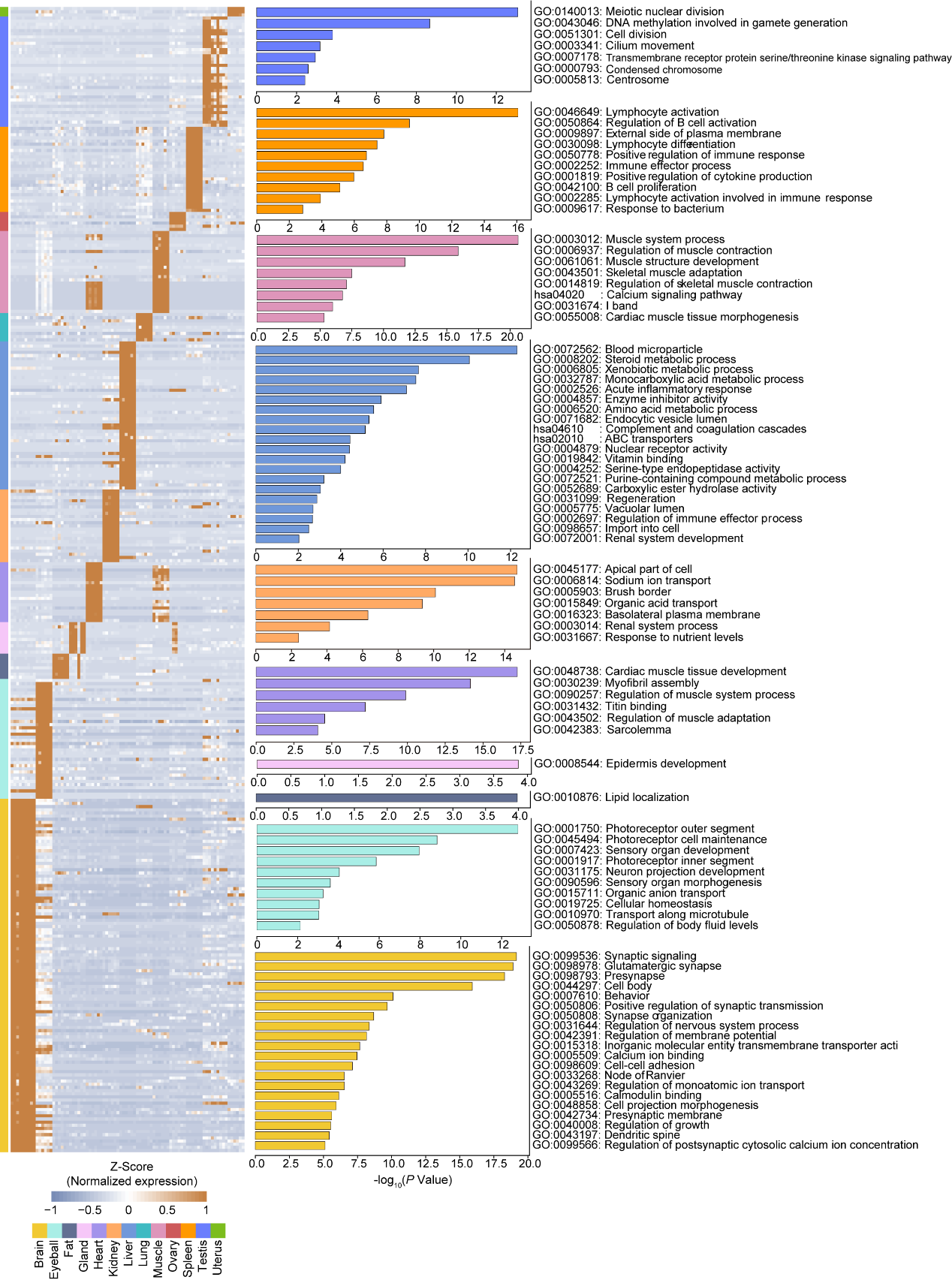


**Figure S5.** Significantly enriched GO terms in tissue-specific expressed genes for each tissue. Tissue speciﬁcity of gene abundance was reﬂected by the tau score (τ).


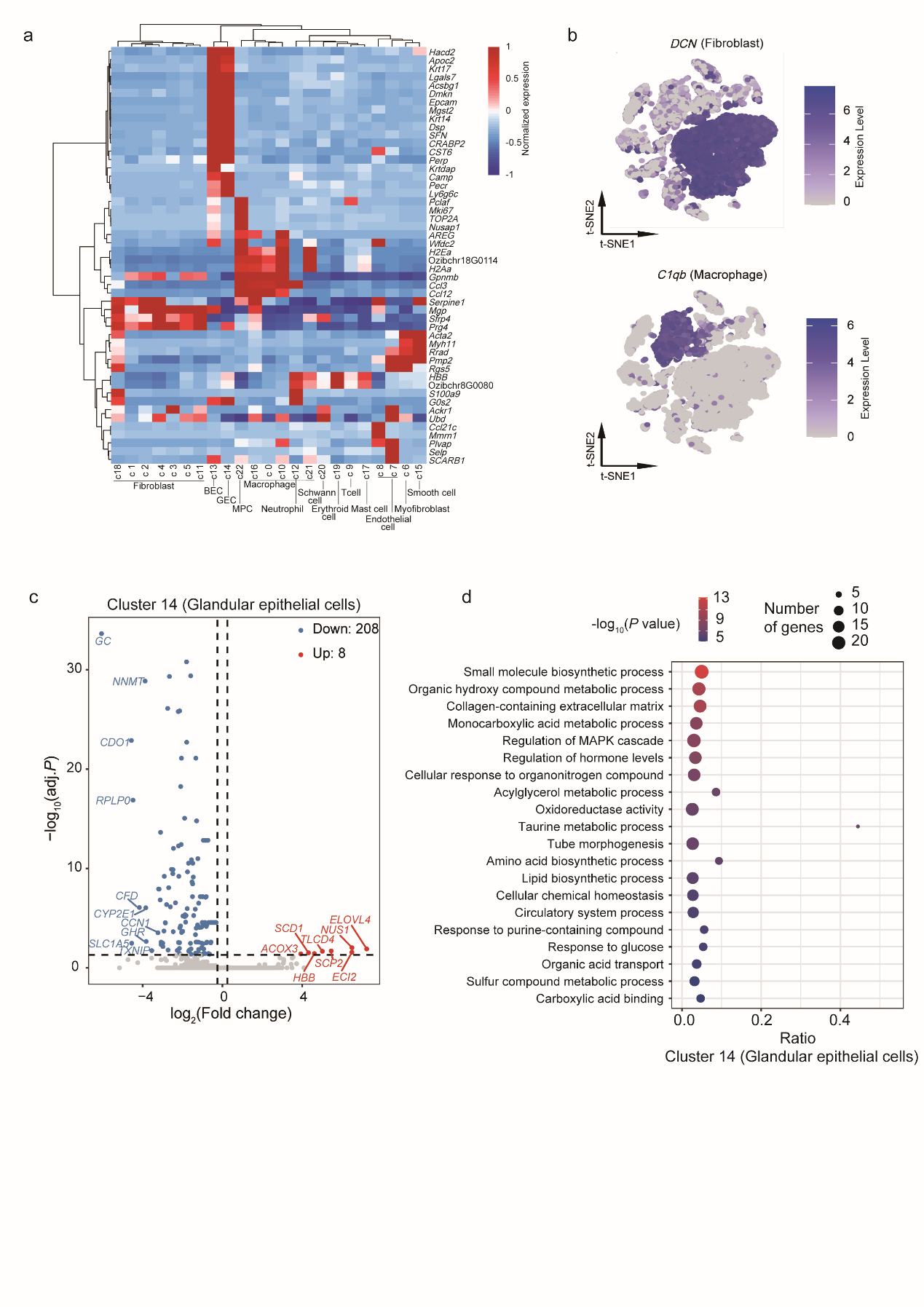


**Figure S6.** Transcriptome map of muskrat analyzed. (a) Heatmap and hierarchical clustering of cell clusters based on the expression of the top 50 most variable genes. (b) tSNE cluster map showing expression of genes characteristic of the major musk gland cell types. Red dashed lines give the boundaries of the main clusters of interest. Differential gene expression analysis in cells of Cluster 14. (c) Volcano plots showing significantly differentially expressed genes (DEGs) (|log_2_FC| >=0.25, corrected *P* value < 0.05) between musk gland of musk secretion and non-secretion stage. (d) Significantly enriched Gene Ontology terms of the DEGs.
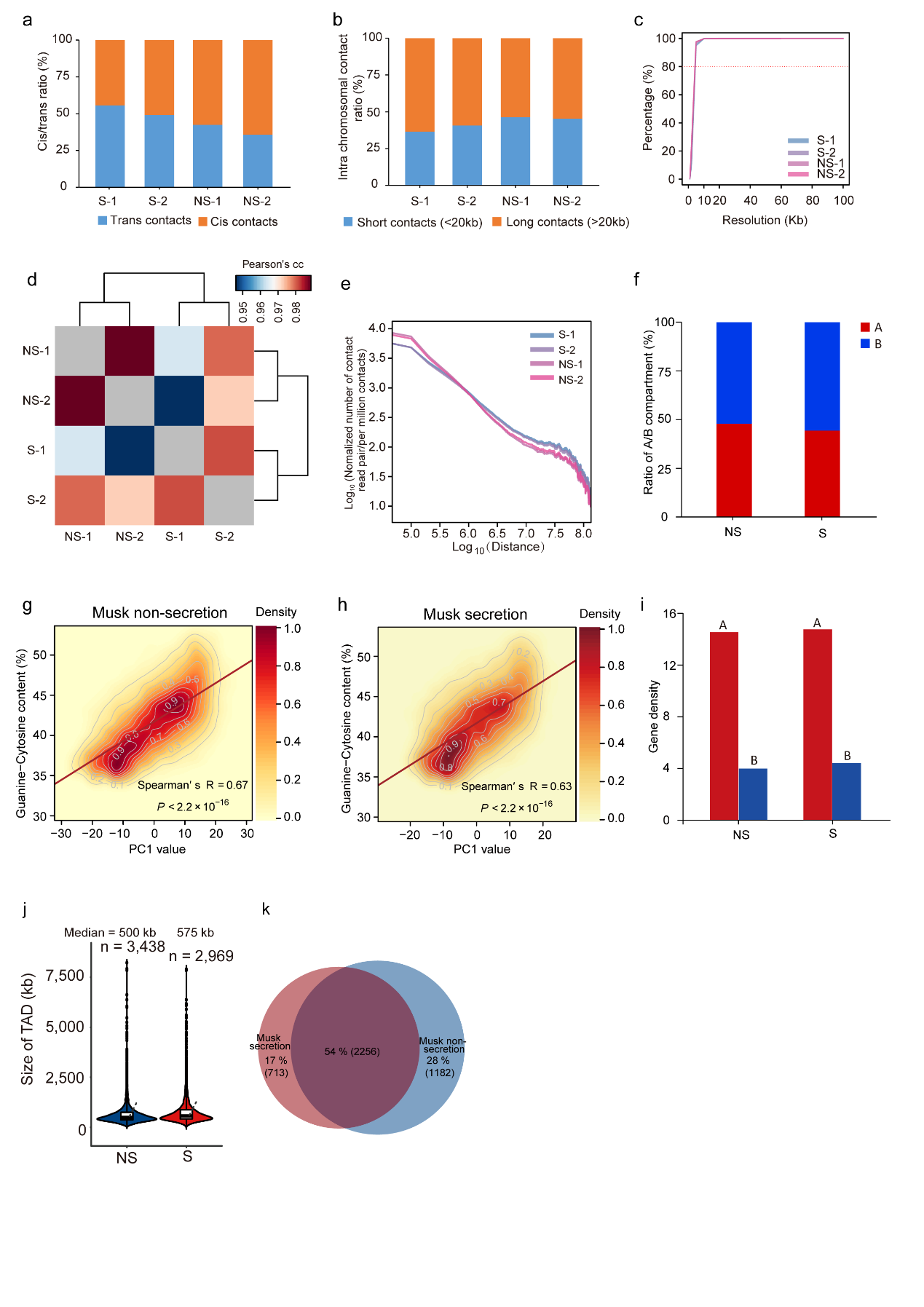


**Figure S7.** Hi-C data quality and global chromatin interaction patterns during two representative musk gland stages. (a) Percentage of *cis* and *trans* interactions in the Hi-C contacts. (b) Percentage of long-range contacts (> 20 kb) and short-range contacts (< 20 kb) of the *cis* interactions. (c) Resolution assessment of *cis* contact matrix. (d) Heatmap of correlation coefficient between Hi-C contact maps during two stages evaluated by HicRep. (e) Curves showing the dependence of normalized contact numbers for the genomic distance. (f) A/B compartment percentages of each stage. (g) GC content in A or B compartments during musk non-secretion stage. (h) GC content in A or B compartments during musk secretion stage. (i) Gene density of A or B compartments. (j) TAD size distribution during the two stages of the musk gland. (k) Overlap of TAD boundaries between the two stages of musk glands.


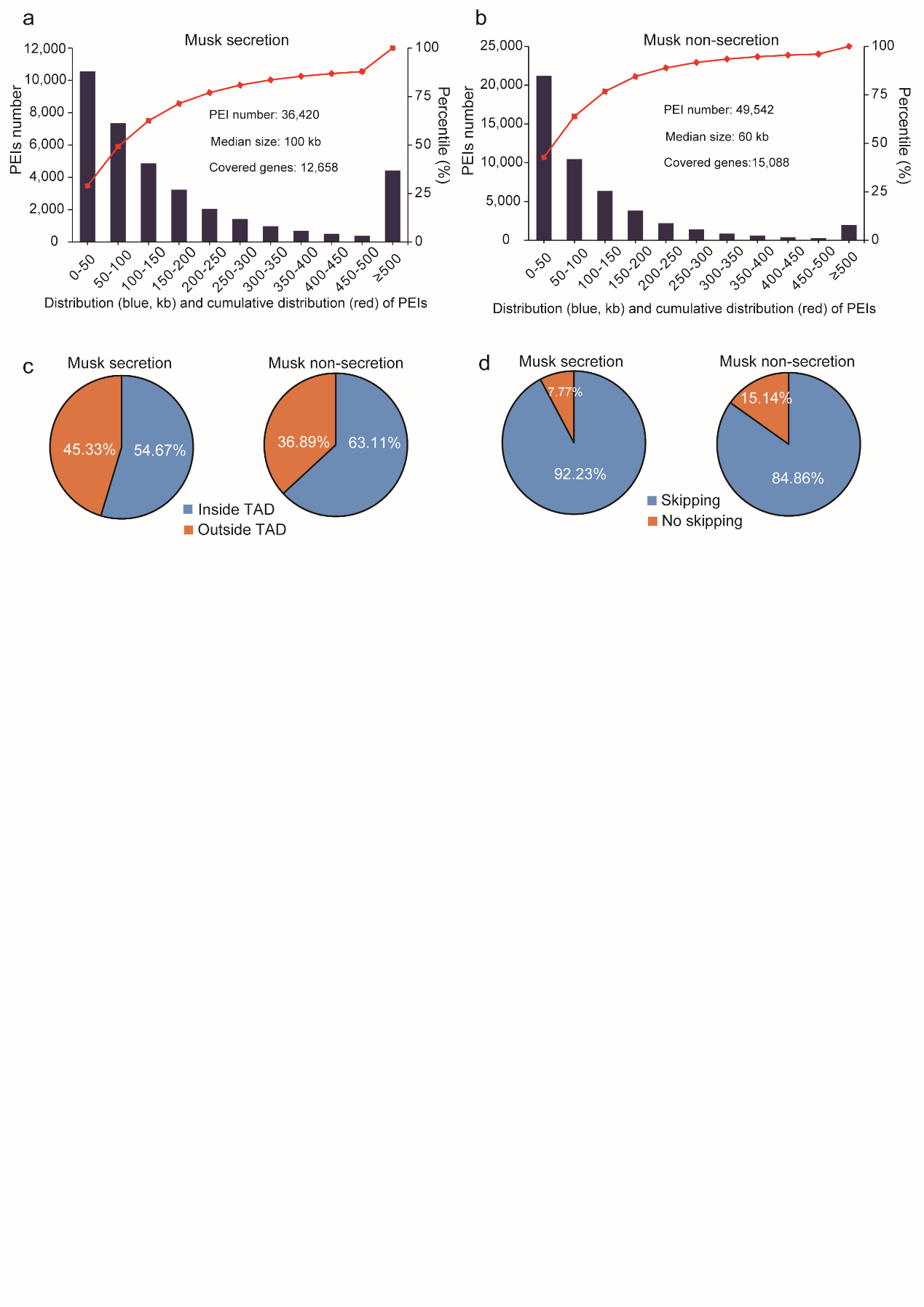


**Figure S8.** Basic features of PEIs. (a) Distribution of PEIs identified during musk secretion stage. (b) Number of PEIs identified during musk non-secretion stage. (c) Proportion of PEIs located within or across TADs. (d) Percentages of enhancers interacting with the nearest promoters. (e) Significantly enriched terms of genes with increased number (≥10) of enhancers in the musk secretion stage. (f) Significantly enriched genes with an increased number (≥10) of enhancers in musk non-secretion stage.


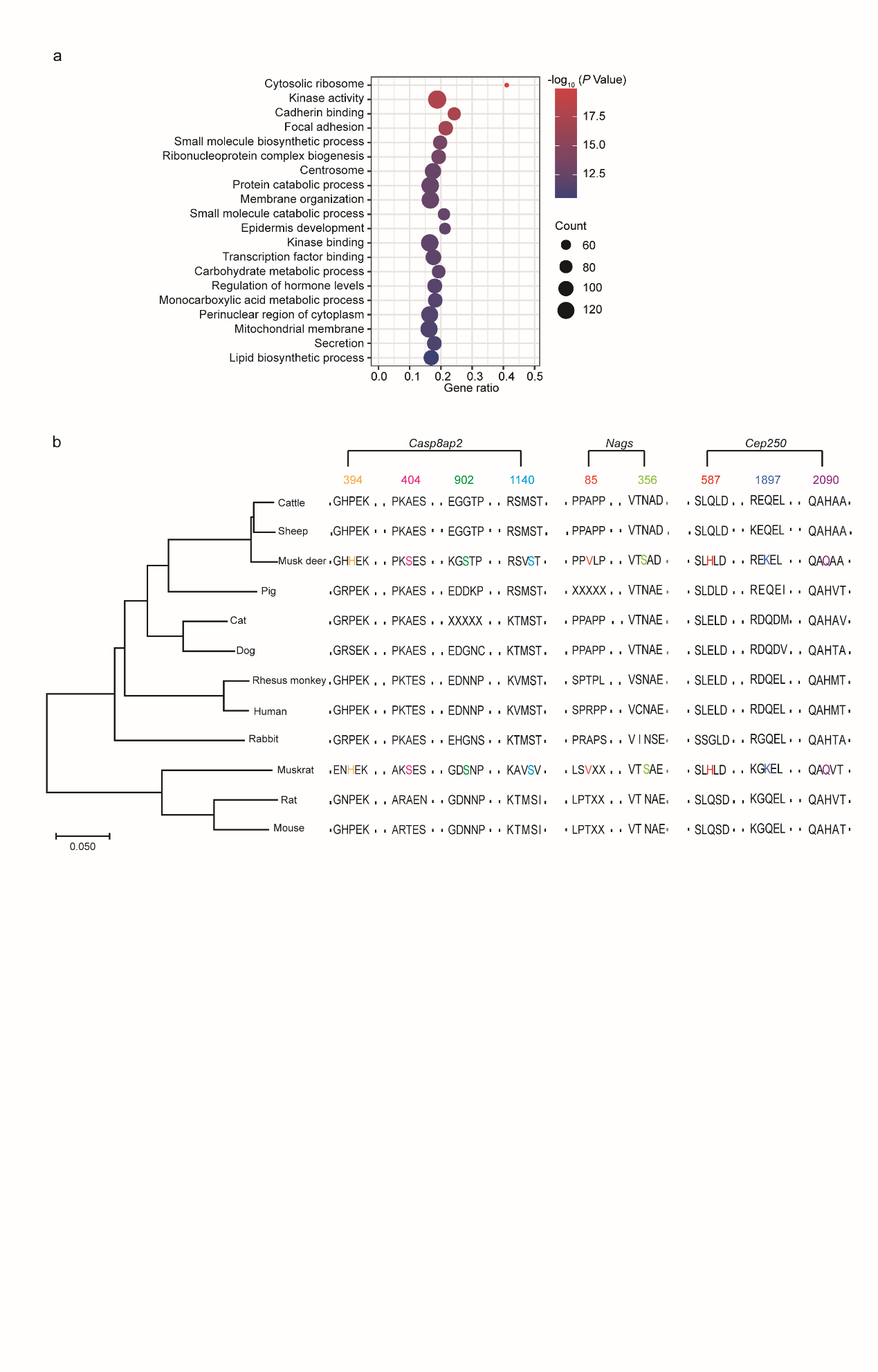


**Figure S9.** (a) Significantly enriched terms of genes in TADs with IS reduced in musk secretion stage. (b) Sequence comparison of parallel evolutionary sites in *Casp8ap2*, *NAGS*, and *Cep250*, *Casp8ap2* regulates cell proliferation and apoptosis; *NAGS* encodes a protein involved in the regulation of the urea cycle, and *CEP250* plays a crucial role in differentiating spermatogonia and meiotic spermatocyte.


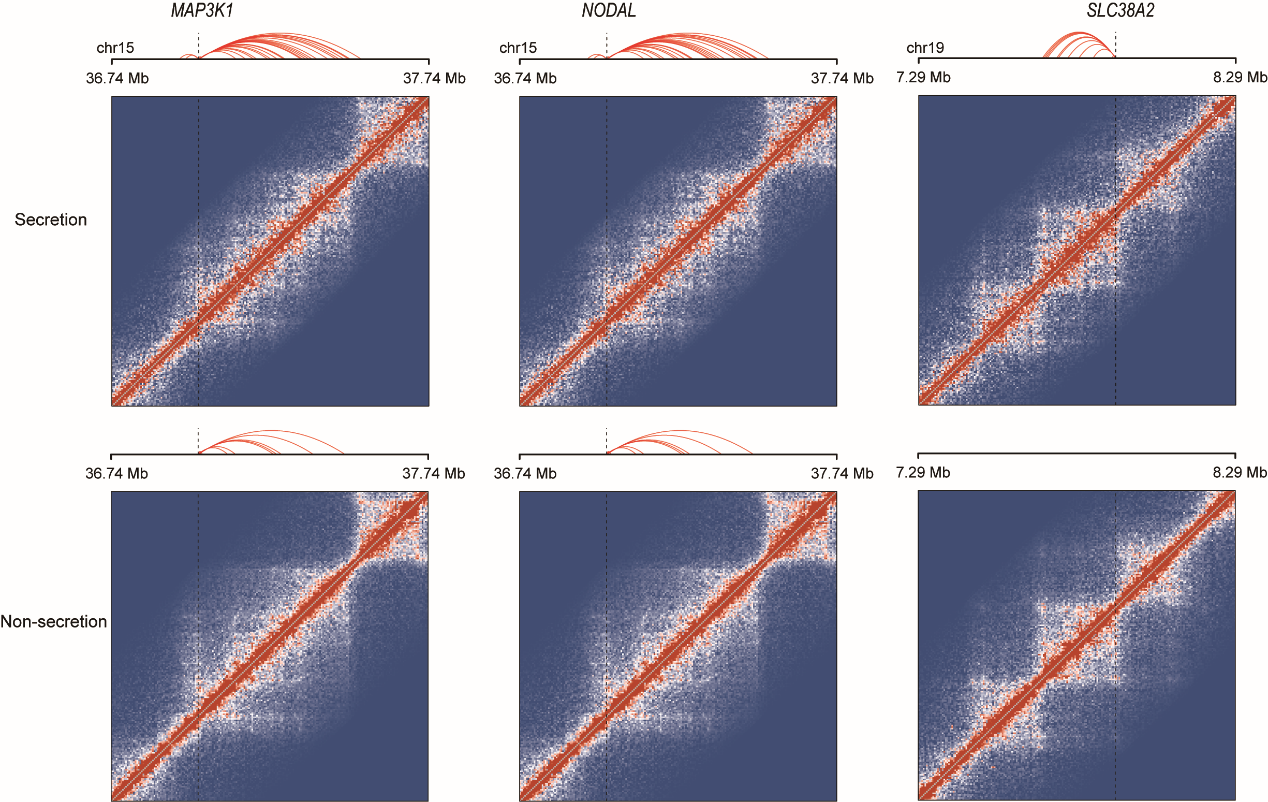


**Figure S10.** Promoter-enhancer interactions (PEIs) rewired in the musk gland of musk secretion and non-secretion stages. (a-c) PEI rewiring of a functional gene *MAP3K1*, *NODAL*, and *SLC38A2* between two stages. Top: schematics of PEIs and Hi-C contact heatmaps of the genomic region containing *MAP3K1*, *NODAL*, and *SLC38A2*. Bottom: gene structures in the region. The dashed line boxes indicate the chromosomal locations of the genes.
