# Supplementary material for: Multiomics analysis provides insights into musk secretion in muskrat and musk deer
Source: Gigascience. 2025 Feb 26;14:giaf006. doi: 10.1093/gigascience/giaf006 (PMC11878540; doi:10.1093/gigascience/giaf006)
Supplement: giaf006_GIGA-D-24-00205_Original_Submission [file giaf006_giga-d-24-00205_original_submission.pdf]

GigaScience

Multi-omics analyses identify distinct patterns of selection in musk secretion animals

--Manuscript Draft--

**Manuscript Number:**	GIGA-D-24-00205
**Full Title:**	Multi-omics analyses identify distinct patterns of selection in musk secretion animals
**Article Type:**	Research
**Funding Information:**	National Natural Science Foundation of China (81973428) — Dr Hang Jie<br>National Natural Science Foundation of China (82274046) — Dr Hang Jie<br>National Natural Science Foundation of China (32272859) — Mr. Zhengrong Yuan<br>Fundamental Research Funds for the Central Universities of Beijing University of Chemical Technology (2022JK017) — Dr Hang Jie<br>the Beijing Nova Program (Z211100002121022，20230484446) — Mr. Shilin Tian
**Abstract:**	Background<br>Musk is secreted by the musk gland of adult male musk-secreting mammals during the breeding season, which is of potential pharmaceutical and cosmetic value. However, efforts to understand the molecular mechanism of musk-secretion are scarce, hindered by the lack of comprehensive multi-omics analyses and respective platform for the related species (including muskrats: Ondatra zibethicus Linnaeus and Chinese forest musk deer: Moschus berezovskii Flerov).<br>Results<br>Hence, we generated chromosome-level genomes of the two species (Ondatra zibethicus Linnaeus and Moschus berezovskii Flerov) and 168 muskrat transcriptomes. Comparative analyses with eleven other vertebrate genomes, we detected genes and amino acid sites with putative adaptive convergent evolution in the two musk-secreting species, primarily related to lipid metabolism, cell cycle, protein binding and immunity functions. Single cell RNA sequencing and Hi-C analysis indicated that enhanced expression during the musk secretion stage of muskrat is related to the biological process “regulation of secretion”. We further developed a freely accessible, user-friendly multi-omics database platform (MuskDB, http://117.78.45.2:1087/home) for musk-secreting mammals.<br>Conclusions<br>Our findings, alongside the provided database, facilitate a deeper understanding of the molecular mechanism underlying the unique phenomenon of musk secretion, and may provide insights for the mating behavior and breeding of muskrat and musk deer.
**Corresponding Author:**	Diyan Li<br>Chengdu University<br>CHINA
**Corresponding Author Secondary Information:**	
**Corresponding Author's Institution:**	Chengdu University
**Corresponding Author's Secondary Institution:**	
**First Author:**	Tao Wang
**First Author Secondary Information:**	
**Order of Authors:**	Tao Wang
	Maosen Yang

	Xin Shi
	Shilin Tian
	Yan Li
	Wenqian Xie
	Zhengting Zou
	Dong Leng
	Ming Zhang
	Chengli Zheng
	Chungang Feng
	Bo Zeng
	Xiaolan Fan
	Huimin Qiu
	Jing Li
	Guijun Zhao
	Diyan Li
	Zhengrong Yuan
	Hang Jie
Order of Authors Secondary Information:	
Additional Information:	
Question	Response
Are you submitting this manuscript to a special series or article collection?	No
**Experimental design and statistics**<br><br>Full details of the experimental design and statistical methods used should be given in the Methods section, as detailed in our Minimum Standards Reporting Checklist. Information essential to interpreting the data presented should be made available in the figure legends.<br><br>Have you included all the information requested in your manuscript?	Yes
**Resources**<br><br>A description of all resources used, including antibodies, cell lines, animals and software tools, with enough information to allow them to be uniquely identified, should be included in the	Yes

Methods section. Authors are strongly encouraged to cite Research Resource Identifiers (RRIDs) for antibodies, model organisms and tools, where possible.<br><br>Have you included the information requested as detailed in our Minimum Standards Reporting Checklist?	
**Availability of data and materials**<br><br>All datasets and code on which the conclusions of the paper rely must be either included in your submission or deposited in publicly available repositories (where available and ethically appropriate), referencing such data using a unique identifier in the references and in the “Availability of Data and Materials” section of your manuscript.<br><br>Have you have met the above requirement as detailed in our Minimum Standards Reporting Checklist?	Yes

Powered by Editorial Manager® and ProduXion Manager® from Aries Systems Corporation

Manuscript Click here to access/download;Manuscript;Manuscript.docx

Multi-omics analyses identify distinct patterns of selection in musk secretion animals

Tao Wang[1], Maosen Yang[2,3], Xin Shi[4,5], Shilin Tian[6], Yan Li[7], Wenqian Xie[3], Zhengting Zou[8], Dong Leng[4], Ming Zhang[4], Chengli Zheng[5], Chungang Feng[9], Bo Zeng[4], Xiaolan Fan[4], Huimin Qiu[10], Jing Li[9], Guijun Zhao[2], Diyan Li[1*], Zhengrong Yuan[3*]and Hang Jie[1,2*]

[1] Antibiotics Research and Re-evaluation Key Laboratory of Sichuan Province, Sichuan Industrial Institute of Antibiotics, School of Pharmacy, Chengdu University, Chengdu 610106, China.
[2] Jinfo Mountain Forestry Ecosystem of Chongqing Observation and Research Station, Chongqing Institute of medicinal plant cultivation, Chongqing college of traditional Chinese medicine, Chongqing 402760, China
[3] College of Biological Science and Technology, Beijing Forestry University, Beijing 100083, China
[4] College of Animal Science and Technology, Sichuan Agricultural University, Chengdu 611130, China
[5] Sichuan Institute of Musk Deer Breeding, Chengdu 611845, China
[6] College of Life Sciences, Wuhan University, Wuhan 430072, China
[7] Chengdu Research Base of Giant Panda Breeding, Chengdu 611081, China
[8] Key Laboratory of Zoological Systematics and Evolution, Institute of Zoology, Chinese Academy of Sciences, Beijing 100101, China
[9] College of Animal Science and Technology, Nanjing Agricultural University, Nanjing 210095 China
[10] College of Agriculture, Kunming University, Kunming 650214, China

Tao Wang, Maosen Yang, Xin Shi, Shilin Tian, Yan Li and Wenqian Xie contribute equally to this work.
[*] For correspondence: Diyan Li, Zhengrong Yuan and Hang Jie.

Abstract

Background

Musk is secreted by the musk gland of adult male musk-secreting mammals during the breeding season, which is of potential pharmaceutical and cosmetic value. However, efforts to understand the molecular mechanism of musk-secretion are scarce, hindered by the lack of comprehensive multi-omics analyses and respective platform for the related species (including muskrats: *Ondatra zibethicus* Linnaeus and Chinese forest musk deer: *Moschus berezovskii* Flerov).

Results

Hence, we generated chromosome-level genomes of the two species (*Ondatra zibethicus* Linnaeus and *Moschus berezovskii* Flerov) and 168 muskrat transcriptomes. Comparative analyses with eleven other vertebrate genomes, we detected genes and amino acid sites with putative adaptive convergent evolution in the two musk-secreting species, primarily related to lipid metabolism, cell cycle, protein binding and immunity functions. Single cell RNA sequencing and Hi-C analysis indicated that enhanced expression during the musk secretion stage of muskrat is related to the biological

process "regulation of secretion". We further developed a freely accessible, user-friendly multi-omics database platform (MuskDB, http://117.78.45.2:1087/home) for musk-secreting mammals.

Conclusions

Our findings, alongside the provided database, facilitate a deeper understanding of the molecular mechanism underlying the unique phenomenon of musk secretion, and may provide insights for the mating behavior and breeding of muskrat and musk deer.

Introduction

Natural musk is mainly secreted by musk gland located between the navel and genitals of mature male forest musk deer (*Moschus berezovskii* Flerov), an endangered artiodactyl species native to southern and central China and northernmost Vietnam [1]. In addition, the muskrat (*Ondatra zibethicus* Linnaeus), a semiaquatic rodent native to North America [2] and Canada but has been introduced to Europe, Asia, South America, and Australia, has similar musk gland and secret musk likewise. The musk secreted by forest musk deer and muskrat produce a specific fragrance, whose chemical composition may be involved in chemical communication, potentially encoding information about sexual maturity and attraction [3]. The chemical composition analysis of musk showed that it contained active macrocyclic ketone components such as muscone and normuscone [4]. Muskrat musk also contains macrocyclic ketone compounds such as muscone and normuscone [5]. This class of substances is thought to be important for exerting drug effects. In our previous research, we found that musk of muskrat and musk deer have up to 272 identical metabolites, including organic compounds such as amino acids, fatty acids, ketones, aldehydes, and steroids [6]. Compared with other musk-secreting mammals (i.e. other musk deer species), there has been more captive breeding practice for the forest musk deer and the muskrat. The high-quality genome sequences of these two species and comparative analyses with the other mammalian genomes can potentially shed light on their genome diversity and the genetic components underlying musk secretion, which may have experienced convergent adaptation during the long process of evolution.

Here, we sequenced the genomes of a male muskrat and a male musk deer. In addition, to characterize the transcriptomic variability with respect to known tissue-specific physiological activities and identify key genes underlying the musk-secreting phenotype, we sequenced 84 RNA-seq libraries and 84 small RNA-seq libraries of various muskrat organs. To accurately depict cell composition and transcriptomic changes in the musk gland of muskrat between musk secretion and non-secretion stages, we further used a single-cell RNA (scRNA) approach to dissect the transcriptional differences on a 10× Genomics system. We also examined the potential chromatin architecture dynamics underlying the phenotype by sequencing three and four Hi-C libraries for musk gland respectively in secretion and non-secretion stage (**Table S1**). Integrated these multi-omics data, we characterized the underlying molecular mechanisms regarding the adaptive evolution of musk secretion in muskrat and musk

deer, relating to the biological process and function of lipid metabolism, regulation of secretion, cell cycle, protein binding and immunity functions, etc.

Results and discussion

Genome assembly of two musk-secreting mammals

We sequenced the genomes of two male (only male secret musk) musk-secreting mammals [*Ondatra zibethicus* (muskrat) (2 years of age) and *Moschus berezovskii Flerov* (forest musk deer) (2.5 years of age)] via integration of Oxford Nanopore Technologies (ONT) long reads, high-throughput chromosome conformation capture (Hi-C) data and BGI T7 paired-end sequences (**Table S1**). We assembled the two chromosome-level genomes by applying an improved assembly method that utilizes Hi-C interaction pairs to cluster ONT long sequences that possess potential linkages and avoid any erroneous overlap caused by long-distance repetitive sequences during string graph assembly[7] (**see methods**). We successfully generated 2.48 Gb and 2.83 Gb for muskrat and musk deer genomes with contig N50 values of 60.53 and 69.45Mb, which anchored onto 28 and 30 chromosomes, respectively (**Table 1, Table S2; Fig. 1a**). The 28 anchored chromosomes in muskrat were confirmed by karyotype analysis (**Fig. S1a**). In particular, five chromosome sequences have reached the gap-free level in the muskrat genome (**Table S3**); and our two assemblies have improved N50 length of the contig for the muskrat and musk deer by 1,048- and 3.56-fold compared to the published sequences[8, 9], respectively (**Fig. S1b**). Our assembled genomes exhibit excellent completeness, as evidenced by the coverage of > 99% paired-end reads across > 99% of the genome, and recovery of averaged 96.95% of BUSCOs (Benchmarking Universal Single-Copy Orthologs) [10] in 9,226 conserved mammalian genes from the mammalia_odb10 database (**Table S4**). Furthermore, we used a reference-free and *k*-mer based approach and estimated a high assembly quality value (QV) of more than 44, exceeding the Vertebrate Genome Project (VGP) standard of QV40[11, 12]. Subsequently, we predicted 1013.31 Mb (40.85%) and 1539.41 Mb (54.33%) transposable elements (TEs) for muskrat and musk deer, respectively (**Table S5**). By combining homology- and *ab initio*-based methods, aided by evidence of transcription, we identified 23,260 and 24,375 protein-coding genes in the muskrat and musk deer genomes, respectively (**Table S6, Fig. S1c**).

Table 1. Global summary of two assemblies for muskrat and musk deer

Genomic features	Muskrat	Musk deer
Assembled genome size (Gb)	2.48	2.83
Percentage of anchoring (%)	97.19	98.79
Contig Number	561	1,173
Contig N50 (Mb)	60.53	69.45
GC content (%)	41.69	42.14
Repeat ratio (%)	40.85	54.33
Predicted number of protein coding genes	23,260	24,375

QV	44.48	44.09
BUSCOs (%)	96.89	96.28

We next explored gene family expansion and contraction in two musk secretion species using the software CAFE [13]. We first reconstructed a maximum likelihood tree of 12 focal mammals using 7,409 one-to-one orthologs. Next, we used OrthoFinder to assign genes from 12 focal mammals into 19,382 gene families and assessed gene family changes with the ancestral branch leading to muskrat or musk deer defined as the foreground. As a result, we found that 181 gene families underwent an expansion and 134 underwent a contraction for the musk deer. Muskrat shows comparable numbers of gene family contraction (166) and expansion (161) events (**Fig. 1b**).

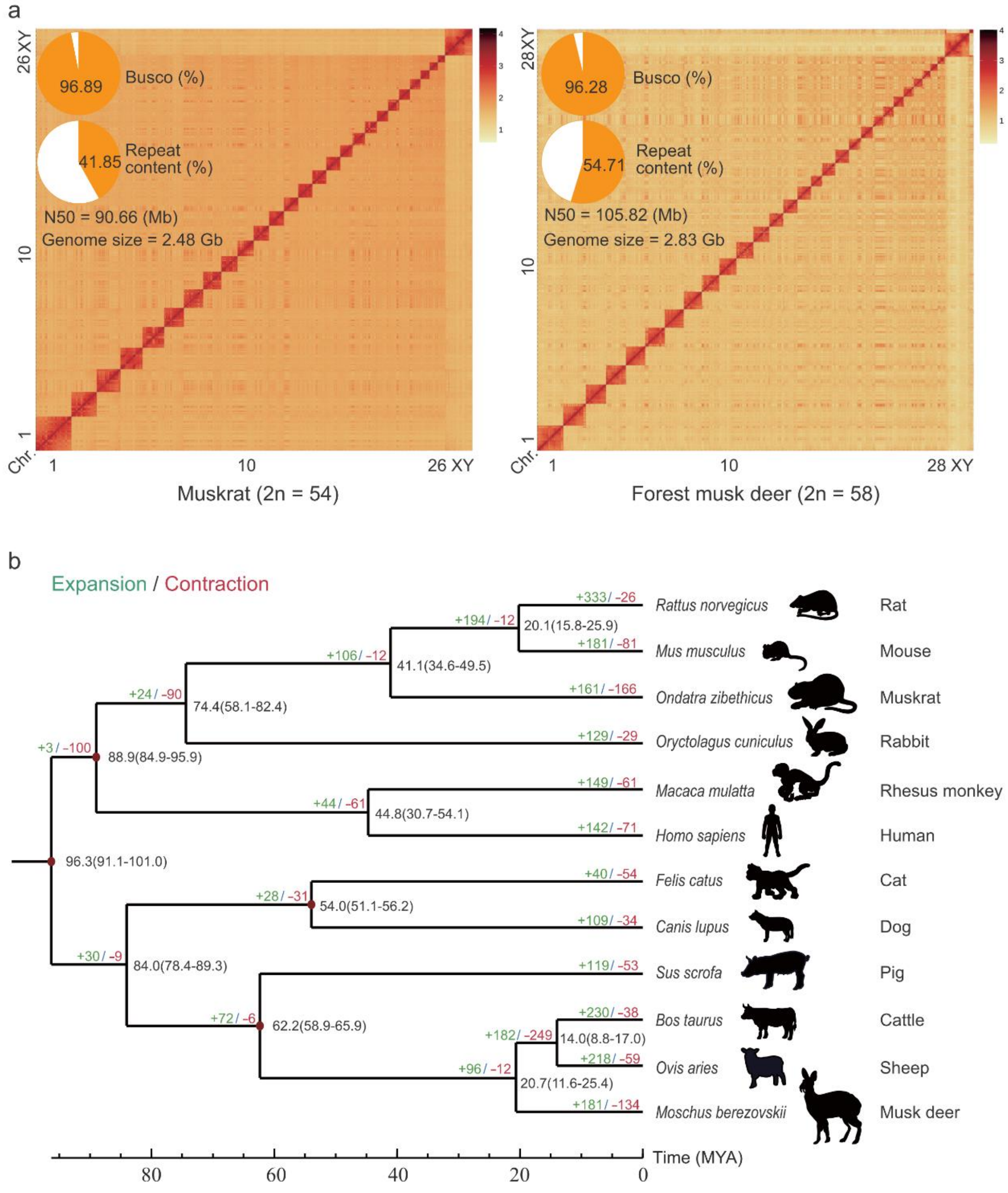

Figure 1 Genomes and maximum likelihood phylogeny based on 7,409 one-to-one orthologues. (a) Hi-C heatmaps for muskrat and forest musk deer generated by Juicebox Assembly Tools [14]. Pie charts represent the proportion of conserved BUSCO gene sets and repeat content. Contig N50 and assembled genome size are shown. (b) Divergence times and expansion and contraction of gene families in muskrat and musk deer genomes. Numbers on the nodes represent divergence times, with the error range shown in parentheses. The numbers of gene families that expanded (green) or contracted (red) in each lineage after speciation are shown on the corresponding branch. The phylogenetic tree of the 12 mammalian species was constructed with a maximum likelihood method using 7,409 one-to-one orthologs.

Tissue-specific expression of genes in musk gland

To explore the genes specifically expressed in musk gland and their functions, we used muskrat as a model animal to conduct further analyses, as muskrat tissue samples are accessible in contrast to the endangered musk deer. We first constructed 84 RNA-seq libraries and 84 small RNA-seq libraries to explore tissue specific expression patterns in the transcriptome of muskrat among 13 tissues (two from entoderm [liver and lung], eight from mesoderm [testis, heart, spleen, kidney, muscle, fat, uterus, and ovary], and three from ectoderm [brain, eyeball, and musk gland]) (**Fig. 2a**), with at least six biological replicates for each stage. We then updated the annotation of distinct transcript types including lncRNAs (**Fig. S2a**) and miRNAs (**Fig. S2b**), representing a core atlas dataset of *de novo* assembled transcripts. After filtering the low expression levels genes with transcripts per million (TPM) < 1 in at least 50% samples in each analyzed tissue, we evaluate the expression levels of 14,861 (63.89%) muskrat genes and particularly looked for tissue specific expression patterns.

The results showed that the transcriptional profiles of each tissue type are highly reproducible among biological replicates (Spearman's $r > 0.80$) (**Fig. 2b**). Ovary, musk gland and uterus also clustered into obviously separate respective groups. Meanwhile, brain and eyeball tissues clustered together (**Fig. 2c**). More than 60% of genes were expressed in each tissue for muskrat (**Fig. S2c**), but expression levels of different genes are skewed. In most tissues, the expression of highly expressed 1,000 genes takes up an average expression of more than 50%, especially for muscle (**Fig. 2d**). We also observed dissimilarities between the gene expression level distribution across tissues. The most abundant transcripts (the top 1,000, as ranked by expression levels) in a tissue, accounted for greater than half of the total transcribed muscle (~71.71%), liver (~68.76%), and heart (~61.34%), whereas testis (~37.70%) had a more uniform distribution (**Fig. 2e, Fig. S2d**). Testis showed the highest number of differentially expressed genes compared with other tissues (**Fig. S2e**).

In terms of expression, the tissue-specific genes were commonly enriched in distinct cellular functions. For example, the specifically expressed genes for the musk

gland were mainly involved in "epidermis development" and those for testis in "male gamete generation" and "meiotic nuclear division" (**Fig. S3**). Muskrat PSGs (*HBEGF*, *PIGR*, *PLCE1*, *NCK2*) was enriched in the "Epidermal growth factor receptor signaling pathway" and REG (*TGFBR3*, *BMP4*, *STRAP*, *ATF2*, *CDC73*) were enriched in "Negative regulation of epithelial cell proliferation". These results indicated the genes responsible for epidermis development have high and specific expression in musk gland and experienced putatively adaptive evolutionary expansion.

In addition, we also found that some musk gland specific genes are related to lipid metabolism, such as *TRPV3* and *LIPM* (**Fig. 2f**). Transient receptor potential (TRP) channels are polymodal sensors that convert a multitude of environmental cues into cellular signaling events essential for physiology [15]. *TRPV3* is activated by warm temperatures as well as numerous chemicals, including plant extracts, lipid metabolites, and synthetic small molecules such as 2-aminoethoxydiphenyl borate (2-APB) [16, 17]. In mouse, *LIPM* (lipase) also have a restricted tissue expression in the epidermal tissue [18], supporting the unique and active function of *LIPM* in musk gland function.

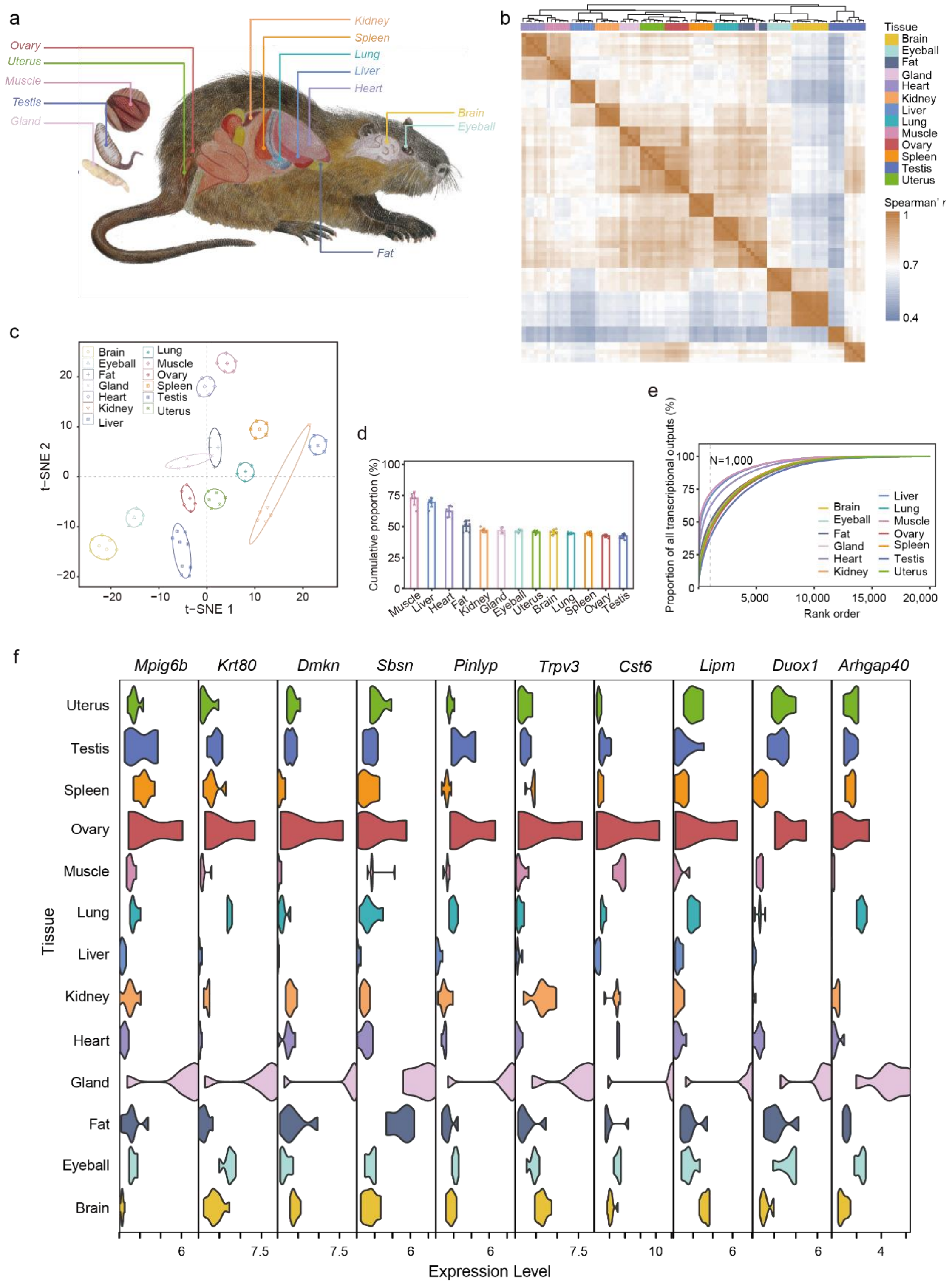

Figure 2: Characteristics of the muskrat BodyMap transcriptome. (a) Samples derived from 13 tissues and were used for muskrat transcriptome reconstruction. Hierarchical clustering (b) and t - distributed stochastic neighbor embedding (t-SNE) clustering (c) of samples using expression values (TPM). For the t-SNE plot, the ellipses indicate the samples of the same tissue with similar transcriptional profiles, constructed at a probability of 0.95. (n = 19,800). (d)The cumulative expression proportion of top 1,000 highly expressed genes in all samples. (e)Abundance distribution of transcripts across 13 tissues. The x-axis indicates the proportion of transcripts sorted from highest to lowest expression, with the vertical dashed line indicating the top 1,000 of highest abundance transcripts. The y-axis indicates the accumulated fraction of transcripts relative to the total

transcripts. Colored lines represent mean values across different tissues. (f)The genes specifically expressed in musk gland.

Single-cell reconstruction of musk secretion remodeling in the muskrat adult musk gland

To depict transcriptomic changes during musk secretion at the single-cell level We further measured the transcriptional differences between representative secretion and non-secretion stages in musk gland by 10× Genomics scRNA-seq system (**Fig. 3a**). After quality filtering, the transcriptome profiles of 19,398 cells were available for cell-type characterization (12,128, and 7,270 cells for musk secretion and non-secretion stages, respectively) (**Table S1**). To explore the cell types of these musk glands, we performed the uniform manifold approximation and projection (UMAP) analysis and identified 23 cell clusters (**Fig. 3b, c**). We surveyed the expression patterns of the top 50 most variable genes (**Fig. 3d**), which could cluster these cells into 13 known cell types (**Table S7**).

We found the vast majority of collected cells (49.53% and 59.16%) possess characteristics typical of fibroblasts (clusters 1, 2, 3, 4, 5, 11 and 18) with higher expression of *IGFBP3* [19, 20], *DCN* [21] and *C3* [22] (**Fig. 3e**). Clusters 0, 10, 16 and 21 were identified as macrophages with higher expression of *C1qa*, *C1qb*, *C1qc*, *Ctss*, *Cd14* and *Cd68* [23]; cluster 21with higher expression of *Coro1a* [23] and *Cd74* [24, 25]. Cluster17 was identified as mastocytes with expressed gene markers of *Alox5*, *Cpa3*, *Kit* and *Srgn* [26]. Cluster 22 expressed *Ccnb2*, *Hmgb2*, *Hmgb3*, *Mcm6*, *Ube2c* and *Uhrf1* [27] mesenchymal progenitor cell (MPCS) markers. In addition, a recent study [28] found that proliferative marker genes *Top2a*, *Mki67* and *Birc5* were markers for cluster 22 in our study. Neutrophile granulocyte (cluster 12) have a high expression of *Adam8*, *Arg2*, *Anxa1* and *C5ar1* [24]. Endothelial cells (clusters 7 and 8) expressed markers of *Plvap*, *Cav1*, *Cav2*, *Emcn*, *Gpihbp1*, *Pecam1* and *Tm4sf1* [23]. Myoepithelial cell (cluster 6) expressed gene markers of *Acta2*, *Myh11*, *Myl9*, *Mylk*, *Tpm* and *De* [29, 30]. T cell (cluster 9) expressed *Rapgef6*, *Ltb*, *Rpl12*, *Rplp1*, *Rps16* and *Rps23* [24] and *CD3* gene markers [31-34]. Basal epithelial cells (cluster 13) expressed *Ccnd2*, *Krt14* and *Krt17* markers [35]. Acinar cells/glandular epithelial cells (GEC) (cluster 14) expressed *Cited4*, *Epcam*, *Crabp2* and *phyh2* gene markers. Smooth muscle cells (cluster 15) highly expressed *Des*, *Myh11*, *Acta2*, and *Tpm2* [29, 36]. In addition, the *PPP1R14A* gene highly expressed in cluster 15 could inhibit the myosin phosphatase and lead to increased phosphorylation of myosin then enhanced smooth muscle contraction. Cluster 20 highly expressed *SOX1*, *S100* [37], *MPZ* [38], *NCAM* [39-41], *SCN7A* and *CRYAB*. *SCN7A* is one of the many voltage-gated sodium channel proteins. *CRYAB* is highly expressed in many neurological diseases, and the protein encoded by the *S100B* gene might play a role in Ca^{2+} flux stimulation and promoting astrocyte hyperplasia. The chromosomal rearrangement or expression change of S100B is associated with neurological diseases such as Alzheimer's disease, Down's syndrome and epilepsy. Thus, cluster 20 cells were defined as Swann cells. Cluster 19 cells highly

expressed *Alas2*, *Bpgm* and *Mkrn1* were identified as erythrocyte precursor cell [23]. Next, we focus on the cell clusters with increased number of cells in musk secretion stage (**Table S8**), which included cluster12, cluster14, cluster17 and cluster21. We first compared the differentially expressed genes (DEGs) ($|\log_2 FC| >= 0.25$, corrected *P* value <0.05) of the same cluster in different stages (**Fig. S4a**). Then we conducted gene enrichment analysis of these genes (**Fig. S4b**). Intriguingly, the DEGs in cluster 14 were involved in “Regulation of hormone levels”, “lipid biosynthetic process”, and “organic acid transport” pathways, which are related to musk secretion.

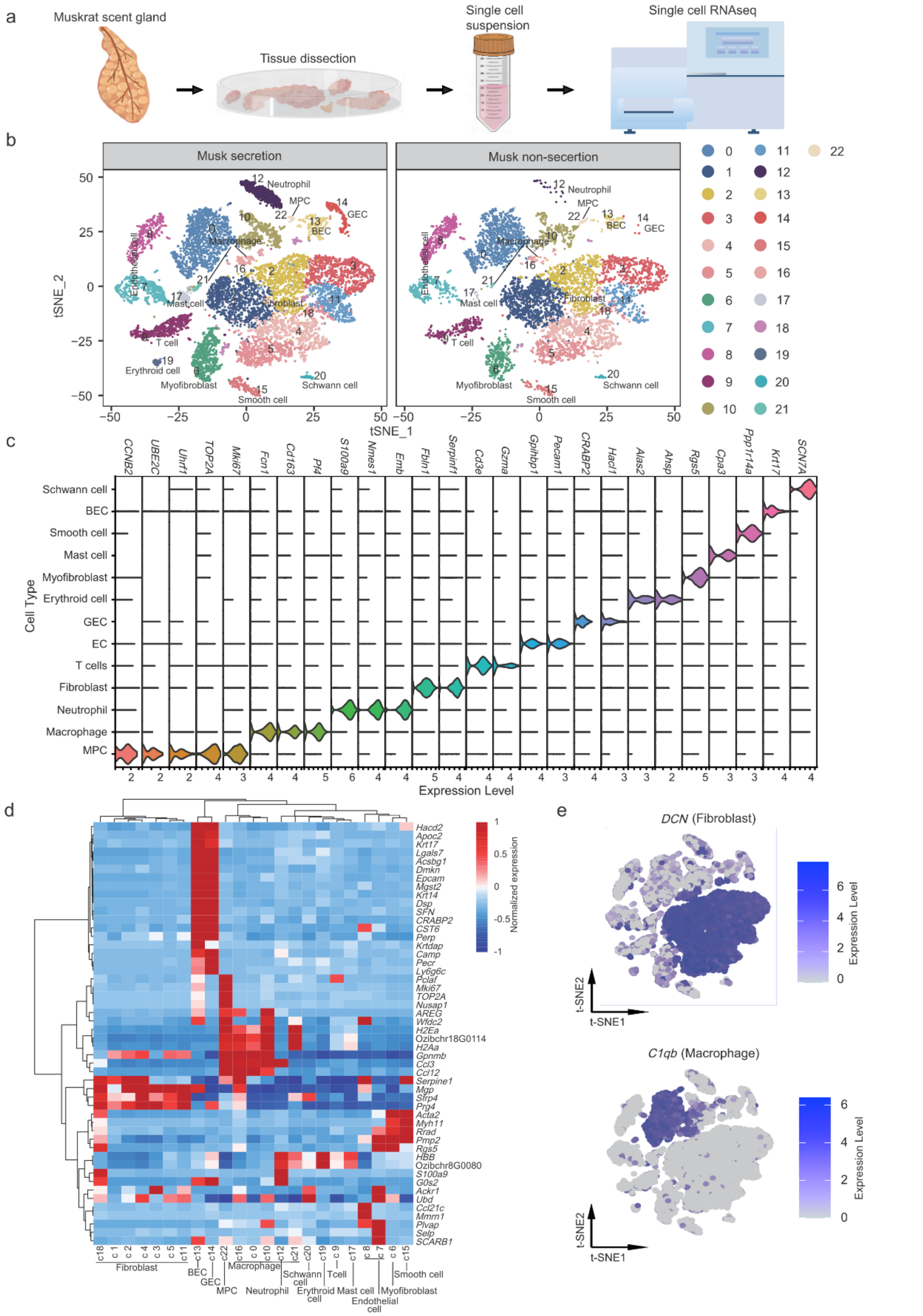

Figure 3: Transcriptome map of muskrat analyzed. (a) Schematic representation of muskrat musk gland tissue preparation for single cell transcriptome analysis. (b) tSNE and UMAP cluster map revealing 23 specific clusters representing the major musk gland cell types. (c)Violin plots showing expression of representative differentially expressed genes for each cluster. (d) Heatmap and hierarchical clustering of cell clusters based on the expression of the top 50 most variable genes. (e) tSNE cluster map showing expression of genes characteristic of the major musk gland cell types. Red dashed lines give the boundaries of the main clusters of interest.

Pseudotime reconstruction traces of the origin and specification of acinar cells/glandular epithelial cells

Our scRNA results showed that acinar cells/glandular epithelial cells (cluster 14) have an increased number of cells in musk secretion stage (with 2.76% and 0.12% in musk secretion and non- secretion stages respectively), and this cell type is related to musk secretion. Thus, we further explored the origin and differentiation of the cells of this cluster. Acinar cells/glandular epithelial cells (cluster 14) expressed *Cited4*, *Epcam*, *Crabp2* and *phyh2* gene markers [23]. We used cell lineage trajectory analysis to elucidate the origin and differentiation of the acinar cells/glandular epithelial cells in muskrat. Pseudotime analysis based on transcript profiling enabled a clear reconstruction of acinar cells/glandular epithelial cells in the male muskrat gland.

The high concordance of scRNA status between cluster13 and cluster14 indicated that muskrat acinar cells/glandular epithelial cells derive directly from the basal cells, and the basal cells derive directly from the mesenchymal progenitor cell (**Fig. 4a, b**). This scenario on the biological origin of glandular epithelial cells was consistent with the scRNA of mouse mammary epithelial cells by Han et al. [42]. Figure 5a shows these trajectories among cells of clusters 13, 14 and 22. Most cells followed one pathway, whereas a smaller but significant population followed a different pathway. The markers for acinar cells/glandular epithelial cells (*CRABP2*, *Hacl1*, *Eci2*, and *Pecr*) of cluster 14, found by pseudotime reconstruction, displayed high and specific expressions compared with the other two clusters (**Fig. 4c, d**). These genes were mainly involved in lipometabolic functions. For example, *CRABP2* is involved in the metabolism and transportation of retinoic acid from the cytosol to the RARs (retinoic acid receptors) located in the nucleus [43]. *Hacl1* is an enzyme that catalyzes the hydrolysis of long-chain fatty acids [44]. Enoyl-CoA delta-isomerase 2 (ECI2) is a protein that catalyzes the isomerization of unsaturated fatty acid intermediates during beta-oxidation, a process that breaks down fatty acids to produce energy [45]. Peroxisomal trans-2-enoyl-CoA reductase (PECR) is a protein that plays a role in the metabolism of fatty acids, specifically by reducing unsaturated and polyunsaturated fatty acids to their saturated forms in peroxisomes [46]. A previous study showed that muskrat musk contained fatty acids (29.32%) by gas chromatography–mass spectrometry, which are the main component of musk [47]. Combined together, these results indicated that the function of lipometabolism is important in the development and evolution of musk gland.

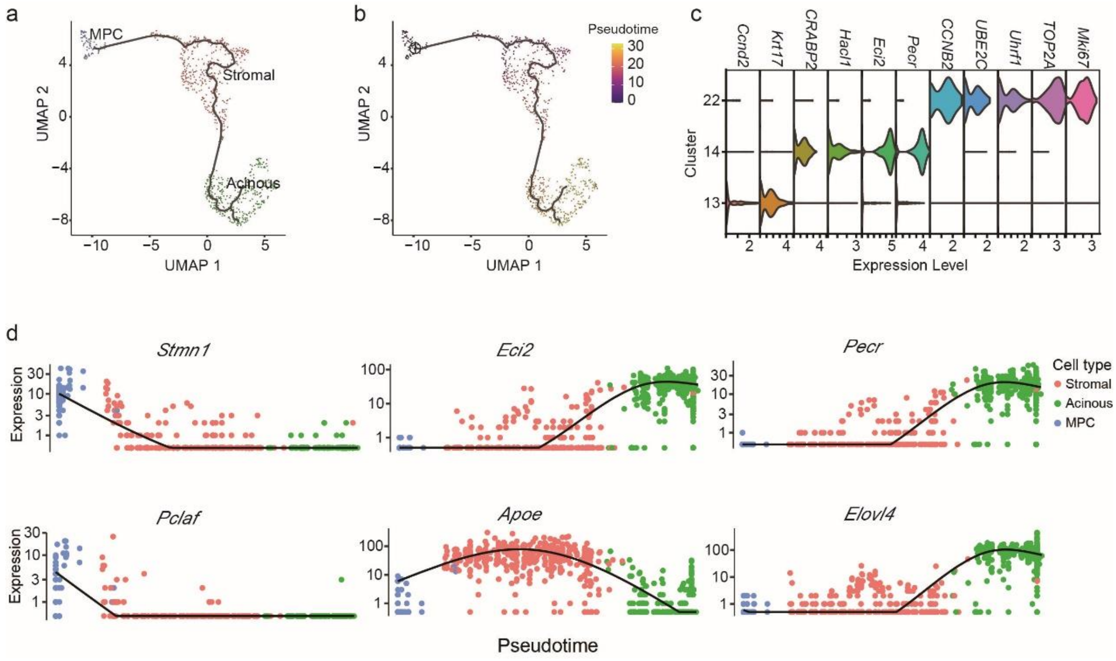

Figure 4: Specification and differentiation of mesenchymal progenitor cell, basal cell and acinar cells/glandular epithelial cell lineages. UMAP visualization of the muskrat mesenchymal progenitor cell (cluster 22), basal cell (cluster 13), and acinar cells/glandular epithelial cells (cluster 14). Cells are color-coded by cluster (a) and pseudotime reconstruction traces (b). (c) Violon plot of genes selected as population markers for each of the 13, 14 and 22 clusters. (d) Genes that displayed divergent expression patterns during acinar cells/glandular epithelial cells' generation in muskrat.

Dynamic changes in compartmentalization and TAD for musk secretion

In the eukaryotic cell nucleus, genomic DNA is highly folded and spatially organized into a hierarchy of 3D structures, including chromosome territories, compartments, topologically associating domains (TADs), and long-range interactions [48], which play important roles in transcriptional regulation [49]. To elucidate the multiscale regulatory rewiring of chromatin architecture during musk secretion, we used in situ Hi-C to map chromatin contacts for musk glands between the secretion and non-secretion stages. We generated a total of ~2.77 billion valid contacts (~692.44 million [M] contacts per sample (**Tables S9**) and reached a maximum resolution of 5 kb by merging the intrachromosomal contacts of the replicates at each stage) (**Tables S10**). Most (~54.29%) contacts occurred within chromosomes, exhibited high reproducibility among the biological replicates, and consisted dominantly (~57.82%) of long-range interactions (≥20 kb) (**Fig. S5a-d**). All samples showed strong decrease in contact probability with an increase in the distance between loci (**Fig. S5e**).

At the sub-chromosome level, we explored various compartmental rearrangement scenarios between musk secretion and non-secretion periods. Replicates of each stage shared similar A/B compartment patterns, ~44.4% and ~48.0% of the whole genome were Compartment A bins for musk secretion and non-secretion periods respectively (**Fig. S5f**). Compartment A was positively correlated with Guanine-Cytosine content

(Spearman's r > 0.60, $P < 2.20 \times 10^{-16}$) (**Fig. S5g-h**) and has a high gene density (**Fig. S5i**). We then constructed genome-wide inter-chromosomal contact maps by dividing the genome into 500-kb regions, it was revealed that the muskrat chromosomes have a similar likelihood to mutually contact each other during development: micro- and macrochromosomes tended to be self-associated, small and gene rich chromosomes preferentially contacted with each other more frequently (**Fig. 5a**). We identified substantial number of regions showing compartmental switching in the musk gland between two stages (~153.6 Mb, or ~6.2% of the genome) (**Fig. 5b**). In these regions, most switching was from A to B (120.80 Mb, embedded with 781 genes), which indicated that these regions were more closed in the musk secretion stage compared with the non-secretion stage. The rest were transient switches, from B to A (32.8 Mb, embedded with 164 genes) (**Fig. 5b, c**). Because these active chromatin regions are of potential functional significance, we further checked the genes located in regions that were subject to B-to-A switching events. They were primarily involved in "epithelial cell differentiation", "nephron development", "epidermis development", "cytoplasmic translation", "extracellular matrix organization", "negative regulation of endopeptidase activity", "cell morphogenesis", "protein activation cascade", "epithelial cell development", "intracellular steroid hormone receptor signaling pathway" and "regulation of membrane potential" processes (**Fig. 5d**). For example, *ROS1* and *SOX9* (involved in the differentiation of stem cells into various cell lineages and plays a role in the maintenance of tissue homeostasis), were specifically located in the compartment A region at the musk secretion stage (**Fig. 5e**). Similarly, we found that *NUS1*, which is highly expressed in GEC of musk secretion, undergoes B to A switching. NUS1 encodes Nogo-B receptor (NgBR), which is involved in many important cellular processes, such as cholesterol transport, lipid metabolism and neurodevelopment [50]. In addition, we also identified *NPNT* in GEC differential genes, in REG and in genes with B to A switching. *NPNT* encodes extracellular matrix proteins that participate in a variety of cellular processes and play an important role in regulating cell adhesion, differentiation, spreading, and survival [51]. These results suggest that two important functions of the musk gland during the musk secretion stage are cell specialization and lipid metabolism.

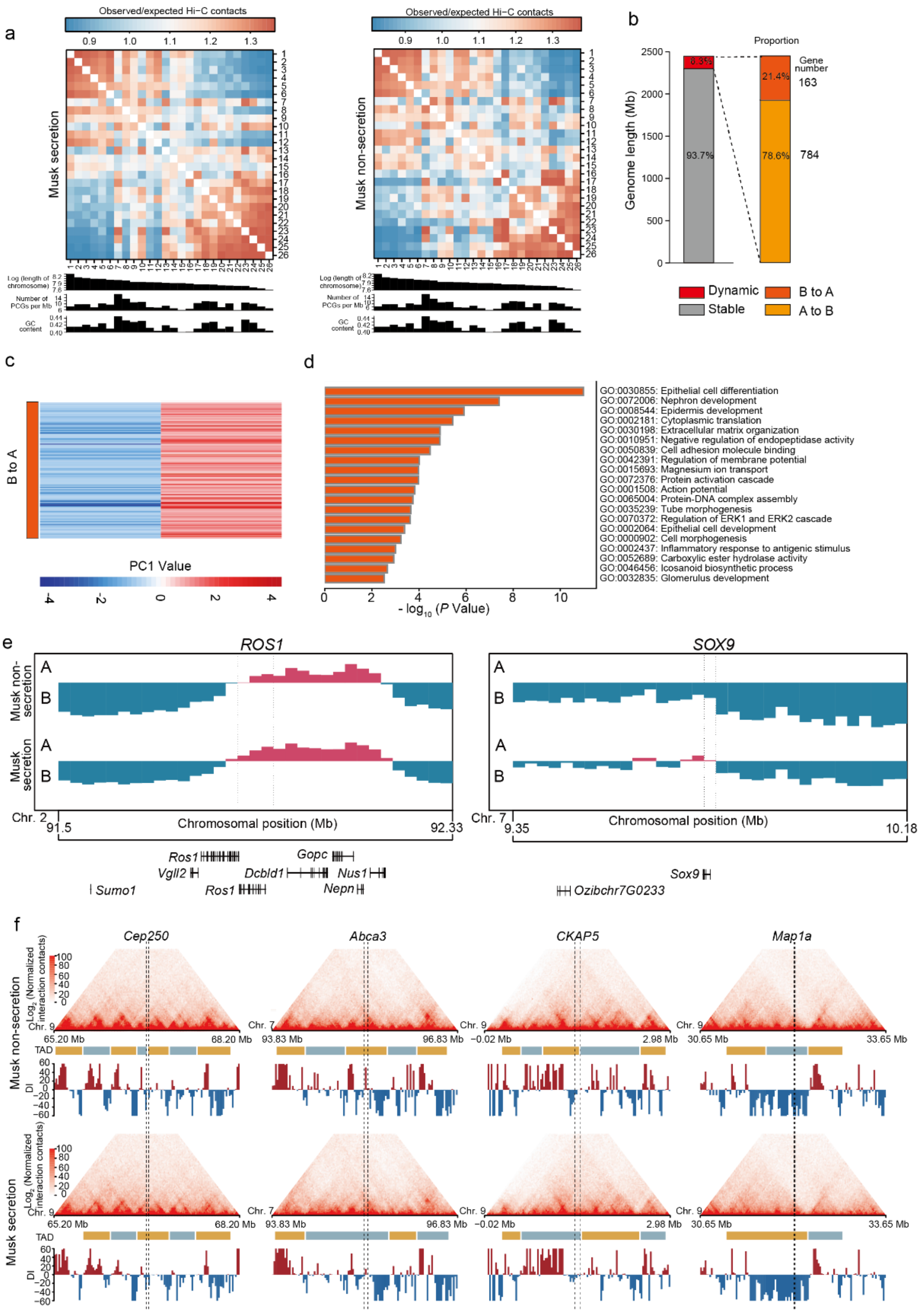

Figure 5: Global chromatin interaction patterns in musk gland of musk secretion and non-secretion stages. (a) Observed/expected contact matrices between chromosome pairs. Also shown are the length, gene density, and GC content of each chromosome. (b) Genomic lengths and proportions of stable and dynamic compartments. Dynamic compartments are classified into two types of transitions (A to B, and B to A). (c) Heatmap of the PC1 values for the compartment B to A switching regions. (d) The most enriched GO-BP terms for genes within B to A switch regions. (e) Three representative functional genes (red) subject to compartment switching between musk secretion and non-secretion stages, including *ROS1* and *SOX9*. The dashed line boxes indicate the chromosomal locations of the interested genes. Gene structures are indicated below the tracks. The

black arrows indicate the direction of the gene transcription. (f) TAD rewired in musk gland of musk secretion and non-secretion stages. The directionality index (DI) score changes between musk secretion and non-secretion stages for all regions in-between two consecutive TAD boundaries (centre marked by triangle) were shown. Examples of changed TADs emerging in each stage showing (top panel: musk non-secretion stage, bottom: musk secretion stage).

At the TAD structure level, we first used the directionality index (DI) score and a Hidden Markov Model (HMM) algorithm implemented in the TADtool software [52] to assign TAD boundaries. A total of 2,969 and 3,438 TADs were subsequently detected in musk secretion and non-secretion stages in the musk gland, with a median size of ~733.50 kb (**Fig. S6a**). We observe that only 54% (2,256) of the positioning of TADs remains stable between two stages (**Fig. S6b**), many changes in chromatin structure occur. Then we used the insulation score (IS) to evaluate the overall extent of changes in chromatin conformation between the two stages. For the TAD with increased IS in musk secretion stage, the content genes were involved in the "Regulation of proteolysis", "Response to radiation", "Regulation of lipid metabolic process", and "Transcription coregulator activity" pathways (**Fig. S6c**). Which indicated that the active function of regulation of lipid metabolism in the musk secretion stage.

Global rewiring of PEIs underpinning functional divergence during musk secretion

The interactions between enhancers and their target-gene promoters (PEIs) are an important part of the gene regulatory process and could be causally related to spatiotemporal expression [53]. Therefore, we next compiled an extensive genome-wide catalog of PEIs (median size of ~75 kb that primarily existed in TADs [59.53%]) in musk gland tissue (**Fig. S7a-c**) for each stage at a 5 kb resolution using the PSYCHIC algorithm [54]. PEIs cannot be reliably inferred from a genomic distance, therefore, we observed that ~87.98% of enhancers interacted with a more distant promoter instead of those closer by (**Fig. S7d**). This spatial proximity data highlights the complexity of PEIs [55, 56].

Our results demonstrated that candidate loci can be analyzed in future studies of musk secretion mechanisms. Typically, *Synpo2* (which enables alpha-actinin binding activity and filamin binding activity, involved in positive regulation of actin filament bundle assembly and positive regulation of cell migration [57]), *Egfr* [58] (the protein EGFR is a cell surface protein that binds to epidermal growth factor, thus inducing receptor dimerization and tyrosine autophosphorylation leading to cell proliferation and differentiation in various tissues), and *F3* [59](this factor enables cells to initiate the blood coagulation cascades) showed more PEIs and contacted with more enhancers during the musk non-secretion stage, though these interactions and number of contacted enhancers decreased in musk secretion stage (**Fig 6**). These results indicated that genes in the musk non-secretion stage play important roles in cell proliferation, differentiation and migration, which may provide the foundation for acinar cells/glandular epithelial cells generation. On the other hand, *Gins4* [60], *Cyld* [61], *Sil1* [62], *Map3k1* [63]

(which are related to binding and enzyme activity) and *Slc38a2* [64] (which is related to symporter activity and amino acid transmembrane transporter activity), exhibited more PEIs and were regulated by more enhancers during the musk secretion stage. These protein-binding activities including protein tyrosine kinase activity and protein kinase binding activity and so on were much active in the musk secretion stage, which indicate that synthesis and secretion activity were very active.

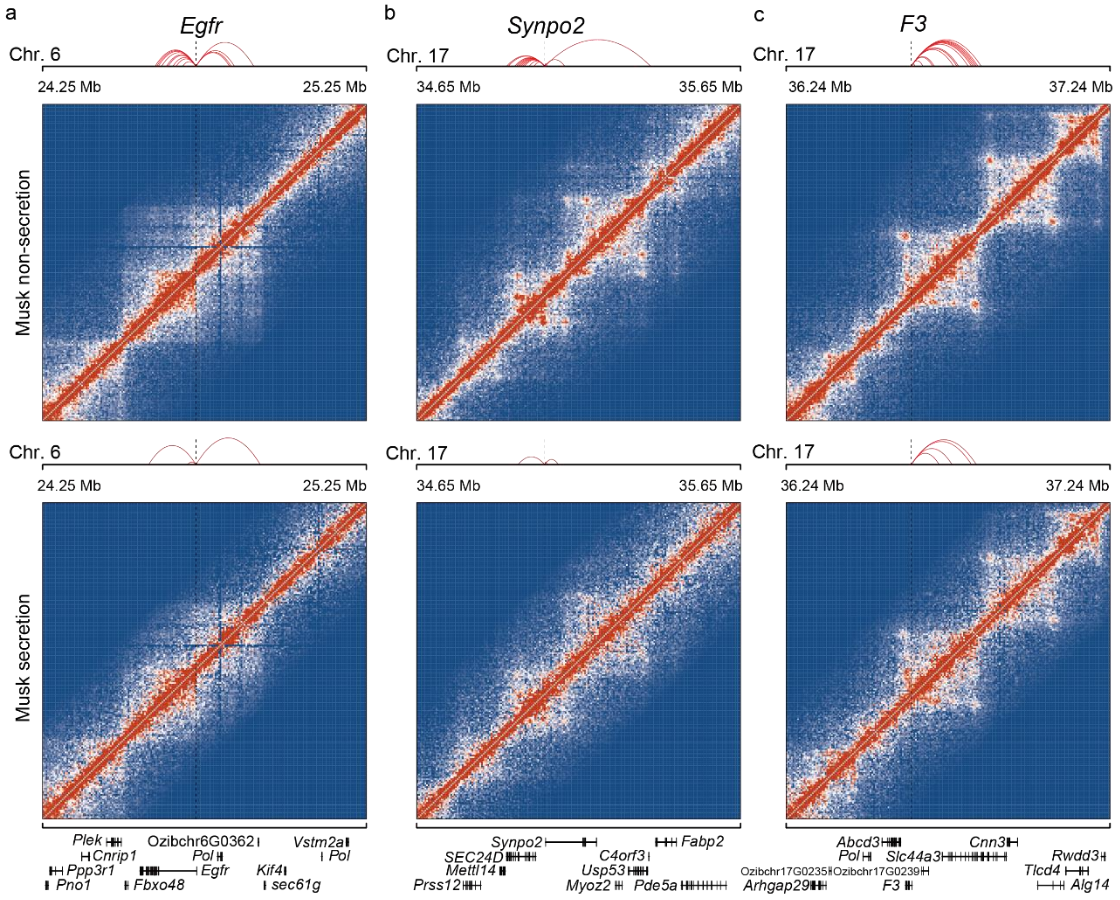

Figure 6: Promoter-enhancer interactions (PEIs) rewired in the musk gland of musk secretion and non-secretion stages. (a-c) PEI rewiring of a functional gene *Egfr*, *Synpo2*, and *F3* between two stages. Top: schematics of PEIs and Hi-C contact heatmaps of the genomic region containing *Egfr*, *Synpo2*, and *F3*. Bottom: gene structures in the region. The dashed line boxes indicate the chromosomal locations of the genes.

Positive selection and rapid evolution genes in muskrat and musk deer were mainly involved in metabolism of lipids and epithelial regulation

To explore the function of positively selected genes (PSGs) and rapidly evolving genes (REGs) in muskrat and musk deer during evolution, and together with chromatin structure changed genes. We next analyzed 7,409 gene trees based on one-to-one orthologs, each constrained to the reconstructed species phylogeny. By applying branch tests and branch-site tests in PAML [65] to the corresponding branches, we identified

443 rapidly evolving genes (REGs) and 399 positively selected genes (PSGs) for muskrats, 523 REGs and 199 PSGs for musk deer respectively. Interestingly, the gene *Hdac1* was in GO term "epidermal cell differentiation", which is enriched by gene family expansion in the forest musk deer and muskrat, coinciding with the musk gland specific gene. Of these respective sets of genes, they were mainly involved in the "Metabolism of proteins" and "Metabolism of lipids" (**Table S11, S12**). We also found that PSGs like *ANAPC4*, *CDC16*, and *RBL2* were involved in the cell cycle pathway (**Fig. S8a**). In addition, convergent sites refer to both previously defined "parallel" and "convergent" sites [66] were also analyzed. Based on the phylogenetic tree shown in Fig 1a, amino acid sequences at each node for all the 7,409 single-copy orthologs were deduced under the "aaml" model using CODEML in PAML [65]. As a result, 244 genes were detected as convergent evolution for muskrat and musk deer. Consistent with the result of REGs and PSGs, we also performed separate functional gene enrichment analyses for convergent evolution genes, and found that the gene sets were also significantly enriched for "Metabolism of proteins" (P = 0.041, Fisher's exact test), "Metabolism of lipids" (P = 0.018, Fisher's exact test).

RDH8 is a convergent evolution gene involved in "The canonical retinoid cycle in rods (twilight vision)" in muskrat and musk deer. Although there are olfactory receptor genes contracted in these two species, several vision related genes were PSGs or convergent evolution genes, indicating they have a sensory trade-off otherwise observed in arboreal species [67] and giraffe [68]. Which is consistent with the both species are timid and sensitive. TEX15 is a testis-specific protein, is required for TE silencing. A previous study found that TEX15 as a new essential epigenetic regulator may function as a nuclear effector of MILI to silence TEs by DNA methylation [69]. In our study we found that *TEX15* gene is an outlier with six unique amino acid convergent substitutions for muskrat and musk deer (**Fig. S8b**). Which indicated that the *TEX15* gene not only play a role in male germ cells, but it might also have an important role in the formation of the male characteristic organ of a musk-secreting species. Notably, among the genes specifically expressed in musk gland (**Fig. 3f**), *KRT80* and *MPIG6B* genes were also PSGs in muskrat.

For "metabolism of lipids" pathway, we identified 14 convergent evolution genes (*SLC44A2, GBA, RUFY1, PTGES2, GPAT3, PIK3R5, ACSL5, PIK3C2B, MED12, PIP5K1B, CYP27B1, MCAT, BDH2, ACBD6*) in this pathway (P value = 0.018, Fisher's exact test) in muskrat and musk deer (**Fig. 7a**). We also checked the overlapped genes between PSGs and convergent evolution genes, and found *NOP2*, *DST* and *FAM160A1* were shared between them (**Fig. 7b**). These genes are limited studied, such as FAM160A1 is a member of the UPF0518 family of proteins, each containing a conserved retinoic acid induced 16 (RAI16)-like domain with unknown biological function [70]. Among these convergent evolution genes, *ACSL5* and *GBA* have two

amino acids substitutions (**Fig. 7c**). The protein encoded by the *ACSL5* gene is an isozyme of the long-chain fatty-acid-coenzyme A ligase family, which catalyzes the formation of fatty acyl-CoAs from long-chain fatty acids (C16–C20). Fatty acyl-CoAs are then used in lipid synthesis or β-oxidation mediated pathways [71]. *GBA1* gene encodes the lysosomal enzyme beta-glucocerebrosidase (GCase) that degrades glucosylceramide and is pivotal in glycosphingolipid substrate metabolism [72]. In addition, choline is essential for the synthesis of phospholipids [73], and the gene *SLC44A2* participated in the process. These results indicated a rapid evolution of lipid metabolism in these two species. In addition, some convergent evolution genes have roles in "cell differentiation" ($P = 0.013$, Fisher's exact test) were also detected (**Tables S9 and S10**). This might be because the male musk gland has a cyclic change, in the stage of musk secretion stage, the musk gland is atrophic, and in the stage of musk non-secretion, the glands become larger accompanied with cell proliferation and differentiation.

In addition, for the DEGs in cell cluster 14, we also found that five genes overlapped with REG, including those associated with carboxylic acid transport (*SLC26A2*), transcription factor activity (*ZNF317*), Cell division (*NCKAP51*) and collagen-containing extracellular matrix (GPC4 and COL6A3). It is worth mentioning that *NCKAP51* is also a REG of forest musk deer. Studies in closely related species have found that it targets mir-2425-5p to regulate the proliferation and differentiation of bovine myogenic satellite cells [74]. GPC4 is differentially expressed in dental epithelial and mesenchymal cells [75], also expressed in renal epithelial cells to regulate epithelial branching morphogenesis [76]. COL6A3 encodes collagen type VI and is normally expressed in tumor epithelial cells to promote invasion and metastasis [77]. Two PSGS, cyclin (*WEE1*) and integrin ligand (*NPNT*) coincided with the differential genes of GEC. *WEE1* can regulate cell division by mediating G2/M phase progression of epithelial cells [78]. *NPNT* was found to be highly expressed in epithelial and mesenchymal cells of the tooth germ and regulated the differentiation of Sox2+ cells in dental epithelial cells through the EGFR-PI3K-Akt signaling pathway [79]. These genes provide evolutionary evidence for the epithelial-mesenchymal transition of muskrat musk gland during musk secretion and non-secretion. Furthermore, the PSG or convergent evolution genes (**Fig. S8c**) involved in TAD changing were also related to metabolism of lipids and epithelial regulation pathways, such as *NAGS* catalyzing the production of N-acetylglutamate (NAG), an important substance that regulates urea synthesis [80]. *CEP250* plays a crucial role in differentiating spermatogonia and meiotic spermatocytes [81]. *CASP8AP2* play a role in regulating cell proliferation, apoptosis and gene expression [82].

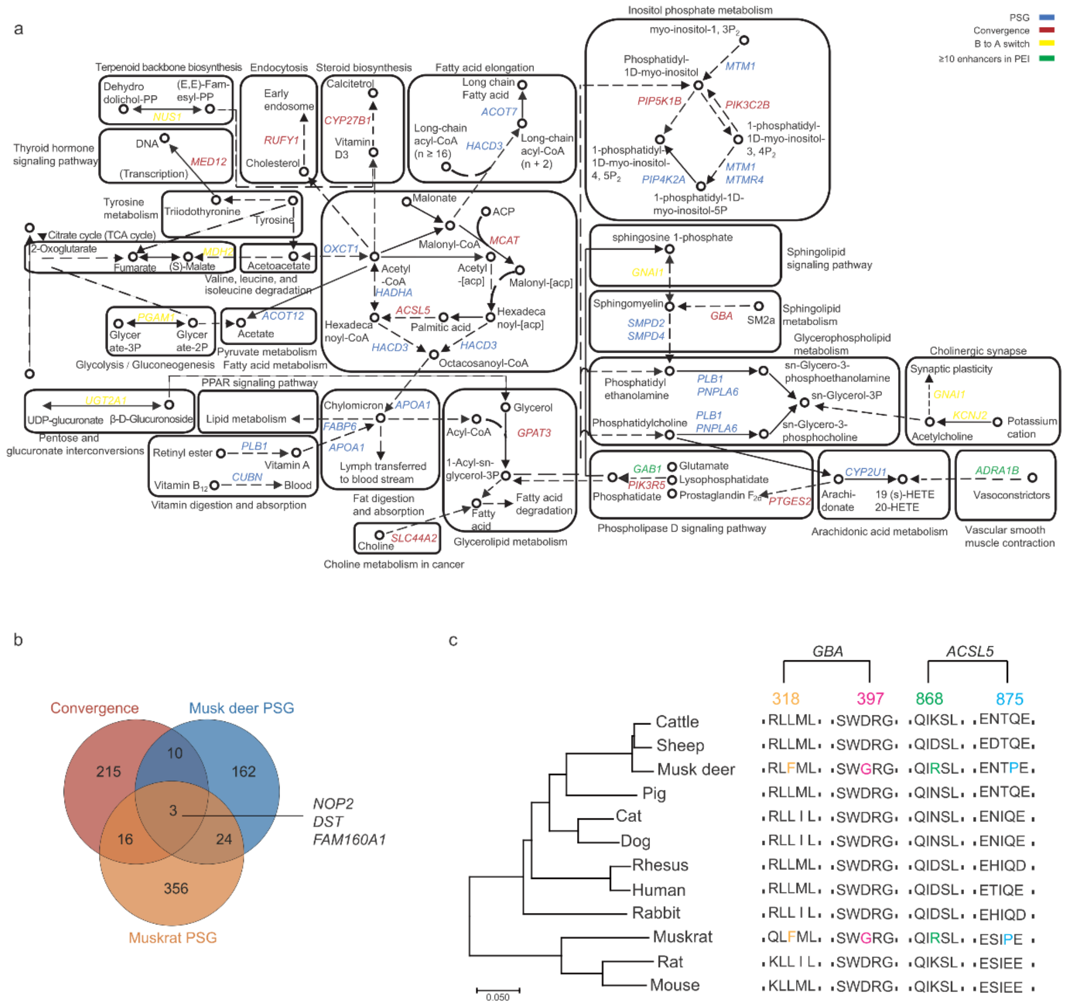

Figure 7: The adaptive evolution of muskrat and musk deer. (a) Enrichment for PSGs, genes evolved in parallel, B to A switched and PEI changed that functions in metabolism of lipids. The PSGs are shown in blue, and the parallel genes are shown in red. (b) The sequence alignment shows sites that evolved in parallel in *GBA* and *ACSL5*.

The MuskDB platform

According to the above described sequencing data, we generated a database (MuskDB). MuskDB contains 47,635 gene entries from the genomes of two species, with information on 831 biological pathways,103 bulk RNA transcriptomes, two single cell transcriptomes and three Hi-C datasets. The bulk transcriptome data were derived from different tissues, and secretion conditions of adult individuals (**Table S1**). The single cell transcriptome data including two secretion stages of muskrat, which could be classified to 23 cell clusters. Expression heatmap of each gene in each cluster and tissue could be accessed using searching tool on the platform. The platform also holds three Hi-C data (secretion stage in July and non-secretion stage in October of muskrat musk gland, and blood sample of musk deer), including 343,392 promoter and enhancer

interactions. The Pan-JBrowse browser in MuskDB displays all genes structure of muskrat and musk deer. Thus, MuskDB contains the largest amount of musk secretion-related multi-omics data up to now, with online tools facilitating data exploration. At the homepage, the tools include “Blast”, “Sequence Fetch”, “Gene Sequence Extraction”, “Transposable Elements”, “Gene Synteny Viewer”, “Phylogenetic Tree”, “Gene Expression”, “Single Cell Expression” and “Hic Search” (**Fig. 8a**). MuskDB provides comprehensive information on muskrat and musk deer genes, including their annotation, location and expression. The heat maps in ‘Gene Expression’ and ‘Single Cell Expression’ show the expression of genes respectively in 13 tissues (**Fig. 8b**) and 13 different cell types (**Fig. 8c**). In ‘Hic Search’, users could enter a gene name or genome region to show the contacts information of this gene or in this region. For example, when gene *Synpo2* is entered, the results showed the contacts profile of this gene in two stages of secretion and non-secretion (**Fig. 8d**).

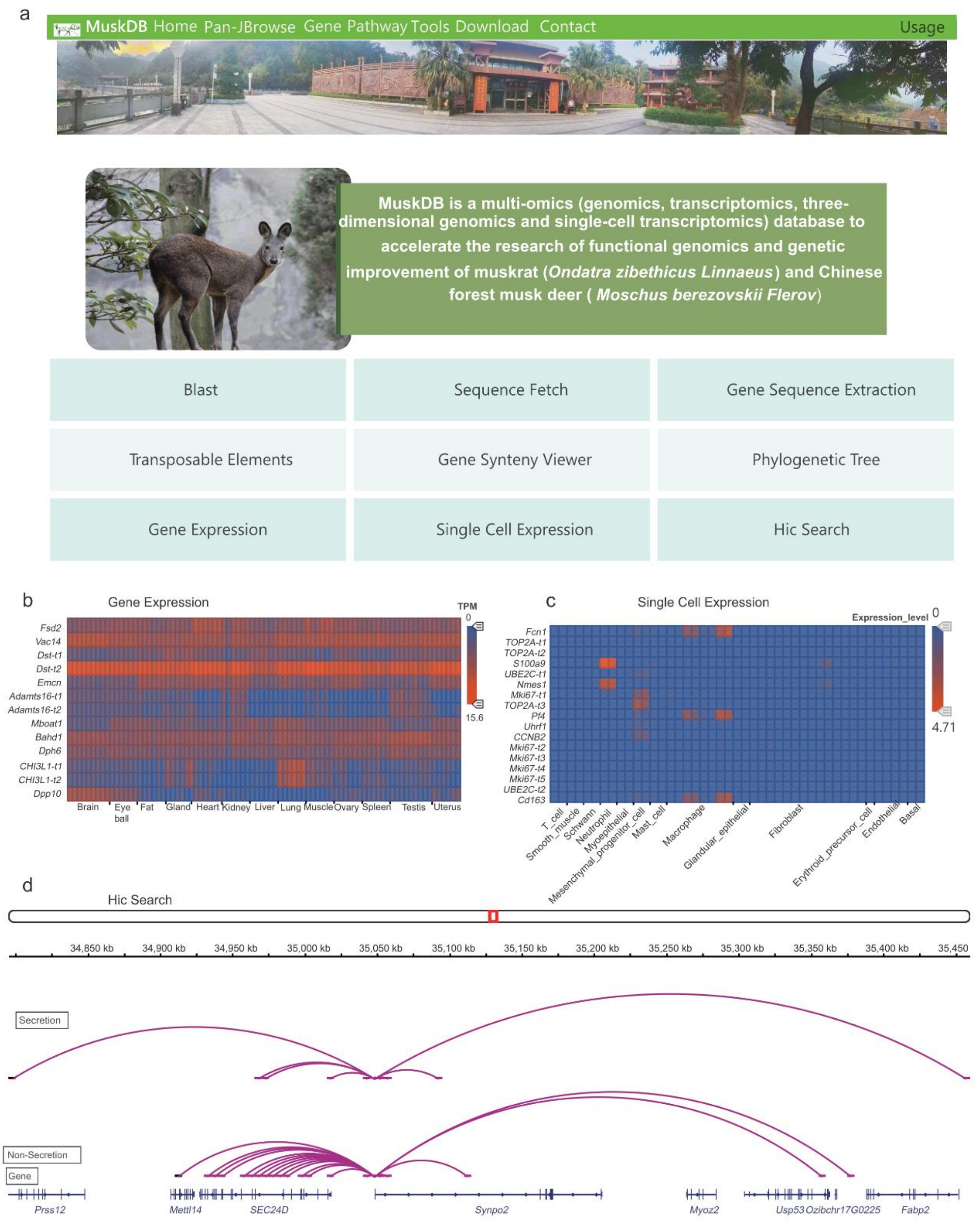

Figure 8: Overview of MuskDB and its application in musk secretion animal's functional genomics. (a) MuskDB homepage contents, showing available online tools. (b) The expression pattern of genes in different tissues shown by MuskDB. (c) The expression pattern of genes in different cell clusters shown by MuskDB. (d) Hi-C contacts of gene *Synpo2* in musk secretion and non-secretion stage shown by MuskDB.

Conclusion

This study provided an open-source, web-accessible, user-friendly multi-omics database platform of musk secretion animals, including two high-quality genome

assemblies of musk secretion species (muskrat and musk deer), a BodyMap transcriptome of muskrat, and comprehensively analyzes Hi-C, RNA-seq and scRNA-seq between musk secretion and non-secretion stages of muskrat *in vivo*. GO terms of stage-specific signature genes of musk glands were identified by scRNA-seq and emphasized significant functional differences between the musk secretion and non-secretion stages. Because of their unique evolutionary adaptations, the muskrat and musk deer have always been a topic of interest on animal evolution and physiology. Our high-quality genome assembly provides more precise comparative insights into the genetic basis of their biological features. We identified genetic changes underlying adaptations of both species to the musk secretion, notably related to the adaptation of lipid metabolism, cell cycle regulation, and sensory perception. Our single cell RNA sequencing results may potentially facilitate the development of new strategies for the organoid culturing of musk gland. Overall, these results show that active lipid metabolism may underlie the adaptation evolution of musk secretion. However, because of the complexity of musk gland, more research on the functional consequences of musk secretion-specific genetic variants is perspectively needed.

Materials and methods

Methods

Genome sequencing

Blood from a muskrat and a forest musk deer were collected from the Chongqing Institute of Medicinal Plant Cultivation, following the ethical guidelines for Animal Care and Use of Chengdu University. High-quality DNA was extracted from 20 mL of muskrat and forest musk deer blood using a DNeasy Blood & Tissue Kit (Qiagen, Valencia, USA) according to the manufacturer's instructions. Some of this DNA was then used to construct Nanopore libraries, which were sequenced using GridION X5 sequencers (Oxford Nanopore Technologies, Oxford, UK), and the remainder of the DNA was used to construct re-sequencing (NGS) libraries with an insert size of 400 bp, which were sequenced using the BGI T7 platform. The musk gland samples at musk secretion and non-secretion stages were used to construct Hi-C libraries, which were subsequently sequenced using the Illumina NovaSeq platform (Illumina).

Genome assembly and assessment

We used an optimized four-step genome assembly strategy[7] to generate complete assemblies of muskrat and musk deer genomes. First, we used the high-quality Nanopore sequences and applied a 'correct-then-assemble' strategy using NextDenovo (v2.5.0; https://github.com/Nextomics/NextDenovo) to assemble the initial contigs. The high-quality of T7 paired-end reads and Nanopore long reads were utilized for the correction of the initial contigs using the software NextPolish (v1.4.1) with its 'best' algorithm modules[83]. Thus, we yielded the ContigV1 assembly. Second, we

generated unique mapped pairs by aligning the Hi-C read pairs to ContigV1 using Bowtie2 software[84] with a single-ended model. We then discarded invalid self-ligated and unligated fragments using the HiCUP pipeline (version 0.8.0) [85]. We obtained the valid interaction pairs and used to calculate linkage frequency among all contigs using an agglomerative hierarchical clustering algorithm. The linked contigs were then clustered based on the Hi-C signal density, indicating potential homologous chromosome associations. Third, we realigned the Nanopore reads to ContigV1 using package Minimap2[86]. Suboptimal alignment reads were removed, and mapped reads of each contig group were extracted. Local assembly was performed for each classified mapped read to avoid false overlap relationships caused by repetitive sequences during assembly[87]. All contigs were corrected again using appropriate parameters, similar to the first step. Forth, chromosome-scale genomes were anchored using linkage information, restriction enzyme site, and string graph formulation with the ALLHiC algorithm [88]. Any placement and orientation errors displaying distinct chromatin interaction patterns were manually adjusted.

To assess the quality of genome assemblies, we used the Merqury package to evaluate assembly precision by measuring QV values [11]. BUSCO analysis (version 5.2.1)[10] was used to assess the assembly completeness by searching against 9,226 conserved mammalian genes from the mammalia_odb10 database. Additionally, T7 paired-end reads were aligned to the assembled genome using BWA software[89] to calculate the alignment ratio and coverage depth, for assessing the assembly completeness.

Transposable element (TE) annotation

We predicted the genome TEs by utilizing a combination of homology searching and *ab initio* prediction methods. We performed the homology searching by applying the RepeatMasker [90] and RepeatProteinMask packages to compare the genome against the Repbase TE library. In parallel, we performed *ab initio* prediction by constructing a reference repeat library using the results from PILER[91], LTR FINDER[92], and RepeatScout[93], and then searching the genome against this library using RepeatMasker. In addition, we employed the Tandem Repeats Finder package[94] with specific parameters ("2 7 7 80 10 50 2000 -d –h") to predict tandem repeats in the genome.

Protein-coding gene prediction

We predicted protein-coding gene models via integration of homology- and *ab initio*-based methods, with additional evidence from transcription data. In the homology-based approach, we used protein repertoires from model mammalian species such as *Homo sapiens* (GCA_000001405.28), *Mus musculus* (GCA_000001635.8), *Equus caballus* (GCA_002863925.1), *Canis lupus familiaris* (GCA_000002285.2), *Rattus norvegicus* (GCA_015227675.2), *Sus scrofa* (GCA_000003025.6) and *Bos taurus*

(GCA_002263795.2) were used as queries. These queries were searched against the target genome using the TBLASTN algorithm [95]. The resulting BLAST hits were conjoined using the Solar (Sorting Out Local Alignment Results) to obtain a comprehensive set of alignments. Next, the gene structures within each BLAST hit were determined using the GeneWise pipeline[96], allowing us to define gene models with high accuracy and specificity. Subsequently, we obtained transcriptomic data for muskrat and muskdeer from the NCBI database, with accession numbers listed in **Table S1**. The RNA-seq data were aligned to the genome using the Tophat software[97], enabling the identification of potential exonic regions and splicing junctions. Gene models, represented by the Cufflinks-set, were then assembled from the mapped reads using Cufflinks[98]. In the *ab initio* method, we aligned the assembled transcripts with the assembled genome using the Program to Assemble Spliced Alignment (PASA) [99]. This allowed us to assemble the transcript alignments into gene structure models, which were then used as the training set for Augustus[100], SNAP[101], and GlimmerHMM[102] pipelines. With these training sets, we conducted ab initio prediction of coding regions in the repeat-masked genome using Augustus, GlimmerHMM, and SNAP. Furthermore, we employed GeneID[103] and GeneScan[104] to directly generate predicted gene models in the repeat-masked genome. After completing the aforementioned methods, the generated gene models were integrated using EVidenceModeler. We assigned weights to each type of evidence as follows: Homology-set > Cufflinks-set > Augustus > GeneID = SNAP = GlimmerHMM = GeneScan. Furthermore, we used PASA2 to update the gene models, incorporating untranslated regions and information on alternative splicing variations.

To annotate the protein-coding genes, we searched for functional motifs, domains, and information on the possible biological processes of the genes in established databases such as SwissProt[105], the NR database (from NCBI), and the KEGG (Kyoto Encyclopedia of Genes and Genomes)[106].

Identification of one-to-one orthologous genes

In addition to the gene sets generated from our two assembled genomes, we also downloaded gene sets from 10 other mammalian genomes including human, macaque, mouse, horse, cattle, sheep, pig, cat, dog and rabbit from the Ensembl database. We used these 12 gene sets to identify orthologous genes. To accomplish this, we first selected the longest translation to represent each gene and filtered our genes with fewer than 50 amino acids. Then, we performed an all-against-all BLASTP comparison with an E-value threshold of 1e-7 to determine similarities between genes across the 12 species. We extracted alignment pairs from each pair of genomes while restricting a maximum of five hits per protein sequence. These alignment pairs were used as input for the MCScanX algorithm[107], which helped detect collinear blocks of coding genes and identify orthologous gene pairs with high confidence. We specifically focused on

one-to-one orthologous genes between pairs of mammalian species.

After integrating a matrix of orthologous genes for the 12 mammalian species, we ensured that that each orthologous cluster included all the species. Subsequently, we performed multiple sequence alignment for these one-to-one orthologs using PRANK (v.170427) [108] and applied the Gblocks package (v0.91b)[109] to minimize the impact of alignment errors and divergent regions. Alignments shorter than 90 nucleotides were discarded to maintain quality. Through this process, we identified 7,409 one-to-one orthologs among the 12 species.

Phylogeny construction and divergence time estimation

We initially used MODELTEST[110] to analyze the codon alignments of one-to-one orthologs and determined that the general time-reversible (GTR) substitution model was the most suitable for the observed data. Therefore, we conducted phylogenetic tree for the 12 mammals using the maximum likelihood method implemented in the RaxML package[111]. The best-fitting substitution model "GTR+GAMMAX" was utilized, and 1,000 bootstrap replicates were performed to assess the robustness of the tree topology. To estimate divergence times, we employed the MCMCTree program from the PAML package (version 4.9) [65].

Gene family clustering, expansion and contraction analysis

We utilized the OrthoFinder package (v2.3.1) [112] to identify gene families by detecting orthogroups and paralogous genes based on the results of the all-against-all BLASTP analysis (see also the method "Identification of one-to-one orthologous genes"). The expansion and contraction of gene families were evaluated by comparing cluster sizes between the ancestral species and each of the 12 mammalian species. This analysis was performed using the Café program[13], which employs a probabilistic graphical model. By using conditional likelihoods as test statistics, we calculated *P-values* for each lineage and set a threshold of *P-value* < 0.05 to determine gene families that exhibited significant expansion or contraction.

Identification of positively selected genes (PSGs) and rapidly evolving genes (REGs)

We employed the CodeML program in the PAML package (version 4.9) [65] to identify PSGs and REGs based on the 7,409 orthologous genes. For PSGs, we utilized the free-ratio branch-site mode (model = 1) as an alternative model, assuming positive selection on the foreground branch. The null model allowed sites to undergo purifying selection or evolve neutrally. For REGs, we utilized the branch model, specifically the one-ratio model (model = 0) as the null model assuming the same evolutionary rate for all branches, and the two-ratio model (model = 2) as an alternative model allowing different evolutionary rates for the foreground branch. The likelihood ratio test (LRT) method was used to detect differences between the nested models, and *P-values* were computed based on $\chi 2$ statistics. Multiple testing was corrected using the false

discovery rate (FDR) method.

Functional enrichment analysis

The gene set enrichment analyses, including Gene Ontology (GO), KEGG pathway, and Reactome analyses, were performed using the KOBAS 3.0 software[113, 114], with human homologs as references. The statistical significance of enrichment was assessed using the binomial distribution test, and the P-values were adjusted for multiple testing using the Benjamini method.

Functional enrichment analysis

The GO, KEGG pathway and Reactome enrichment analyses for gene sets were all implemented by KOBAS 3.0 software based on human homologs [113, 115]. The binomial distribution test was used for statistical significance, and the P-values were corrected for multiple testing by the Benjamini-Hochberg method.

Animals and sample collection for RNA-seq for muskrat

To fully investigate the muskrat transcriptome, we used a total of 84 samples from 13 tissues (3 to 6 samples from each of the 13 tissues were collected). The healthy muskrats were isoflurane euthanasia, we adjust the isofurane flow rate or concentration to 5% or greater, and continue isoflurane exposure until one minute after the muskrats breathing stops. The required tissues were then collected using standard anatomical techniques, excess blood vessels and fat were removed in phosphate buffer solution, and then flash-frozen in liquid nitrogen and stored in a -80°C refrigerator for subsequent studies.

Muskrat transcriptome reconstruction

Total RNA was extracted from each sample using RNAiso Plus reagent (TaKaRa, #9108) according to the manufacturer's instructions. We estimated the integrity and quality of the total RNA using a Bioanalyzer 2100 system (Agilent Technologies, Palo Alto, CA, USA) and an RNA 6000 Nano kit. Eighty-four poly-A RNA-seq libraries were constructed. LncRNA and mRNA were then sequenced using the NOVAseq-6000 platform with a paired-end sequencing length of 150 bp (PE150) at Shenggong Bioengineering Co., LTD (Shanghai, China). MicroRNA was sequenced by the NEXTSEQ550 platform with a single-end sequencing length of 75 bp (SE75) at Shenggong Bioengineering Co., LTD (Shanghai, China). In total, we then generated a total of 1.37 Tb high-quality RNA-seq data and 27.13 Gb high-quality miRNA data (~13.54 Gb sequences per sample) (**Table S1**).

Clean reads were obtained after quality control filtering. Using Fast - toolkit software (http://hannonlab.cshl.edu/fastx_toolkit/) to remove the clean reads low-quality reads (quality value less than 30 bases accounted for more than 20%). The mRNA sequenced

reads were aligned to our self-assembled muskrat genome by the STAR alignment tool (version 2.6.0), with on average ~97% (~57.06 million) of aligned reads for each library. Read Counts were quantified using feature Counts (version 2.0.1). Gene-level transcript abundance was estimated as transcripts per million (TPM).

Gene transcriptional profiling across tissues

We calculated the tissue specificity of gene abundance reflected by the tau score (τ)[116](ranging from 0 to 1, with 1 for highly tissue-specific genes and 0 for ubiquitously transcribed genes) for each gene with scaled TPM values. For each tissue, we averaged all replicates and then calculated τ to account for unequal numbers of replicates among tissues. We used $\tau \geq 0.75$ as the cut-off for tissue-specific genes. We calculated the abundance distribution (i.e., transcriptome complexity) of distinct transcripts across tissues, reflected as the fraction of total RNAs contributed by the most highly expressed genes. Differential gene expression analysis was performed using edgeR (version 3.40.2) [117], with a Benjamini &. Hochberg adjusted P value ≤0.01 and $\log_2$(fold change) ≥1 as cut-offs for statistical significance.

Single cell preparation

After harvesting, musk gland tissues were washed in ice-cold RPMI1640 and dissociated using Demonstrated_Protocol_Adult_Mouse_Nuclei_Isolation_RevA. (10× Genomics Catalog No.CG000393 Rev A) from Miltenyi Biotec as instructions. DNase treatment was optional according to the viscosity of the homogenate. Cell count and viability were estimated using a fluorescence Cell Analyzer (Countstar® Rigel S2) with AO/PI reagent after the removal erythrocytes (Miltenyi 130-094-183) and then debris and dead cells removal was decided to be performed or not (Miltenyi 130-109-398/130-090-101). Finally fresh cells were washed twice in the RPMI1640 and then resuspended at 1×10^6 cells per mL in 1×PBS and 0.04% bovine serum albuminat.

Single cell RNA-seq library construction and sequencing

ScRNA libraries were prepared using Chromium Next GEM Single Cell 3′ Reagent Kits v3.1 (10× Genomics). Briefly, the appropriate number of cells were mixed with reverse transcription reagent and then loaded to the sample well in Chromium Next GEM Chip G. Subsequently Gel Beads and Partitioning Oil were dispensed into corresponding wells separately in the chip. After emulsion droplet generation reverse transcription was performed at 53 °C for 45 minutes and inactivated at 85°C for 5 minutes. Next, cDNA was purified from broken droplet and amplified in the PCR reaction. The amplified cDNA product was then cleaned, fragmented, end repaired, A-tailed and ligated to the sequencing adaptor. Finally, the indexed PCR was performed to amplify the DNA representing 3’ polyA part of expressing genes which also

contained Cell Bar code and Unique Molecular Index. The indexed sequencing libraries were cleanup with SPRI beads, quantified by quantitative PCR (KAPA Biosystems KK4824) and then sequenced on illumina NovaSeq 6000 with PE150 read length.

Single cell RNA sequencing data quality control

Fastp (v0.20.1) [118] was used to trim primer sequence and low quality bases of raw reads and collect the basic statistics. The specific parameters could be summarized as below: (1) A 4 bp sliding window was moved from the front (5') to tail. Once the mean quality of the bases in the window was below 10, the bases along with the subsequent bases, would be dropped, the leading N bases were also trimmed (--cut_front --cut_front_window_size 4 --cut_front_mean_quality 10); (2) A 1 bp sliding window was moved from tail (3') to front. The bases in the window were dropped if its mean quality was below 3, the trailing N bases were also trimmed, similar to the Trimmomatic TRAILING method (--cut_tail --cut_tail_window_size 1--cut_tail_mean_quality 3); (3) The auto adapter was detected for PE data (detect_adapter_for_pe); (4) The trimmed Reads shorter than 60 bp were discarded (--length_required 60). The cleaned reads after trimming were used in the following steps.

Processing the single cell RNA sequencing data

The Cell Ranger [119] Single-Cell Software Suite was used to perform sample demultiplexing, barcode processing and single-cell 3' gene counting (http://software.10xgenomics.com/single-cell/overview/welcome).
The clustering and visualization were finished by Seurat [120], with the following steps: (1) Data normalization. LogNormalize, a global-scaling normalization method, was employed to normalize the expression. The expression measurement of one transcript was divided by those of all the transcripts of the cell and multiplied by a scale factor (10,000 by default), and then the result was logarithmic transformed. (2) Detection of highly variable features. FindVariableFeatures was used to get 2,000 features per dataset. (3) Scaling. A linear transformation ('scaling'), a standard pre-processing step prior to dimensional reduction techniques, was applied. (4) Dimensional reduction. PCA on the scaled data was performed, and the first 15 principal components were used in the following steps. (5) Clustering. A graph-based approach was applied to cluster the cells. (6) tSNE/UMAP. The non-linear dimensional reduction technique was used to visualize and explore these datasets. (7) Cluster markers. FindAllMarkers with the default parameters except "logfc.threshold=1" was used to find markers that determined the cell clusters via the differential expression, and the top 9 markers were visualized.

Human, mouse and rat miRNA and miRNA precursor sequences were downloaded

from miRBase (Version 22.1). The software miRdeep2 (version 0.1.2) was used to first index the muskrat genome, and then the data we had initially processed were converted into the format required by the software and compared with the muskrat genome. Finally, the sequences were compared with known miRNA sequences and miRNA precursor sequences. Combined with the position of genome alignment, the matching degree was calculated to form the minimum free energy of stem-ring miRNA, to identify whether it is a muskrat miRNA. The prediction of new miRNA is similar. Mature miRNAs of muskrat relatives human, mouse and rat were selected as reference, and mirdeep2.pl of the software was used for prediction. The screening criteria for new miRNA should meet the miRDeep2 Score25 and have a secondary structure with p-value < 0.05 as candidate miRNA. Then, the information of new miRNA predicted by all samples should be counted, and the candidate new miRNA predicted by at least two samples should be considered as new miRNA. The number of miRNA reads between samples was counted by TPM (Tags per million) and its expression was calculated.

Hi-C data of musk gland tissue

Hi-C data processing

To process the Hi-C datasets, we utilized Juicer, an efficient open-source tool [121]. Initially, we aligned the high-quality Hi-C reads to the genome using the BWA-mem module. Subsequently, we eliminated abnormal, duplicate, and low-quality alignments (MAPQ < 30). Using the KR algorithm, we constructed a normalized contact matrix at different resolutions, including 5Kb, 25Kb, 100Kb, 500Kb, and 1Mb.

Resolution evaluation of the Hi-C matrix

To determine the optimal resolution for our Hi-C matrix, we divided the genome into window sizes ranging from 1Kb to 1Mb. For each bin, we counted the number of *cis* contacts, defined as any contact where one read mapped within that bin, and calculated the percentage of bins with contacts greater than 1000. We identified the minimum window size with a percentage greater than 80 as the optimal resolution for our Hi-C matrix.

Identification of compartment A/B at the resolution of 100 Kb and 25 Kb

At a resolution of 100 Kb, compartment A/B analysis was performed as previously described. Briefly, a Pearson correlation matrix was generated using the 'cor' function in R. The first three principal components were then obtained by applying the 'prcomp' function in R to the correlation matrix. Bins at 100 Kb with positive Spearman's correlation between PC1 values and gene density were classified as compartment A, while bins with negative correlation were classified as compartment B.

For compartment A/B identification at a resolution of 25 Kb, the A-B index value was used as previously described, representing the comparative likelihood of a sequence interacting with A or B at a resolution of 100 Kb. Bins at 25 Kb with positive values

(indicating a greater association with A at 100 Kb) were identified as A compartments, while bins with negative values (indicating a greater association with B at 100 Kb) were identified as B compartments.

Identification of inter-chromosome interaction pattern

The patterns of inter-chromosome interactions were calculated following the previous protocol [122]. In brief, the observed number of contacts was normalized against the expected contacts in each inter-chromosome pair.

Identification of TAD

We identified TADs from the normalized contact matrix at a resolution of 25 Kb. We used the directionality index (DI) score and a Hidden Markov Model (HMM) algorithm implemented in the TADtool software with default parameters [52] to assign TAD boundaries.

Promoter-Enhancer interaction (PEI) analysis

We first combined the clean data of biological replicates and constructed the normalized contact matrix at 5 Kb resolution. We then used PSYCHIC software to generate raw PEIs [54]. We then filtered low confidence PEIs with interaction distance lower than 10 Kb or FDR greater than 0.001.

Expression analysis of mRNA, lncRNA and miRNA

We collected 84 samples of 13 tissues (including brain, eyeball, fat, gland, heart, kidney, liver, lung, muscle, spleen and testis) at the stage of musk secretion. All the samples were snap frozen in liquid nitrogen immediately after collection and then stored at −80 °C until RNA extraction.

We used TRIzol Reagent (Invitrogen, Carlsbad, CA, USA) to isolate total RNA following the manufacturer's instructions. The RNA purity was determined by measuring absorbance at 260 nm and 280 nm on an ND-1000 spectrophotometer (NanoDrop 2000, Thermo Fisher Scientific, Waltham, MA, USA) to calculate the A260/280 ratio; the integrity and concentration were estimated using Agilent 2100 Bioanalyzer (Agilent Technologies, Palo Alto, Calif.) with the Agilent RNA Nano Kit. In addition, RNA integrity was examined by 1% agarose gel electrophoresis. Samples with RIN (RNA integrity number) values above 7.5 and 28S/18S ≥ 1.0 were used for library construction and sequencing. The Ribo-Zero ™ Gold Kit (Illumina, San Diego, CA, USA) was used to remove rRNA from the total RNA. Subsequently, the sequencing libraries were generated following manufacturer recommendations with varied index label by NEBNext® Ultra ™ Directional RNA Library Prep Kit for Illumina (NEB, Ispawich, USA). Subsequently, the first cDNA strand was synthesized using random hexamer primers and RNA fragments as template. Second strand cDNA synthesis was subsequently performed using buffer, dNTPs, DNA polymerase I and RNase H. The library fragments were purified with QiaQuick PCR kits and elution with EB buffer, then the terminal repair, poly (A)-tailing and adapter ligation were implemented. To

select cDNA fragments of preferentially 300 bp in length, the library fragments were purified and the UNG enzyme was used to digest the second strand of cDNA. PCR was performed by aiming the expected size of amplicons, and the library was completed. The resulting 69 libraries were sequenced using the Illumina HiSeq platform with a paired-end sequencing length of 150 bp (PE150) at Annoroad Gene Technology Corporation (Beijing, PR China).

Ethics Statement

All research involving animals was conducted according to Regulations for the Administration of Affairs Concerning Experimental Animals (Ministry of Science and Technology, China, revised in March 2017), and approved by the animal ethical and welfare committee (AEWC) of Chengdu University under permit No. YXY-2021630272.

Competing Interests

The authors declare that they have no conflict of interest.

Funding

This research was funded by National Natural Science Foundation of China (81973428 to H.J., 82274046 to H.J. and 32272859 to Z.Y.), the Fundamental Research Funds of Chongqing (2022JK017) to H.J, the Beijing Nova Program (Z211100002121022 and 20230484446) to S.T.

Data availability

The clean RNA, and DNA sequencing data of muskrat and musk deer have been submitted to the NCBI (PRJNA985369 and PRJNA985371).

Authors' Contributions

Tao Wang: Writing - Original Draft, Writing - Review & Editing. **Maosen Yang:** Formal analysis, Software, Visualization. **Xin Shi:** Formal analysis, Data Curation, Visualization. **Shilin Tian:** Formal analysis, Funding acquisition, Software. **Yan Li:** Formal analysis, Methodology. **Dong Leng:** Formal analysis, Visualization. **Ming Zhang:** Data Curation, Resources. **Zheng Chengli:** Data Curation, Resources. **Chungang Feng:** Writing - Review & Editing. **Bo Zeng:** Investigation. **Xiaolan Fan:** Data Curation, Resources. **Zhao Guijun:** Supervision. **Diyan Li:** Conceptualization, Methodology, Validation, Writing - Original Draft. **Zhengrong Yuan:** Funding acquisition, Writing - Review & Editing. **Hang Jie:** Funding acquisition, Writing - Review & Editing, Resources.

References

1. He, L., et al., *Effects of crowding and sex on fecal cortisol levels of captive forest musk deer.* Biol Res, 2014. **47**(1): p. 48.
2. Mychajliw, A.M. and R.G. Harrison, *Genetics reveal the origin and timing of a cryptic insular introduction of muskrats in North America.* PLoS One, 2014. **9**(10): p. e111856.
3. Sokolov, V.E., et al., *Musk deer (Moschus moschiferus): Reinvestigation of main lipid components from preputial gland secretion.* J Chem Ecol, 1987.

13(1): p. 71-83.

4. Li, D., et al., *The musk chemical composition and microbiota of Chinese forest musk deer males.* Sci Rep, 2016. **6**: p. 18975.

5. Li, Y., et al., *Comparison of amino acid profiles and metabolic gene expression in muskrat scented glands in secretion and non-secretion season.* Sci Rep, 2017. **7**: p. 41158.

6. Shi, X., et al., *Correlation Analysis between Muskrat (Ondatra zibethicus) Musk and Traditional Musk.* Animals (Basel), 2023. **13**(10).

7. Tian, S., et al., *Comparative analyses of bat genomes identify distinct evolution of immunity in Old World fruit bats.* Sci Adv, 2023. **9**(18): p. eadd0141.

8. Chen, C., et al., *Ruminant-specific genes identified using high-quality genome data and their roles in rumen evolution.* Sci Bull (Beijing), 2022. **67**(8): p. 825-835.

9. Li, H., et al., *Chromosome-level Genome of the Muskrat (Ondatra zibethicus).* Genome Biol Evol, 2022. **14**(10).

10. Simao, F.A., et al., *BUSCO: assessing genome assembly and annotation completeness with single-copy orthologs.* Bioinformatics, 2015. **31**(19): p. 3210-2.

11. Rhie, A., et al., *Merqury: reference-free quality, completeness, and phasing assessment for genome assemblies.* Genome Biol, 2020. **21**(1): p. 245.

12. Editorial, N.B., *A reference standard for genome biology.* Nat Biotechnol, 2018. **36**(12): p. 1121.

13. De Bie, T., et al., *CAFE: a computational tool for the study of gene family evolution.* Bioinformatics, 2006. **22**(10): p. 1269-71.

14. Durand, N.C., et al., *Juicebox Provides a Visualization System for Hi-C Contact Maps with Unlimited Zoom.* Cell Syst, 2016. **3**(1): p. 99-101.

15. Clapham, D.E., *TRP channels as cellular sensors.* Nature, 2003. **426**(6966): p. 517-24.

16. Deng, Z., et al., *Gating of human TRPV3 in a lipid bilayer.* Nat Struct Mol Biol, 2020. **27**(7): p. 635-644.

17. Xu, H., et al., *Oregano, thyme and clove-derived flavors and skin sensitizers activate specific TRP channels.* Nat Neurosci, 2006. **9**(5): p. 628-35.

18. Holmes, R.S., L.A. Cox, and J.L. VandeBerg, *Comparative studies of mammalian acid lipases: Evidence for a new gene family in mouse and rat (Lipo).* Comp Biochem Physiol Part D Genomics Proteomics, 2010. **5**(3): p. 217-26.

19. Ding, J.F., et al., *IGFBP3 epigenetic promotion induced by METTL3 boosts cardiac fibroblast activation and fibrosis.* Eur J Pharmacol, 2023. **942**: p. 175494.

20. Wu, J.J., et al., *Microbiota-host crosstalk in the newborn and adult rumen at single-cell resolution.* BMC Biol, 2022. **20**(1): p. 280.

21. Fan, C., et al., *Single-Cell Transcriptome Integration Analysis Reveals the Correlation Between Mesenchymal Stromal Cells and Fibroblasts.* Front Genet, 2022. **13**: p. 798331.

22. Afzali, B. and C. Kemper, *Fibroblast tissue priming-not so nice to C you!* Immunity, 2021. **54**(5): p. 847-850.
23. Farmer, D.T., et al., *Defining epithelial cell dynamics and lineage relationships in the developing lacrimal gland.* Development, 2017. **144**(13): p. 2517-2528.
24. Park, J., et al., *Single-cell transcriptomics of the mouse kidney reveals potential cellular targets of kidney disease.* Science, 2018. **360**(6390): p. 758-763.
25. Gladka, M.M., et al., *Single-Cell Sequencing of the Healthy and Diseased Heart Reveals Cytoskeleton-Associated Protein 4 as a New Modulator of Fibroblasts Activation.* Circulation, 2018. **138**(2): p. 166-180.
26. Tusi, B.K., et al., *Population snapshots predict early haematopoietic and erythroid hierarchies.* Nature, 2018. **555**(7694): p. 54-60.
27. Xie, T., et al., *Single-Cell Deconvolution of Fibroblast Heterogeneity in Mouse Pulmonary Fibrosis.* Cell Rep, 2018. **22**(13): p. 3625-3640.
28. Castle, J.C., et al., *Immunomic, genomic and transcriptomic characterization of CT26 colorectal carcinoma.* BMC Genomics, 2014. **15**(1): p. 190.
29. Guo, M., et al., *SINCERA: A Pipeline for Single-Cell RNA-Seq Profiling Analysis.* PLoS Comput Biol, 2015. **11**(11): p. e1004575.
30. Ichikawa, T., et al., *Peroxynitrite augments fibroblast-mediated tissue remodeling via myofibroblast differentiation.* Am J Physiol Lung Cell Mol Physiol, 2008. **295**(5): p. L800-8.
31. Yeh, M.Y., et al., *Chitosan promotes immune responses, ameliorates glutamic oxaloacetic transaminase and glutamic pyruvic transaminase, but enhances lactate dehydrogenase levels in normal mice in vivo.* Exp Ther Med, 2016. **11**(4): p. 1300-1306.
32. Chueh, F.S., et al., *Crude extract of Polygonum cuspidatum promotes immune responses in leukemic mice through enhancing phagocytosis of macrophage and natural killer cell activities in vivo.* In Vivo, 2015. **29**(2): p. 255-61.
33. Chueh, F.S., et al., *Crude extract of Polygonum cuspidatum stimulates immune responses in normal mice by increasing the percentage of Mac-3-positive cells and enhancing macrophage phagocytic activity and natural killer cell cytotoxicity.* Mol Med Rep, 2015. **11**(1): p. 127-32.
34. Lin, C.C., et al., *Extract of Hedyotis diffusa Willd influences murine leukemia WEHI-3 cells in vivo as well as promoting T- and B-cell proliferation in leukemic mice.* In Vivo, 2011. **25**(4): p. 633-40.
35. Plasschaert, L.W., et al., *A single-cell atlas of the airway epithelium reveals the CFTR-rich pulmonary ionocyte.* Nature, 2018. **560**(7718): p. 377-381.
36. Sharifiaghdas, F., et al., *Comparing supportive properties of poly lactic-co-glycolic acid (PLGA), PLGA/collagen and human amniotic membrane for human urothelial and smooth muscle cells engineering.* Urol J, 2014. **11**(3): p. 1620-8.
37. Demir, I.E., et al., *Investigation of Schwann cells at neoplastic cell sites*

before the onset of cancer invasion. J Natl Cancer Inst, 2014. **106**(8).

38. Saiki, T., et al., *The Effects of Insulin on Immortalized Rat Schwann Cells, IFRS1.* Int J Mol Sci, 2021. **22**(11).
39. Deborde, S., et al., *Schwann cells induce cancer cell dispersion and invasion.* J Clin Invest, 2016. **126**(4): p. 1538-54.
40. Dezawa, M. and E. Adachi-Usami, *Role of Schwann cells in retinal ganglion cell axon regeneration.* Prog Retin Eye Res, 2000. **19**(2): p. 171-204.
41. Azam, S.H. and C.V. Pecot, *Cancer's got nerve: Schwann cells drive perineural invasion.* J Clin Invest, 2016. **126**(4): p. 1242-4.
42. Han, Y., et al., *Coordinate control of basal epithelial cell fate and stem cell maintenance by core EMT transcription factor Zeb1.* Cell Rep, 2022. **38**(2): p. 110240.
43. Lixa, C., et al., *Retinoic Acid Binding Leads to CRABP2 Rigidification and Dimerization.* Biochemistry, 2019. **58**(41): p. 4183-4194.
44. Casteels, M., et al., *The role of 2-hydroxyacyl-CoA lyase, a thiamin pyrophosphate-dependent enzyme, in the peroxisomal metabolism of 3-methyl-branched fatty acids and 2-hydroxy straight-chain fatty acids.* Biochem Soc Trans, 2007. **35**(Pt 5): p. 876-80.
45. Itkonen, H.M., et al., *Lipid degradation promotes prostate cancer cell survival.* Oncotarget, 2017. **8**(24): p. 38264-38275.
46. Piórkowska, K., et al., *Evolution of peroxisomal trans-2-enoyl-CoA reductase (PECR) as candidate gene for meat quality.* Livestock Science, 2017. **201**: p. 85-91.
47. Zhang, M., et al., *Regulatory Roles of Peroxisomal Metabolic Pathways Involved in Musk Secretion in Muskrats.* J Membr Biol, 2019. **252**(1): p. 61-75.
48. Lieberman-Aiden, E., et al., *Comprehensive mapping of long-range interactions reveals folding principles of the human genome.* Science, 2009. **326**(5950): p. 289-93.
49. Schoenfelder, S. and P. Fraser, *Long-range enhancer-promoter contacts in gene expression control.* Nat Rev Genet, 2019. **20**(8): p. 437-455.
50. Zhang, R., B.S. Tang, and J.F. Guo, *Research advances on neurite outgrowth inhibitor B receptor.* J Cell Mol Med, 2020. **24**(14): p. 7697-7705.
51. Sun, Y., et al., *The emerging role of NPNT in tissue injury repair and bone homeostasis.* J Cell Physiol, 2018. **233**(3): p. 1887-1894.
52. Kruse, K., et al., *TADtool: visual parameter identification for TAD-calling algorithms.* Bioinformatics, 2016. **32**(20): p. 3190-3192.
53. Li, D., et al., *Dynamic transcriptome and chromatin architecture in granulosa cells during chicken folliculogenesis.* Nature Communications, 2022. **13**(1): p. 131.
54. Ron, G., et al., *Promoter-enhancer interactions identified from Hi-C data using probabilistic models and hierarchical topological domains.* Nat Commun, 2017. **8**(1): p. 2237.
55. Schoenfelder, S., et al., *The pluripotent regulatory circuitry connecting promoters to their long-range interacting elements.* Genome Res, 2015. **25**(4):

p. 582-97.
56. Sanyal, A., et al., *The long-range interaction landscape of gene promoters.* Nature, 2012. **489**(7414): p. 109-13.
57. Kai, F., J.P. Fawcett, and R. Duncan, *Synaptopodin-2 induces assembly of peripheral actin bundles and immature focal adhesions to promote lamellipodia formation and prostate cancer cell migration.* Oncotarget, 2015. **6**(13): p. 11162-74.
58. Kosaka, T., et al., *Prognostic implication of EGFR, KRAS, and TP53 gene mutations in a large cohort of Japanese patients with surgically treated lung adenocarcinoma.* J Thorac Oncol, 2009. **4**(1): p. 22-9.
59. Unruh, D. and C. Horbinski, *Beyond thrombosis: the impact of tissue factor signaling in cancer.* J Hematol Oncol, 2020. **13**(1): p. 93.
60. MacNeill, S.A., *Structure and function of the GINS complex, a key component of the eukaryotic replisome.* Biochem J, 2010. **425**(3): p. 489-500.
61. Bowen, S., et al., *Mutations in the CYLD gene in Brooke-Spiegler syndrome, familial cylindromatosis, and multiple familial trichoepithelioma: lack of genotype-phenotype correlation.* J Invest Dermatol, 2005. **124**(5): p. 919-20.
62. Anttonen, A.K., et al., *Novel SIL1 mutations and exclusion of functional candidate genes in Marinesco-Sjogren syndrome.* Eur J Hum Genet, 2008. **16**(8): p. 961-9.
63. Pearlman, A., et al., *Mutations in MAP3K1 cause 46,XY disorders of sex development and implicate a common signal transduction pathway in human testis determination.* Am J Hum Genet, 2010. **87**(6): p. 898-904.
64. Nardi, F., et al., *Proteasomal modulation of cellular SNAT2 (SLC38A2) abundance and function by unsaturated fatty acid availability.* J Biol Chem, 2015. **290**(13): p. 8173-84.
65. Yang, Z., *PAML 4: phylogenetic analysis by maximum likelihood.* Mol Biol Evol, 2007. **24**(8): p. 1586-91.
66. Zhang, J. and S. Kumar, *Detection of convergent and parallel evolution at the amino acid sequence level.* Mol Biol Evol, 1997. **14**(5): p. 527-36.
67. Nummela, S., et al., *Exploring the mammalian sensory space: co-operations and trade-offs among senses.* J Comp Physiol A Neuroethol Sens Neural Behav Physiol, 2013. **199**(12): p. 1077-92.
68. Liu, C., et al., *A towering genome: Experimentally validated adaptations to high blood pressure and extreme stature in the giraffe.* Sci Adv, 2021. **7**(12).
69. Yang, F., et al., *TEX15 associates with MILI and silences transposable elements in male germ cells.* Genes Dev, 2020. **34**(11-12): p. 745-750.
70. Chan, J.Y., et al., *Whole exome sequencing identifies recessive germline mutations in FAM160A1 in familial NK/T cell lymphoma.* Blood Cancer Journal, 2018. **8**(11): p. 111.
71. Luo, Q., et al., *Role of ACSL5 in fatty acid metabolism.* Heliyon, 2023. **9**(2): p. e13316.
72. Polinski, N.K., et al., *Decreased glucocerebrosidase activity and substrate accumulation of glycosphingolipids in a novel GBA1 D409V knock-in mouse model.*

PLoS One, 2021. **16**(6): p. e0252325.

73. Hirai, K., et al., *Molecular and Functional Analysis of Choline Transporters and Antitumor Effects of Choline Transporter-Like Protein 1 Inhibitors in Human Pancreatic Cancer Cells.* Int J Mol Sci, 2020. **21**(15).

74. Tong, H.L., et al., *MiR-2425-5p targets RAD9A and MYOG to regulate the proliferation and differentiation of bovine skeletal muscle-derived satellite cells.* Sci Rep, 2017. **7**(1): p. 418.

75. Chen, J., et al., *Genome-wide identification of potential odontogenic genes involved in the dental epithelium-mesenchymal interaction during early odontogenesis.* BMC Genomics, 2023. **24**(1): p. 163.

76. Karihaloo, A., et al., *Hepatocyte growth factor-mediated renal epithelial branching morphogenesis is regulated by glypican-4 expression.* Mol Cell Biol, 2004. **24**(19): p. 8745-52.

77. Ho, C.M., et al., *Collagen type VI regulates the CDK4/6-p-Rb signaling pathway and promotes ovarian cancer invasiveness, stemness, and metastasis.* Am J Cancer Res, 2021. **11**(3): p. 668-690.

78. Donker, L., et al., *A mechanical G2 checkpoint controls epithelial cell division through E-cadherin-mediated regulation of Wee1-Cdk1.* Cell Rep, 2022. **41**(2): p. 111475.

79. Arai, C., et al., *Nephronectin plays critical roles in Sox2 expression and proliferation in dental epithelial stem cells via EGF-like repeat domains.* Sci Rep, 2017. **7**: p. 45181.

80. Al Kaabi, E.H. and A.W. El-Hattab, *N-acetylglutamate synthase deficiency: Novel mutation associated with neonatal presentation and literature review of molecular and phenotypic spectra.* Mol Genet Metab Rep, 2016. **8**: p. 94-8.

81. Floriot, S., et al., *CEP250 is Required for Maintaining Centrosome Cohesion in the Germline and Fertility in Male Mice.* Front Cell Dev Biol, 2021. **9**: p. 754054.

82. Li, Z.G., M.Y. Wu, and H.T. Jia, *[Research Progress on Expression Regulation, Function and Clinical Significance of CASP8AP2 Gene].* Zhongguo Shi Yan Xue Ye Xue Za Zhi, 2015. **23**(2): p. 557-61.

83. Hu, J., et al., *NextPolish: a fast and efficient genome polishing tool for long-read assembly.* Bioinformatics, 2020. **36**(7): p. 2253-2255.

84. Langmead, B. and S.L. Salzberg, *Fast gapped-read alignment with Bowtie 2.* Nat Methods, 2012. **9**(4): p. 357-9.

85. Wingett, S., et al., *HiCUP: pipeline for mapping and processing Hi-C data.* F1000Res, 2015. **4**: p. 1310.

86. Li, H., *Minimap2: pairwise alignment for nucleotide sequences.* Bioinformatics, 2018. **34**(18): p. 3094-3100.

87. Myers, E.W., *The fragment assembly string graph.* Bioinformatics, 2005. **21 Suppl 2**: p. ii79-85.

88. Zhang, X., et al., *Assembly of allele-aware, chromosomal-scale autopolyploid genomes based on Hi-C data.* Nat Plants, 2019. **5**(8): p. 833-845.

89. Li, H. and R. Durbin, *Fast and accurate long-read alignment with Burrows-*

Wheeler transform. Bioinformatics, 2010. **26**(5): p. 589-95.
90. Bergman, C.M. and H. Quesneville, *Discovering and detecting transposable elements in genome sequences.* Brief Bioinform, 2007. **8**(6): p. 382-92.
91. Edgar, R.C. and E.W. Myers, *PILER: identification and classification of genomic repeats.* Bioinformatics, 2005. **21 Suppl 1**: p. i152-8.
92. Xu, Z. and H. Wang, *LTR_FINDER: an efficient tool for the prediction of full-length LTR retrotransposons.* Nucleic Acids Res, 2007. **35**(Web Server issue): p. W265-8.
93. Price, A.L., N.C. Jones, and P.A. Pevzner, *De novo identification of repeat families in large genomes.* Bioinformatics, 2005. **21 Suppl 1**: p. i351-8.
94. Benson, G., *Tandem repeats finder: a program to analyze DNA sequences.* Nucleic Acids Res, 1999. **27**(2): p. 573-80.
95. Mount, D.W., *Using the Basic Local Alignment Search Tool (BLAST).* CSH Protoc, 2007. **2007**: p. pdb top17.
96. Birney, E., M. Clamp, and R. Durbin, *GeneWise and Genomewise.* Genome Res, 2004. **14**(5): p. 988-95.
97. Kim, D., et al., *TopHat2: accurate alignment of transcriptomes in the presence of insertions, deletions and gene fusions.* Genome Biol, 2013. **14**(4): p. R36.
98. Trapnell, C., et al., *Differential gene and transcript expression analysis of RNA-seq experiments with TopHat and Cufflinks.* Nat Protoc, 2012. **7**(3): p. 562-78.
99. Haas, B.J., et al., *Improving the Arabidopsis genome annotation using maximal transcript alignment assemblies.* Nucleic acids research, 2003. **31**(19): p. 5654-5666.
100. Stanke, M. and S. Waack, *Gene prediction with a hidden Markov model and a new intron submodel.* Bioinformatics, 2003. **19 Suppl 2**: p. ii215-25.
101. Korf, I., *Gene finding in novel genomes.* BMC Bioinformatics, 2004. **5**: p. 59.
102. Majoros, W.H., M. Pertea, and S.L. Salzberg, *TigrScan and GlimmerHMM: two open source ab initio eukaryotic gene-finders.* Bioinformatics, 2004. **20**(16): p. 2878-9.
103. Guigo, R., *Assembling genes from predicted exons in linear time with dynamic programming.* J Comput Biol, 1998. **5**(4): p. 681-702.
104. Burge, C. and S. Karlin, *Prediction of complete gene structures in human genomic DNA.* J Mol Biol, 1997. **268**(1): p. 78-94.
105. UniProt Consortium, T., *UniProt: the universal protein knowledgebase.* Nucleic Acids Res, 2018. **46**(5): p. 2699.
106. Kanehisa, M., et al., *Data, information, knowledge and principle: back to metabolism in KEGG.* Nucleic Acids Res, 2014. **42**(Database issue): p. D199-205.
107. Wang, Y., et al., *MCScanX: a toolkit for detection and evolutionary analysis of gene synteny and collinearity.* Nucleic Acids Res, 2012. **40**(7): p. e49.
108. Veidenberg, A., A. Medlar, and A. Loytynoja, *Wasabi: An Integrated Platform for Evolutionary Sequence Analysis and Data Visualization.* Mol Biol Evol, 2016. **33**(4): p. 1126-30.
109. Talavera, G. and J. Castresana, *Improvement of phylogenies after removing*

divergent and ambiguously aligned blocks from protein sequence alignments. Syst Biol, 2007. **56**(4): p. 564-77.

110. Posada, D. and K.A. Crandall, *MODELTEST: testing the model of DNA substitution.* Bioinformatics, 1998. **14**(9): p. 817-8.
111. Stamatakis, A., *RAxML version 8: a tool for phylogenetic analysis and post-analysis of large phylogenies.* Bioinformatics, 2014. **30**(9): p. 1312-3.
112. Li, L., C.J. Stoeckert, Jr., and D.S. Roos, *OrthoMCL: identification of ortholog groups for eukaryotic genomes.* Genome Res, 2003. **13**(9): p. 2178-89.
113. Xie, C., et al., *KOBAS 2.0: a web server for annotation and identification of enriched pathways and diseases.* Nucleic Acids Res, 2011. **39**(Web Server issue): p. W316-22.
114. Mao, X., et al., *Automated genome annotation and pathway identification using the KEGG Orthology (KO) as a controlled vocabulary.* Bioinformatics, 2005. **21**(19): p. 3787-3793.
115. Yang, J., et al., *The I-TASSER Suite: protein structure and function prediction.* Nat Methods, 2015. **12**(1): p. 7-8.
116. Yanai, I., et al., *Genome-wide midrange transcription profiles reveal expression level relationships in human tissue specification.* Bioinformatics, 2005. **21**(5): p. 650-9.
117. Robinson, M.D., D.J. McCarthy, and G.K. Smyth, *edgeR: a Bioconductor package for differential expression analysis of digital gene expression data.* Bioinformatics, 2010. **26**(1): p. 139-40.
118. Chen, S., et al., *fastp: an ultra-fast all-in-one FASTQ preprocessor.* Bioinformatics, 2018. **34**(17): p. i884-i890.
119. Zheng, G.X., et al., *Massively parallel digital transcriptional profiling of single cells.* Nat Commun, 2017. **8**: p. 14049.
120. Butler, A., et al., *Integrating single-cell transcriptomic data across different conditions, technologies, and species.* Nat Biotechnol, 2018. **36**(5): p. 411-420.
121. Durand, N.C., et al., *Juicer Provides a One-Click System for Analyzing Loop-Resolution Hi-C Experiments.* Cell Syst, 2016. **3**(1): p. 95-8.
122. Battulin, N., et al., *Comparison of the 3D organization of sperm and fibroblast genomes using the Hi-C approach.* Genome Biol, 2015. **16**(1): p. 77.

Figure 1

Click here to access/download;Figure;Figure1.pdf

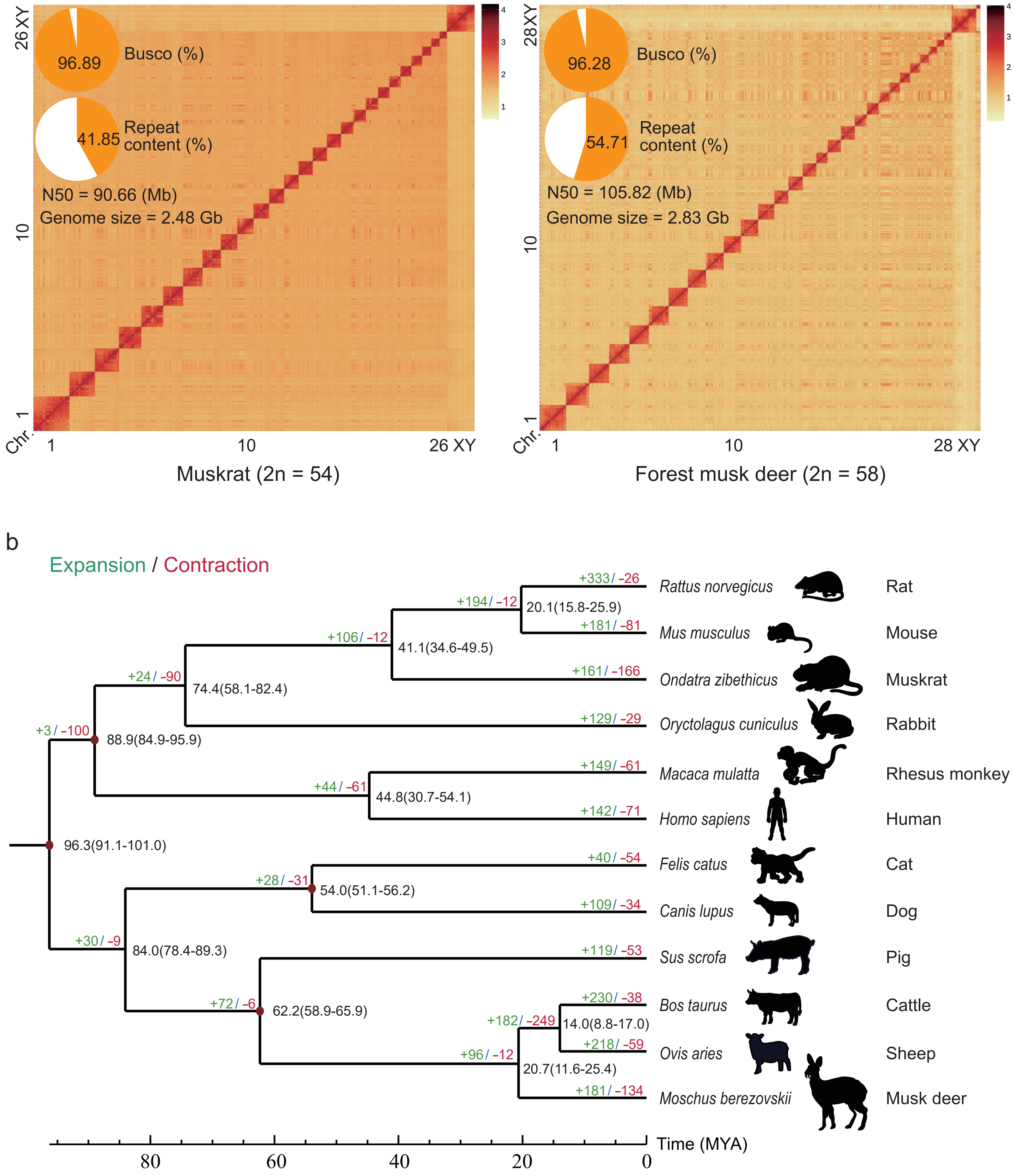

Figure 2

Click here to access/download;Figure;Figure2.pdf

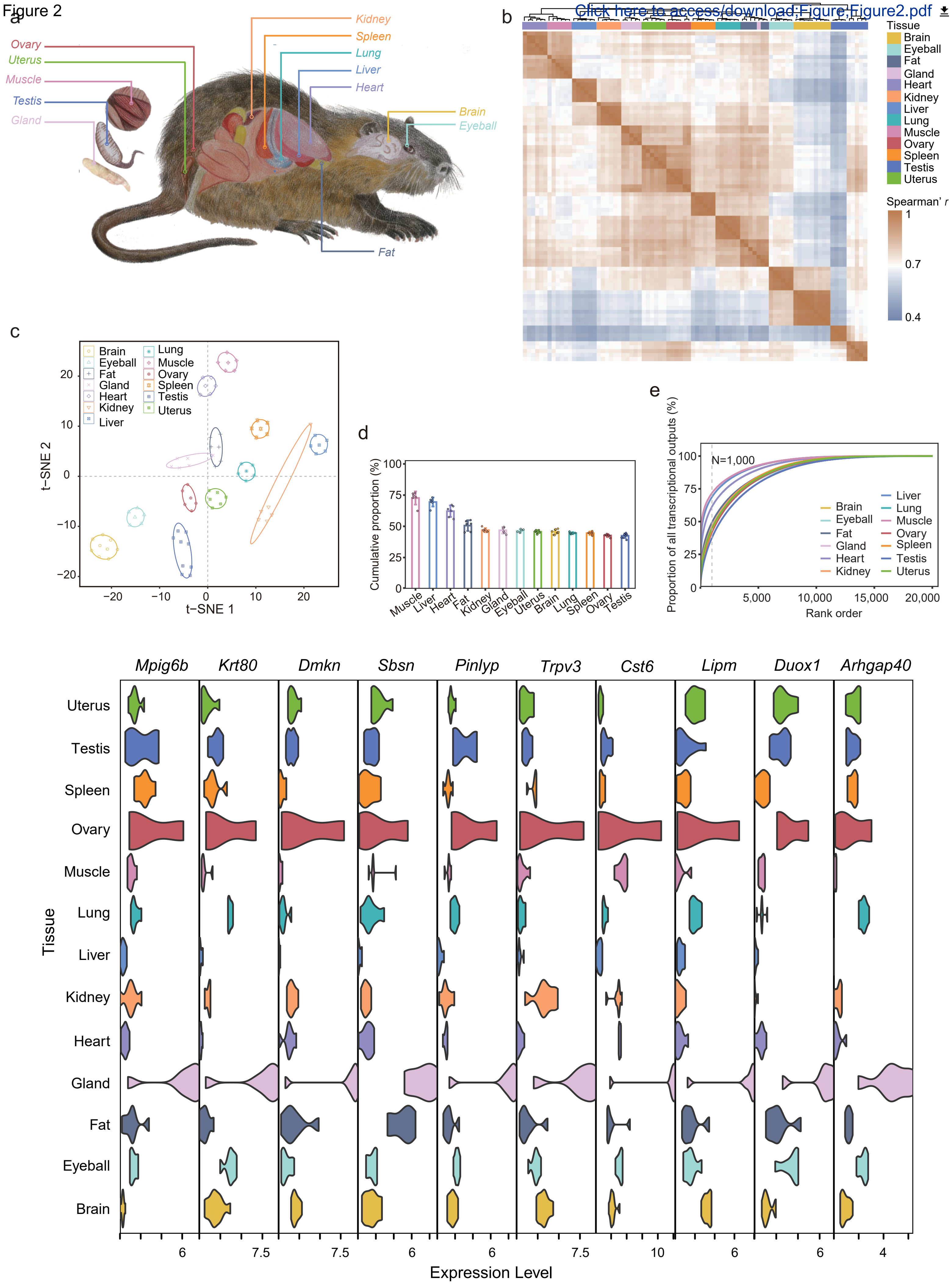

Figure 3

Click here to access/download;Figure;Figure3.pdf

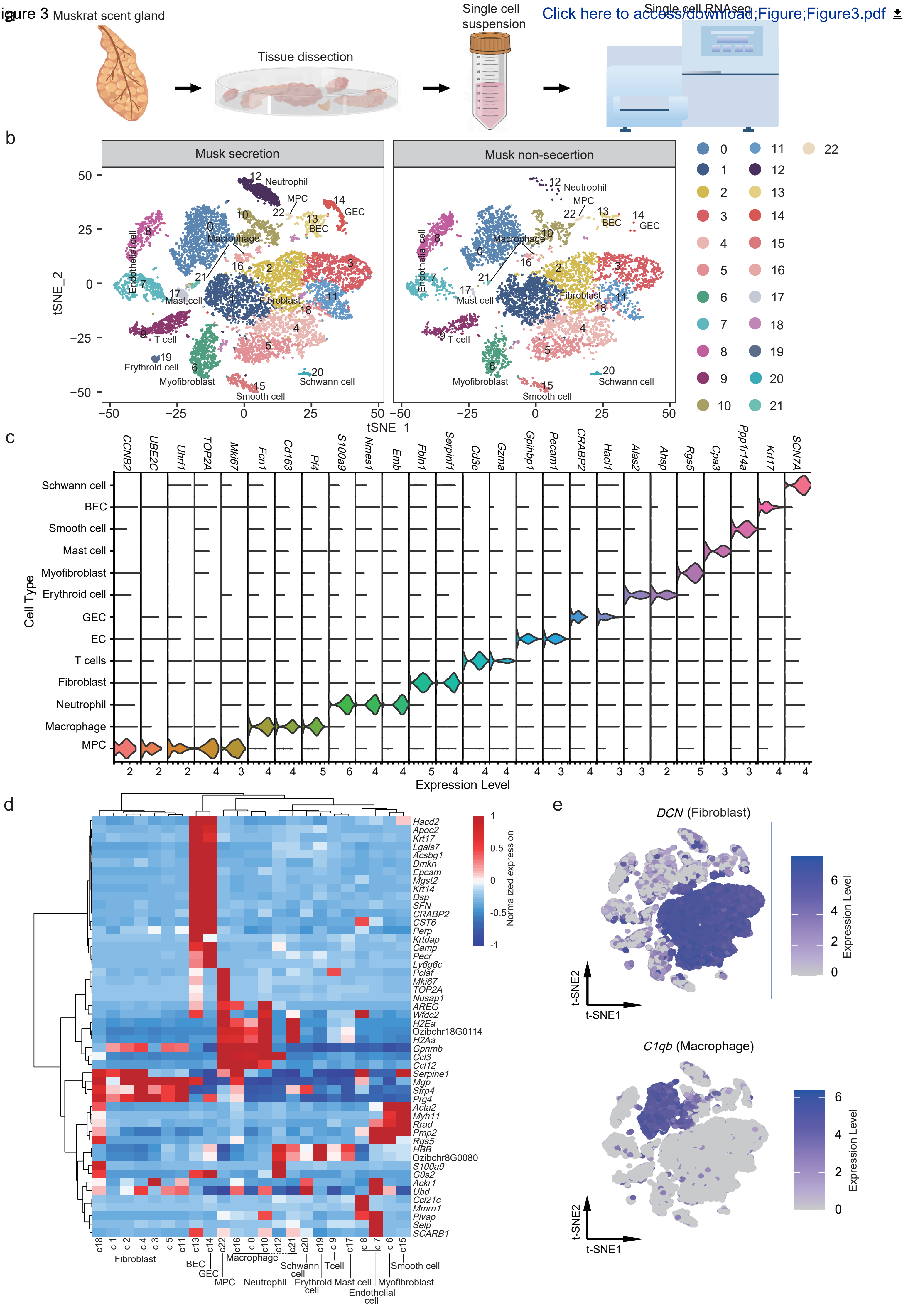

Figure 4

Click here to access/download;Figure;Figure4.pdf

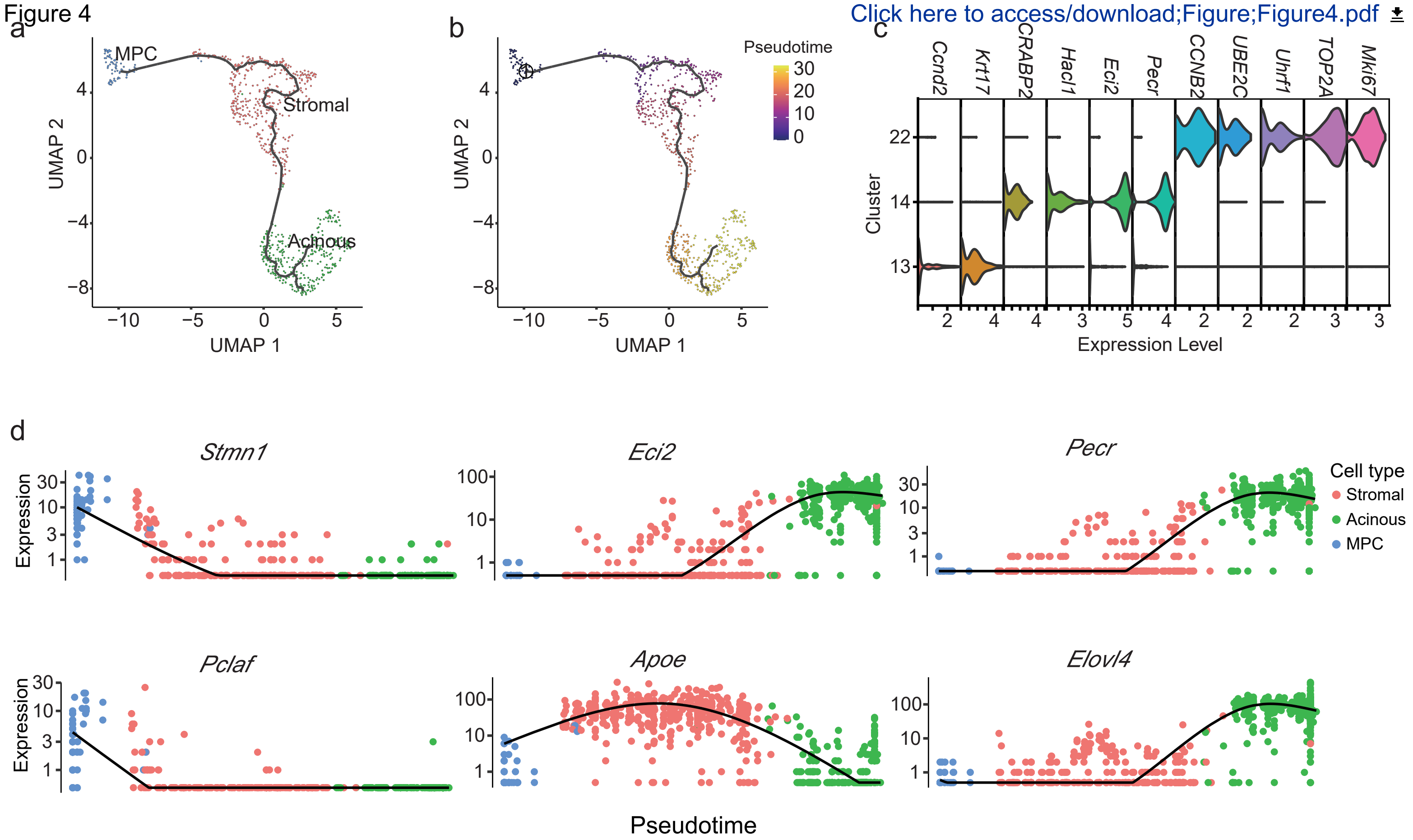

Figure 5

Click here to access/download;Figure;Figure5.pdf

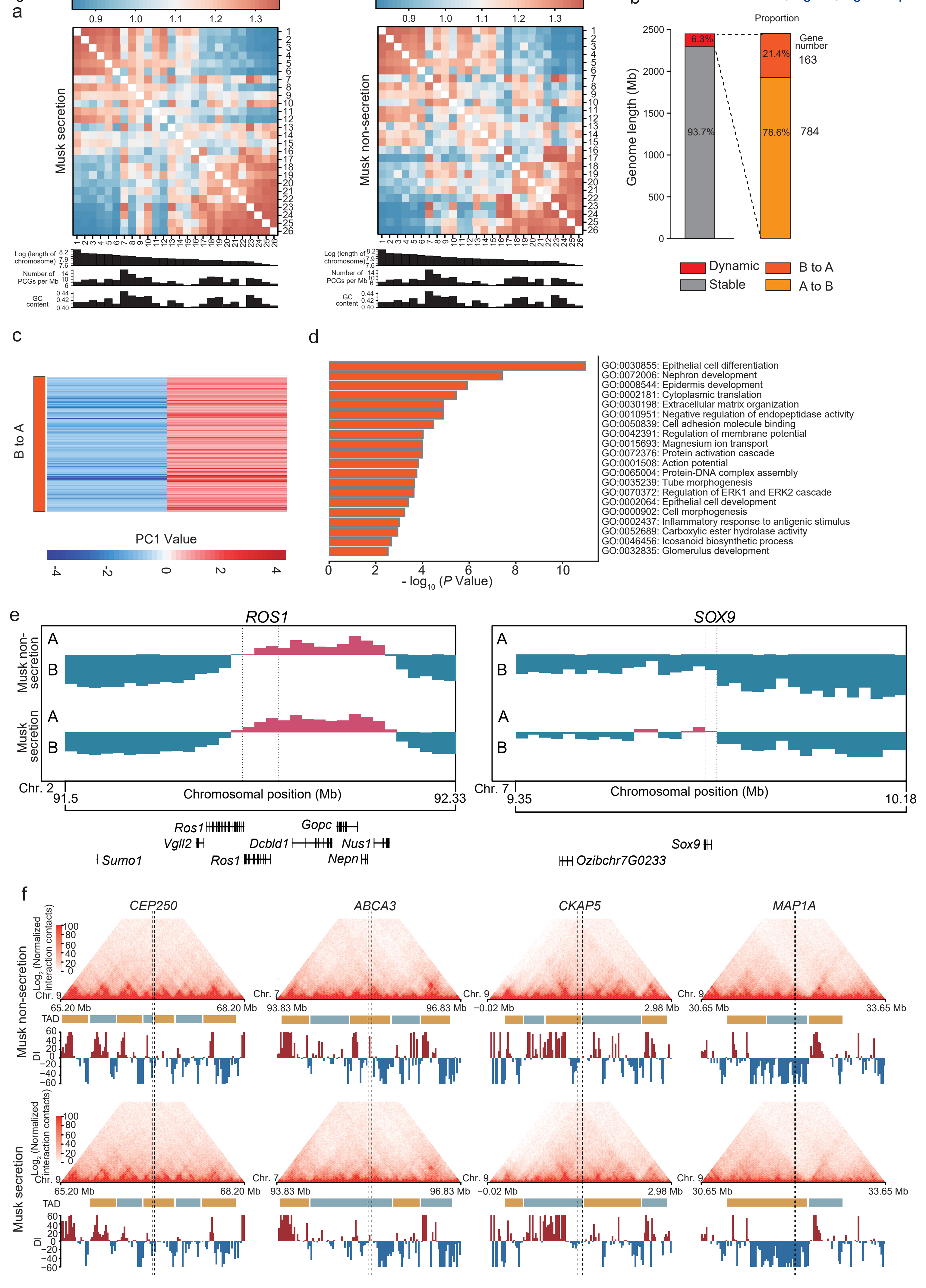

Figure 6

Click here to access/download;Figure;Figure6.pdf

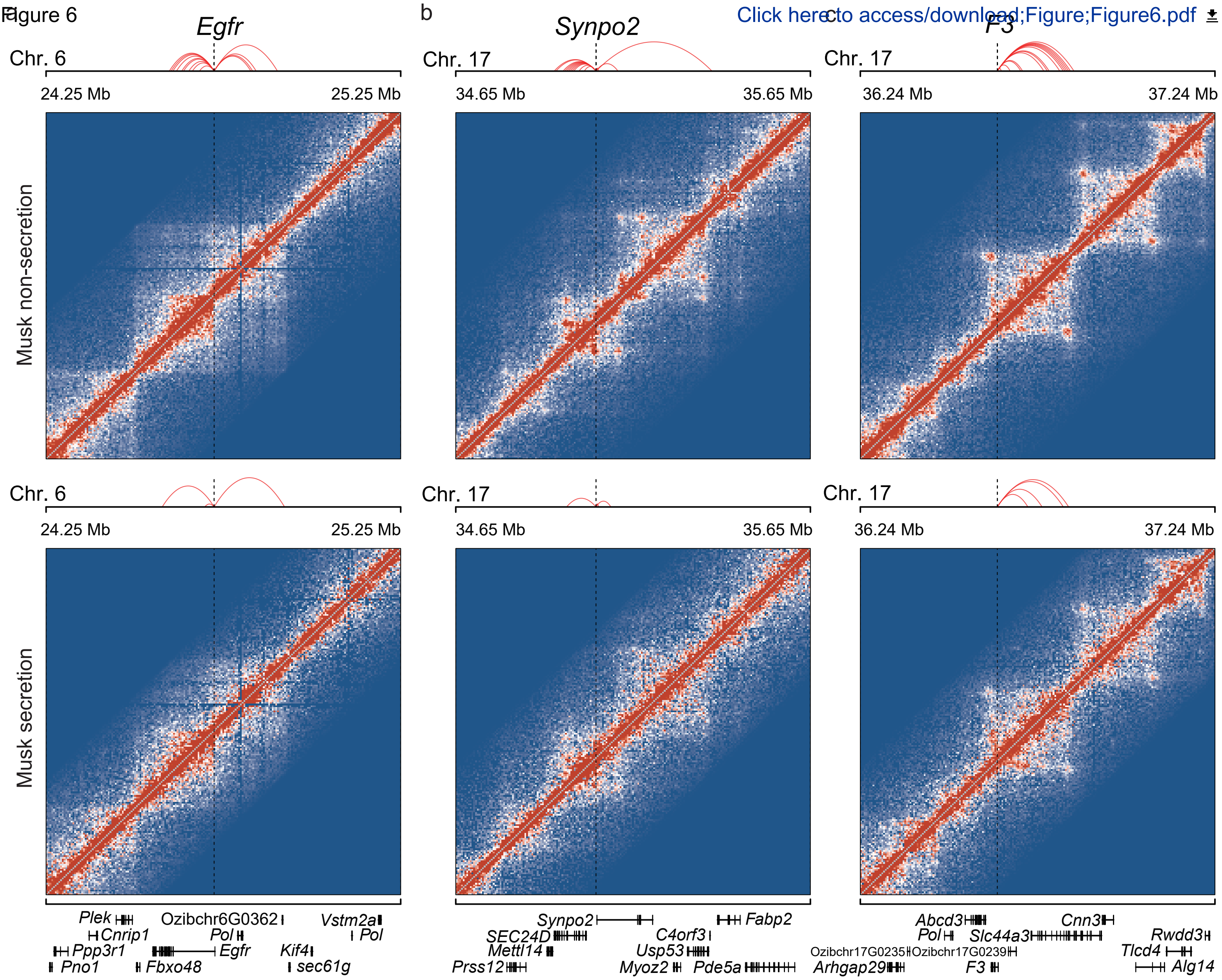

Click here to access/download;Figure;Figure7.pdf

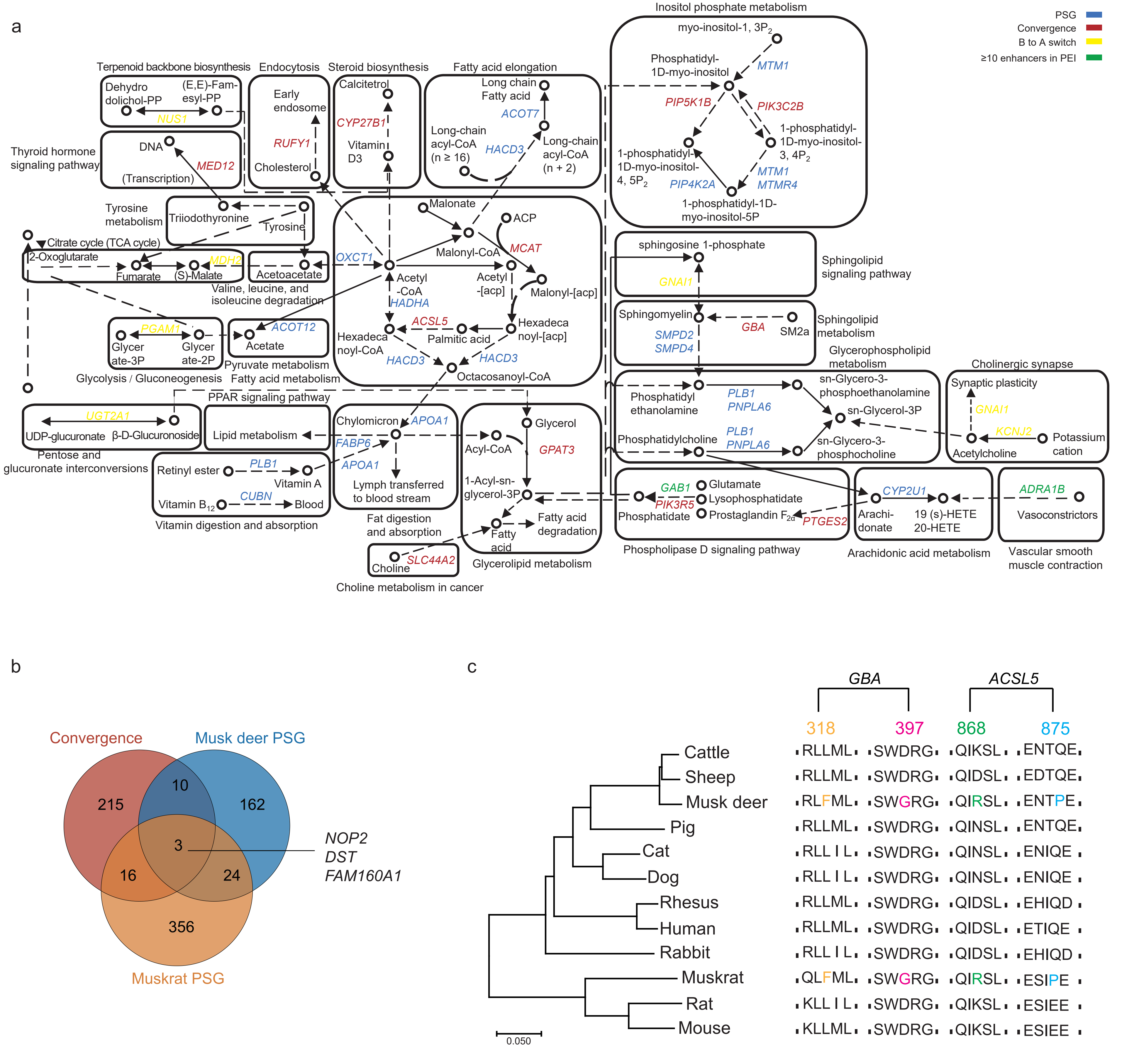

Figure 8

Click here to access/download;Figure;Figure8.pdf

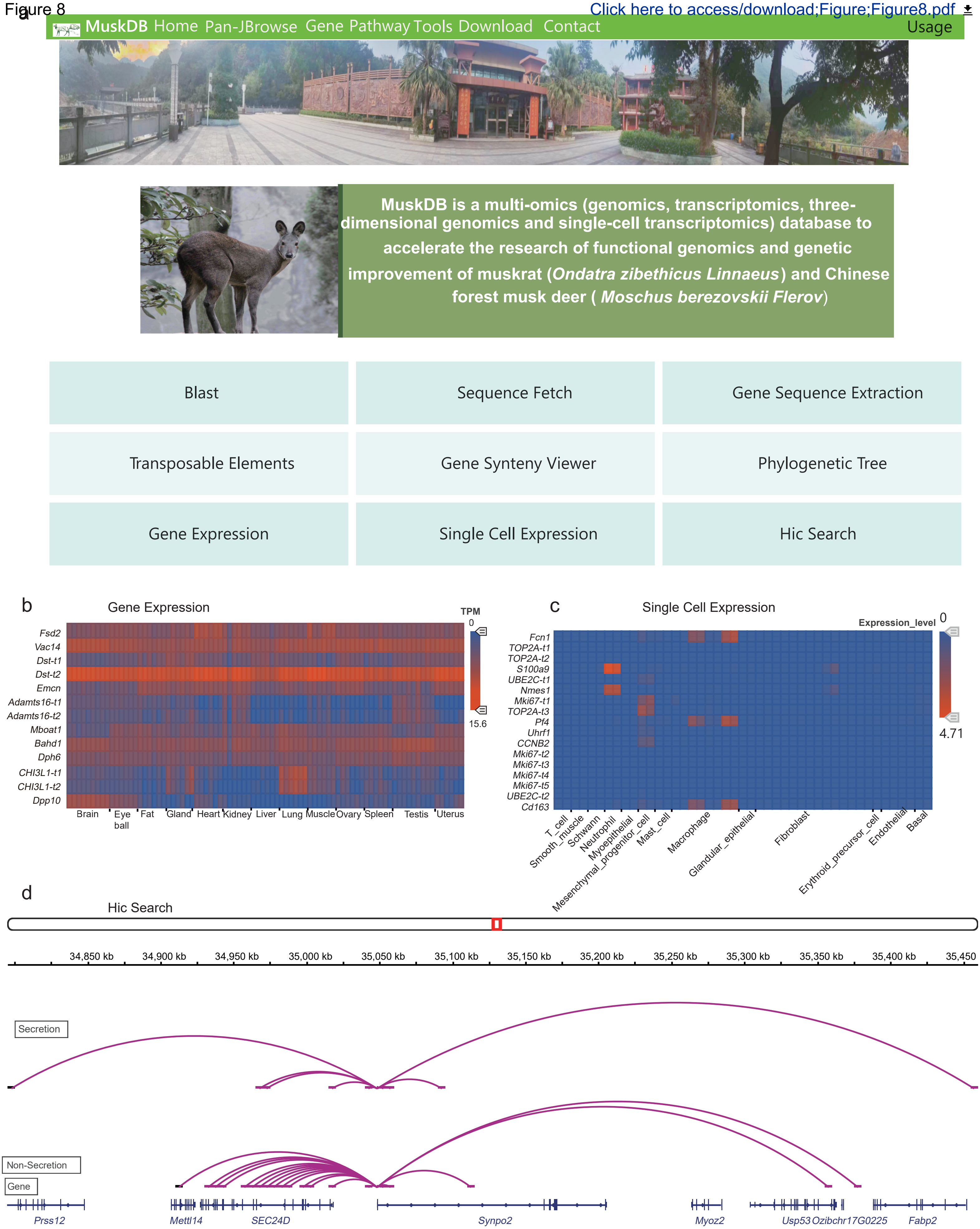

Table S1 to S12

Click here to access/download
Supplementary Material
Additional file1.xlsx

Supplementary Figure 1 to 8

Click here to access/download
Supplementary Material
Surpporting information.docx
