# Supplementary material for: Multiomics analysis provides insights into musk secretion in muskrat and musk deer
Source: Gigascience. 2025 Feb 26;14:giaf006. doi: 10.1093/gigascience/giaf006 (PMC11878540; doi:10.1093/gigascience/giaf006)
Supplement: giaf006_GIGA-D-24-00205_Revision_1 [file giaf006_giga-d-24-00205_revision_1.pdf]

GigaScience

Multi-omics analyses identify distinct patterns of selection in musk secretion animals

--Manuscript Draft--

**Manuscript Number:**	GIGA-D-24-00205R1
**Full Title:**	Multi-omics analyses identify distinct patterns of selection in musk secretion animals
**Article Type:**	Research
**Funding Information:**	National Natural Science Foundation of China (32272859) — Mr. Zhengrong Yuan<br>National Natural Science Foundation of China (81973428) — Dr Hang Jie<br>National Natural Science Foundation of China (82274046) — Dr Hang Jie<br>the Fundamental Research Funds of Chongqing (2022JK017) — Dr Hang Jie<br>the Beijing Nova Program (Z211100002121022，20230484446) — Mr. Shilin Tian
**Abstract:**	Background<br>Musk, secreted by the musk gland of adult male musk-secreting mammals, holds significant pharmaceutical and cosmetic potential. However, understanding the molecular mechanisms of musk secretion remain limited, largely due to the lack of comprehensive multi-omics analyses and available platforms for relevant species, such as muskrats (Ondatra zibethicus Linnaeus) and Chinese forest musk deer (Moschus berezovskii Flerov).<br>Results<br>We generated chromosome-level genomes for both species (Ondatra zibethicus Linnaeus and Moschus berezovskii Flerov) along with 168 muskrat transcriptomes. Comparative analysis with eleven other vertebrate genomes revealed genes and amino acid sites with signs of adaptive convergent evolution, primarily linked to lipid metabolism, cell cycle regulation, protein binding, and immunity. Single-cell RNA sequencing and Hi-C analyses demonstrated enhanced gene expression during muskrat musk secretion, particularly in biological processes related to “regulation of secretion.” Additionally, we developed MuskDB (http://117.78.45.2:1087/home), a freely accessible multi-omics database platform for musk-secreting mammals.<br>Conclusions<br>Genes such as SMPDL3A and NRCAM exhibited specific PEIs and compartment transitions, suggesting a role in musk secretion. The study concludes that the evolution of musk secretion in muskrats and musk deer is likely driven by lipid metabolism and cell specialization. This underscores the complexity of the musk gland and calls for further investigation into musk secretion-specific genetic variants.
**Corresponding Author:**	Diyan Li<br>Chengdu University<br>Chengdu, CHINA
**Corresponding Author Secondary Information:**	
**Corresponding Author's Institution:**	Chengdu University
**Corresponding Author's Secondary Institution:**	
**First Author:**	Tao Wang
**First Author Secondary Information:**	
**Order of Authors:**	Tao Wang
	Maosen Yang

Powered by Editorial Manager® and ProduXion Manager® from Aries Systems Corporation

	Xin Shi
	Shilin Tian
	Yan Li
	Wenqian Xie
	Zhengting Zou
	Dong Leng
	Ming Zhang
	Chengli Zheng
	Chungang Feng
	Bo Zeng
	Xiaolan Fan
	Huimin Qiu
	Jing Li
	Guijun Zhao
	Diyan Li
	Zhengrong Yuan
	Hang Jie
**Order of Authors Secondary Information:**	

Response to Reviewers:

Dear editor,
We sincerely thank you for allowing us to revise our manuscript entitled 'Multi-omics analyses identify distinct patterns of selection in musk secretion animals' (original manuscript No. GIGA-D-24-00205) with substantial improvements and resolve all the concerns raised by the reviewers. 1) We revised our manuscript carefully, reanalyzed the data, especially the convergent evolution genes; 2) 'Better Papers Faster' was asked for our originally submitted and the current revised manuscripts for editing service to check for grammar, and polish the writing; 3) all figures are recognized.
Below we provide our point-to-point responses, and hope that you and the reviewers are satisfied many thanks for your consideration of our manuscript for possible publication in GigaScience.
We look forward to hearing a positive response from you.

Best regards,
Zhengrong Yuan, Diyan Li and Hang Jie

Detailed responses to reviewers
All comments provided by reviewers are in gray italics, and our responses are in black.
Important revisions in the manuscript are marked in red.

Reviewer 1
Comment 1-1:
Wang et al. utilized multi-omics data to investigate the mechanism underlying musk secreting, by sequencing the genomes of two musk-secreting mammals (muskrat and musk deer), 188 RNA-seq libraries of muskrat organs, scRNA-seq, and HiC data. The size of dataset generated here is large and such omics data would provide great resources for related studies. However, I have several concerns.
Major:
1.The data presented in this study is impressive, but no strong connections among different data analysis sections (genome assembly, bulk RNA-seq analysis, scRNA-seq, HiC and selection analysis). The authors should carefully re-organize and connect these sections by logic. Five figures are recommended.
Suggestions on section organization: PSG and REG analysis should be moved after genome assembly part. The PEI (Line373) and compartment/TAD analysis (Line296) should be merged as one section.

Response 1-1:
Thanks for your positive and helpful comments.
(1)As suggested, only five figures were displayed in our revised manuscript.
(2)PSG and REG analysis was moved after genome assembly part.
(3)The PEI and compartment/TAD analysis were merged as one section.

Comment 1-2:
This study also sequenced 84 small RNA-seq libraries (line 78/line 145). But no analysis about small RNA was found in this manuscript. The small RNA-seq is miRNA-seq (Line 720)? I also noticed the sequencing of lncRNA-seq (Line 718). Too confusing.
Response 1-2:
Thanks for the comments, yes, we also sequenced 84 small RNA-seq libraries (it is miRNA). We only did basic analysis of miRNA (Figure S4b), including mapping, expression and t-SNE clustering analysis. But we did not do differential expression and function enrichment analysis among different tissues. For lncRNA-seq libraries, we mostly focused on analysis the expression profile of mRNAs. The lncRNA analysis was also similar to miRNA, only including mapping, expression and t-SNE clustering analysis (Figure S4a).

Figure S4. Gene expression from 13 tissues in muskrat. (a) t - distributed stochastic neighbor embedding (t-SNE) clustering of samples using lncRNA (left panel) and miRNA (right panel) expression. (b) The lncRNAs (left panel) and miRNAs (right panel) specifically expressed in musk gland. (c) The proportion of expressed genes of each sample. Each dot represents a sample in every tissue. (d) Abundance distribution of transcripts across tissues. The x-axis indicates the proportion of transcripts sorted from highest to lowest abundance, with the vertical dashed line indicating the top 1,000 of highest abundance transcripts. The y-axis indicates the accumulated fraction of transcripts relative to the total transcripts. Colored lines represent mean values across tissues. (e) Heatmap of DEGs (differentially expressed genes) numbers in pairwise comparisons among the 13 tissues. DEGs were identified using the threshold of |log2fold change| > 1 and corrected ($P < 0.01$).

Comment 1-3:
The material&method part was poorly organized and written and I can not follow. For example, in the part named "Expression analysis of mRNA, lncRNA and miRNA"(Line863), the contents were the experimental details of RNA-seq with no expression analysis methods. Also this experimental details repeated the previous contents (Line704-724) but with inconsistent description.
Response 1-3:
Thanks for your constructive comments. Sorry for the repeated “Expression analysis” methods. We deleted it and carefully revised the expression analysis method to the subsection of “Muskrat transcriptome reconstruction”.

Comment 1-4:
Many typos. The authors should check and revise carefully.
Response 1-4:
Thanks for the helpful comments. Sorry for our carelessness. In our revised manuscript, we have checked and revised carefully.

Comment 1-5:
Line94-96 The sequencing depth and quality should be mentioned here. Also only OZ sequencing data information was found in Table S1.
Response 1-5:
Thanks for your suggestions. We describe the sequencing depth of the two musk-secreting mammals (muskrat and musk deer): briefly, muskrat and forest musk deer had 235.25-fold and 246.02-fold sequencing depths, respectively. The quality sequencing is also shown in Table S1.

Comment 1-6:
Line 122-125 Simplify the statement, especially the use of software (CAFE, OrthoFinder..) which should be in Method.
Response 1-6:
We are sincerely grateful for your comments. We Simplify the statement, especially the

use of software, as they are already described in the Methods. We also checked the results section for similar errors and made the same modifications, with the modification process marked in red.

Comment 1-7:
What function modules are the expanded or contracted gene families enriched in ? Or the numbers here mean nothing.
Response 1-7:
Thanks for the constructive comments. Enrichment analysis of expanded and contracted genes in the two musk-secreting mammals were performed using KEGG pathway, Reactome and Go ontology. The Top 10 enrichment pathways are shown in Fig S2a and the original results of enrichment analysis are shown in Table S7-S8.
GO enrichment analyses of contracted genes showed that these two species were both involved in pathways like “Olfactory Signaling Pathway”, “Olfactory transduction”, “Signaling by GPCR”, “B cell receptor signaling pathway”, “Tight junction Immunoregulatory interactions between a Lymphoid and a non-Lymphoid cell”, “Natural killer cell mediated cytotoxicity” and “Autoimmune thyroid disease” (Fig. S2a). Gene families underwent an expansion in these two species were involved in biological processes like “Cell Cycle”, “Meiotic synapsis”, “Mitotic Anaphase” and “Estrogen-dependent gene expression” ($P < 0.05$) (all Fisher’s exact test) (Table S7, Table S8).

Comment 1-8:
Line 131 The legend of Figure 1 should be revised, maybe "Genome assembly and gene family evolution in muskrat and musk deer"?. Other Figure legends should be corrected.
Response 1-8:
Thanks, as suggested, we have modified the legend of Figure 1 to: Genome assembly and gene family evolution in muskrat and musk deer. Other Figure legends were also corrected.

Comment 1-9:
Line 171-172 PSGs and REGs should be explained here or re-organized.
Response 1-9:
Thanks for the comment. The PSGs and REGs were re-organized to “Positive selection and rapid evolution genes in muskrat and musk deer were mainly involved in metabolism of lipids and epithelial regulation” part.

Comment 1-10:
Line 186-196: Each sub-figure should have a sub-legend, rather than mix them together, e.g. (b) and (c).
Response 1-10:
As suggested, we revised each sub-figure have a sub-legend (such as new Figure 2, Figure 3 and Figure 4 and so on).

Comment 1-11:
Line 187: "tissues *and* were used". Line 190: in the line"constructed at a probability of 0.95. (n = 19,800). (d)The cumulative", there should be no dot (.) after 0.95, "n" should be italic, also no space before "The". I hope the authors check and revise such typos carefully. In the following, I will not point them one by one.
Response 1-11:
Thanks for the comments. Changes have been made accordingly.

Comment 1-12:
Line 200: "We" to ", we". Line 219: Neutrophil.
Response 1-12:
Changes have been made as suggested.

Comment 1-13:
Line 238: total cell numbers of secretion (12128) and non-secretion (7270) stages are different, direct comparison of cell numbers in each cluster to infer the cell cluster related to secreting is not reasonable. The cell abundance of each cluster should be normalized firstly and them compared between the two stages. State it clearly here, cell number or cell abundance/proportion.

Powered by Editorial Manager® and ProduXion Manager® from Aries Systems Corporation

Response 1-13:
Thanks for the constructive suggestion.
Yes, for the comparison between two stages, we firstly normalized firstly cell abundance of each cluster in each stage. We have revised the statement in the revised manuscript.

Comment 1-14:
Line 270-271: It should be "Figure 4a". This sentence should be removed.
Response 1-14:
Change was made. As suggested, the sentence was removed in our revised manuscript.

Comment 1-15:
Line 296: In the part of HiC analysis, the authors investigated compartment and TAD dynamics between the secreting and non-secreting stages. Despite the observed massive difference, this part looks isolated from previous expression analysis. Logically the author analyzed the expression changes and inferred some candidate genes involved in the secreting progress from both bulk and scRNA-seq, the following step should be examining whether the conformation of these loci (e.g. CRABP2, Hacl1, Eci2, and Pecr) has changed in the secreting process, like from inactive compartment B to active compartment A, TAD fusion and fission. Although the authors described some cases (e.g. ROS1, SOX9, NUS1, NPNT), no expression data (Bulk, scRNA-seq) are provided. Similarly, the PEI analysis is weak. The author merged the HiC data and analyzed the interactions between promoters and enhancers, regardless of methodological issues, the meaningful PEIs should be those showing changes from non-secreting to secreting stage, like increased interaction strength.
Response 1-15:
Thanks for the helpful suggestions. As suggested, we reanalyzed the data. Thus, the candidate genes involved in the secreting progress from scRNA-seq expression were further logically analyzed whether the 3D genome conformation of these loci changed. As a result, Figure 4 and Figure S9 were regenerated.
At the PEI (promoter-enhancer interaction) level, we found that there were two genes (SMPDL3A and NRCAM) with specific PEI in musk secretion stage, also showed a compartment transition from B to A and was a marker gene in Cluster 14 (Fig. 4e). There were more specific long-range interactions (> 25Kb) in musk secretion stage for these two genes (Fig. 4f). The gene SMPDL3A showed a relative higher expression in all clusters at the musk secretion stage (Fig. 4g). SMPDL3A (sphingomyelin phosphodiesterase acid-like 3A) is an enzyme induced by lipid metabolism through liver X receptor that degrades cGAMP, modulating the cGAS-STING pathway which is involved in immune responses and lipid sensing [68]. NRCAM (neuronal cell adhesion molecule) primarily associated with neural development, its expression also promotes malignant cell transformation, cell motility, and metastatic disease [69]. In addition, MAP3K1, NODAL and Slc38a2 also showed significantly more PEIs and contacted with more enhancers during the musk secretion stage (Fig S10). MAP3K1 is a key component of the protein kinase signal transduction cascade and plays a crucial role in cellular signaling pathways [70]. NODAL maintains stem cell pluripotency and promotes directed differentiation [71]. Slc38a2 encodes an amino acid transport protein that facilitates cellular uptake of amino acids [72]. These results suggest that two important functions of the musk gland during the musk secretion stage are lipid metabolism and cell specialization, which indicate that synthesis and secretion activity were very active at this stage. Our results demonstrated that candidate loci can be analyzed in future studies of musk secretion mechanisms.

Figure S10. Promoter-enhancer interactions (PEIs) rewired in the musk gland of musk secretion and non-secretion stages.

Figure 4: Global chromatin interaction patterns in musk gland of musk secretion and non-secretion stages.

Comment 1-16:
Line 346: there is no statement in the manuscript referring to Fig 5f.
Response 1-16:
Thanks for the comment, as suggested, we reanalyzed our data and present

meaningful genes in our revised manuscript and cited the figures. See comment 1-15.

Comment 1-17:
Line 411: This part should be moved following the genome assembly part.
Response 1-17:
As suggested, this part was moved following the genome assembly part.

Comment 1-18:
Line471: The paragraph about five genes in REG and DEGs in cell cluster 14 should be moved to the scRNA-seq part.
Response 1-18:
Thanks for the helpful comment, as suggested, we moved this paragraph to the scRNA-seq part.

Comment 1-19:
Line 500: Database is useful but there are too many figures in the manuscript, and I would suggest shortening this part into one or two sentences in the conclusion section, and moving the figure in the supplementary material.
Response 1-19:
Thanks for the helpful comment. As suggested, there are only five figures in our revised manuscript, including this figure. And we also shortening this part.

Reviewer #2:
The manuscript titled "Multi-omics analyses identify distinct patterns of selection in musk secretion animals" by Wang et al. explored the molecular mechanisms of musk secretion using high-quality genome, Hi-C, RNA-seq, and scRNA-seq data. Additionally, they provide an open database platform (MuskDB), which is an important resource. Although the topic is interesting, some limitations in the text description and data analysis were found in this paper. After these concerns have been revised and clarified, I suggest that this manuscript could be accepted for publication.
Thank you for your positive comments. In our revised manuscript, we reorganized the text, figures and supplementary files. And logically, analyzed 3D genome reorganization of candidate genes with showing their expression profile in musk gland tissue and different cell types.

Comment 2-1:
1. In the part of abstract, the logical flow of the results differs significantly from the overall structure of the article, which can be confusing. Additionally, while the authors described their work using multi-omics analysis, just mentioned different pathways identified by each omics approach. Did the analysis revealed any common pathways or genes that are supported by multiple lines of evidence?
In addition, the application of musk in biomedicine should be mentioned to enhance the research significance of this paper.
Response 2-1:
Thanks for the helpful suggestions. In our revised manuscript, we reanalyzed our data. And presented candidate genes by multiple lines of evidence. The abstract was also rewritten.
In addition, as suggested, the application of musk in biomedicine was added: Natural musk has long been an important component of traditional Chinese medicine, and was used as resuscitation, blood circulation, collateral drainage, detumescence and pain relief [2].

Comment 2-2:
2. In introduction, it is suggested to propose the clear scientific questions and hypotheses. What are the key points of this study? Please highlights of these discoveries.
Response 2-2:
As suggested, in introduction, we propose the clear scientific questions: However, there are no convergent evolutionary studies on musk-producing animals to elucidate the related mechanism of musk secretion. In the process of evolution, which genes are subject to positive and convergent evolution? Which genes are differentially expressed in different cells of the musk gland during the period of musk secretion? And how the molecular process of musk secretion regulated by these genes from chromatin conformation is unknown.

Powered by Editorial Manager® and ProduXion Manager® from Aries Systems Corporation

The key points of this study were also rewritten: Notably, genes like SMPDL3A and NRCAM showed specific PEIs and compartment transitions, indicating their potential role in musk secretion regulation. The study concludes that the adaptation evolution of musk secretion in muskrat and musk deer is likely underpinned by active lipid metabolism and cell specialization, highlighting the complexity of the musk gland and the need for further research into the functional implications of musk secretion-specific genetic variants.

Comment 2-3:
3. The authors assembled the genomes of musk deer and muskrat using ONT, Hi-C, and NGS sequencing data. Given the relatively high error rate associated with ONT sequencing, appropriate correction methods should be applied. Additionally, the specific parameters used in the assembly process were not detailed. Please added in the methods section to provide a reference for these parameters.
Response 2-3:
Thank you for the constructive comments. As suggested, we added the specific detailed parameters used in the assembly process: For the correction of the initial contigs, we utilized the software NextPolish (v1.4.1), employing high-quality T7 paired-end reads and Nanopore long reads, and applied the recommended algorithm modules "best." in Nextpolish.
The specific parameters and pipelines used in the assembly process are available at Zenodo (https://doi.org/10.5281/zenodo.13690583).

Comment 2-4:
4. Figure S1 only shows the results for muskrats; I suggest including an additional figure to display the assembly results for musk deer. Moreover, both newly assembled genomes should be compared with existing reference genomes to further demonstrate the high quality of these assemblies, like collinearity comparison, et al.
Response 2-4:
As suggested, we compared with existing musk deer reference genomes to further demonstrate the high quality of our assemblies.
Our two assemblies have improved N50 length of the contig for the muskrat by 1,048- and 3.56-fold compared to the published sequences [9], respectively (Fig. S1b), and musk deer by 1,048- and 3.56-fold compared to the published sequences (Fig. S1c).

Figure S1: (b) Muskrat genome assembly in this study compared with two previous studies. (d) Musk deer genome assembly in this study compared with two previous studies.

Comment 2-5:
5. Which functions are the expanded and contracted gene families associated with? This should be detailed in a supplementary table.
Response 2-5:
Thanks for the constructive comments. Enrichment analysis of expanded and contracted genes in the two musk-secreting mammals were performed using KEGG pathway, Reactome and Go ontology. The Top 10 enrichment pathways are shown in Fig S2a and the original results of enrichment analysis are shown in Table S7-S8.
GO enrichment analyses of contracted genes showed that these two species were both involved in pathways like “Olfactory Signaling Pathway”, “Olfactory transduction”, “Signaling by GPCR”, “B cell receptor signaling pathway”, “Tight junction Immunoregulatory interactions between a Lymphoid and a non-Lymphoid cell”, “Natural killer cell mediated cytotoxicity” and “Autoimmune thyroid disease” (Fig. S2a). Gene families underwent an expansion in these two species were involved in biological processes like “Cell Cycle”, “Meiotic synapsis”, “Mitotic Anaphase” and “Estrogen-dependent gene expression” ($P < 0.05$) (all Fisher’s exact test) (Table S7, Table S8).

Comment 2-6:
6. Fig. 2e-d: The authors have depicted the gene expression level distribution. However, a tissue-specific expression profile might provide more insightful information.
Response 2-6:
Thanks for the helpful comments, we agree that a tissue-specific expression profile provide more insightful information. We provided the tissue-specific expression genes enrichment analysis results in Figure S4 for each tissue.

Powered by Editorial Manager® and ProduXion Manager® from Aries Systems Corporation

Fig. S4 Significantly enriched GO terms in tissue-specific expressed genes for each tissue. Tissue specificity of gene abundance was reflected by the tau score (τ).

Comment 2-7:
7. Line 168: The number of tissue-specific expressed genes for each tissue should be clearly described for clarity.
Response 2-7:
As suggested, we added the numbers of tissue-specific expressed genes for each tissue.
In terms of expression, there are 3 (uterus), 35 (testis), 27 (spleen), 6 (ovary), 26 (muscle), 9 (lung), 47 (liver), 23 (kidney), 19 (heart), 10 (gland), 8 (fat), 38 (eyeball), and 112 (brain) tissue-specific genes were detected.

Comment 2-8:
8. Line 171-174: It is unclear whether the PSG and REG described in this section are also tissue-specific expressed genes related to the musk gland. These PSGs and REGs should overlapped with musk-gland tissue-specific genes, and then be analyzed for possible functions.
Response 2-8:
Thanks for the helpful comments, the genes here was not musk-gland tissue-specific genes. Thus, we moved these genes' results to "Positive selection and rapid evolution genes in muskrat and musk deer were mainly involved in metabolism of lipids and epithelial regulation" part.

Comment 2-9:
9. If the authors later focus more on the biological function of the Acinar cells/glandular epithelial cells cluster, it would be beneficial to present the marker genes for this specific cell type using immunofluorescence staining.
Response 2-9:
Thanks for the helpful comments, the using immunofluorescence staining is very useful for present the marker genes. We need to collect sample from different musk secretion stage, this need time, we hope we could present this in our further studies.

Comment 2-10:
10. The overall methodological description in the single-cell transcriptomics section is overly simplistic. It is unclear whether the single-cell data from the two stages were analyzed jointly or separately. Additionally, it is not specified whether the pseudotime analysis was conducted using the secretion stage, the non-secretion stage, or both. These aspects need clarification to ensure a more comprehensive understanding of the analysis workflow. Providing detailed information on these points will help readers better understand the study's design and the interpretability of the results.
Response 2-10:
Thanks for the helpful comments, as suggested, we expanded methodological description in the single-cell transcriptomics section. 1) the single-cell data from the two stages were analyzed jointly; 2) pseudotime analysis was conducted using both stage cells.
Pseudotime Analysis
In R, utilizing the Monocle3 software package v1.3.1 (Cao et al., 2019), pseudotime trajectories for all 13, 14 and 22 subpopulations in the musk gland were constructed. This technique sorts individual cells along their developmental paths based on how closely their gene expression patterns match those of other sequenced cells, effectively mapping out the dynamics of cellular changes [110, 111]. Furthermore, the method evaluates how genes work together in space by using Moran's Index to measure their co-expression, which helps to establish a timeline of gene expression changes.
Identification of differentially expressed genes (DEGs) in Cluster 14 cells
DEGs in the Cluster 14 cells of musk secretion and non-secretion stages were identified using the FindMarkers function in Seurat. Genes meeting the thresholds ($|log2FC| > 1$, P-adjusted < 0.05, min.pct = 0.25) were considered as DEGs. The results were visualized using dot plots.

Comment 2-11:
11. The number of differentially expressed genes (DEGs) both in RNA-seq and scRNA-seq should be clearly specified, and detailed information should be presented in a

Powered by Editorial Manager® and ProduXion Manager® from Aries Systems Corporation

supplementary table. Additionally, a more stringent threshold should be applied for identifying DEGs: abs(log2FC)>1 and corrected ($P < 0.05$). Furthermore, it is important to determine whether the tissue-specific expressed genes in the musk gland are differentially expressed between the secretion and non-secretion stages.

Response 2-11:

Thanks for the helpful comments. The number of differentially expressed genes (DEGs) in RNA-seq were presented in Figure S3e a more stringent threshold (abs(log2FC)>1 and corrected ($P < 0.01$)).

As suggested, a supplementary table was provided for these tissue specific genes and DEGs for scRNA-seq (Table S11 and Table S14).

Figure S3e Heatmap of DEGs (differentially expressed genes) numbers in pairwise comparisons among the 13 tissues. DEGs were identified using the threshold of abs(log2FC)>1 and corrected ($P < 0.01$).

Comment 2-12:

12. Line 391-393: The authors explored the changes in PEIs during the secretion and non-secretion periods, but they did not further analyze the impact of these changes on the expression levels of the corresponding genes. Including such an analysis could provide valuable insights into the regulatory effects of PEI changes on gene expression and their potential biological implications.

Response 2-12:

Thanks for the constructive comments. As suggested, we reanalyzed the data. Thus, the candidate genes involved in the secreting progress from scRNA-seq expression were further logically analyzed whether the 3D genome conformation of these loci changed. As a result, Figure 4 and Figure S9 were regenerated. The expression of the genes showed a higher expression in musk secretion stage.

Figure 4. (e) Veen diagram showing overlapping genes with compartment B to A, PEI specific to musk secretion stage, and marker genes in Cluster 14. (f) Promoter-enhancer interactions (PEIs) rewired in the musk gland of musk secretion and non-secretion stages of SMPDL3A and NRCAM. (g) Gene expression of SMPDL3A and NRCAM in each cluster at musk secretion and non-secretion stages.

Comment 2-13:

13. line 417-419: The authors have provided limited information about the criteria used for selecting positively selected genes (PSGs) and rapidly evolving genes (REGs). It is important to clearly specify the threshold settings for these selections. Additionally, the genes identified through this process should be presented in a supplementary table to ensure transparency and allow for further analysis. Moreover, the authors could leverage the species-specific PSGs and REGs to identify sets of convergently positively selected genes and convergently rapidly evolving genes. This approach could yield insights into shared evolutionary pressures or functional adaptations across different species. Including these analyses would enhance the depth and comprehensiveness of the study.

Response 2-13:

Thanks for the comments. For PSGs, we utilized the free-ratio branch-site mode (model = 1) as an alternative model, assuming positive selection on the foreground branch. The null model allowed sites to undergo purifying selection or evolve neutrally. For REGs, we utilized the branch model, specifically the one-ratio model (model = 0) as the null model assuming the same evolutionary rate for all branches, and the two-ratio model (model = 2) as an alternative model allowing different evolutionary rates for the foreground branch. The likelihood ratio test (LRT) method was used to detect differences between the nested models, and P-values were computed based on $\chi 2$ statistics. Multiple testing was corrected using the false discovery rate (FDR) method. As suggested, we provided the PSGs, REGs and convergently genes (Table S6). Convergently positively selected genes and convergently rapidly evolving genes were also detected (Fig S3).

Fig S3. Overlapped genes between PSGs (REGs) and convergent evolution genes (a) and sequence alignment highlighting sites that evolved in 3 representative genes (b).

Comment 2-14:

14. line428: Identifying convergent amino acid substitution sites and genes can indeed be influenced by various confounding factors, potentially leading to a high rate of false

positives. Implementing a multi-method approach will enhance the validity and robustness of the study's conclusions. To improve the accuracy of these identifications, employing multiple methods is advisable. Methods such as "conv_cal" and "CCS" are effective for screening convergent amino acid substitutions. By using a combination of these methods, researchers can cross-verify findings, minimize false positives, and increase confidence in identifying true instances of convergent evolution.
Response 2-14:
As suggested, we used "CCS" methods to screen convergent amino acid substitutions.
Convergent Evolution among musk secretion animals
To test for convergence among musk secretion animals (muskrat and musk deer), we used 12 mammals from Fig. 1b. Based on the phylogenetic tree, we used two methods to detect the convergent amino acid substitutions for each node of the 7,409 single-copy orthologs: 1) method of Zhang and Kumar (Zhang JZ, Kumar S. 1997. Detection of convergent and parallel evolution at the amino acid sequence level. Mol Biol Evol. 14(5):527–536; Zou Z, Zhang J. 2015. Are convergent and parallel amino acid substitutions in protein evolutionmore prevalent than neutral expectations?Mol Biol Evol. 32(8):2085–2096.). A site was assumed as a convergent site if amino acids of a focused node at that site are the same but different with their most recent ancestral amino acids. Amino acid sequences of internal nodes for all the 7,409 single-copy orthologs were reconstructed by CODEML in PAML. For each gene, the number of observed convergent site was compared with the neutral expectations derived from the JTT-fgene model, and the Poisson test was then used to evaluate the difference.
2) CCS method (Xu S, He Z, Guo Zet al. Genome-wide convergence during evolution of mangroves from woody plants. Mol Biol Evol2017; 34: 1008–15.), the convergent signal is identified when all two musk secretion species (muskrat and musk deer) share the same derived character at a conservative site.

Comment 2-15:
15. line411: To ensure robust conclusions, it would be beneficial to include a broader range of species in the analysis. This expanded dataset would help determine the identified sites exhibit true convergence specifically in muskrat and musk deer, reducing the risk of false positives that might arise from a limited set of species.
Response 2-15:
Thanks for the helpful comments. We agree that broader range of species in the analysis will help determine the identified sites exhibit true convergence specifically in muskrat and musk deer, reducing the risk of false positives that might arise from a limited set of species. Considered the quality of the species’ genome and the number of one-to-one orthologous genes in the species, we selected 12 species for our analysis. We will sequenced more musk secretion species genome in our further study.

Comment 2-16:
16. line439: The TEX15 as a testis-specific protein in the text suggests that it may not be directly related to the species-specific trait of musk secretion in the musk gland. Some more apt examples, focusing on genes that are more directly associated with the unique characteristics of the musk gland could provide more relevant insights into the molecular underpinnings of this trait.
Response 2-16:
Thanks for the helpful comments. As suggested, we reanalyzed the data. Thus, the candidate genes involved in the secreting progress from scRNA-seq expression were further logically analyzed whether the 3D genome conformation of these loci changed. As a result, Figure 4 and Figure S9 were regenerated.
At the PEI (promoter-enhancer interaction) level, we found that there were two genes (SMPDL3A and NRCAM) with specific PEI in musk secretion stage, also showed a compartment transition from B to A and was a marker gene in Cluster 14 (Fig. 4e). There were more specific long-range interactions (> 25Kb) in musk secretion stage for these two genes (Fig. 4f). The gene SMPDL3A showed a relative higher expression in all clusters at the musk secretion stage (Fig. 4g). SMPDL3A (sphingomyelin phosphodiesterase acid-like 3A) is an enzyme induced by lipid metabolism through liver X receptor that degrades cGAMP, modulating the cGAS-STING pathway which is involved in immune responses and lipid sensing [68]. NRCAM (neuronal cell adhesion molecule) primarily associated with neural development, its expression also promotes malignant cell transformation, cell motility, and metastatic disease [69]. In addition, MAP3K1, NODAL and Slc38a2 also showed significantly more PEIs and contacted with more enhancers during the musk secretion stage (Fig S9). MAP3K1 is a key

Powered by Editorial Manager® and ProduXion Manager® from Aries Systems Corporation

component of the protein kinase signal transduction cascade and plays a crucial role in cellular signaling pathways [70]. NODAL maintains stem cell pluripotency and promotes directed differentiation [71]. Slc38a2 encodes an amino acid transport protein that facilitates cellular uptake of amino acids [72]. These results suggest that two important functions of the musk gland during the musk secretion stage are lipid metabolism and cell specialization, which indicate that synthesis and secretion activity were very active at this stage. Our results demonstrated that candidate loci can be analyzed in future studies of musk secretion mechanisms.

Comment 2-17:
17. The manuscript shows p-values in all of the pathway enrichment figures, yet the methods section states that p-value correction for multiple testing was done. Additionally, line 431 mentions using p-values to indicate significance. The authors need to clarify this discrepancy regarding multiple testing correction.
It is important for the authors to specify whether the p-values displayed in the figures and mentioned in the text are raw or have been corrected for multiple comparisons. This transparency is essential to ensure that the reported significance levels accurately reflect the analyses' reliability, considering the potential for false positives when multiple tests are performed. Clearly explaining these details is crucial for the study's credibility and reproducibility.
Response 2-17:
Thanks for the comments. As suggested, we have revised the p-value correction methods in our revised manuscript. It is corrected for multiple testing by the Benjamini-Hochberg method as described in our methods part.

Comment 2-18:
18. line452: It would be necessary to provide further details on the three adaptive convergent genes, such as NOP2, DST, and FAM160A1. For instance, creating a visual representation like figure, could help illustrate the situation within the context of convergent evolution.
Response 2-18:
Thanks for the constructive comments. As suggested, we generated a figure to illustrate the situation within the context of convergent evolution (Figure S3).

Fig S3. Overlapped genes between PSGs (REGs) and convergent evolution genes (a) and sequence alignment highlighting sites that evolved in 3 representative genes (b).

Comment 2-19:
19. The authors should explore whether any of the convergently positively selected genes or convergent amino acid replacement genes are related to musk secretion in the musk gland. Investigating whether these genes are specifically expressed in musk gland tissue or display species-specific expression can yield valuable insights into their relevance to the unique traits of the musk gland. These analyses could help to establish a connection between these genes and the adaptive characteristics associated with musk gland function.
Response 2-19:
Thanks for the constructive comments. As suggested, we analyzed whether the convergently positively selected genes or convergent amino acid replacement genes are related to musk secretion in the musk gland. Because there are only 10 musk gland tissue specific genes, there was no overlap with the convergently positively selected genes. Among these 10 genes, KRT80 and MPIG6B genes were also PSGs in muskrat. These two genes were also limited studied. Keratin 80 (KRT80) is an intermediate filament protein that contributes to the structural integrity of epithelial cells. The megakaryocyte and platelet inhibitory receptor gene G6P (MPIG6B) regulates platelets production, aggregation, and activation.

Comment 2-20:
20. In conclusion, the most important findings of this study should be clarified, and the description of the relevant methods will not be repeated.
Response 2-20:
Thanks for the comments. We rewritten the most important findings of this study in both abstract and introduction:
The study identified particular genes, such as SMPDL3A and NRCAM, that exhibit unique patterns of genetic interactions and changes in their genomic neighborhoods,

Powered by Editorial Manager® and ProduXion Manager® from Aries Systems Corporation

	suggesting they may play a key role in controlling the musk secretion process. The research concludes that the evolutionary adaptation for musk production in both the muskrat and the musk deer is likely driven by robust lipid metabolism and specialized cell functions, which underscores the intricate nature of the musk gland. This finding emphasizes the necessity for continued investigation to uncover the full functional impact of genetic variations that are specific to the musk secretion process.<br>The repeated methods were revised in our new manuscript.<br><br>Comment 2-21:<br>Minor suggestions:<br>1. Acronyms: Ensure that all acronyms are spelled out full name in the first time when they appear in the text for better understanding.<br>2. line691 and line698 are same.<br>3. Methods descriptions should also be listed in order of article logic and importance. For example, you should first write "sample collection" section and so on.<br>Response 2-21:<br>Thanks for the constructive comments. As suggested, all acronyms are spelled out full name in the first time when they appear in the text for better understanding; the repeated methods were removed; we first write "sample collection".<br><br>Reviewer #3<br>Comment 3-1:<br>Natural musk is mainly secreted by musk gland located between the navel and genitals of mature male forest musk deer. But the molecular mechanism of musk-secretion is still wait to be uncovered, hindered by the lack of comprehensive multi-omics analyses and respective platform. In this study, the authors successfully generated high quality 2.48 Gb and 2.83 Gb for muskrat and musk deer genomes with contig N50 values of 60.53 and 69.45Mb, which anchored onto 28 and 30 chromosomes, respectively. To explore the genes specifically expressed in musk gland and their functions, we used muskrat as a model animal to conduct further analyses, as muskrat tissue samples are accessible in contrast to the endangered musk deer by 168 muskrat transcriptomes. And the further single cell RNA sequencing and Hi-C analysis indicated that enhanced expression during the musk secretion stage of muskrat is related to the biological process "regulation of secretion". Overall, these results show that active lipid metabolism may underlie the adaptation evolution of musk secretion. Those findings, alongside the provided database, facilitate a deeper understanding of the molecular mechanism underlying the unique phenomenon of musk secretion, and may provide insights for the mating behavior and breeding of muskrat and musk deer.<br>The findings are meaningful, the database is very important. I think it could be accepted to be published after a little polish.<br>Response 3-1:<br>We are delighted to receive your comments. We sincerely appreciate the thoughtful and constructive comments from you and another two anonymous reviewers, in improving our manuscript.<br>As suggested, we mainly focused the analyses on the genes with both expression and 3D genomic changes. In addition, in our revised manuscript, 1) we regenerated all figures, tables and supplementary files; 2) added and revised relative results, methods and discussion; 3) we polish our manuscript with a professional English language editing company. All comments with our point-by-point responses to each reviewer are listed. We sincerely appreciate your assistance in improving our manuscript.
**Additional Information:**	
**Question**	**Response**
Are you submitting this manuscript to a special series or article collection?	No
**Experimental design and statistics**<br><br><br>Full details of the experimental design and statistical methods used should be given in the Methods section, as detailed in our	Yes

Powered by Editorial Manager® and ProduXion Manager® from Aries Systems Corporation

Minimum Standards Reporting Checklist. Information essential to interpreting the data presented should be made available in the figure legends.<br><br>Have you included all the information requested in your manuscript?	
**Resources**<br><br>A description of all resources used, including antibodies, cell lines, animals and software tools, with enough information to allow them to be uniquely identified, should be included in the Methods section. Authors are strongly encouraged to cite Research Resource Identifiers (RRIDs) for antibodies, model organisms and tools, where possible.<br><br>Have you included the information requested as detailed in our Minimum Standards Reporting Checklist?	Yes
**Availability of data and materials**<br><br>All datasets and code on which the conclusions of the paper rely must be either included in your submission or deposited in publicly available repositories (where available and ethically appropriate), referencing such data using a unique identifier in the references and in the "Availability of Data and Materials" section of your manuscript.<br><br>Have you have met the above requirement as detailed in our Minimum Standards Reporting Checklist?	Yes

Manuscript
Click here to access/download;Manuscript;Revised MS.docx

Multi-omics analyses identify distinct patterns of selection in musk secretion animals

Tao Wang[1], Maosen Yang[2,3], Xin Shi[4], Shilin Tian[5], Yan Li[6], Wenqian Xie[7], Zhengting Zou[8], Dong Leng[9], Ming Zhang[9], Chengli Zheng[4], Chungang Feng[10], Bo Zeng[9], Xiaolan Fan[9], Huimin Qiu[11], Jing Li[11], Guijun Zhao[3], Zhengrong Yuan[7*], Diyan Li[3*] and Hang Jie[2*]

[1] School of Basic Medical Sciences, Chengdu University, Chengdu, 610106, China
[2] Jinfo Mountain Forestry Ecosystem of Chongqing Observation and Research Station, Chongqing Institute of medicinal plant cultivation, Chongqing University of Chinese Medicine, Chongqing 402760, China
[3] School of Pharmacy, Chengdu University, Chengdu 610106, China
[4] Sichuan Institute of Musk Deer Breeding, Chengdu 611845, China
[5] College of Life Sciences, Wuhan University, Wuhan 430072, China
[6] Chengdu Research Base of Giant Panda Breeding, Chengdu 611081, China
[7] College of Biological Sciences and Technology, Beijing Forestry University, Beijing 100083, China
[8] Key Laboratory of Zoological Systematics and Evolution, Institute of Zoology, Chinese Academy of Sciences, Beijing 100101, China
[9] College of Animal Science and Technology, Sichuan Agricultural University, Chengdu 611130, China
[10] College of Animal Science and Technology, Nanjing Agricultural University, Nanjing 210095 China
[11] College of Agriculture, Kunming University, Kunming 650214, China

Tao Wang, Maosen Yang, Xin Shi, Shilin Tian, Yan Li and Wenqian Xie contribute equally to this work.
[*] For correspondence: Zhengrong Yuan, Diyan Li and Hang Jie.

Abstract

Background

Musk, secreted by the musk gland of adult male musk-secreting mammals, holds significant pharmaceutical and cosmetic potential. However, understanding the molecular mechanisms of musk secretion remain limited, largely due to the lack of comprehensive multi-omics analyses and available platforms for relevant species, such as muskrats (*Ondatra zibethicus* Linnaeus) and Chinese forest musk deer (*Moschus berezovskii* Flerov).

Results

We generated chromosome-level genomes for both species (*Ondatra zibethicus* Linnaeus and *Moschus berezovskii* Flerov) along with 168 muskrat transcriptomes. Comparative analysis with eleven other vertebrate genomes revealed genes and amino acid sites with signs of adaptive convergent evolution, primarily linked to lipid metabolism, cell cycle regulation, protein binding, and immunity. Single-cell RNA sequencing and Hi-C analyses demonstrated enhanced gene expression during muskrat musk secretion, particularly in biological processes related to “regulation of secretion.”

Additionally, we developed MuskDB (http://117.78.45.2:1087/home), a freely accessible multi-omics database platform for musk-secreting mammals.

Conclusions

Genes such as *SMPDL3A* and *NRCAM* exhibited specific PEIs and compartment transitions, suggesting a role in musk secretion. The study concludes that the evolution of musk secretion in muskrats and musk deer is likely driven by lipid metabolism and cell specialization. This underscores the complexity of the musk gland and calls for further investigation into musk secretion-specific genetic variants.

Introduction

Natural musk (Moschus) is mainly secreted by the musk gland located between the navel and genitals of mature male forest musk deer (*Moschus berezovskii* Flerov), an endangered artiodactyl species native to southern and central China and northernmost Vietnam [1]. Natural musk has long been an important component of traditional Chinese medicine and was used for resuscitation, blood circulation, collateral drainage, detumescence, and pain relief [2]. In addition, the muskrat (*Ondatra zibethicus* Linnaeus), a semiaquatic rodent native to North America [3] and Canada but has been introduced to Europe, Asia, South America, and Australia, has similar musk gland and secret musk likewise. The musk secreted by forest musk deer and muskrat produces a specific fragrance, whose chemical composition may be involved in chemical communication, potentially encoding information about sexual maturity and attraction [4]. The chemical composition analysis of musk showed that it contained active macrocyclic ketone components such as muscone and normuscone [5]. Muskrat musk also contains macrocyclic ketone compounds such as muscone and normuscone [6]. This class of substances is thought to be necessary for exerting drug effects. Our previous research indicated that musk of muskrat and musk deer have up to 272 identical metabolites, including organic compounds such as amino acids, fatty acids, ketones, aldehydes, and steroids [7].

Compared with other musk-secreting mammals (i.e., other musk deer species), there has been more captive breeding practice for the forest musk deer and the muskrat. The high-quality genome sequences of these two species and comparative analyses with the other mammalian genomes can potentially shed light on their genome diversity and the genetic components underlying musk secretion, which may have experienced convergent adaptation during the long process of evolution. However, there are no convergent evolutionary studies on musk-producing animals to elucidate the related mechanism of musk secretion. In the process of evolution, it is crucial to identify which genes are subject to positive and convergent evolution. Additionally, identifying the genes that are differentially expressed in different cells of the musk gland during musk secretion is critical. The regulation of the molecular process of musk secretion by these genes from chromatin conformation remains unknown.

Here, we sequenced the genomes of a male muskrat and a male musk deer. In addition, to characterize the transcriptomic variability with respect to known tissue-specific physiological activities and identify key genes underlying the musk-secreting

phenotype, we sequenced 84 RNA-seq libraries and 84 small RNA-seq libraries of 13 various muskrat organs. To accurately depict cell composition and transcriptomic changes in the musk gland of muskrat between musk secretion and non-secretion stages, we further used a single-cell RNA (scRNA) approach to dissect the transcriptional differences. We also examined the potential chromatin architecture dynamics underlying the phenotype by sequencing three and four Hi-C libraries for the musk gland, respectively, in the secretion and non-secretion stages (**Supplementary Table S1**). Integrated with these multi-omics data, the study identified particular genes, such as *SMPDL3A* and *NRCAM*, that exhibit unique patterns of genetic interactions and changes in their genomic neighborhoods, suggesting they may play a key role in controlling the musk secretion process. The research concludes that the evolutionary adaptation for musk production in both the muskrat and the musk deer is likely driven by robust lipid metabolism and specialized cell functions, underscoring the musk gland's intricate nature. This finding emphasizes the necessity for continued investigation to uncover the full functional impact of genetic variations specific to the musk secretion process.

Results and discussion

Genome assembly of two musk-secreting mammals

We sequenced the genomes of two male (only male secret musk) musk-secreting mammals [*Ondatra zibethicus* (muskrat) (2 years of age) and *Moschus berezovskii Flerov* (forest musk deer) (2.5 years of age)] via integration of Oxford Nanopore Technologies (ONT) long reads, high-throughput chromosome conformation capture (Hi-C) data and BGI T7 paired-end sequences (**Supplementary Table S1**) to over 235.25-fold (~583.41 Gb) and 246.02-fold (~696.23 Gb) coverage, respectively. We assembled the two chromosome-level genomes by applying an improved assembly method that utilizes Hi-C interaction pairs to cluster ONT long sequences with potential linkages and avoid any erroneous overlap caused by long-distance repetitive sequences during string graph assembly [8] (**see methods**). We successfully generated 2.48 Gb and 2.83 Gb for muskrat and musk deer genomes with contig N50 values of 60.53 and 69.45Mb, which anchored onto 28 and 30 chromosomes, respectively (**Supplementary Table 1, Supplementary Table S2; Fig. 1a**). The 28 anchored chromosomes in muskrat were confirmed by karyotype analysis (**Supplementary Fig. S1a**). In particular, five chromosome sequences have reached the gap-free level in the muskrat genome (**Supplementary Table S3**), and our two assemblies have improved the N50 length of the contig by 1,048- and 3.56-fold for the muskrat, 1,048- and 3.56-fold for musk deer compared to the published sequences, respectively (**Supplementary Fig. S1b-c**). Our assembled genomes exhibit excellent completeness, as evidenced by the coverage of > 99% paired-end reads across > 99% of the genome, and recovery of averaged 96.95% of BUSCOs (Benchmarking Universal Single-Copy Orthologs) [9] in 9,226 conserved mammalian genes from the mammalia_odb10 database (**Supplementary Table S4**). Furthermore, we used a reference-free and *k*-mer-based approach and estimated a high assembly quality value (QV) of more than 44, exceeding

the Vertebrate Genome Project (VGP) standard of QV40 [10, 11]. Subsequently, we predicted 1013.31 Mb (40.85%) and 1539.41 Mb (54.33%) transposable elements (TEs) for muskrat and musk deer, respectively (**Supplementary Table S5**). By combining homology- and *ab initio*-based methods, aided by evidence of transcription, we identified 23,260 and 24,375 protein-coding genes in the muskrat and musk deer genomes, respectively (**Supplementary Table S5, Supplementary Fig. S1d-e**).

Table 1. Global summary of two assemblies for muskrat and musk deer

Genomic features	Muskrat	Musk deer
Assembled genome size (Gb)	2.48	2.83
Percentage of anchoring (%)	97.19	98.79
Contig Number	561	1,173
Contig N50 (Mb)	60.53	69.45
GC content (%)	41.69	42.14
Repeat ratio (%)	40.85	54.33
Predicted number of protein coding genes	23,260	24,375
QV	44.48	44.09
BUSCOs (%)	96.89	96.28

Next, we explored gene family expansion and contraction in two musk secretion species. As a result, we determined that 181 gene families underwent an expansion, and 134 underwent a contraction for the musk deer. Muskrat shows comparable numbers of gene family contraction (166) and expansion (161) events (**Fig. 1b**). GO enrichment analyses of contracted genes indicated that these two species were both involved in pathways like “Olfactory Signaling Pathway,” “Olfactory transduction,” “Signaling by GPCR,” “B cell receptor signaling pathway,” “Tight junction Immunoregulatory interactions between a Lymphoid and a non-Lymphoid cell,” “Natural killer cell-mediated cytotoxicity” and “Autoimmune thyroid disease” (**Supplementary Fig. S2a**). Gene families underwent an expansion in these two species and were involved in biological processes like “Cell Cycle,” “Meiotic synapsis,” “Mitotic Anaphase,” and “Estrogen-dependent gene expression” ($P < 0.05$) (all Fisher’s exact test) (**Supplementary Table S7-8**).

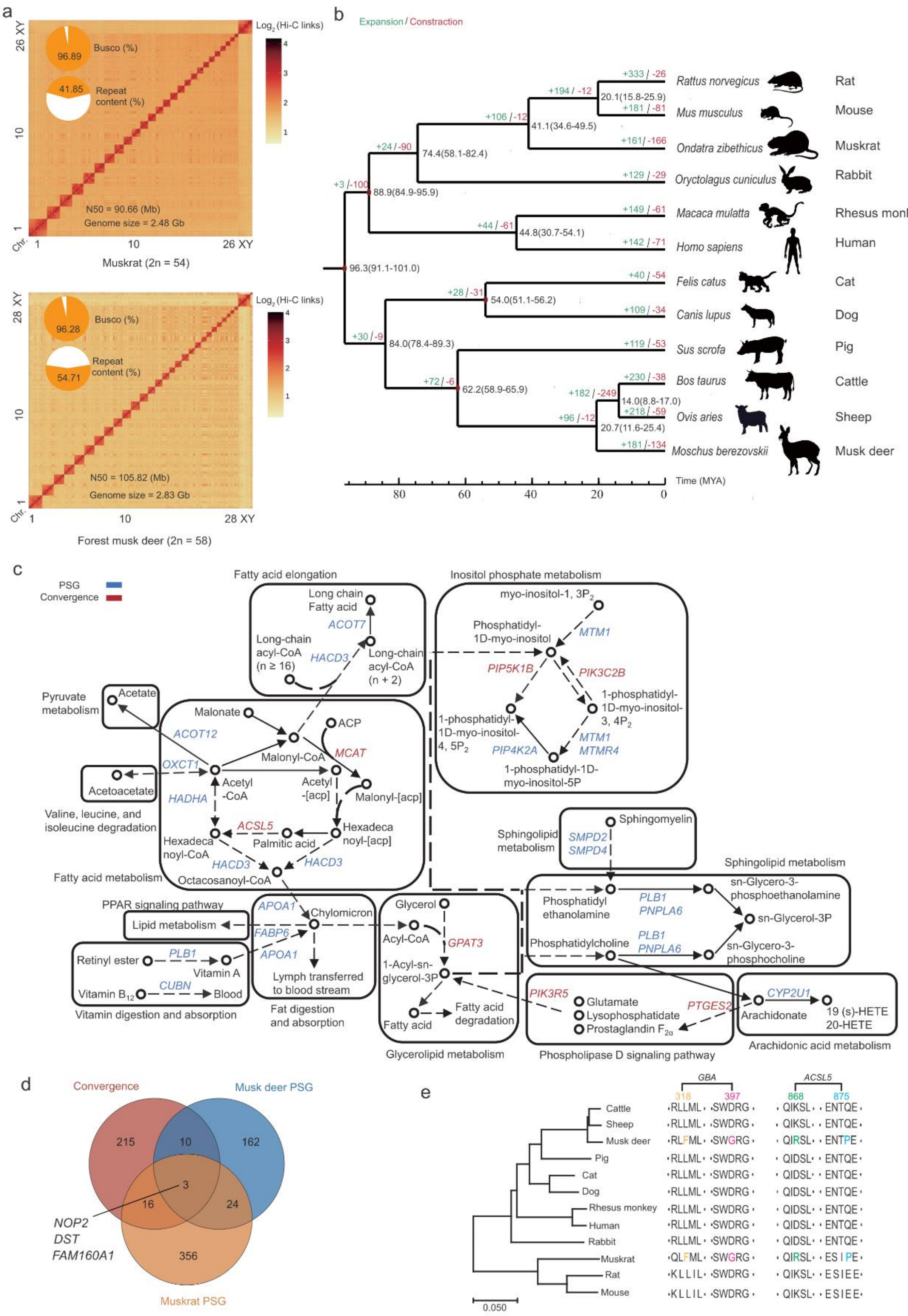

Figure 1: **Genome assembly and gene family evolution in muskrat and musk deer.** (a) Hi-C heatmaps for muskrat and forest musk deer. Pie charts represent the proportion of conserved BUSCO gene sets and repeat content. Contig N50 and assembled genome size are shown. (b) Divergence times and expansion and contraction of gene families in muskrat and musk deer genomes. Numbers on the nodes represent divergence times, with the error range shown in parentheses. The numbers of gene families that expanded (green) or contracted (red) in each lineage

after speciation are shown on the corresponding branch. (c) Enrichment for PSGs and genes evolved in parallel that functions in the metabolism of lipids (only partial pathways were shown). The PSGs are shown in blue, and the parallel genes are shown in red. (d) The Venn diagram shows the number of PSGs and REGs in muskrat and musk deer. (e)The sequence alignment shows sites that evolved in parallel in *GBA* and *ACSL5*.

Positive selection and rapid evolution genes in muskrat and musk deer were mainly involved in the metabolism of lipids and epithelial regulation

To explore the function of positively selected genes (PSGs) and rapidly evolving genes (REGs) in muskrat and musk deer during evolution, along with chromatin structure changed genes. We next analyzed 7,409 gene trees based on one-to-one orthologs, each constrained to the reconstructed species phylogeny. By applying branch tests and branch-site tests in PAML [12] to the corresponding branches, we identified 443 rapidly evolving genes (REGs) and 399 positively selected genes (PSGs) for muskrats, 523 REGs, and 199 PSGs for musk deer, respectively **(Supplementary Table S6)**. Interestingly, the gene *Hdac1* was in GO term "epidermal cell differentiation," which is enriched by gene family expansion in the forest musk deer and muskrat, coinciding with the musk gland-specific gene. These respective sets of genes were mainly involved in the "Metabolism of proteins" and "Metabolism of lipids" (**Supplementary Table S9-10**). We also identified that PSGs like *ANAPC4*, *CDC16*, and *RBL2* were involved in the cell cycle pathway (**Supplementary Fig. S2b**). Muskrat PSGs (*HBEGF*, *PIGR*, *PLCE1*, *NCK2*) were enriched in the "Epidermal growth factor receptor signaling pathway", and REG (*TGFBR3*, *BMP4*, *STRAP*, *ATF2*, *CDC73*) were enriched in "Negative regulation of epithelial cell proliferation". These results indicated that the genes responsible for epidermis development have a high and specific expression in the musk gland and have experienced putatively adaptive evolutionary expansion. In addition, we deduced the convergent sites in musk secretion species (muskrat and musk deer) at each node for all the 7,409 single-copy orthologs based on the phylogenetic tree (**Fig 1b**) using two methods (JTT-Fgene [13, 14] and CCS [15]). As a result, total of 244 genes were identified under the JTT-Fgene model (FDR < 0.05, Poisson test), whereas 457 genes were identified by the CCS method [15]. 209 genes were detected as convergent evolution for muskrat and musk deer by both two methods (JTT-Fgene and CCS). We performed functional gene enrichment analyses for convergent evolution genes, and found that the gene sets were also significantly enriched for "Metabolism of proteins" ($P = 0.045$, Fisher's exact test), "Metabolism of lipids and lipoproteins" ($P = 0.035$, Fisher's exact test).

RDH8 is a convergent evolution gene involved in "The canonical retinoid cycle in rods (twilight vision)" in muskrat and musk deer. Although olfactory receptor genes are contracted in these two species, several vision-related genes were PSGs or convergent

evolution genes, indicating they have a sensory trade-off otherwise observed in arboreal species [16] and giraffes [17], which is consistent with the fact that both species are timid and sensitive. TEX15, a testis-specific protein, is required for TE silencing. A previous study indicated that TEX15, a new essential epigenetic regulator, may function as a nuclear effector of MILI to silence TEs by DNA methylation [18]. Our study suggested that the *TEX15* gene is an outlier with seven unique amino acid convergent substitutions for muskrat and musk deer (**Supplementary Fig. S2c**). This indicated that the *TEX15* gene not only plays a role in male germ cells, but it might also have an essential role in forming the male characteristic organ of a musk-secreting species.

For the "Metabolism of lipids and lipoproteins" pathway, we identified 10 convergent evolution genes (*SLC44A2, GBA, PTGES2, GPAT3, PIK3R5, ACSL5, MED12, ACBD6, PIP5K1B, BDH2*) in this pathway (*P* value = 0.035, Fisher's exact test) in muskrat and musk deer (**Fig. 1c**). We also checked the overlapped genes between PSGs (REGs) and convergent evolution genes, and found *NOP2*, *DST*, *FAM160A1* and *CKAP5* were shared between them (**Fig. 1d, Supplementary Fig. S3**). These genes have been less studied, such as FAM160A1, a member of the UPF0518 family of proteins, each containing a conserved retinoic acid-induced 16 (RAI16)-like domain with unknown biological function [19]. CKAP5 enables the formation of persistent actin bundles on dynamic microtubules [20]. Among these convergent evolution genes, *ACSL5* and *GBA* have two amino acid substitutions (**Fig. 1e**). The protein encoded by the *ACSL5* gene is an isozyme of the long-chain fatty-acid-coenzyme A ligase family, which catalyzes the formation of fatty acyl-CoAs from long-chain fatty acids (C16–C20). Fatty acyl-CoAs are then used in lipid synthesis or β-oxidation mediated pathways [21]. The *GBA1* gene encodes the lysosomal enzyme beta-glucocerebrosidase (GCase) that degrades glucosylceramide, and is pivotal in glycosphingolipid substrate metabolism [22]. In addition, choline is essential for synthesizing phospholipids [23], and the gene *SLC44A2* participated in the process. These results indicated a rapid evolution of lipid metabolism in these two species. In addition, some convergent evolution genes that also have roles in "Metabolism of lipids" ($P = 0.0002$) and "Cell Cycle, Mitotic" ($P = 0.006$) were also detected (**Supplementary Fig. S2a**). This might be because the male musk gland has a cyclic change; in the musk secretion stage, the musk gland is atrophic, and in the stage of musk non-secretion, the glands become larger, accompanied by cell proliferation and differentiation.

Tissue-specific expression of genes in the musk gland

To explore the genes specifically expressed in the musk gland and their functions, we used a muskrat as a model animal to conduct further analyses, as muskrat tissue samples are accessible in contrast to the endangered musk deer. We first constructed 84 RNA-seq libraries and 84 small RNA-seq libraries to explore tissue-specific expression

patterns in the transcriptome of muskrat among 13 tissues (two from entoderm [liver and lung], eight from mesoderm [testis, heart, spleen, kidney, muscle, fat, uterus, and ovary], and three from ectoderm [brain, eyeball, and musk gland]) (**Fig. 2a**), with at least six biological replicates for each stage. We then updated the annotation of distinct transcript types, including lncRNAs and miRNAs (**Supplementary Fig. S4a**), representing a core atlas dataset of *de novo* assembled transcripts. There are 10 and 3 gland-tissue-specific expressed lncRNAs and miRNAs were detected, respectively (**Supplementary Fig. S4b**).

After filtering the low expression levels genes with transcripts per million (TPM) < 1 in at least 50% samples in each analyzed tissue, we evaluated the expression levels of 14,861 (63.89%) muskrat genes and mainly looked for tissue-specific expression patterns. The results showed that the transcriptional profiles of each tissue type are highly reproducible among biological replicates (Spearman's $r > 0.80$) (**Fig. 2b**). Ovary, musk gland, and uterus also clustered into obviously separate respective groups. Meanwhile, brain and eyeball tissues clustered together (**Fig. 2c**). More than 60% of genes were expressed in each tissue for muskrat (**Supplementary Fig. S4c**), but expression levels of different genes are skewed. In most tissues, the expression of highly expressed 1,000 genes takes up an average expression of more than 50%, especially for muscle (**Fig. 2d**). We also observed dissimilarities between the gene expression level distribution across tissues. The most abundant transcripts (the top 1,000, as ranked by expression levels) in a tissue, accounted for greater than half of the total transcribed muscle (~71.71%), liver (~68.76%), and heart (~61.34%), whereas testis (~37.70%) had a more uniform distribution (**Fig. 2e, Supplementary Fig. S4d**). Testis showed the highest number of differentially expressed genes compared with other tissues (**Supplementary Fig. S4e**).

In terms of protein-coding gene expression, there are 3 (uterus), 35 (testis), 27 (spleen), 6 (ovary), 26 (muscle), 9 (lung), 47 (liver), 23 (kidney), 19 (heart), 10 (gland), 8 (fat), 38 (eyeball), and 112 (brain) tissue-specific genes were detected (**Supplementary Table S11**). The tissue-specific genes were commonly enriched in distinct cellular functions. For example, the specifically expressed genes for the musk gland were mainly involved in "epidermis development" and those for testis in "male gamete generation" and "meiotic nuclear division" (**Supplementary Fig. S5**). Notably, among the genes specifically expressed in musk gland (**Fig. 2f**), *KRT80* and *MPIG6B* genes were also PSGs in muskrat. Keratin 80 (KRT80) is an intermediate filament protein that contributes to the structural integrity of epithelial cells [24]. The megakaryocyte and platelet inhibitory receptor gene G6P (*MPIG6B*) regulates platelet production, aggregation, and activation [25]. In addition, we also found that some musk gland-specific genes are related to lipid metabolism, such as *TRPV3* and *LIPM* (**Fig. 2f**). Transient receptor potential (TRP) channels are polymodal sensors that convert a

multitude of environmental cues into cellular signaling events essential for physiology [26]. *TRPV3* is activated by warm temperatures and numerous chemicals, including plant extracts, lipid metabolites, and synthetic small molecules such as 2-aminoethoxydiphenyl borate (2-APB) [27, 28]. In mice, *LIPM* (lipase) also has a restricted tissue expression in the epidermal tissue [29], supporting the unique and active function of *LIPM* in musk gland function.

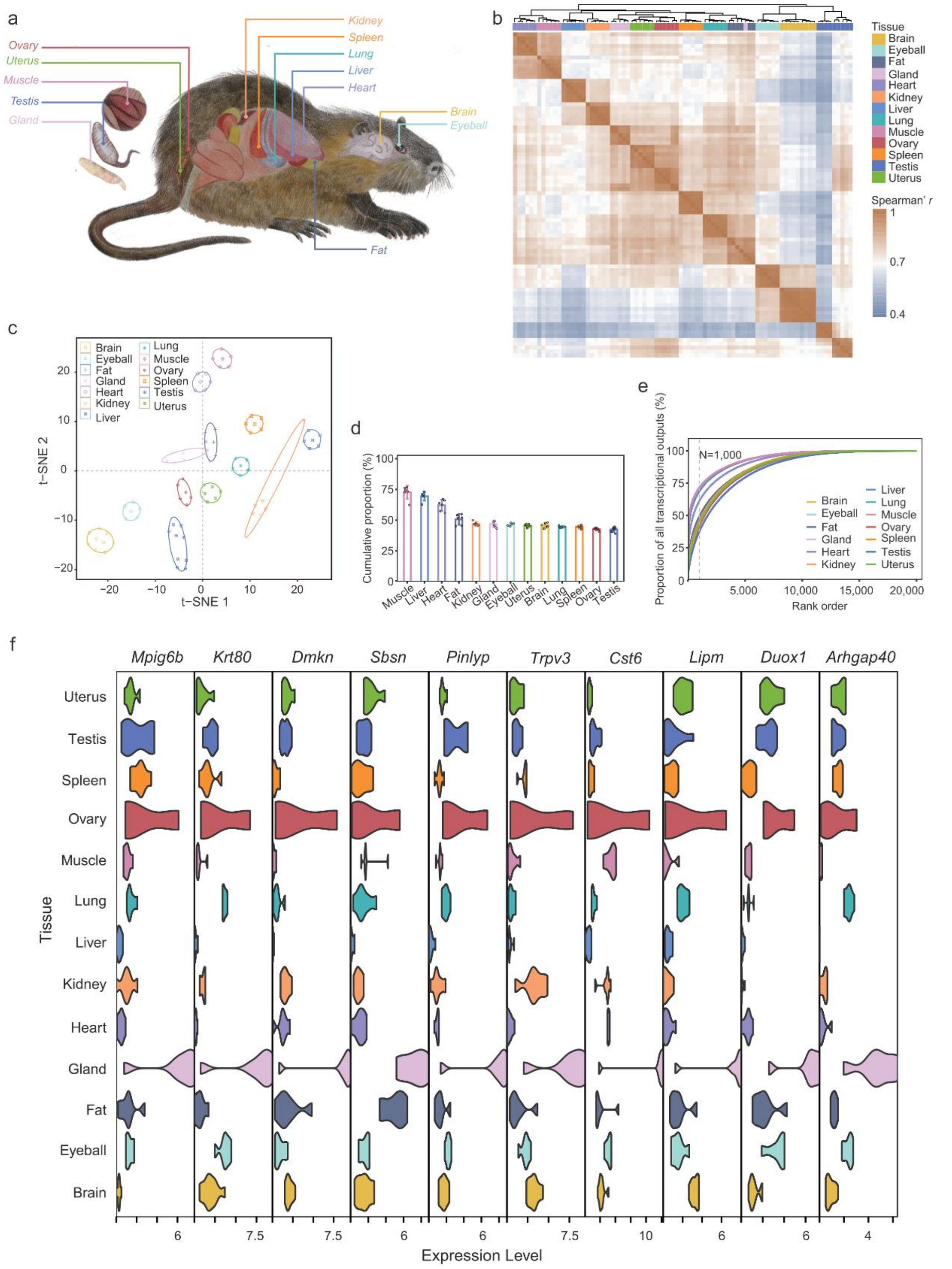

Figure 2: Characteristics of the muskrat BodyMap transcriptome. (a) Samples derived from 13

tissues were used for muskrat transcriptome reconstruction. (b) Hierarchical clustering and Spearman' *r* heatmap of samples using expression values (TPM). (c) t - distributed stochastic neighbor embedding (t-SNE) clustering of samples using expression values (TPM). The ellipses indicate the samples of the same tissue with similar transcriptional profiles, constructed at a probability of 0.95 (n = 19,800). (d) The cumulative expression proportion of the top 1,000 highly expressed genes in all samples. (e) Abundance distribution of transcripts across 13 tissues. The x-axis indicates the proportion of transcripts sorted from highest to lowest expression, with the vertical dashed line indicating the top 1,000 of the highest abundance transcripts. The y-axis indicates the accumulated fraction of transcripts relative to the total transcripts. Colored lines represent mean values across different tissues. (f) The genes specifically expressed in musk gland.

Single-cell reconstruction of musk secretion remodeling in the muskrat adult musk gland

To depict transcriptomic changes during musk secretion at the single-cell level, we further measured the transcriptional differences between representative secretion and non-secretion stages in the musk gland by 10× Genomics scRNA-seq system (**Fig. 3a**). After quality filtering, the transcriptome profiles of 19,398 cells were available for cell-type characterization (12,128, and 7,270 cells for musk secretion and non-secretion stages, respectively) (**Supplementary Table S1**). To explore the cell types of these musk glands, we performed the uniform manifold approximation and projection (UMAP) analysis and identified 23 cell clusters (**Fig. 3b, c**). We surveyed the expression patterns of the top 50 most variable genes (**Supplementary Fig. S6a**), which could cluster these cells into 13 known cell types (**Supplementary Table S12**).

We found the vast majority of collected cells (49.53% and 59.16%) possess characteristics typical of fibroblasts (clusters 1, 2, 3, 4, 5, 11, and 18) with higher expression of *IGFBP3* [30, 31], *DCN* [32], and *C3* [33] (**Supplementary Fig. S6b**). Clusters 0, 10, 16, and 21 were identified as macrophages with higher expression of *C1qa*, *C1qb*, *C1qc*, *Ctss*, *Cd14,* and *Cd68* [34]; cluster 21 with higher expression of *Coro1a* [34] and *Cd74* [35, 36]. Cluster 17 was identified as mastocytes with expressed *Alox5*, *Cpa3*, *Kit,* and *Srgn* [37]gene markers. Cluster 22 expressed *Ccnb2*, *Hmgb2*, *Hmgb3*, *Mcm6*, *Ube2c,* and *Uhrf1* [38] mesenchymal progenitor cell (MPCS) markers. In addition, a recent study [39] suggested that proliferative marker genes *Top2a*, *Mki67,* and *Birc5* were markers for cluster 22 in our study. Neutrophil granulocytes (cluster 12) highly expressed *Adam8*, *Arg2*, *Anxa1,* and *C5ar1* [35]. Endothelial cells (clusters 7 and 8) expressed markers of *Plvap*, *Cav1*, *Cav2*, *Emcn*, *Gpihbp1*, *Pecam1,* and *Tm4sf1* [34]. Myoepithelial cells (cluster 6) expressed gene markers of *Acta2*, *Myh11*, *Myl9*, *Mylk*, *Tpm,* and *De* [40, 41]. T cell (cluster 9) expressed *Rapgef6*, *Ltb*, *Rpl12*, *Rplp1*, *Rps16,* and *Rps23* [35] , and *CD3* gene markers [42-45]. Basal epithelial cells (cluster 13) expressed *Ccnd2*, *Krt14* and *Krt17* markers [46]. Acinar cells/glandular epithelial cells (GEC) (cluster 14) expressed *Cited4*, *Epcam*, *Crabp2,* and *phyh2* gene markers. Smooth muscle cells (cluster 15) highly expressed *Des*, *Myh11*, *Acta2*, and *Tpm2* [40, 47]. In addition, the *PPP1R14A* gene highly expressed in cluster 15 could inhibit the myosin phosphatase, lead to increased phosphorylation of myosin, and enhance smooth

muscle contraction. Cluster 20 highly expressed *SOX1*, *S100* [48], *MPZ* [49], *NCAM* [50-52], *SCN7A* and *CRYAB*. *SCN7A* is one of the many voltage-gated sodium channel proteins. *CRYAB* is highly expressed in many neurological diseases, and the protein encoded by the *S100B* gene might play a role in Ca^{2+} flux stimulation and promoting astrocyte hyperplasia. The chromosomal rearrangement or expression change of S100B is associated with neurological diseases such as Alzheimer's disease, Down's syndrome, and epilepsy. Thus, cluster 20 cells were defined as Swann cells. Cluster 19 cells with highly expressed *Alas2*, *Bpgm,* and *Mkrn1* were identified as erythrocyte precursor cells [34].

Next, we focus on the cell clusters with increased cell abundance in the musk secretion stage (**Supplementary Table S13**), which included cluster 12, cluster 14, cluster 17, and cluster 21. Intriguingly, the DEGs in cluster 14 (**Supplementary Table S14, Supplementary Fig. S6c**) were involved in the "Regulation of hormone levels," "lipid biosynthetic process," and "organic acid transport" pathways, which are related to musk secretion (**Supplementary Fig. S6d**). In addition, for the DEGs in cell cluster 14, we also determined that five genes overlapped with REG, including those associated with carboxylic acid transport (*SLC26A2*), transcription factor activity (*ZNF317*), Cell division (*NCKAP51*) and collagen-containing extracellular matrix (GPC4 and COL6A3). It is worth mentioning that *NCKAP51* is also a REG of forest musk deer. Studies in closely related species have suggested that it targets mir-2425-5p to regulate the proliferation and differentiation of bovine myogenic satellite cells [53]. GPC4 is differentially expressed in dental epithelial and mesenchymal cells [54], also expressed in renal epithelial cells to regulate epithelial branching morphogenesis [55]. COL6A3 encodes collagen type VI and is usually expressed in tumor epithelial cells to promote invasion and metastasis [56]. Two PSGS, cyclin (*WEE1*) and integrin ligand (*NPNT*), coincided with the differential genes of GEC. *WEE1* can regulate cell division by mediating the G2/M phase progression of epithelial cells [57]. *NPNT* was found to be highly expressed in epithelial and mesenchymal cells of the tooth germ and regulated the differentiation of Sox2+ cells in dental epithelial cells through the EGFR-PI3K-Akt signaling pathway [58]. These genes provide evolutionary evidence for the epithelial-mesenchymal transition of the muskrat musk gland during musk secretion and non-secretion.

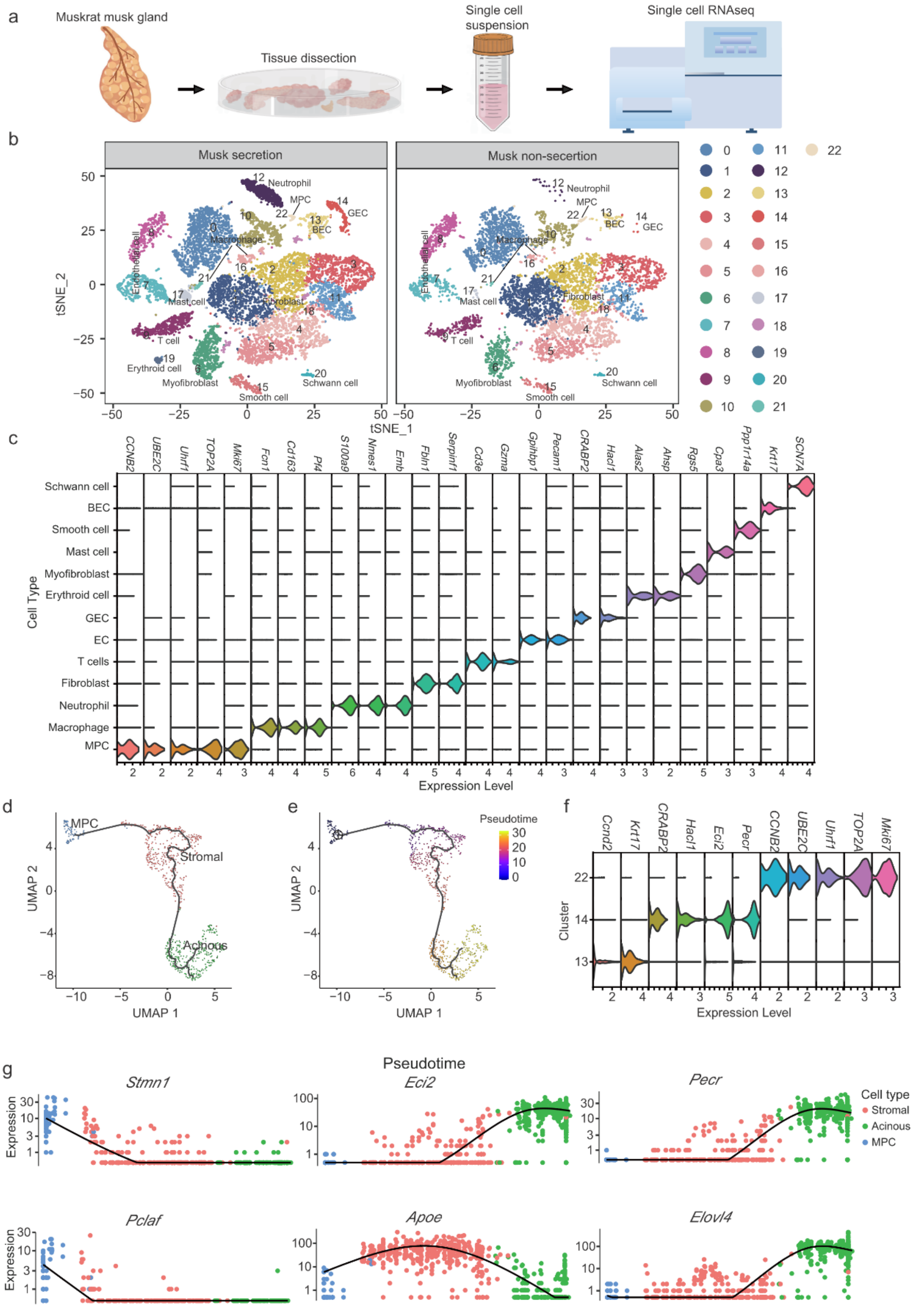

Figure 3: Musk gland single-cell transcriptome map of muskrats. (a) Schematic representation of muskrat musk gland tissue preparation for single-cell transcriptome analysis. (b) tSNE and UMAP cluster map revealing 23 specific clusters representing the major musk gland cell types. (c) Violin plots show the expression of representative differentially expressed genes for each cluster. (d-e). (d) UMAP visualization of the muskrat mesenchymal progenitor cell (cluster 22), basal cell (cluster 13), and acinar cells/glandular epithelial cells (cluster 14). Cells are color-coded by cluster. (e) Cells are color-coded by pseudotime reconstruction traces. (f) Violin plot of genes selected as population

markers for each of the 13, 14, and 22 clusters. (g) Genes that displayed divergent expression patterns during acinar cells/glandular epithelial cells' generation in muskrat.

Pseudotime reconstruction traces of the origin and specification of acinar cells/glandular epithelial cells

Our scRNA results showed that acinar cells/glandular epithelial cells (cluster 14) have an increased number of cells in the musk secretion stage (with a relative abundance of 2.76% and 0.12% in musk secretion and non-secretion stages, respectively), and this cell type is related to musk secretion. Thus, we further explored the origin and differentiation of the cells of this cluster. Acinar cells/glandular epithelial cells (cluster 14) expressed *Cited4*, *Epcam*, *Crabp2,* and *phyh2* gene markers [34]. We used cell lineage trajectory analysis to elucidate the origin and differentiation of the acinar cells/glandular epithelial cells in muskrat. Pseudotime analysis based on transcript profiling enabled a precise reconstruction of acinar cells/glandular epithelial cells in the male muskrat gland.

The high concordance of scRNA status between cluster13 and cluster14 indicated that muskrat acinar cells/glandular epithelial cells derive directly from the basal cells, and the basal cells derive directly from the mesenchymal progenitor cell (**Fig. 3d, e**). This scenario on the biological origin of glandular epithelial cells was consistent with the scRNA of mouse mammary epithelial cells [59]. The markers for acinar cells/glandular epithelial cells (*CRABP2*, *Hacl1*, *Eci2*, and *Pecr*) of cluster 14, found by pseudotime reconstruction, displayed high and specific expressions compared with the other two clusters (**Fig. 3f, g**). These genes were mainly involved in lipometabolic functions. For example, *CRABP2* is involved in the metabolism and transportation of retinoic acid from the cytosol to the RARs (retinoic acid receptors) located in the nucleus [60]. *Hacl1* is an enzyme that catalyzes the hydrolysis of long-chain fatty acids [61]. Enoyl-CoA delta-isomerase 2 (ECI2) is a protein that catalyzes the isomerization of unsaturated fatty acid intermediates during beta-oxidation, a process that breaks down fatty acids to produce energy [62]. Peroxisomal trans-2-enoyl-CoA reductase (PECR) is a protein that plays a role in the metabolism of fatty acids, specifically by reducing unsaturated and polyunsaturated fatty acids to their saturated forms in peroxisomes [63]. A previous study showed that muskrat musk contained fatty acids (29.32%) by gas chromatography-mass spectrometry, which are the main components of musk [64]. Combined, these results indicated that the function of lipometabolism is important in the development and evolution of the musk gland.

Dynamic changes in compartmentalization, TAD, and PEI for musk secretion

In the eukaryotic cell nucleus, genomic DNA is highly folded and spatially organized into a hierarchy of 3D structures, including chromosome territories, compartments, topologically associating domains (TADs), and long-range interactions [65], which play

important roles in transcriptional regulation [66]. To elucidate the multiscale regulatory rewiring of chromatin architecture during musk secretion, we used in situ Hi-C to map chromatin contacts for musk glands between the secretion and non-secretion stages. We generated a total of ~2.77 billion valid contacts (~692.44 million [M] contacts per sample (**Supplementary Table S15**) and reached a maximum resolution of 5 kb by merging the intrachromosomal contacts of the replicates at each stage) (**Supplementary Table S16**). Most (~54.29%) contacts occurred within chromosomes, exhibited high reproducibility among the biological replicates, and consisted dominantly (~57.82%) of long-range interactions (≥20 kb) (**Supplementary Fig. S7a-d**). All samples showed a strong decrease in contact probability with increased distance between loci (**Supplementary Fig. S7e**). All samples showed similar A/B compartment patterns; ~44.4% and ~48.0% of the whole genome were Compartment A bins for musk secretion and non-secretion periods, respectively (**Supplementary Fig. S7f**). Compartment A was positively correlated with Guanine-Cytosine content (Spearman's r > 0.60, $P < 2.20 \times 10^{-16}$) (**Supplementary Fig. S7g-h**) and has a high gene density (**Supplementary Fig. S7i**). We then constructed genome-wide inter-chromosomal contact maps by dividing the genome into 500-kb regions; it was revealed that the muskrat chromosomes have a similar likelihood to mutually contact each other during development: micro- and macrochromosomes tended to be self-associated, small and gene-rich chromosomes preferentially contacted with each other more frequently (**Fig. 4a**). A total of 2,969 and 3,438 TADs were subsequently detected in musk secretion and non-secretion stages in the musk gland, with a median size of ~500 and 575 kb respectively (**Supplementary Fig. S7j**). We observe that only 54% (2,256) of the positioning of TADs remains stable between the two stages (**Supplementary Fig. S7k**). We also compiled an extensive genome-wide catalog of PEIs in musk gland tissue at a 5 kb resolution. The median sizes were ~100 and ~60 kb (54.67% and 63.11% PEIs existed primarily in TADs) for musk secretion and non-secretion stages respectively (**Supplementary Fig. S8a-c**). We observed that ~87.98% of enhancers interacted with a more distant promoter instead of those closer by (**Supplementary Fig. S8d**). This spatial proximity data highlights the complexity of PEIs [67].

Next, we compared the 3D genome differences in the musk gland between the two stages. At the sub-chromosome level, we identified substantial number of regions showing compartmental switching in the musk gland between two stages (~153.6 Mb, or ~6.2% of the genome) (**Fig. 4b**). In these regions, most switching was from A to B (120.80 Mb, embedded with 781 genes), which indicated that these regions were more closed in the musk secretion stage compared with the non-secretion stage. The rest were transient switches, from B to A (32.8 Mb, embedded with 164 genes) (**Fig. 4b, c**). Because these active chromatin regions are of potential functional significance, we further checked the genes in areas subject to B-to-A switching events. They were primarily involved in "epithelial cell differentiation," "nephron development," "epidermis development," "cytoplasmic translation," "extracellular matrix organization," "negative regulation of endopeptidase activity," "cell morphogenesis," "protein activation cascade," "epithelial cell development," "intracellular steroid

hormone receptor signaling pathway" and "regulation of membrane potential" processes (**Fig. 4d**).

At the TAD structure level, many changes in chromatin structure occur. We used the insulation score (IS) to evaluate the overall extent of changes in chromatin conformation between the two stages. For the TAD with increased IS in the musk secretion stage, the content genes were involved in the "Regulation of proteolysis," "Response to radiation," "Regulation of lipid metabolic process," and "Transcription coregulator activity" pathways (**Supplementary Fig. S9a**), which indicated that the active function of regulation of lipid metabolism in the musk secretion stage. Furthermore, the PSG or convergent evolution genes (**Supplementary Fig. S9b**) involved in TAD changing were also related to the metabolism of lipids and epithelial regulation pathways, such as *NAGS* catalyzing the production of N-acetylglutamate (NAG). This vital substance regulates urea synthesis [68]. *CEP250* plays a crucial role in differentiating spermatogonia and meiotic spermatocytes [69]. *CASP8AP2* plays a role in regulating cell proliferation, apoptosis, and gene expression [70].

At the PEI (promoter-enhancer interaction) level, we determined that there were two genes (*SMPDL3A* and *NRCAM*) with specific PEI in musk secretion stage, also showed a compartment transition from B to A and was a marker gene in Cluster 14 (**Fig. 4e**). There were more specific long-range interactions (> 25Kb) in musk secretion stage for these two genes (**Fig. 4f**). The gene *SMPDL3A* showed a relative higher expression in all clusters at the musk secretion stage (**Fig. 4g**). SMPDL3A (sphingomyelin phosphodiesterase acid-like 3A) is an enzyme induced by lipid metabolism through liver X receptor that degrades cGAMP, modulating the cGAS-STING pathway which is involved in immune responses and lipid sensing [71]. NRCAM (neuronal cell adhesion molecule) primarily associated with neural development, its expression also promotes malignant cell transformation, cell motility, and metastatic disease [72]. In addition, *MAP3K1*, *NODAL,* and *Slc38a2* also showed significantly more PEIs and were contacted with more enhancers during the musk secretion stage (**Supplementary Fig. S10**). MAP3K1 is a critical component of the protein kinase signal transduction cascade and plays a crucial role in cellular signaling pathways [73]. *NODAL* maintains stem cell pluripotency and promotes directed differentiation [74]. *Slc38a2* encodes an amino acid transport protein facilitating cellular uptake of amino acids [75]. These results suggest that two essential functions of the musk gland during the musk secretion stage are lipid metabolism and cell specialization, which indicate that synthesis and secretion activity were very active at this stage. Our results demonstrated that candidate loci can be analyzed in future studies of musk secretion mechanisms.

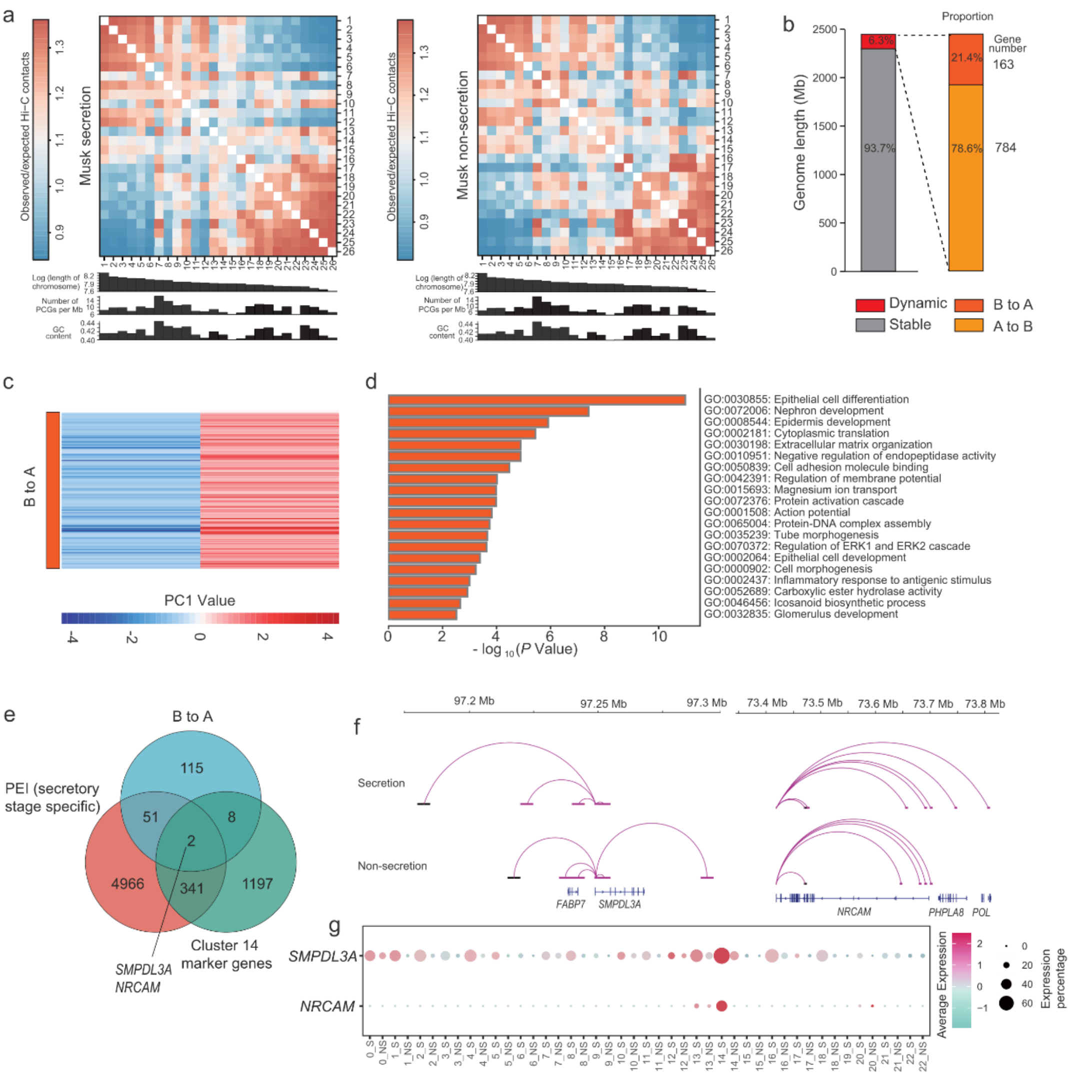

Figure 4: Global chromatin interaction patterns in musk gland of musk secretion and non-secretion stages. (a) Observed/expected contact matrices between chromosome pairs. Also shown are the length, gene density, and GC content of each chromosome. (b) Genomic lengths and proportions of stable and dynamic compartments. Dynamic compartments are classified into two types of transitions (A to B, and B to A). (c) Heatmap of the PC1 values for the compartment B to A switching regions. (d) The most enriched GO-BP terms for genes within B to A switch regions. (e) Veen diagram showing overlapping genes with compartment B to A, PEI specific to musk secretion stage, and marker genes in Cluster 14. (f) Promoter-enhancer interactions (PEIs) rewired in the musk gland of musk secretion and non-secretion stages of *SMPDL3*A and *NRCAM*. (g) Gene expression of *SMPDL3*A and *NRCAM* in each cluster at musk secretion and non-secretion stages.

The MuskDB platform

According to the sequencing data described above, we generated a database (MuskDB). MuskDB contains 47,635 gene entries from the genomes of two species, with information on 831 biological pathways,103 bulk RNA transcriptomes, two single-cell transcriptomes, and three Hi-C datasets. The platform also holds three Hi-C data sets (secretion stage in July and non-secretion stage in October for the muskrat musk gland

and blood sample of musk deer), including 343,392 promoter and enhancer interactions. On the homepage, the tools include “Blast,” “Sequence Fetch,” “Gene Sequence Extraction,” “Transposable Elements,” “Gene Synteny Viewer,” “Phylogenetic Tree,” “Gene Expression,” “Single Cell Expression” and “Hic Search” (**Fig. 5a**). MuskDB provides comprehensive information on muskrat and musk deer genes, including their annotation, location and expression. The heat maps in ‘Gene Expression’ and ‘Single Cell Expression’ show the expression of genes respectively in 13 tissues (**Fig. 5b**) and 13 different cell types (**Fig. 5c**). In ‘Hic Search,’ users could enter a gene name or genome region to show the contacts information of this gene or in this region. For example, when gene *Synpo2* is entered, the results showed the contact profile of this gene in two stages of secretion and non-secretion (**Fig. 5d**).

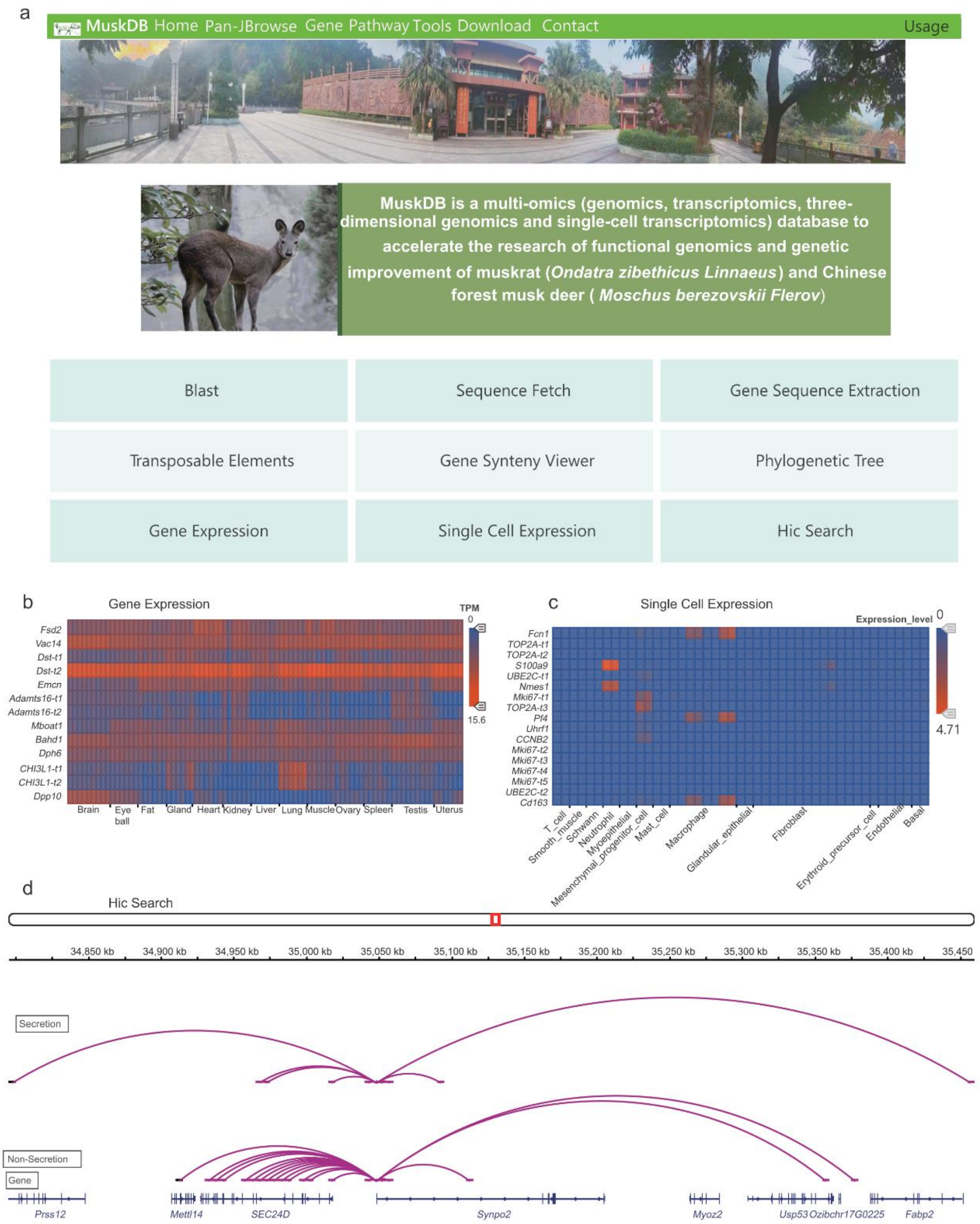

Figure 5: Overview of MuskDB and its application in musk secretion animal's functional genomics. (a) MuskDB homepage contents show available online tools. (b) The expression pattern of genes in different tissues is shown by MuskDB. (c) The expression pattern of genes in various cell clusters is shown by MuskDB. (d) Hi-C contacts of gene *Synpo2* in musk secretion and non-secretion stage shown by MuskDB.

Conclusion

This study provided an open-source, web-accessible, user-friendly multi-omics database platform of musk secretion animals, including two high-quality genome

assemblies of musk secretion species (muskrat and musk deer), a BodyMap transcriptome of muskrat, and comprehensively analyzes Hi-C, RNA-seq, and scRNA-seq between musk secretion and non-secretion stages of muskrat *in vivo*. GO terms of stage-specific signature genes of musk glands were identified by scRNA-seq and emphasized significant functional differences between the musk secretion and non-secretion stages. Because of their unique evolutionary adaptations, the muskrat and musk deer have always been a topic of interest in animal evolution and physiology. Our high-quality genome assembly provides more precise comparative insights into the genetic basis of their biological features. We identified genetic changes underlying adaptations of both species to the musk secretion, notably related to the adaptation of lipid metabolism, cell cycle regulation, and sensory perception. Our single-cell RNA sequencing results may potentially facilitate the development of new strategies for the organoid culturing of the musk gland. These results show that active lipid metabolism may underlie the adaptation evolution of musk secretion. However, because of the complexity of the musk gland, more research on the functional consequences of musk secretion-specific genetic variants is perspectively needed.

Materials and methods

Methods

Sample collection

For genome sequencing, blood from a male muskrat and a male forest musk deer were collected from the Chongqing Institute of Medicinal Plant Cultivation, following the ethical guidelines for Animal Care and Use of Chongqing University of Chinese Medicine. To fully investigate the muskrat transcriptome, we used 84 samples from 13 tissues (3 to 6 samples from each of the 13 tissues were collected). The healthy muskrats were isoflurane euthanasia; we adjusted the isoflurane flow rate or concentration to 5% or greater and continued isoflurane exposure until one minute after the muskrat's breathing stopped. The required tissues were then collected using standard anatomical techniques, and excess blood vessels and fat were removed in phosphate buffer solution and then flash-frozen in liquid nitrogen and stored in a -80°C refrigerator for subsequent studies.

Genome sequencing

Blood high-quality DNA was extracted from 20 mL of muskrat and forest musk deer blood using a DNeasy Blood & Tissue Kit (Qiagen, Valencia, USA) according to the manufacturer's instructions. Some of this DNA was then used to construct Nanopore libraries, which were sequenced using GridION X5 sequencers (Oxford Nanopore Technologies, Oxford, UK), and the remainder of the DNA was used to construct re-sequencing (NGS) libraries with an insert size of 400 bp, which were sequenced using the BGI T7 platform. The musk gland samples at musk secretion and non-secretion

stages were used to construct Hi-C libraries, which were subsequently sequenced using the Illumina NovaSeq platform (Illumina).

Genome assembly and assessment

We used an optimized four-step genome assembly strategy [8] to generate complete assemblies of muskrat and musk deer genomes. First, we used the high-quality Nanopore sequences and applied a 'correct-then-assemble' strategy using NextDenovo (v2.5.0; https://github.com/Nextomics/NextDenovo) to assemble the initial contigs. To correct the initial contigs, we utilized the software NextPolish (v1.4.1), employing high-quality T7 paired-end reads and Nanopore long reads, and applied the recommended algorithm modules "best." in Nextpolish [76]. The specific parameters and pipelines used in the assembly process are available at Zenodo (https://doi.org/10.5281/zenodo.13690583). Thus, we yielded the ContigV1 assembly. Second, we generated unique mapped pairs by aligning the Hi-C read pairs to ContigV1 using Bowtie2 software [77] with a single-ended model. We then discarded invalid self-ligated and unligated fragments using the HiCUP pipeline (version 0.8.0) [78]. We obtained the valid interaction pairs and calculated linkage frequency among all contigs using an agglomerative hierarchical clustering algorithm. The linked contigs were clustered based on the Hi-C signal density, indicating potential homologous chromosome associations. Third, we realigned the Nanopore reads to ContigV1 using package Minimap2 [79]. Suboptimal alignment reads were removed, and mapped reads of each contig group were extracted. Local assembly was performed for each classified mapped read to avoid false overlap relationships caused by repetitive sequences during assembly [80]. All contigs were corrected again using appropriate parameters, similar to the first step. Fourth, chromosome-scale genomes were anchored using linkage information, restriction enzyme site, and string graph formulation with the ALLHiC algorithm [81]. Any placement and orientation errors displaying distinct chromatin interaction patterns were manually adjusted.

To assess the quality of genome assemblies, we used the Merqury package to evaluate assembly precision by measuring QV values [10]. BUSCO analysis (version 5.2.1) [9] was used to assess the assembly completeness by searching against 9,226 conserved mammalian genes from the mammalia_odb10 database. Additionally, T7 paired-end reads were aligned to the assembled genome using BWA software [82] to calculate the alignment ratio and coverage depth, for assessing the assembly completeness.

Transposable element (TE) annotation

We predicted the genome TEs by combining homology searching and *ab initio* prediction methods. We performed the homology searching by applying the RepeatMasker [83] and RepeatProteinMask packages to compare the genome against the Repbase TE library. In parallel, we performed *ab initio* prediction by constructing

a reference repeat library using the results from PILER [84], LTR FINDER [85], and RepeatScout [86]. Then, we searched the genome against this library using RepeatMasker. In addition, we employed the Tandem Repeats Finder package [87] with specific parameters ("2 7 7 80 10 50 2000 -d –h") to predict tandem repeats in the genome.

Protein-coding gene prediction

We predicted protein-coding gene models via integration of homology- and *ab initio*-based methods, with additional evidence from transcription data. In the homology-based approach, we used protein repertoires from model mammalian species such as *Homo sapiens* (GCA_000001405.28), *Mus musculus* (GCA_000001635.8), *Equus caballus* (GCA_002863925.1), *Canis lupus familiaris* (GCA_000002285.2), *Rattus norvegicus* (GCA_015227675.2), *Sus scrofa* (GCA_000003025.6) and *Bos taurus* (GCA_002263795.2) were used as queries. These queries were searched against the target genome using the TBLASTN algorithm [88]. The resulting BLAST hits were conjoined using the Solar (Sorting Out Local Alignment Results) to obtain a comprehensive set of alignments. Next, the gene structures within each BLAST hit were determined using the GeneWise pipeline [89], allowing us to define gene models with high accuracy and specificity. Subsequently, we obtained transcriptomic data for muskrats and musk deer from the NCBI database, with accession numbers listed in **Supplementary Table S1**. The RNA-seq data were aligned to the genome using the Tophat software [90], enabling the identification of potential exonic regions and splicing junctions. Gene models, represented by the Cufflinks-set, were assembled from the mapped reads using Cufflinks [91]. In the *ab initio* method, we aligned the assembled transcripts with the assembled genome using the Program to Assemble Spliced Alignment (PASA) [92]. This allowed us to assemble the transcript alignments into gene structure models used as the training set for Augustus [93], SNAP [94], and GlimmerHMM [95] pipelines. With these training sets, we conducted ab initio prediction of coding regions in the repeat-masked genome using Augustus, GlimmerHMM, and SNAP. Furthermore, we employed GeneID [96] and GeneScan [97] to directly generate predicted gene models in the repeat-masked genome. After completing the aforementioned methods, the generated gene models were integrated using EVidenceModeler. We assigned weights to each type of evidence as follows: Homology-set > Cufflinks-set > Augustus > GeneID = SNAP = GlimmerHMM = GeneScan. Furthermore, we used PASA2 to update the gene models, incorporating untranslated regions and information on alternative splicing variations.

To annotate the protein-coding genes, we searched for functional motifs, domains, and information on the possible biological processes of the genes in established databases such as SwissProt [98], the NR database (from NCBI), and the KEGG (Kyoto Encyclopedia of Genes and Genomes) [99].

Identification of one-to-one orthologous genes

In addition to the gene sets generated from our two assembled genomes, we also downloaded gene sets from 10 other mammalian genomes, including human, macaque, mouse, horse, cattle, sheep, pig, cat, dog, and rabbit, from the Ensembl database. We used these 12 gene sets to identify orthologous genes. To accomplish this, we first selected the longest translation to represent each gene and filtered our genes with fewer than 50 amino acids. Then, we performed an all-against-all BLASTP comparison with an E-value threshold of 1e-7 to determine similarities between genes across the 12 species. We extracted alignment pairs from each pair of genomes while restricting a maximum of five hits per protein sequence. These alignment pairs were used as input for the MCScanX algorithm [100], which helped detect collinear blocks of coding genes and identify orthologous gene pairs with high confidence. We specifically focused on one-to-one orthologous genes between pairs of mammalian species.

After integrating a matrix of orthologous genes for the 12 mammalian species, we ensured each orthologous cluster included all the species. Subsequently, we performed multiple sequence alignment for these one-to-one orthologs using PRANK (v.170427) [101] . We applied the Gblocks package (v0.91b) [102] to minimize the impact of alignment errors and divergent regions. Alignments shorter than 90 nucleotides were discarded to maintain quality. Through this process, we identified 7,409 one-to-one orthologs among the 12 species.

Phylogeny construction and divergence time estimation

We initially used MODELTEST [103] to analyze the codon alignments of one-to-one orthologs and determined that the general time-reversible (GTR) substitution model was the most suitable for the observed data. Therefore, we conducted a phylogenetic tree for the 12 mammals using the maximum likelihood method implemented in the RaxML package [104]. The best-fitting substitution model "GTR+GAMMAX" was utilized, and 1,000 bootstrap replicates were performed to assess the robustness of the tree topology. To estimate divergence times, we employed the MCMCTree program from the PAML package (version 4.9) [12].

Gene family clustering, expansion, and contraction analysis

We utilized the OrthoFinder package (v2.3.1) [105] to identify gene families by detecting orthogroups and paralogous genes based on the results of the all-against-all BLASTP analysis (see also the method "Identification of one-to-one orthologous genes"). The expansion and contraction of gene families were evaluated by comparing cluster sizes between the ancestral species and each of the 12 mammalian species. This analysis used the Café program [106], which employs a probabilistic graphical model. Using conditional likelihoods as test statistics, we calculated *P-values* for each lineage and set a threshold of $P < 0.05$ to determine gene families that exhibited significant expansion or contraction.

Identification of positively selected genes (PSGs) and rapidly evolving genes (REGs)

We employed the CodeML program in the PAML package (version 4.9) [12] to identify PSGs and REGs based on the 7,409 orthologous genes. For PSGs, we utilized the free-ratio branch-site mode (model = 1) as an alternative model, assuming positive selection on the foreground branch. The null model allowed sites to undergo purifying selection or evolve neutrally. For REGs, we utilized the branch model, specifically the one-ratio model (model = 0) as the null model assuming the same evolutionary rate for all branches, and the two-ratio model (model = 2) as an alternative model allowing different evolutionary rates for the foreground branch. The likelihood ratio test (LRT) method was used to detect differences between the nested models, and *P-values* were computed based on $\chi 2$ statistics. Multiple testing was corrected using the false discovery rate (FDR) method.

Convergent evolution among musk secretion animals

To test for convergence among musk secretion animals (muskrat and musk deer), we used 12 mammals from Fig. 1b. Based on the phylogenetic tree, we used two methods to detect the convergent amino acid substitutions for each node of the 7,409 single-copy orthologs: 1) method of Zhang and Kumar [13, 14], a site was assumed as a convergent site if amino acids of a focused node at that site are the same but different with their most recent ancestral amino acids. Amino acid sequences of internal nodes for all the 7,409 single-copy orthologs were reconstructed by CODEML in PAML. For each gene, the number of observed convergent site was compared with the neutral expectations derived from the JTT-fgene model, and the Poisson test was then used to evaluate the difference. 2) CCS method [15], the convergent signal is identified when all two musk secretion species (muskrat and musk deer) share the same derived character at a conservative site.

Functional enrichment analysis

The gene set enrichment analyses, including Gene Ontology (GO), KEGG pathway, and Reactome analyses, were performed using the KOBAS 3.0 software [107, 108], with human homologs as references. The statistical significance of enrichment was assessed using the binomial distribution test, and the P-values were adjusted for multiple testing using the Benjamini method.

Muskrat transcriptome reconstruction

Total RNA was extracted from each sample using RNAiso Plus reagent (TaKaRa, #9108) according to the manufacturer's instructions. We estimated the integrity and quality of the total RNA using a Bioanalyzer 2100 system (Agilent Technologies, Palo Alto, CA, USA) and an RNA 6000 Nano kit. Eighty-four poly-A RNA-seq libraries were constructed. LncRNAs were then sequenced using the NOVAseq-6000 platform with a paired-end sequencing length of 150 bp (PE150) at Shenggong Bioengineering

Co., LTD (Shanghai, China). MicroRNA was sequenced by the NEXTSEQ550 platform with a single-end sequencing length of 75 bp (SE75) at Shenggong Bioengineering Co., LTD (Shanghai, China). In total, we then generated a total of 1.37 Tb high-quality RNA-seq data (~16.33 Gb sequences per sample) and 27.13 Gb high-quality miRNA data (~0.32 Gb sequences per sample) (**Supplementary Table S1**).

Clean reads were obtained after quality control filtering. Using Fast - toolkit software (http://hannonlab.cshl.edu/fastx_toolkit/) to remove the clean and low-quality reads (quality value less than 30 bases accounted for more than 20%). The lncRNA sequenced reads were aligned to our self-assembled muskrat genome by the STAR alignment tool (version 2.6.0), with, on average, ~97% (~57.06 million) of aligned reads for each library. Read Counts were quantified using feature Counts (version 2.0.1). Gene-level transcript abundance was estimated as transcripts per million (TPM).

For miRNA analysis, human, mouse, and rat miRNA and miRNA precursor sequences were downloaded from miRBase (Version 22.1). The software miRdeep2 (version 0.1.2) was used to first index the muskrat genome, and then the data we had initially processed were converted into the format required by the software and compared with the muskrat genome. Finally, the sequences were compared with known miRNA sequences and miRNA precursor sequences. Combined with the position of genome alignment, the matching degree was calculated to form the minimum free energy of stem-ring miRNA, to identify whether it is a muskrat miRNA. The prediction of new miRNA is similar. Mature miRNAs of muskrat relatives, such as humans, mice, and rats, were selected as a reference, and mirdeep2.pl of the software was used for prediction. The screening criteria for new miRNA should meet the miRDeep2 Score25 and have a secondary structure with p-value < 0.05 as candidate miRNA. Then, the information of new miRNA predicted by all samples should be counted, and the candidate new miRNA predicted by at least two samples should be considered new miRNA. The number of miRNA reads between samples was counted by TPM (Tags per million), and its expression was calculated.

Gene transcriptional profiling across tissues

We calculated the tissue specificity of gene abundance reflected by the tau score (τ) (ranging from 0 to 1, with 1 for highly tissue-specific genes and 0 for ubiquitously transcribed genes) for each gene with scaled TPM values [109]. For each tissue, we averaged all replicates and then calculated τ to account for unequal numbers of replicates among tissues. We used $\tau \geq 0.75$ as the cut-off for tissue-specific genes. We calculated the abundance distribution (i.e., transcriptome complexity) of distinct transcripts across tissues, reflected as the fraction of total RNAs contributed by the most highly expressed genes. Differential gene expression analysis was performed using edgeR (version 3.40.2) [110], with a Benjamini &. Hochberg adjusted P value $\leq$0.01 and $\log_2$(fold change) $\geq$1 as cut-offs for statistical significance.

Single-cell preparation

After harvesting, musk gland tissues were washed in ice-cold RPMI1640 and dissociated using Demonstrated_Protocol_Adult_Mouse_Nuclei_Isolation_RevA. (10× Genomics Catalog No.CG000393 Rev A) from Miltenyi Biotec as instructions. DNase treatment was optional according to the viscosity of the homogenate. Cell count and viability were estimated using a fluorescence Cell Analyzer (Countstar® Rigel S2) with AO/PI reagent after the removal of erythrocytes (Miltenyi 130-094-183). Then, debris and dead cell removal was performed or not (Miltenyi 130-109-398/130-090-101). Finally, fresh cells were washed twice in the RPMI1640 and then resuspended at 1×10^6 cells per mL in 1×PBS and 0.04% bovine serum albumin.

Single-cell RNA-seq library construction and sequencing

ScRNA libraries were prepared using Chromium Next GEM Single Cell 3′ Reagent Kits v3.1 (10× Genomics). Briefly, the appropriate number of cells were mixed with reverse transcription reagent and then loaded to the sample well in Chromium Next GEM Chip G. Subsequently, Gel Beads and Partitioning Oil were dispensed into corresponding wells separately in the chip. After emulsion droplet generation, reverse transcription was performed at 53 °C for 45 minutes and inactivated at 85 °C for 5 minutes. Next, cDNA was purified from a broken droplet and amplified in the PCR reaction. The amplified cDNA product was then cleaned, fragmented, end-repaired, A-tailed, and ligated to the sequencing adaptor. Finally, the indexed PCR was performed to amplify the DNA representing the 3' polyA part of expressing genes, which also contained a Cell Bar code and a Unique Molecular Index. The indexed sequencing libraries were cleaned with SPRI beads, quantified by quantitative PCR (KAPA Biosystems KK4824), and then sequenced on Illumina NovaSeq 6000 with PE150 read length.

Single-cell RNA sequencing data quality control

Fastp (v0.20.1) [111] was used to trim primer sequence and low-quality bases of raw reads and collect the basic statistics. The specific parameters could be summarized as below: (1) A 4 bp sliding window was moved from the front (5') to tail. Once the mean quality of the bases in the window was below 10, the bases, along with the subsequent bases, would be dropped, the leading N bases were also trimmed (--cut_front --cut_front_window_size 4 --cut_front_mean_quality 10); (2) A 1 bp sliding window was moved from tail (3') to front. The bases in the window were dropped if its mean quality was below 3, the trailing N bases were also trimmed, similar to the Trimmomatic TRAILING method (--cut_tail --cut_tail_window_size 1--cut_tail_mean_quality 3); (3) The auto adapter was detected for PE data (detect_adapter_for_pe); (4) The trimmed Reads shorter than 60 bp were discarded (--length_required 60). The cleaned reads after trimming were used in the following steps.

Processing the single-cell RNA sequencing data

The Cell Ranger [112] Single-Cell Software Suite performed sample demultiplexing, barcode processing, and single-cell 3' gene counting (http://software.10xgenomics.com/single-cell/overview/welcome). The single-cell data from the two stages were analyzed jointly.

The clustering and visualization were finished by Seurat [113], with the following steps: (1) Data normalization. LogNormalize, a global-scaling normalization method, was employed to normalize the expression. The expression measurement of one transcript was divided by those of all the transcripts of the cell and multiplied by a scale factor (10,000 by default), and then the result was logarithmically transformed. (2) Detection of highly variable features. FindVariableFeatures was used to get 2,000 features per dataset. (3) Scaling. A linear transformation ('scaling'), a standard pre-processing step before dimensional reduction techniques, was applied. (4) Dimensional reduction. PCA on the scaled data was performed, and the first 15 principal components were used in the following steps. (5) Clustering. A graph-based approach was applied to cluster the cells. (6) tSNE/UMAP. The non-linear dimensional reduction technique was used to visualize and explore these datasets. (7) Cluster markers. FindAllMarkers with the default parameters except "logfc.threshold=1" was used to find markers that determined the cell clusters via the differential expression, and the top 9 markers were visualized.

Pseudotime analysis

In R, utilizing the Monocle3 software package v1.3.1 (Cao et al., 2019), pseudotime trajectories for all 13, 14, and 22 subpopulations in the musk gland were constructed. This technique sorts individual cells along their developmental paths based on how closely their gene expression patterns match those of other sequenced cells, effectively mapping out the dynamics of cellular changes [114, 115]. Furthermore, the method evaluates how genes work together in space by using Moran's Index to measure their co-expression, which helps to establish a timeline of gene expression changes.

Identification of differentially expressed genes (DEGs) in Cluster 14 cells

DEGs in the Cluster 14 cells of musk secretion and non-secretion stages were identified using the FindMarkers function in Seurat. Genes meeting the thresholds ($|\log_2 FC| > 1$, P-adjusted < 0.05, min.pct = 0.25) were considered as DEGs. The results were visualized using dot plots.

Hi-C data of musk gland tissue

Hi-C data processing

We utilized Juicer, an efficient open-source tool [116], to process the Hi-C datasets. Initially, we aligned the high-quality Hi-C reads to the genome using the BWA-mem module. Subsequently, we eliminated abnormal, duplicate, and low-quality alignments (MAPQ < 30). We constructed a normalized contact matrix using the KR algorithm at different resolutions, including 5Kb, 25Kb, 100Kb, 500Kb, and 1Mb.

Resolution evaluation of the Hi-C matrix

To determine the optimal resolution for our Hi-C matrix, we divided the genome into window sizes ranging from 1Kb to 1Mb. For each bin, we counted the number of *cis* contacts, defined as any contact where one read mapped within that bin, and calculated the percentage of bins with contacts greater than 1000. We identified the minimum window size with a percentage greater than 80 as the optimal resolution for our Hi-C matrix.

Identification of compartment A/B at the resolution of 100 Kb and 25 Kb

At a resolution of 100 Kb, compartment A/B analysis was performed as previously described. Briefly, a Pearson correlation matrix was generated using the 'cor' function in R. The first three principal components were obtained by applying the 'prcomp' function in R to the correlation matrix. Bins at 100 Kb with positive Spearman's correlation between PC1 values and gene density were classified as compartment A, while bins with negative correlation were classified as compartment B.

For compartment A/B identification at a resolution of 25 Kb, the A-B index value was used as previously described, representing the comparative likelihood of a sequence interacting with A or B at a resolution of 100 Kb. Bins at 25 Kb with positive values (indicating a greater association with A at 100 Kb) were identified as A compartments, while bins with negative values (indicating a greater association with B at 100 Kb) were identified as B compartments.

Identification of inter-chromosome interaction pattern

The patterns of inter-chromosome interactions were calculated following the previous protocol [117]. In brief, the observed number of contacts was normalized against the expected contacts in each inter-chromosome pair.

Identification of TAD

We identified TADs from the normalized contact matrix at a resolution of 25 Kb. We used the directionality index (DI) score and a Hidden Markov Model (HMM) algorithm implemented in the TADtool software with default parameters [118] to assign TAD boundaries.

Promoter-enhancer interaction (PEI) analysis

We first combined the clean data of biological replicates and constructed the normalized contact matrix at 5 Kb resolution. We then used PSYCHIC software to generate raw PEIs [119]. We then filtered low-confidence PEIs with interaction distances lower than 10 Kb or FDR greater than 0.001.

Ethics Statement

All animal research was conducted according to the Regulations for the Administration of Affairs Concerning Experimental Animals (Ministry of Science and Technology, China, revised in March 2017) and approved by the Animal Ethical and Welfare Committee (AEWC) of Chengdu University under permit No. YXY-2022630272.

Competing Interests

The authors declare that they have no conflict of interest.

Funding

This research was funded by the National Natural Science Foundation of China (81973428, 82274046 to H.J., and 32272859 to Z.Y.), the Fundamental Research Funds of Chongqing (2022JK017) to H.J., and the Beijing Nova Program (Z211100002121022 and 20230484446) to S.T.

Additional Files

Supplementary Fig. S1

Karyotype and genome assembly of muskrats.

Supplementary Fig. S2

Terms for expanded and contracted genes in muskrat and musk deer

Supplementary Fig. S3

Sequence alignment highlighting sites that evolved in *DST*, *CKAP5*, and *NoP2*.

Supplementary Fig. S4

Gene expression from 13 tissues in muskrat.

Supplementary Fig. S5

Significantly enriched GO terms in tissue-specific expressed genes for each tissue. Tissue specificity of gene abundance was reflected by the tau score (τ).

Supplementary Fig. S6

Transcriptome map of muskrat analyzed.

Supplementary Fig. S7

Hi-C data quality and global chromatin interaction patterns during two representative musk gland stages.

Supplementary Fig. S8

Basic features of PEIs.

Supplementary Fig. S9

PSGs (REGs) involved in cell cycle and sequence alignment highlighting sites that evolved in parallel in *TEX15*, *Casp8ap2*, *NAGS*, and *Cep250*.

Supplementary Fig. S10

Promoter-enhancer interactions (PEIs) rewired in the musk gland of musk secretion and non-secretion stages.

Supplementary Table S1

Sequencing data summary.

Supplementary Table S2

Summary for the chromosome-scale genomes of muskrat and musk.

Supplementary Table S3

Overview of chromosome-scale genomes of muskrat and musk deer.

Supplementary Table S4

Summary of assembly quality evaluation.

Supplementary Table S5

Summary of the TEs and statistics of gene structure in muskrat and musk deer genomes.

Supplementary Table S6

PSGs, REGs and convergent evolution gene in muskrat and muskdeer genomes.

Supplementary Table S7

The significantly enriched terms for expanded genes in muskrat and musk deer.

Supplementary Table S8

The significantly enriched terms for contracted genes in muskrat and musk deer.

Supplementary Table S9

The significantly enriched terms for rapid evolution genes in muskrat.

Supplementary Table S10

The significantly enriched terms for positive selected and rapid evolution genes in musk deer.

Supplementary Table S11

Tissue-specific genes in each tissue.

Supplementary Table S12

Marker genes of each cell type.

Supplementary Table S13

Number of cells of each cell type in two musk secretion stages.

Supplementary Table S14

DEGs in comparison between musk secretion and non-secretion stages detected in cluster 14.

Supplementary Table S15

Hi-C data summary.

Supplementary Table S16

Hi-C matrix resolution evaluation by Cis contacts.

Authors' Contributions

Tao Wang: Writing - Original Draft, Writing - Review & Editing. **Maosen Yang:** Formal analysis, Software, Visualization. **Xin Shi:** Formal analysis, Data Curation, Visualization. **Shilin Tian:** Formal analysis, Funding acquisition, Software. **Yan Li:** Formal analysis, Methodology. **Wenqian Xie:** Formal analysis. **Zhengting Zou:** Review & Editing. **Dong Leng:** Formal analysis, Visualization. **Ming Zhang:** Data Curation, Resources. **Chengli Zheng:** Data Curation, Resources. **Chungang Feng:** Writing - Review & Editing. **Bo Zeng:** Investigation. **Xiaolan Fan:** Data Curation, Resources. **Huimin Qiu:** Formal analysis. **Jing Li:** Software. **Guijun Zhao:** Supervision. **Zhengrong Yuan:** Funding acquisition, Writing - Review & Editing. **Diyan Li:** Conceptualization, Methodology, Validation, Writing - Original Draft. **Hang Jie:** Funding acquisition, Writing - Review & Editing, Resources.

References

1. He, L., et al., *Effects of crowding and sex on fecal cortisol levels of captive forest musk deer.* Biol Res, 2014. **47**(1): p. 48.
2. Lv, S., et al., *Chemical compositions and pharmacological activities of natural musk (Moschus) and artificial musk: A review.* Journal of Ethnopharmacology, 2022. **284**: p. 114799.
3. Mychajliw, A.M. and R.G. Harrison, *Genetics reveal the origin and timing of a cryptic insular introduction of muskrats in North America.* PLoS One, 2014. **9**(10): p. e111856.
4. Sokolov, V.E., et al., *Musk deer (Moschus moschiferus): Reinvestigation of main lipid components from preputial gland secretion.* J Chem Ecol, 1987. **13**(1): p. 71-83.
5. Li, D., et al., *The musk chemical composition and microbiota of Chinese forest musk deer males.* Sci Rep, 2016. **6**: p. 18975.
6. Li, Y., et al., *Comparison of amino acid profiles and metabolic gene expression in muskrat scented glands in secretion and non-secretion season.* Sci Rep, 2017. **7**: p. 41158.
7. Shi, X., et al., *Correlation Analysis between Muskrat (Ondatra zibethicus) Musk and Traditional Musk.* Animals (Basel), 2023. **13**(10).
8. Tian, S., et al., *Comparative analyses of bat genomes identify distinct evolution of immunity in Old World fruit bats.* Sci Adv, 2023. **9**(18): p. eadd0141.
9. Simao, F.A., et al., *BUSCO: assessing genome assembly and annotation completeness with single-copy orthologs.* Bioinformatics, 2015. **31**(19): p. 3210-2.
10. Rhie, A., et al., *Merqury: reference-free quality, completeness, and phasing assessment for genome assemblies.* Genome Biol, 2020. **21**(1): p. 245.
11. Editorial, N.B., *A reference standard for genome biology.* Nat Biotechnol, 2018. **36**(12): p. 1121.
12. Yang, Z., *PAML 4: phylogenetic analysis by maximum likelihood.* Mol Biol Evol, 2007. **24**(8): p. 1586-91.
13. Zhang, J. and S. Kumar, *Detection of convergent and parallel evolution at the amino acid sequence level.* Molecular Biology and Evolution, 1997. **14**(5): p. 527-536.
14. Zou, Z. and J. Zhang, *Are Convergent and Parallel Amino Acid Substitutions in Protein Evolution More Prevalent Than Neutral Expectations?* Molecular Biology and Evolution, 2015. **32**(8): p. 2085-2096.
15. Xu, S., et al., *Genome-Wide Convergence during Evolution of Mangroves from Woody Plants.* Mol Biol Evol, 2017. **34**(4): p. 1008-1015.
16. Nummela, S., et al., *Exploring the mammalian sensory space: co-operations and trade-offs among senses.* J Comp Physiol A Neuroethol Sens Neural Behav Physiol, 2013. **199**(12): p. 1077-92.
17. Liu, C., et al., *A towering genome: Experimentally validated adaptations to high blood pressure and extreme stature in the giraffe.* Sci Adv, 2021. **7**(12).
18. Yang, F., et al., *TEX15 associates with MILI and silences transposable elements in male germ cells.* Genes Dev, 2020. **34**(11-12): p. 745-750.
19. Chan, J.Y., et al., *Whole exome sequencing identifies recessive germline mutations in FAM160A1 in familial NK/T cell lymphoma.* Blood Cancer Journal, 2018. **8**(11): p. 111.
20. Sabo, J., et al., *CKAP5 enables formation of persistent actin bundles templated by dynamically instable microtubules.* Current Biology, 2024. **34**(2): p. 260-272.e7.
21. Luo, Q., et al., *Role of ACSL5 in fatty acid metabolism.* Heliyon, 2023. **9**(2): p. e13316.

22. Polinski, N.K., et al., *Decreased glucocerebrosidase activity and substrate accumulation of glycosphingolipids in a novel GBA1 D409V knock-in mouse model.* PLoS One, 2021. **16**(6): p. e0252325.
23. Hirai, K., et al., *Molecular and Functional Analysis of Choline Transporters and Antitumor Effects of Choline Transporter-Like Protein 1 Inhibitors in Human Pancreatic Cancer Cells.* Int J Mol Sci, 2020. **21**(15).
24. Li, C., et al., *Keratin 80 promotes migration and invasion of colorectal carcinoma by interacting with PRKDC via activating the AKT pathway.* Cell Death Dis, 2018. **9**(10): p. 1009.
25. Attar, L.J., et al., *MPIG6B Gene-Related Myelofibrosis: A Rare Inherited Disease That Is Frequently Described in Arab Population.* Avicenna J Med, 2024. **14**(1): p. 69-72.
26. Clapham, D.E., *TRP channels as cellular sensors.* Nature, 2003. **426**(6966): p. 517-24.
27. Deng, Z., et al., *Gating of human TRPV3 in a lipid bilayer.* Nat Struct Mol Biol, 2020. **27**(7): p. 635-644.
28. Xu, H., et al., *Oregano, thyme and clove-derived flavors and skin sensitizers activate specific TRP channels.* Nat Neurosci, 2006. **9**(5): p. 628-35.
29. Holmes, R.S., L.A. Cox, and J.L. VandeBerg, *Comparative studies of mammalian acid lipases: Evidence for a new gene family in mouse and rat (Lipo).* Comp Biochem Physiol Part D Genomics Proteomics, 2010. **5**(3): p. 217-26.
30. Ding, J.F., et al., *IGFBP3 epigenetic promotion induced by METTL3 boosts cardiac fibroblast activation and fibrosis.* Eur J Pharmacol, 2023. **942**: p. 175494.
31. Wu, J.J., et al., *Microbiota-host crosstalk in the newborn and adult rumen at single-cell resolution.* BMC Biol, 2022. **20**(1): p. 280.
32. Fan, C., et al., *Single-Cell Transcriptome Integration Analysis Reveals the Correlation Between Mesenchymal Stromal Cells and Fibroblasts.* Front Genet, 2022. **13**: p. 798331.
33. Afzali, B. and C. Kemper, *Fibroblast tissue priming-not so nice to C you!* Immunity, 2021. **54**(5): p. 847-850.
34. Farmer, D.T., et al., *Defining epithelial cell dynamics and lineage relationships in the developing lacrimal gland.* Development, 2017. **144**(13): p. 2517-2528.
35. Park, J., et al., *Single-cell transcriptomics of the mouse kidney reveals potential cellular targets of kidney disease.* Science, 2018. **360**(6390): p. 758-763.
36. Gladka, M.M., et al., *Single-Cell Sequencing of the Healthy and Diseased Heart Reveals Cytoskeleton-Associated Protein 4 as a New Modulator of Fibroblasts Activation.* Circulation, 2018. **138**(2): p. 166-180.
37. Tusi, B.K., et al., *Population snapshots predict early haematopoietic and erythroid hierarchies.* Nature, 2018. **555**(7694): p. 54-60.
38. Xie, T., et al., *Single-Cell Deconvolution of Fibroblast Heterogeneity in Mouse Pulmonary Fibrosis.* Cell Rep, 2018. **22**(13): p. 3625-3640.
39. Castle, J.C., et al., *Immunomic, genomic and transcriptomic characterization of CT26 colorectal carcinoma.* BMC Genomics, 2014. **15**(1): p. 190.
40. Guo, M., et al., *SINCERA: A Pipeline for Single-Cell RNA-Seq Profiling Analysis.* PLoS Comput Biol, 2015. **11**(11): p. e1004575.
41. Ichikawa, T., et al., *Peroxynitrite augments fibroblast-mediated tissue remodeling via myofibroblast differentiation.* Am J Physiol Lung Cell Mol Physiol, 2008. **295**(5): p. L800-8.

42. Yeh, M.Y., et al., *Chitosan promotes immune responses, ameliorates glutamic oxaloacetic transaminase and glutamic pyruvic transaminase, but enhances lactate dehydrogenase levels in normal mice in vivo.* Exp Ther Med, 2016. **11**(4): p. 1300-1306.
43. Chueh, F.S., et al., *Crude extract of Polygonum cuspidatum promotes immune responses in leukemic mice through enhancing phagocytosis of macrophage and natural killer cell activities in vivo.* In Vivo, 2015. **29**(2): p. 255-61.
44. Chueh, F.S., et al., *Crude extract of Polygonum cuspidatum stimulates immune responses in normal mice by increasing the percentage of Mac-3-positive cells and enhancing macrophage phagocytic activity and natural killer cell cytotoxicity.* Mol Med Rep, 2015. **11**(1): p. 127-32.
45. Lin, C.C., et al., *Extract of Hedyotis diffusa Willd influences murine leukemia WEHI-3 cells in vivo as well as promoting T- and B-cell proliferation in leukemic mice.* In Vivo, 2011. **25**(4): p. 633-40.
46. Plasschaert, L.W., et al., *A single-cell atlas of the airway epithelium reveals the CFTR-rich pulmonary ionocyte.* Nature, 2018. **560**(7718): p. 377-381.
47. Sharifiaghdas, F., et al., *Comparing supportive properties of poly lactic-co-glycolic acid (PLGA), PLGA/collagen and human amniotic membrane for human urothelial and smooth muscle cells engineering.* Urol J, 2014. **11**(3): p. 1620-8.
48. Demir, I.E., et al., *Investigation of Schwann cells at neoplastic cell sites before the onset of cancer invasion.* J Natl Cancer Inst, 2014. **106**(8).
49. Saiki, T., et al., *The Effects of Insulin on Immortalized Rat Schwann Cells, IFRS1.* Int J Mol Sci, 2021. **22**(11).
50. Deborde, S., et al., *Schwann cells induce cancer cell dispersion and invasion.* J Clin Invest, 2016. **126**(4): p. 1538-54.
51. Dezawa, M. and E. Adachi-Usami, *Role of Schwann cells in retinal ganglion cell axon regeneration.* Prog Retin Eye Res, 2000. **19**(2): p. 171-204.
52. Azam, S.H. and C.V. Pecot, *Cancer's got nerve: Schwann cells drive perineural invasion.* J Clin Invest, 2016. **126**(4): p. 1242-4.
53. Tong, H.L., et al., *MiR-2425-5p targets RAD9A and MYOG to regulate the proliferation and differentiation of bovine skeletal muscle-derived satellite cells.* Sci Rep, 2017. **7**(1): p. 418.
54. Chen, J., et al., *Genome-wide identification of potential odontogenic genes involved in the dental epithelium-mesenchymal interaction during early odontogenesis.* BMC Genomics, 2023. **24**(1): p. 163.
55. Karihaloo, A., et al., *Hepatocyte growth factor-mediated renal epithelial branching morphogenesis is regulated by glypican-4 expression.* Mol Cell Biol, 2004. **24**(19): p. 8745-52.
56. Ho, C.M., et al., *Collagen type VI regulates the CDK4/6-p-Rb signaling pathway and promotes ovarian cancer invasiveness, stemness, and metastasis.* Am J Cancer Res, 2021. **11**(3): p. 668-690.
57. Donker, L., et al., *A mechanical G2 checkpoint controls epithelial cell division through E-cadherin-mediated regulation of Wee1-Cdk1.* Cell Rep, 2022. **41**(2): p. 111475.
58. Arai, C., et al., *Nephronectin plays critical roles in Sox2 expression and proliferation in dental epithelial stem cells via EGF-like repeat domains.* Sci Rep, 2017. **7**: p. 45181.

59. Han, Y., et al., *Coordinate control of basal epithelial cell fate and stem cell maintenance by core EMT transcription factor Zeb1.* Cell Rep, 2022. **38**(2): p. 110240.
60. Lixa, C., et al., *Retinoic Acid Binding Leads to CRABP2 Rigidification and Dimerization.* Biochemistry, 2019. **58**(41): p. 4183-4194.
61. Casteels, M., et al., *The role of 2-hydroxyacyl-CoA lyase, a thiamin pyrophosphate-dependent enzyme, in the peroxisomal metabolism of 3-methyl-branched fatty acids and 2-hydroxy straight-chain fatty acids.* Biochem Soc Trans, 2007. **35**(Pt 5): p. 876-80.
62. Itkonen, H.M., et al., *Lipid degradation promotes prostate cancer cell survival.* Oncotarget, 2017. **8**(24): p. 38264-38275.
63. Piórkowska, K., et al., *Evolution of peroxisomal trans-2-enoyl-CoA reductase (PECR) as candidate gene for meat quality.* Livestock Science, 2017. **201**: p. 85-91.
64. Zhang, M., et al., *Regulatory Roles of Peroxisomal Metabolic Pathways Involved in Musk Secretion in Muskrats.* J Membr Biol, 2019. **252**(1): p. 61-75.
65. Lieberman-Aiden, E., et al., *Comprehensive mapping of long-range interactions reveals folding principles of the human genome.* Science, 2009. **326**(5950): p. 289-93.
66. Schoenfelder, S. and P. Fraser, *Long-range enhancer-promoter contacts in gene expression control.* Nat Rev Genet, 2019. **20**(8): p. 437-455.
67. Li, D., et al., *Dynamic transcriptome and chromatin architecture in granulosa cells during chicken folliculogenesis.* Nature Communications, 2022. **13**(1): p. 131.
68. Al Kaabi, E.H. and A.W. El-Hattab, *N-acetylglutamate synthase deficiency: Novel mutation associated with neonatal presentation and literature review of molecular and phenotypic spectra.* Mol Genet Metab Rep, 2016. **8**: p. 94-8.
69. Floriot, S., et al., *CEP250 is Required for Maintaining Centrosome Cohesion in the Germline and Fertility in Male Mice.* Front Cell Dev Biol, 2021. **9**: p. 754054.
70. Li, Z.G., M.Y. Wu, and H.T. Jia, *[Research Progress on Expression Regulation, Function and Clinical Significance of CASP8AP2 Gene].* Zhongguo Shi Yan Xue Ye Xue Za Zhi, 2015. **23**(2): p. 557-61.
71. Shin, H. and H. Chung, *SMPDL3A links cholesterol metabolism to the cGAS-STING pathway.* Immunity, 2023. **56**(11): p. 2459-2461.
72. Zhou, L., et al., *Liver cancer stem cell dissemination and metastasis: uncovering the role of NRCAM in hepatocellular carcinoma.* J Exp Clin Cancer Res, 2023. **42**(1): p. 311.
73. Suddason, T. and E. Gallagher, *A RING to rule them all? Insights into the Map3k1 PHD motif provide a new mechanistic understanding into the diverse roles of Map3k1.* Cell Death Differ, 2015. **22**(4): p. 540-8.
74. Mulas, C., T. Kalkan, and A. Smith, *NODAL Secures Pluripotency upon Embryonic Stem Cell Progression from the Ground State.* Stem Cell Reports, 2017. **9**(1): p. 77-91.
75. Gauthier-Coles, G., et al., *Identification and characterization of a novel SNAT2 (SLC38A2) inhibitor reveals synergy with glucose transport inhibition in cancer cells.* Front Pharmacol, 2022. **13**: p. 963066.
76. Hu, J., et al., *NextPolish: a fast and efficient genome polishing tool for long-read assembly.* Bioinformatics, 2020. **36**(7): p. 2253-2255.
77. Langmead, B. and S.L. Salzberg, *Fast gapped-read alignment with Bowtie 2.* Nat Methods, 2012. **9**(4): p. 357-9.
78. Wingett, S., et al., *HiCUP: pipeline for mapping and processing Hi-C data.* F1000Res, 2015.

4: p. 1310.
79. Li, H., *Minimap2: pairwise alignment for nucleotide sequences.* Bioinformatics, 2018. **34**(18): p. 3094-3100.
80. Myers, E.W., *The fragment assembly string graph.* Bioinformatics, 2005. **21 Suppl 2**: p. ii79-85.
81. Zhang, X., et al., *Assembly of allele-aware, chromosomal-scale autopolyploid genomes based on Hi-C data.* Nat Plants, 2019. **5**(8): p. 833-845.
82. Li, H. and R. Durbin, *Fast and accurate long-read alignment with Burrows-Wheeler transform.* Bioinformatics, 2010. **26**(5): p. 589-95.
83. Bergman, C.M. and H. Quesneville, *Discovering and detecting transposable elements in genome sequences.* Brief Bioinform, 2007. **8**(6): p. 382-92.
84. Edgar, R.C. and E.W. Myers, *PILER: identification and classification of genomic repeats.* Bioinformatics, 2005. **21 Suppl 1**: p. i152-8.
85. Xu, Z. and H. Wang, *LTR_FINDER: an efficient tool for the prediction of full-length LTR retrotransposons.* Nucleic Acids Res, 2007. **35**(Web Server issue): p. W265-8.
86. Price, A.L., N.C. Jones, and P.A. Pevzner, *De novo identification of repeat families in large genomes.* Bioinformatics, 2005. **21 Suppl 1**: p. i351-8.
87. Benson, G., *Tandem repeats finder: a program to analyze DNA sequences.* Nucleic Acids Res, 1999. **27**(2): p. 573-80.
88. Mount, D.W., *Using the Basic Local Alignment Search Tool (BLAST).* CSH Protoc, 2007. **2007**: p. pdb top17.
89. Birney, E., M. Clamp, and R. Durbin, *GeneWise and Genomewise.* Genome Res, 2004. **14**(5): p. 988-95.
90. Kim, D., et al., *TopHat2: accurate alignment of transcriptomes in the presence of insertions, deletions and gene fusions.* Genome Biol, 2013. **14**(4): p. R36.
91. Trapnell, C., et al., *Differential gene and transcript expression analysis of RNA-seq experiments with TopHat and Cufflinks.* Nat Protoc, 2012. **7**(3): p. 562-78.
92. Haas, B.J., et al., *Improving the Arabidopsis genome annotation using maximal transcript alignment assemblies.* Nucleic acids research, 2003. **31**(19): p. 5654-5666.
93. Stanke, M. and S. Waack, *Gene prediction with a hidden Markov model and a new intron submodel.* Bioinformatics, 2003. **19 Suppl 2**: p. ii215-25.
94. Korf, I., *Gene finding in novel genomes.* BMC Bioinformatics, 2004. **5**: p. 59.
95. Majoros, W.H., M. Pertea, and S.L. Salzberg, *TigrScan and GlimmerHMM: two open source ab initio eukaryotic gene-finders.* Bioinformatics, 2004. **20**(16): p. 2878-9.
96. Guigo, R., *Assembling genes from predicted exons in linear time with dynamic programming.* J Comput Biol, 1998. **5**(4): p. 681-702.
97. Burge, C. and S. Karlin, *Prediction of complete gene structures in human genomic DNA.* J Mol Biol, 1997. **268**(1): p. 78-94.
98. UniProt Consortium, T., *UniProt: the universal protein knowledgebase.* Nucleic Acids Res, 2018. **46**(5): p. 2699.
99. Kanehisa, M., et al., *Data, information, knowledge and principle: back to metabolism in KEGG.* Nucleic Acids Res, 2014. **42**(Database issue): p. D199-205.
100. Wang, Y., et al., *MCScanX: a toolkit for detection and evolutionary analysis of gene synteny and collinearity.* Nucleic Acids Res, 2012. **40**(7): p. e49.

101. Veidenberg, A., A. Medlar, and A. Loytynoja, *Wasabi: An Integrated Platform for Evolutionary Sequence Analysis and Data Visualization.* Mol Biol Evol, 2016. **33**(4): p. 1126-30.
102. Talavera, G. and J. Castresana, *Improvement of phylogenies after removing divergent and ambiguously aligned blocks from protein sequence alignments.* Syst Biol, 2007. **56**(4): p. 564-77.
103. Posada, D. and K.A. Crandall, *MODELTEST: testing the model of DNA substitution.* Bioinformatics, 1998. **14**(9): p. 817-8.
104. Stamatakis, A., *RAxML version 8: a tool for phylogenetic analysis and post-analysis of large phylogenies.* Bioinformatics, 2014. **30**(9): p. 1312-3.
105. Li, L., C.J. Stoeckert, Jr., and D.S. Roos, *OrthoMCL: identification of ortholog groups for eukaryotic genomes.* Genome Res, 2003. **13**(9): p. 2178-89.
106. De Bie, T., et al., *CAFE: a computational tool for the study of gene family evolution.* Bioinformatics, 2006. **22**(10): p. 1269-71.
107. Xie, C., et al., *KOBAS 2.0: a web server for annotation and identification of enriched pathways and diseases.* Nucleic Acids Res, 2011. **39**(Web Server issue): p. W316-22.
108. Mao, X., et al., *Automated genome annotation and pathway identification using the KEGG Orthology (KO) as a controlled vocabulary.* Bioinformatics, 2005. **21**(19): p. 3787-3793.
109. Yanai, I., et al., *Genome-wide midrange transcription profiles reveal expression level relationships in human tissue specification.* Bioinformatics, 2005. **21**(5): p. 650-9.
110. Robinson, M.D., D.J. McCarthy, and G.K. Smyth, *edgeR: a Bioconductor package for differential expression analysis of digital gene expression data.* Bioinformatics, 2010. **26**(1): p. 139-40.
111. Chen, S., et al., *fastp: an ultra-fast all-in-one FASTQ preprocessor.* Bioinformatics, 2018. **34**(17): p. i884-i890.
112. Zheng, G.X., et al., *Massively parallel digital transcriptional profiling of single cells.* Nat Commun, 2017. **8**: p. 14049.
113. Butler, A., et al., *Integrating single-cell transcriptomic data across different conditions, technologies, and species.* Nat Biotechnol, 2018. **36**(5): p. 411-420.
114. Wang, T., et al., *Insights into left-right asymmetric development of chicken ovary at the single-cell level.* J Genet Genomics, 2024.
115. Leng, D., et al., *Single nucleus/cell RNA-seq of the chicken hypothalamic-pituitary-ovarian axis offers new insights into the molecular regulatory mechanisms of ovarian development.* Zoological Research, 2024. **45**(5): p. 1088-1107.
116. Durand, N.C., et al., *Juicer Provides a One-Click System for Analyzing Loop-Resolution Hi-C Experiments.* Cell Syst, 2016. **3**(1): p. 95-8.
117. Battulin, N., et al., *Comparison of the 3D organization of sperm and fibroblast genomes using the Hi-C approach.* Genome Biol, 2015. **16**(1): p. 77.
118. Kruse, K., et al., *TADtool: visual parameter identification for TAD-calling algorithms.* Bioinformatics, 2016. **32**(20): p. 3190-3192.
119. Ron, G., et al., *Promoter-enhancer interactions identified from Hi-C data using probabilistic models and hierarchical topological domains.* Nat Commun, 2017. **8**(1): p. 2237.

Figure 1

Click here to access/download;Figure;Figure1.pdf

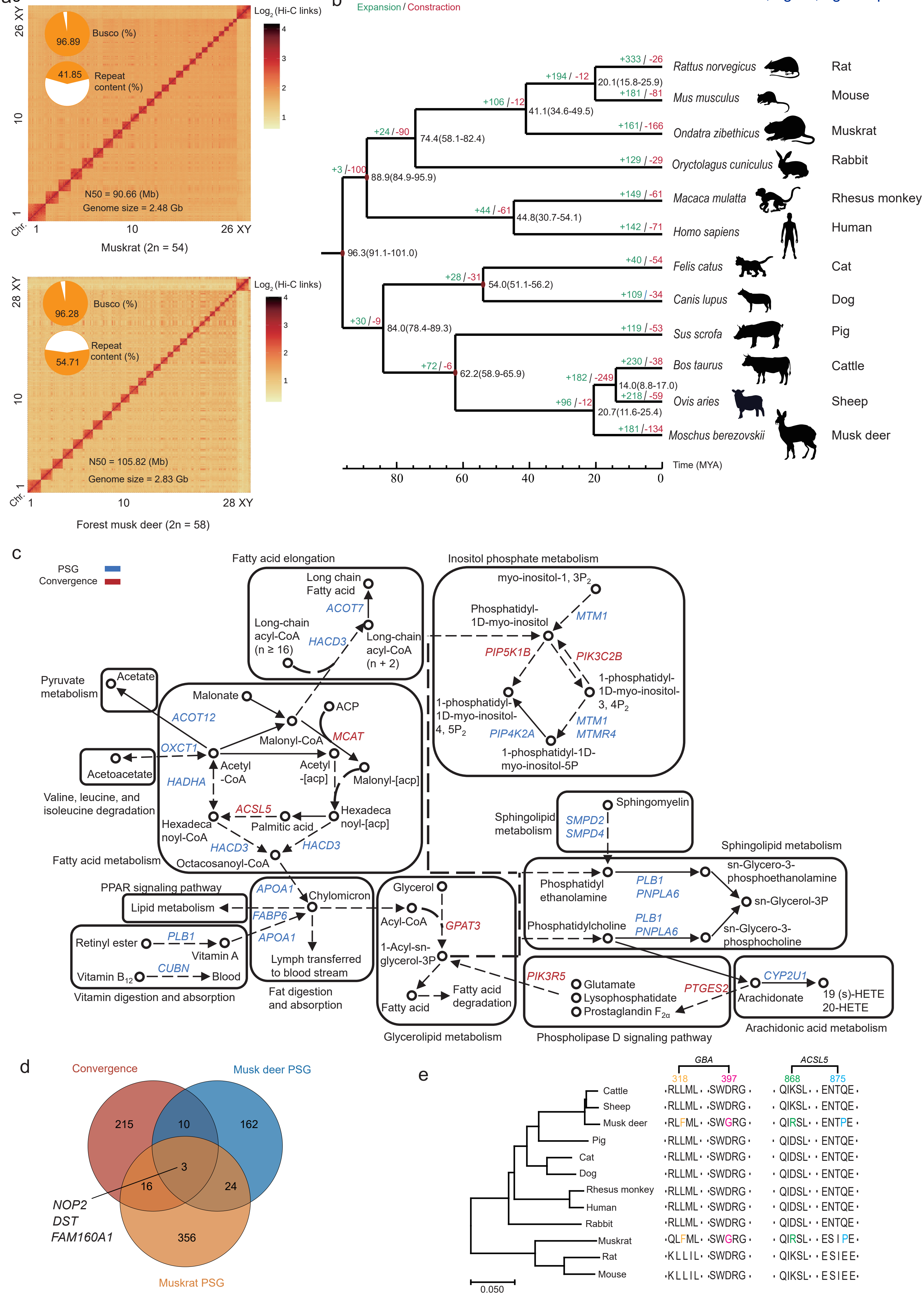

Figure 2

Click here to access/download;Figure;Figure2.pdf

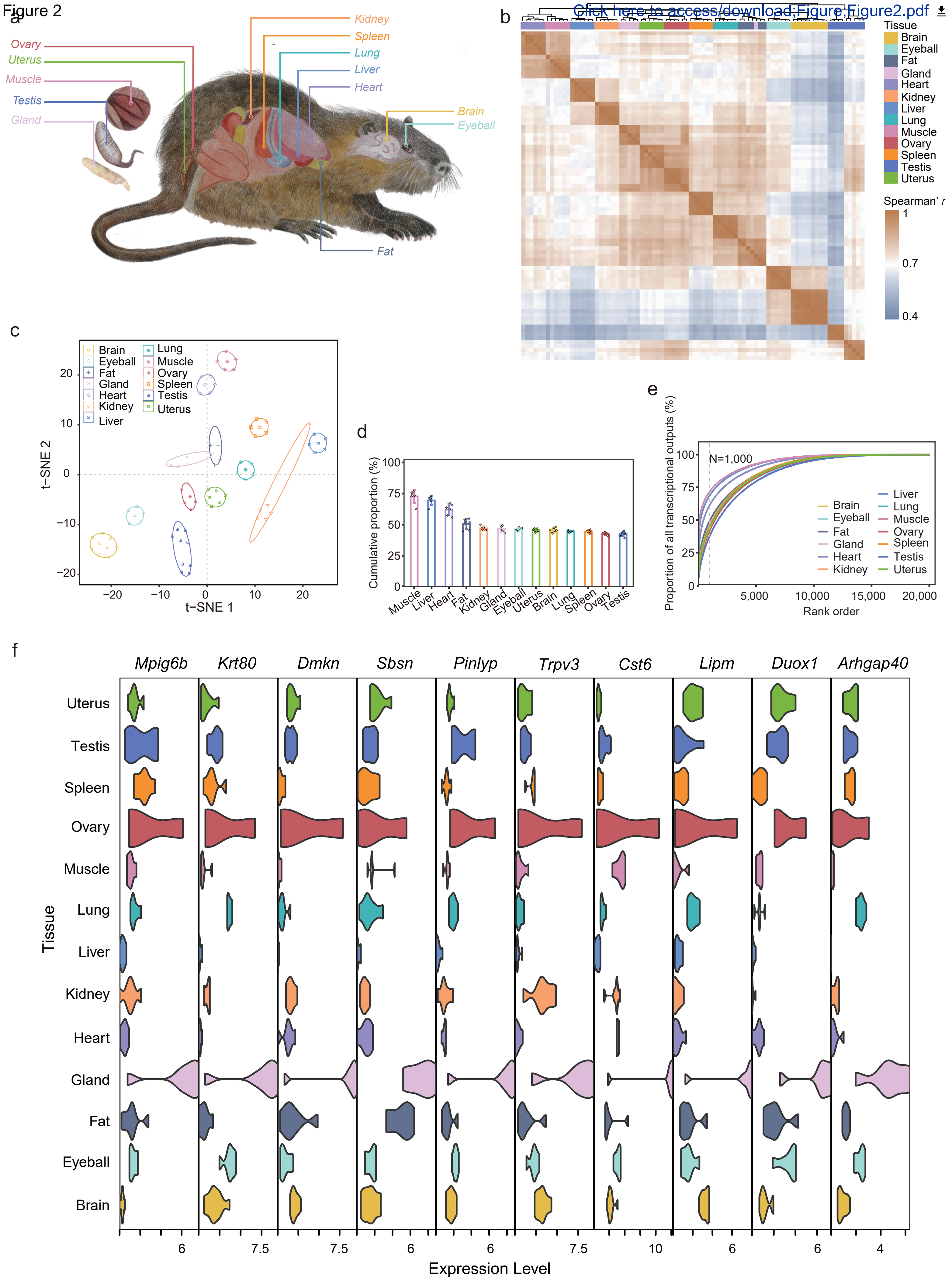

Figure 3 Click here to access/download;Figure;Figure3.pdf

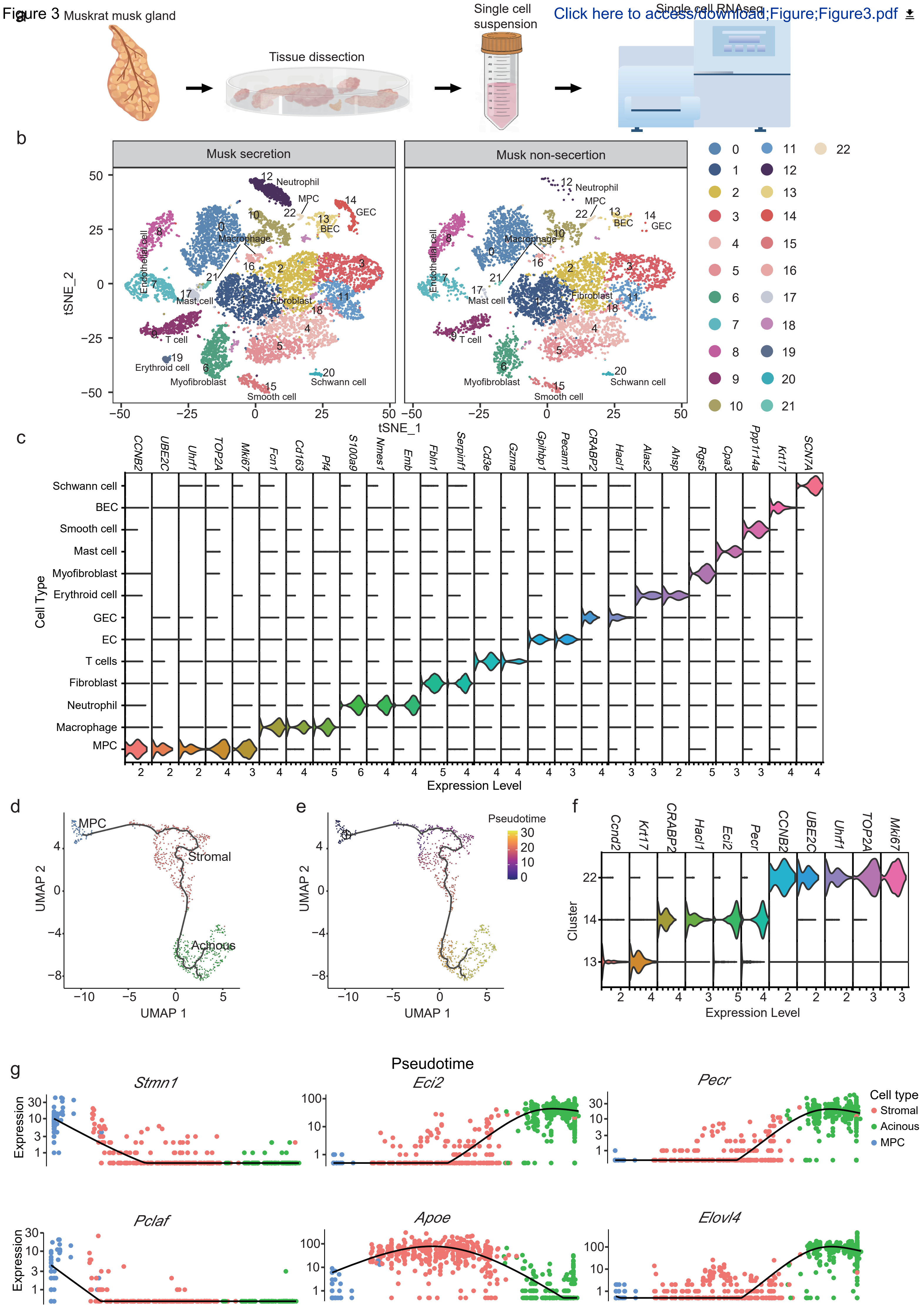

Figure 4

Click here to access/download;Figure;Figure4.pdf

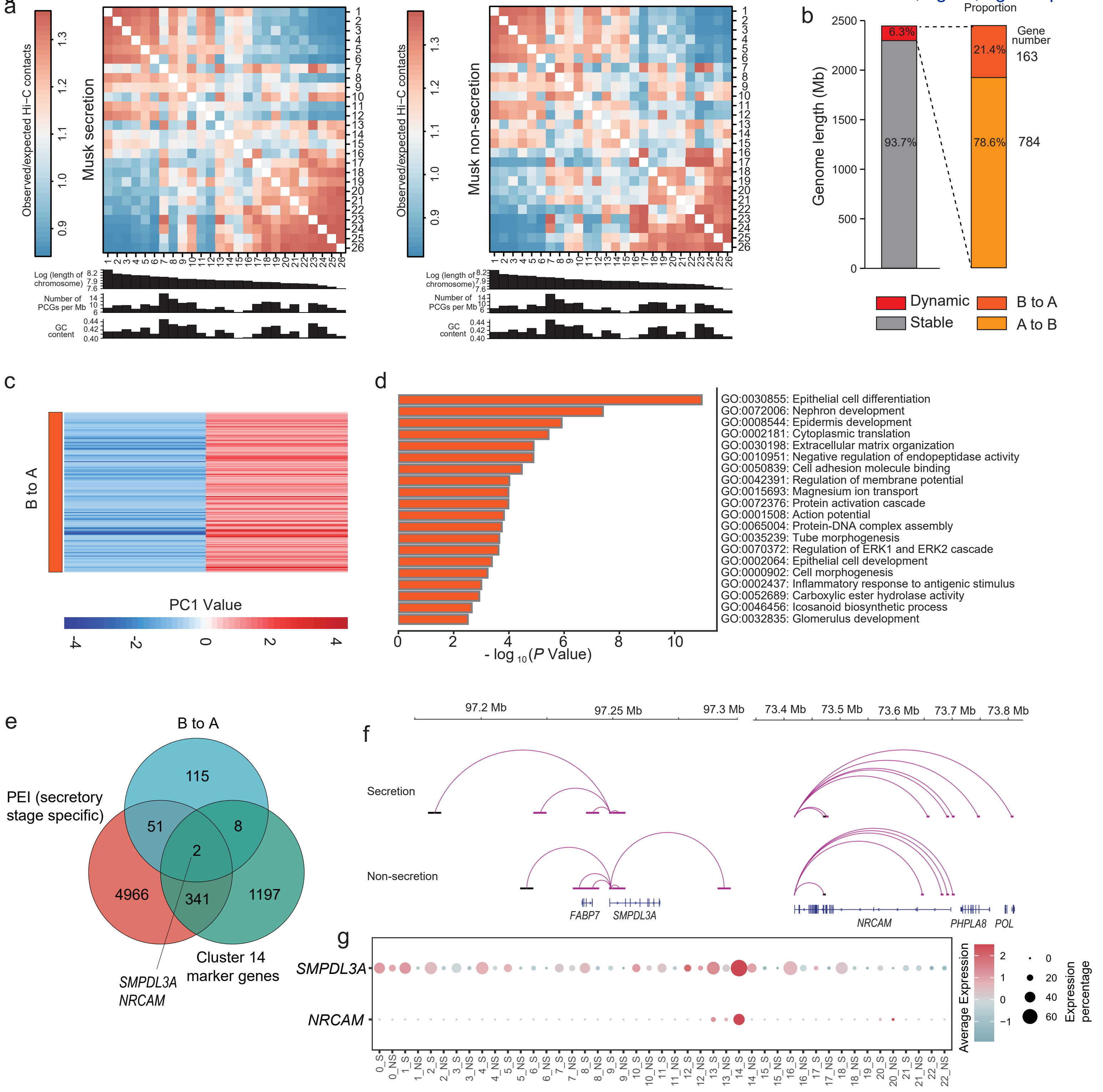

Figure 5

Click here to access/download;Figure;Figure5.pdf

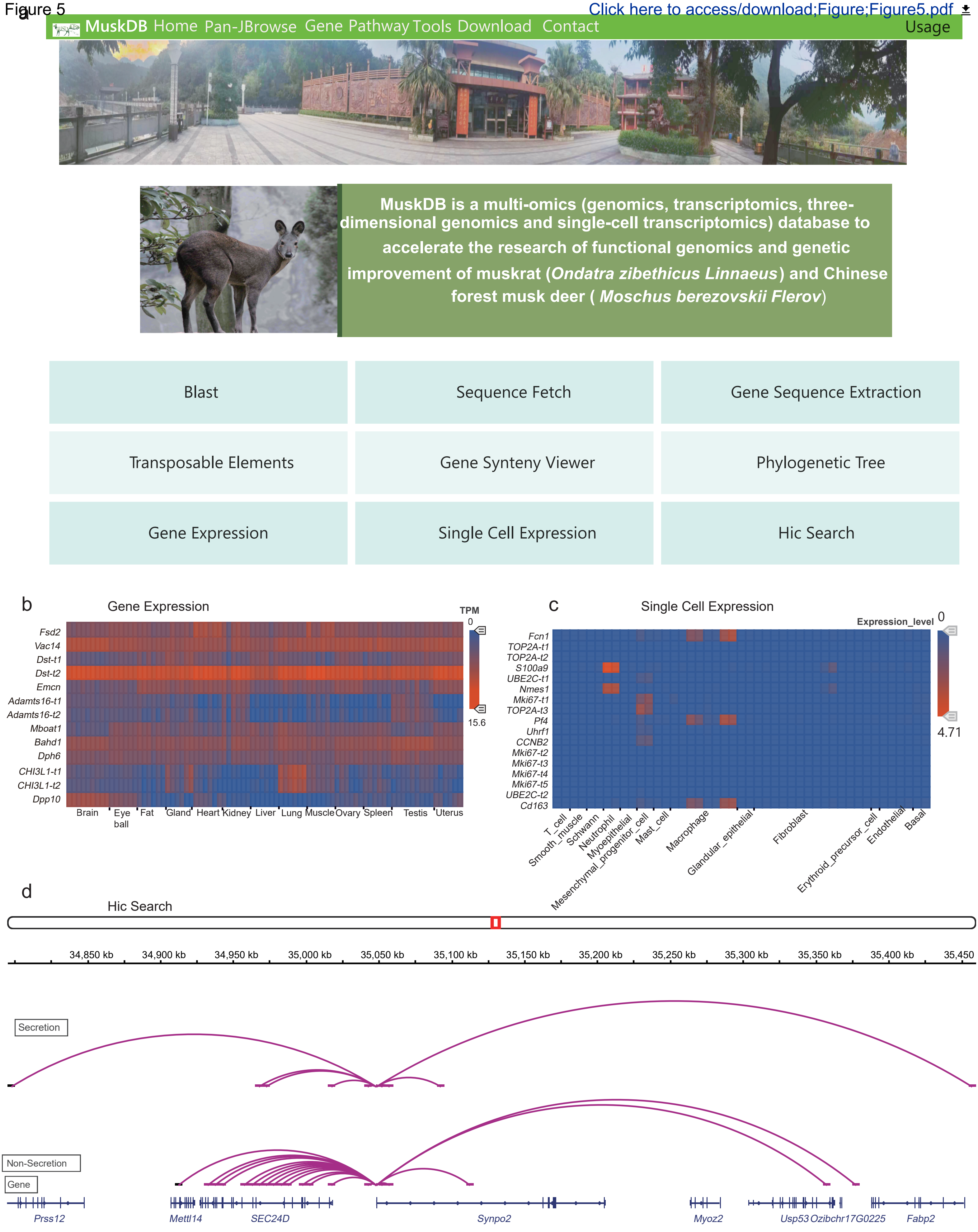

Click here to access/download
Supplementary Material
Additional file1.xlsx

Click here to access/download
Supplementary Material
Surpporting information.docx

Dear editor,

We sincerely thank you for allowing us to revise our manuscript entitled '***Multi-omics analyses identify distinct patterns of selection in musk secretion animals***' (original manuscript No. **GIGA-D-24-00205**) with substantial improvements and resolve all the concerns raised by the reviewers. 1) We revised our manuscript carefully, reanalyzed the data, especially the convergent evolution genes; 2) 'Better Papers Faster' was asked for our originally submitted and the current revised manuscripts for editing service to check for grammar, and polish the writing; 3) all figures are recognized.

Below we provide our point-to-point responses, and hope that you and the reviewers are satisfied many thanks for your consideration of our manuscript for possible publication in *GigaScience*.

We look forward to hearing a positive response from you.

Best regards,

Zhengrong Yuan, Diyan Li and Hang Jie

Detailed responses to reviewers

All comments provided by reviewers are in gray italics, and our responses are in black. Important revisions in the manuscript are marked in red.

Reviewer 1

Comment 1-1:

Wang et al. utilized multi-omics data to investigate the mechanism underlying musk secreting, by sequencing the genomes of two musk-secreting mammals (muskrat and musk deer), 188 RNA-seq libraries of muskrat organs, scRNA-seq, and HiC data. The size of dataset generated here is large and such omics data would provide great resources for related studies. However, I have several concerns.

Major:

1.The data presented in this study is impressive, but no strong connections among different data analysis sections (genome assembly, bulk RNA-seq analysis, scRNA-seq, HiC and selection analysis). The authors should carefully re-organize and connect these sections by logic. Five figures are recommended.

Suggestions on section organization: PSG and REG analysis should be moved after genome assembly part. The PEI (Line373) and compartment/TAD analysis (Line296) should be merged as one section.

Response 1-1:

Thanks for your positive and helpful comments.

(1) As suggested, only five figures were displayed in our revised manuscript.

(2) PSG and REG analysis was moved after genome assembly part.

(3) The PEI and compartment/TAD analysis were merged as one section.

Comment 1-2:

This study also sequenced 84 small RNA-seq libraries (line 78/line 145). But no analysis about small RNA was found in this manuscript. The small RNA-seq is miRNA-seq (Line 720)? I also noticed the sequencing of lncRNA-seq (Line 718). Too confusing.

Response 1-2:

Thanks for the comments, yes, we also sequenced 84 small RNA-seq libraries (it is miRNA). We only did basic analysis of miRNA (Figure S4b), including mapping, expression and t-SNE clustering analysis. But we did not do differential expression and function enrichment analysis among different tissues. For lncRNA-seq libraries, we mostly focused on analysis the expression profile of mRNAs. The lncRNA analysis was also similar to miRNA, only including mapping, expression and t-SNE clustering analysis (Figure S4a).

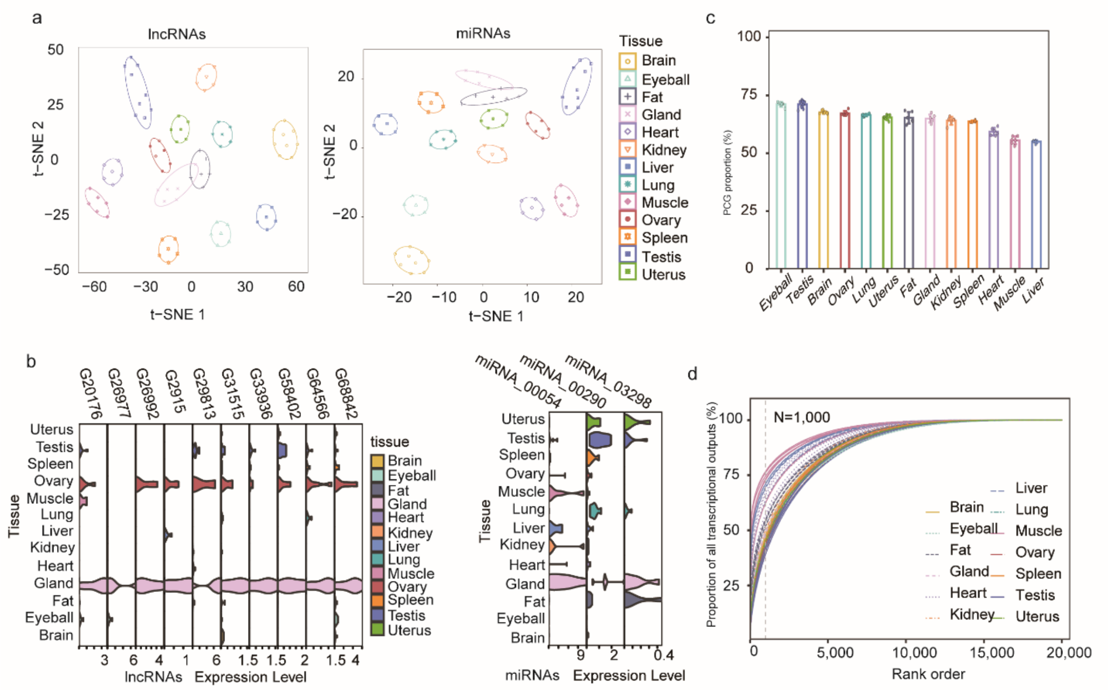

e

	Eyeball	Fat	Gland	Heart	Kidney	Liver	Lung	Muscle	Ovary	Spleen	Testis	Uterus
Brain	3,549	5,312	5,384	5,262	5,376	6,704	5,666	5,726	5,291	6,340	6,078	5,047
Eyeball		4,078	3,843	4,644	4,304	6,353	4,399	5,120	3,670	5,472	4,871	3,696
Fat			1,396	3,314	3,403	4,900	2,189	3,915	2,320	3,808	5,665	2,180
Gland				3,489	3,531	4,681	2,804	3,792	2,126	3,818	5,642	2,485
Heart					3,947	4,782	4,081	2,195	4,003	4,671	6,387	3,778
Kidney						3,802	3,858	4,474	3,952	4,687	5,914	3,716
Liver							5,310	4,827	5,626	5,331	7,626	5,449
Lung								4,480	3,065	3,361	5,996	2,872
Muscle									4,833	4,799	6,726	4,566
Ovary										4,018	4,732	1,727
Spleen											6,384	3,713
Testis												5,275

Figure S4. Gene expression from 13 tissues in muskrat. (a) t - distributed stochastic neighbor embedding (t-SNE) clustering of samples using lncRNA (left panel) and miRNA (right panel) expression. (b) The lncRNAs (left panel) and miRNAs (right panel) specifically expressed in musk gland. (c) The proportion of expressed genes of each sample. Each dot represents a sample in every tissue. (d) Abundance distribution of transcripts across tissues. The x-axis indicates the proportion of transcripts sorted from highest to lowest abundance, with the vertical dashed line indicating the top 1,000 of highest abundance transcripts. The y-axis indicates the accumulated fraction of transcripts relative to the total transcripts. Colored lines represent mean values across tissues. (e) Heatmap of DEGs (differentially expressed genes) numbers in pairwise comparisons among the 13 tissues. DEGs were identified using the threshold of |$\log_2$fold change| > 1 and corrected ($P < 0.01$).

Comment 1-3:

The material&method part was poorly organized and written and I can not follow. For example, in the part named "Expression analysis of mRNA, lncRNA and miRNA"(Line863), the contents were the experimental details of RNA-seq with no expression analysis methods. Also this experimental details repeated the previous contents (Line704-724) but with inconsistent description.

Response 1-3:

Thanks for your constructive comments. Sorry for the repeated “Expression analysis” methods. We deleted it and carefully revised the expression analysis method to the subsection of “Muskrat transcriptome reconstruction”.

Comment 1-4:

Many typos. The authors should check and revise carefully.

Response 1-4:

Thanks for the helpful comments. Sorry for our carelessness. In our revised manuscript, we have checked and revised carefully.

Comment 1-5:

Line94-96 The sequencing depth and quality should be mentioned here. Also only OZ sequencing data information was found in Table S1.

Response 1-5:

Thanks for your suggestions. We describe the sequencing depth of the two musk-secreting mammals (muskrat and musk deer): briefly, muskrat and forest musk deer had 235.25-fold and 246.02-fold sequencing depths, respectively. The quality sequencing is also shown in Table S1.

Comment 1-6:

Line 122-125 Simplify the statement, especially the use of software (CAFE, OrthoFinder..) which should be in Method.

Response 1-6:

We are sincerely grateful for your comments. We Simplify the statement, especially the use of software, as they are already described in the Methods. We also checked the results section for similar errors and made the same modifications, with the modification process marked in red.

Comment 1-7:

What function modules are the expanded or contracted gene families enriched in ? Or the numbers here mean nothing.

Response 1-7:

Thanks for the constructive comments. Enrichment analysis of expanded and contracted genes in the two musk-secreting mammals were performed using KEGG pathway, Reactome and Go ontology. The Top 10 enrichment pathways are shown in Fig S2a and the original results of enrichment analysis are shown in Table S7-S8.

GO enrichment analyses of contracted genes showed that these two species were both involved in pathways like “Olfactory Signaling Pathway”, “Olfactory transduction”, “Signaling by GPCR”, “B cell receptor signaling pathway”, “Tight junction Immunoregulatory interactions between a Lymphoid and a non-Lymphoid cell”, “Natural killer cell mediated cytotoxicity” and “Autoimmune thyroid disease” (Fig. S2a). Gene families underwent an expansion in these two species were involved in biological processes like “Cell Cycle”, “Meiotic synapsis”, “Mitotic Anaphase” and “Estrogen-dependent gene expression” (P < 0.05) (all Fisher’s exact test) (Table S7, Table S8).

Comment 1-8:

Line 131 The legend of Figure 1 should be revised, maybe "Genome assembly and gene family evolution in muskrat and musk deer"?. Other Figure legends

should be corrected.

Response 1-8:

Thanks, as suggested, we have modified the legend of Figure 1 to: Genome assembly and gene family evolution in muskrat and musk deer. Other Figure legends were also corrected.

Comment 1-9:

Line 171-172 PSGs and REGs should be explained here or re-organized.

Response 1-9:

Thanks for the comment. The PSGs and REGs were re-organized to “Positive selection and rapid evolution genes in muskrat and musk deer were mainly involved in metabolism of lipids and epithelial regulation” part.

Comment 1-10:

Line 186-196: Each sub-figure should have a sub-legend, rather than mix them together, e.g. (b) and (c).

Response 1-10:

As suggested, we revised each sub-figure have a sub-legend (such as new Figure 2, Figure 3 and Figure 4 and so on).

Comment 1-11:

*Line 187: "tissues *and* were used". Line 190: in the line"constructed at a probability of 0.95. (n = 19,800). (d)The cumulative", there should be no dot (.) after 0.95, "n" should be italic, also no space before "The". I hope the authors check and revise such typos carefully. In the following, I will not point them one by one.*

Response 1-11:

Thanks for the comments. Changes have been made accordingly.

Comment 1-12:

Line 200: "We" to ", we". Line 219: Neutrophil.

Response 1-12:

Changes have been made as suggested.

Comment 1-13:

Line 238: total cell numbers of secretion (12128) and non-secretion (7270) stages are different, direct comparison of cell numbers in each cluster to infer the cell cluster related to secreting is not reasonable. The cell abundance of each cluster should be normalized firstly and them compared between the two stages. State it clearly here, cell number or cell abundance/proportion.

Response 1-13:

Thanks for the constructive suggestion.

Yes, for the comparison between two stages, we firstly normalized firstly cell abundance of each cluster in each stage. We have revised the statement in the revised manuscript.

Comment 1-14:

Line 270-271: It should be "Figure 4a". This sentence should be removed.

Response 1-14:

Change was made. As suggested, the sentence was removed in our revised manuscript.

Comment 1-15:

Line 296: In the part of HiC analysis, the authors investigated compartment and TAD dynamics between the secreting and non-secreting stages. Despite the observed massive difference, this part looks isolated from previous expression analysis. Logically the author analyzed the expression changes and inferred some candidate genes involved in the secreting progress from both bulk and scRNA-seq, the following step should be examining whether the conformation of these loci (e.g. CRABP2, Hacl1, Eci2, and Pecr) has changed in the secreting process, like from inactive compartment B to active compartment A, TAD fusion and fission. Although the authors described some cases (e.g. ROS1, SOX9, NUS1, NPNT), no expression data (Bulk, scRNA-seq) are provided. Similarly, the PEI analysis is weak. The author merged the HiC data and analyzed the interactions between promoters and enhancers, regardless of methodological issues, the meaningful PEIs should be those showing changes from non-secreting to secreting stage, like increased interaction strength.

Response 1-15:

Thanks for the helpful suggestions. As suggested, we reanalyzed the data. Thus, the candidate genes involved in the secreting progress from scRNA-seq expression were further logically analyzed whether the 3D genome conformation of these loci changed. As a result, Figure 4 and Figure S9 were regenerated.

At the PEI (promoter-enhancer interaction) level, we found that there were two genes (SMPDL3A and NRCAM) with specific PEI in musk secretion stage, also showed a compartment transition from B to A and was a marker gene in Cluster 14 (Fig. 4e). There were more specific long-range interactions (> 25Kb) in musk secretion stage for these two genes (Fig. 4f). The gene SMPDL3A showed a relative higher expression in all clusters at the musk secretion stage (Fig. 4g). SMPDL3A (sphingomyelin phosphodiesterase acid-like 3A) is an enzyme induced by lipid metabolism through liver X receptor that degrades cGAMP, modulating the cGAS-STING pathway which is involved in immune responses and lipid sensing [68]. NRCAM (neuronal cell adhesion molecule) primarily associated with neural development, its expression also promotes malignant cell transformation, cell motility, and metastatic disease [69]. In addition,

MAP3K1, NODAL and Slc38a2 also showed significantly more PEIs and contacted with more enhancers during the musk secretion stage (Fig S10). MAP3K1 is a key component of the protein kinase signal transduction cascade and plays a crucial role in cellular signaling pathways [70]. NODAL maintains stem cell pluripotency and promotes directed differentiation [71]. Slc38a2 encodes an amino acid transport protein that facilitates cellular uptake of amino acids [72]. These results suggest that two important functions of the musk gland during the *musk s*ecretion stage are lipid metabolism and cell specialization, which indicate that synthesis and secretion activity were very active at this stage. Our results demonstrated that candidate loci can be analyzed in future studies of musk secretion mechanisms.

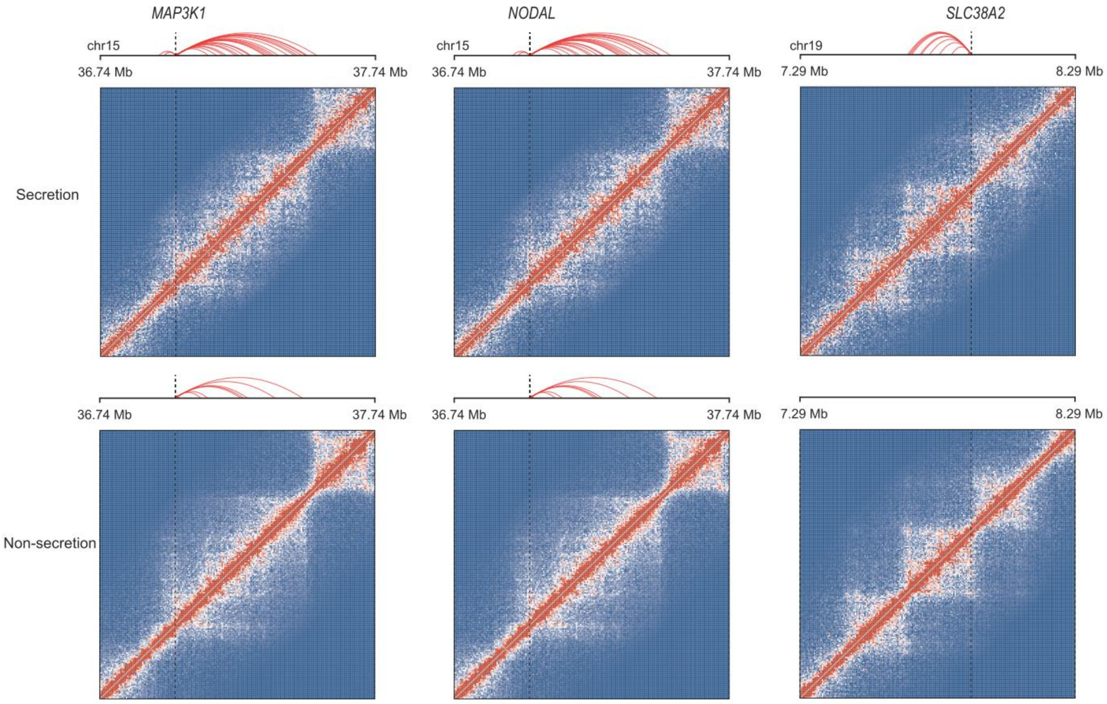

Figure S10. Promoter-enhancer interactions (PEIs) rewired in the musk gland of musk secretion and non-secretion stages.

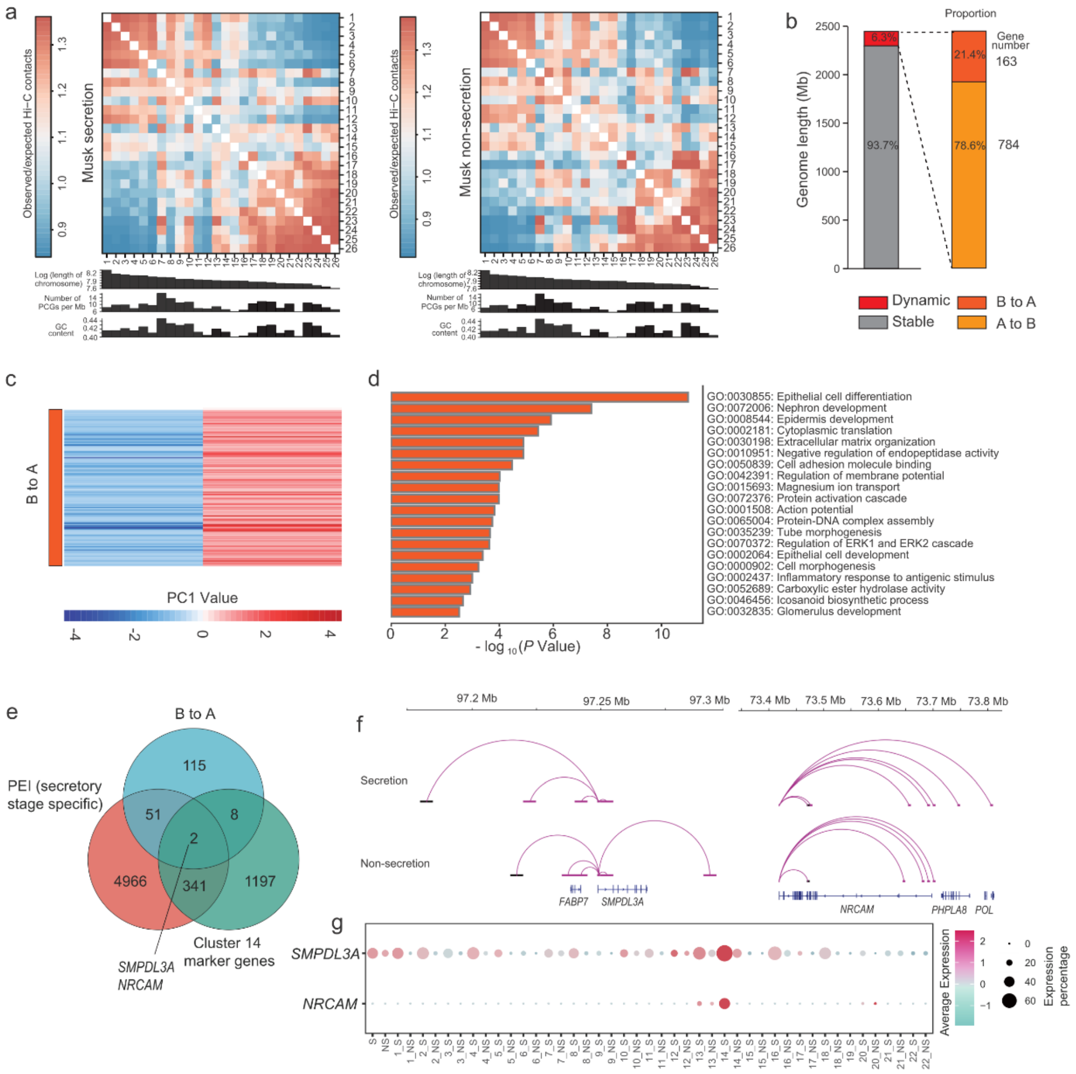

Figure 4: Global chromatin interaction patterns in musk gland of musk secretion and non-secretion stages.

Comment 1-16:

Line 346: there is no statement in the manuscript referring to Fig 5f.

Response 1-16:

Thanks for the comment, as suggested, we reanalyzed our data and present meaningful genes in our revised manuscript and cited the figures. See comment 1-15.

Comment 1-17:

Line 411: This part should be moved following the genome assembly part.

Response 1-17:

As suggested, this part was moved following the genome assembly part.

Comment 1-18:

Line471: The paragraph about five genes in REG and DEGs in cell cluster 14 should be moved to the scRNA-seq part.

Response 1-18:

Thanks for the helpful comment, as suggested, we moved this paragraph to the scRNA-seq part.

Comment 1-19:

Line 500: Database is useful but there are too many figures in the manuscript, and I would suggest shortening this part into one or two sentences in the conclusion section, and moving the figure in the supplementary material.

Response 1-19:

Thanks for the helpful comment. As suggested, there are only five figures in our revised manuscript, including this figure. And we also shortening this part.

Reviewer #2:

The manuscript titled "Multi-omics analyses identify distinct patterns of selection in musk secretion animals" by Wang et al. explored the molecular mechanisms of musk secretion using high-quality genome, Hi-C, RNA-seq, and scRNA-seq data. Additionally, they provide an open database platform (MuskDB), which is an important resource. Although the topic is interesting, some limitations in the text description and data analysis were found in this paper. After these concerns have been revised and clarified, I suggest that this

manuscript could be accepted for publication.

Thank you for your positive comments. In our revised manuscript, we reorganized the text, figures and supplementary files. And logically, analyzed 3D genome reorganization of candidate genes with showing their expression profile in musk gland tissue and different cell types.

Comment 2-1:

1. In the part of abstract, the logical flow of the results differs significantly from the overall structure of the article, which can be confusing. Additionally, while the authors described their work using multi-omics analysis, just mentioned different pathways identified by each omics approach. Did the analysis revealed any common pathways or genes that are supported by multiple lines of evidence?

In addition, the application of musk in biomedicine should be mentioned to enhance the research significance of this paper.

Response 2-1:

Thanks for the helpful suggestions. In our revised manuscript, we reanalyzed our data. And presented candidate genes by multiple lines of evidence. The abstract was also rewritten.

In addition, as suggested, the application of musk in biomedicine was added: Natural musk has long been an important component of traditional Chinese medicine, and was used as resuscitation, blood circulation, collateral drainage, detumescence and pain relief [2].

Comment 2-2:

2. In introduction, it is suggested to propose the clear scientific questions and hypotheses. What are the key points of this study? Please highlights of these discoveries.

Response 2-2:

As suggested, in introduction, we propose the clear scientific questions: However, there are no convergent evolutionary studies on musk-producing animals to elucidate the related mechanism of musk secretion. In the process of evolution, which genes are subject to positive and convergent evolution? Which genes are differentially expressed in different cells of the musk gland during the period of musk secretion? And how the molecular process of musk secretion regulated by these genes from chromatin conformation is unknown.

The key points of this study were also rewritten: Notably, genes like *SMPDL3A* and *NRCAM* showed specific PEIs and compartment transitions, indicating their potential role in musk secretion regulation. The study concludes that the adaptation evolution of musk secretion in muskrat and musk deer is likely underpinned by active lipid metabolism and cell specialization, highlighting the complexity of the musk gland and the need for further research into the functional implications of musk secretion-specific genetic variants.

Comment 2-3:

3. The authors assembled the genomes of musk deer and muskrat using ONT, Hi-C, and NGS sequencing data. Given the relatively high error rate associated with ONT sequencing, appropriate correction methods should be applied. Additionally, the specific parameters used in the assembly process were not detailed. Please added in the methods section to provide a reference for these parameters.

Response 2-3:

Thank you for the constructive comments. As suggested, we added the specific detailed parameters used in the assembly process: For the correction of the initial contigs, we utilized the software NextPolish (v1.4.1), employing high-quality T7 paired-end reads and Nanopore long reads, and applied the recommended algorithm modules "best." in Nextpolish.

The specific parameters and pipelines used in the assembly process are available at Zenodo (https://doi.org/10.5281/zenodo.13690583).

Comment 2-4:

4. Figure S1 only shows the results for muskrats; I suggest including an additional figure to display the assembly results for musk deer. Moreover, both newly assembled genomes should be compared with existing reference genomes to further demonstrate the high quality of these assemblies, like collinearity comparison, et al.

Response 2-4:

As suggested, we compared with existing musk deer reference genomes to further demonstrate the high quality of our assemblies.

Our two assemblies have improved N50 length of the contig for the muskrat by 1,048- and 3.56-fold compared to the published sequences [9], respectively (Fig. S1b), and musk deer by 1,048- and 3.56-fold compared to the published sequences (Fig. S1c).

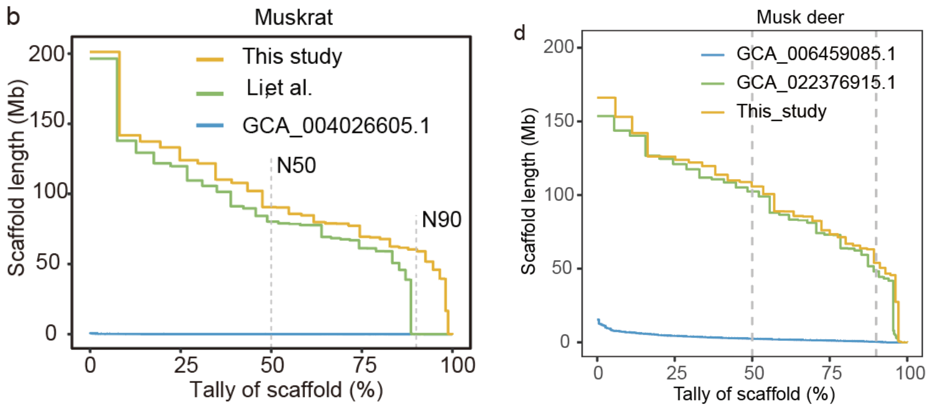

Figure S1: (b) Muskrat genome assembly in this study compared with two previous studies. (d) Musk deer genome assembly in this study compared with two previous studies.

Comment 2-5:

5. Which functions are the expanded and contracted gene families associated with? This should be detailed in a supplementary table.

Response 2-5:

Thanks for the constructive comments. Enrichment analysis of expanded and

contracted genes in the two musk-secreting mammals were performed using KEGG pathway, Reactome and Go ontology. The Top 10 enrichment pathways are shown in Fig S2a and the original results of enrichment analysis are shown in Table S7-S8.

GO enrichment analyses of contracted genes showed that these two species were both involved in pathways like “Olfactory Signaling Pathway”, “Olfactory transduction”, “Signaling by GPCR”, “B cell receptor signaling pathway”, “Tight junction Immunoregulatory interactions between a Lymphoid and a non-Lymphoid cell”, “Natural killer cell mediated cytotoxicity” and “Autoimmune thyroid disease” (Fig. S2a). Gene families underwent an expansion in these two species were involved in biological processes like “Cell Cycle”, “Meiotic synapsis”, “Mitotic Anaphase” and “Estrogen-dependent gene expression” ($P < 0.05$) (all Fisher’s exact test) (Table S7, Table S8).

Comment 2-6:

6. Fig. 2e-d: The authors have depicted the gene expression level distribution. However, a tissue-specific expression profile might provide more insightful information.

Response 2-6:

Thanks for the helpful comments, we agree that a tissue-specific expression profile provide more insightful information. We provided the tissue-specific expression genes enrichment analysis results in Figure S4 for each tissue.

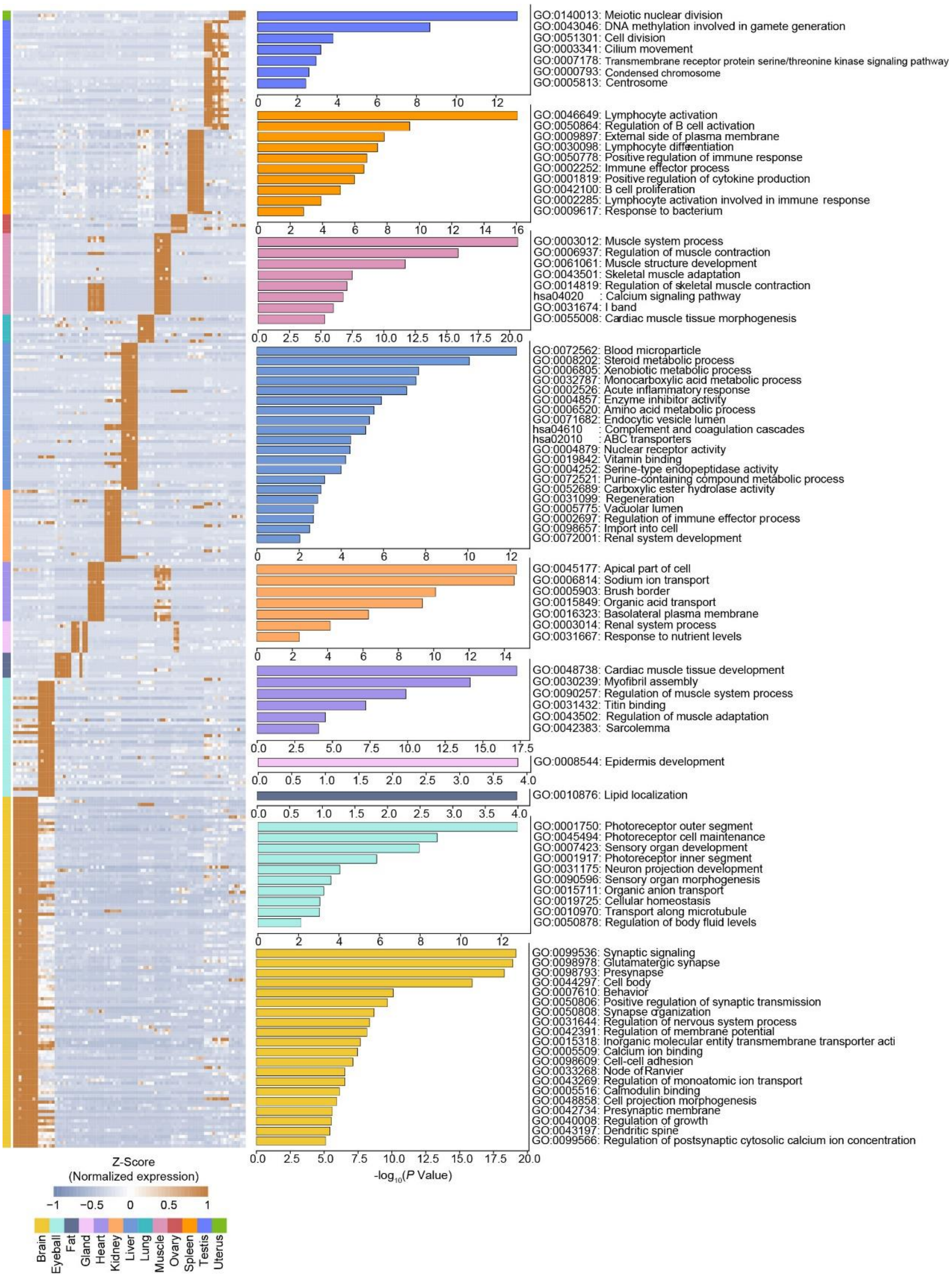

Fig. S4 Significantly enriched GO terms in tissue-specific expressed genes for each tissue. Tissue specificity of gene abundance was reflected by the tau score (τ).

Comment 2-7:

7. Line 168: The number of tissue-specific expressed genes for each tissue should be clearly described for clarity.

Response 2-7:

As suggested, we added the numbers of tissue-specific expressed genes for each tissue.

In terms of expression, there are 3 (uterus), 35 (testis), 27 (spleen), 6 (ovary), 26 (muscle), 9 (lung), 47 (liver), 23 (kidney), 19 (heart), 10 (gland), 8 (fat), 38 (eyeball), and 112 (brain) tissue-specific genes were detected.

Comment 2-8:

8. Line 171-174: It is unclear whether the PSG and REG described in this section are also tissue-specific expressed genes related to the musk gland. These PSGs and REGs should overlapped with musk-gland tissue-specific genes, and then be analyzed for possible functions.

Response 2-8:

Thanks for the helpful comments, the genes here was not musk-gland tissue-specific genes. Thus, we moved these genes' results to "Positive selection and rapid evolution genes in muskrat and musk deer were mainly involved in metabolism of lipids and epithelial regulation" part.

Comment 2-9:

9. If the authors later focus more on the biological function of the Acinar cells/glandular epithelial cells cluster, it would be beneficial to present the marker genes for this specific cell type using immunofluorescence staining.

Response 2-9:

Thanks for the helpful comments, the using immunofluorescence staining is very useful for present the marker genes. We need to collect sample from different musk secretion stage, this need time, we hope we could present this in our further studies.

Comment 2-10:

10. The overall methodological description in the single-cell transcriptomics section is overly simplistic. It is unclear whether the single-cell data from the two stages were analyzed jointly or separately. Additionally, it is not specified whether the pseudotime analysis was conducted using the secretion stage, the non-secretion stage, or both. These aspects need clarification to ensure a more comprehensive understanding of the analysis workflow. Providing detailed information on these points will help readers better understand the study's design and the interpretability of the results.

Response 2-10:

Thanks for the helpful comments, as suggested, we expanded methodological description in the single-cell transcriptomics section. 1) the single-cell data from the two stages were analyzed jointly; 2) pseudotime analysis was conducted using both stage cells.

Pseudotime Analysis

In R, utilizing the Monocle3 software package v1.3.1 (Cao et al., 2019), pseudotime trajectories for all 13, 14 and 22 subpopulations in the musk gland were constructed. This technique sorts individual cells along their developmental paths based on how closely their gene expression patterns match those of other sequenced cells, effectively mapping out the dynamics of cellular changes [110, 111]. Furthermore, the method evaluates how genes work together in space by using Moran's Index to measure their co-expression, which helps to establish a timeline of gene expression changes.

Identification of differentially expressed genes (DEGs) in Cluster 14 cells

DEGs in the Cluster 14 cells of musk secretion and non-secretion stages were identified using the FindMarkers function in Seurat. Genes meeting the thresholds ($|\log_2 FC| > 1$, *P*-adjusted < 0.05, min.pct = 0.25) were considered as DEGs. The results were visualized using dot plots.

Comment 2-11:

11. The number of differentially expressed genes (DEGs) both in RNA-seq and scRNA-seq should be clearly specified, and detailed information should be presented in a supplementary table. Additionally, a more stringent threshold should be applied for identifying DEGs: abs(log2FC)>1 and corrected ($P < 0.05$). Furthermore, it is important to determine whether the tissue-specific expressed genes in the musk gland are differentially expressed between the secretion and non-secretion stages.

Response 2-11:

Thanks for the helpful comments. The number of differentially expressed genes (DEGs) in RNA-seq were presented in Figure S3e a more stringent threshold (abs(log2FC)>1 and corrected ($P < 0.01$)).

As suggested, a supplementary table was provided for these tissue specific genes and DEGs for scRNA-seq (Table S11 and Table S14).

e

Eyeball	Fat	Gland	Heart	Kidney	Liver	Lung	Muscle	Ovary	Spleen	Testis	Uterus	
3,549	5,312	5,384	5,262	5,376	6,704	5,666	5,726	5,291	6,340	6,078	5,047	Brain
	4,078	3,843	4,644	4,304	6,353	4,399	5,120	3,670	5,472	4,871	3,696	Eyeball
		1,396	3,314	3,403	4,900	2,189	3,915	2,320	3,808	5,665	2,180	Fat
			3,489	3,531	4,681	2,804	3,792	2,126	3,818	5,642	2,485	Gland
				3,947	4,782	4,081	2,195	4,003	4,671	6,387	3,778	Heart
					3,802	3,858	4,474	3,952	4,687	5,914	3,716	Kidney
						5,310	4,827	5,626	5,331	7,626	5,449	Liver
							4,480	3,065	3,361	5,996	2,872	Lung
								4,833	4,799	6,726	4,566	Muscle
									4,018	4,732	1,727	Ovary
										6,384	3,713	Spleen
											5,275	Testis

Figure S3e Heatmap of DEGs (differentially expressed genes) numbers in pairwise comparisons among the 13 tissues. DEGs were identified using the threshold of abs(log2FC)>1 and corrected ($P < 0.01$).

Comment 2-12:

12. Line 391-393: The authors explored the changes in PEIs during the secretion and non-secretion periods, but they did not further analyze the impact of these changes on the expression levels of the corresponding genes. Including such an analysis could provide valuable insights into the regulatory

effects of PEI changes on gene expression and their potential biological implications.

Response 2-12:

Thanks for the constructive comments. As suggested, we reanalyzed the data. Thus, the candidate genes involved in the secreting progress from scRNA-seq expression were further logically analyzed whether the 3D genome conformation of these loci changed. As a result, Figure 4 and Figure S9 were regenerated. The expression of the genes showed a higher expression in musk secretion stage.

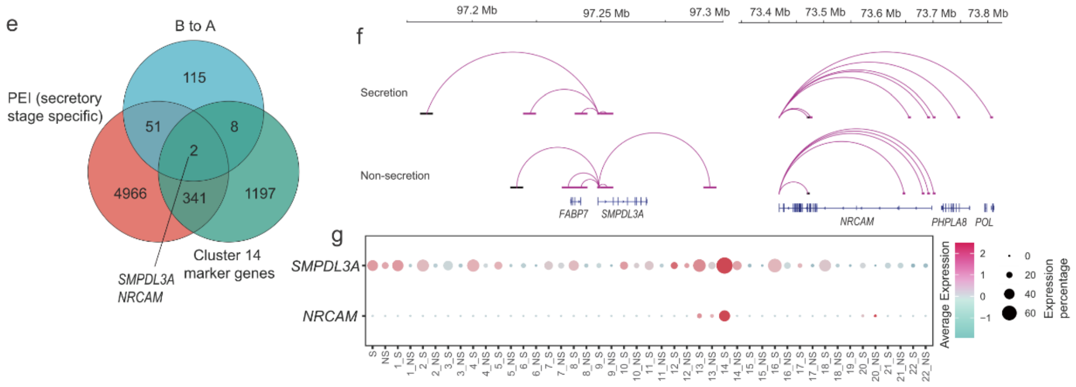

Figure 4. (e) Veen diagram showing overlapping genes with compartment B to A, PEI specific to musk secretion stage, and marker genes in Cluster 14. (f) Promoter-enhancer interactions (PEIs) rewired in the musk gland of musk secretion and non-secretion stages of *SMPDL3A* and *NRCAM*. (g) Gene expression of *SMPDL3A* and *NRCAM* in each cluster at musk secretion and non-secretion stages.

Comment 2-13:

13. line 417-419: The authors have provided limited information about the criteria used for selecting positively selected genes (PSGs) and rapidly evolving genes (REGs). It is important to clearly specify the threshold settings for these selections. Additionally, the genes identified through this process should be presented in a supplementary table to ensure transparency and allow for further analysis. Moreover, the authors could leverage the species-specific PSGs and

REGs to identify sets of convergently positively selected genes and convergently rapidly evolving genes. This approach could yield insights into shared evolutionary pressures or functional adaptations across different species. Including these analyses would enhance the depth and comprehensiveness of the study.

Response 2-13:

Thanks for the comments. For PSGs, we utilized the free-ratio branch-site mode (model = 1) as an alternative model, assuming positive selection on the foreground branch. The null model allowed sites to undergo purifying selection or evolve neutrally. For REGs, we utilized the branch model, specifically the one-ratio model (model = 0) as the null model assuming the same evolutionary rate for all branches, and the two-ratio model (model = 2) as an alternative model allowing different evolutionary rates for the foreground branch. The likelihood ratio test (LRT) method was used to detect differences between the nested models, and P-values were computed based on $\chi 2$ statistics. Multiple testing was corrected using the false discovery rate (FDR) method.

As suggested, we provided the PSGs, REGs and convergently genes (**Table S6**). Convergently positively selected genes and convergently rapidly evolving genes were also detected (**Fig S3**).

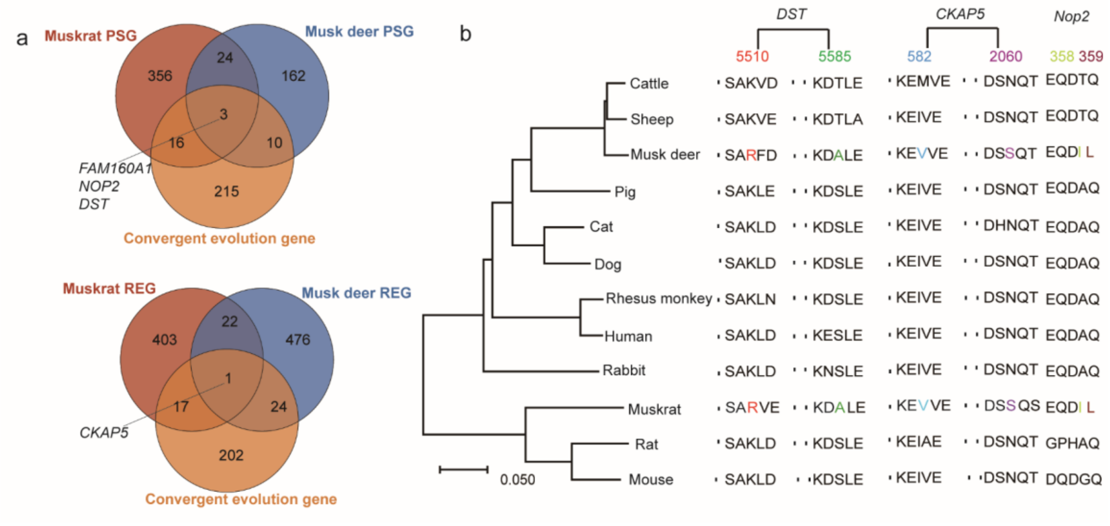

Fig S3. Overlapped genes between PSGs (REGs) and convergent evolution genes (a) and sequence alignment highlighting sites that evolved in 3 representative genes (b).

Comment 2-14:

14. line428: Identifying convergent amino acid substitution sites and genes can indeed be influenced by various confounding factors, potentially leading to a high rate of false positives. Implementing a multi-method approach will enhance the validity and robustness of the study's conclusions. To improve the accuracy of these identifications, employing multiple methods is advisable. Methods such as "conv_cal" and "CCS" are effective for screening convergent amino acid substitutions. By using a combination of these methods, researchers can cross-verify findings, minimize false positives, and increase confidence in identifying true instances of convergent evolution.

Response 2-14:

As suggested, we used "CCS" methods to screen convergent amino acid substitutions.

Convergent Evolution among musk secretion animals

To test for convergence among musk secretion animals (muskrat and musk deer), we used 12 mammals from Fig. 1b. Based on the phylogenetic tree, we used two methods to detect the convergent amino acid substitutions for each node of the 7,409 single-copy orthologs: 1) method of Zhang and Kumar (Zhang JZ, Kumar S. 1997. Detection of convergent and parallel evolution at the amino acid sequence level. Mol Biol Evol. 14(5):527–536; Zou Z, Zhang J. 2015. Are convergent and parallel amino acid substitutions in protein evolutionmore prevalent than neutral expectations?Mol Biol Evol. 32(8):2085–2096.). A site was assumed as a convergent site if amino acids of a focused node at that site are the same but different with their most recent ancestral amino acids. Amino acid sequences of internal nodes for all the 7,409 single-copy orthologs were reconstructed by CODEML in PAML. For each gene, the number of observed convergent site was compared with the neutral expectations derived from the JTT-fgene model, and the Poisson test was then used to evaluate the difference. 2) CCS method (Xu S, He Z, Guo Zet al. Genome-wide convergence during

evolution of mangroves from woody plants. Mol Biol Evol2017; 34: 1008–15.), the convergent signal is identified when all two musk secretion species (muskrat and musk deer) share the same derived character at a conservative site.

Comment 2-15:

15. line411: To ensure robust conclusions, it would be beneficial to include a broader range of species in the analysis. This expanded dataset would help determine the identified sites exhibit true convergence specifically in muskrat and musk deer, reducing the risk of false positives that might arise from a limited set of species.

Response 2-15:

Thanks for the helpful comments. We agree that broader range of species in the analysis will help determine the identified sites exhibit true convergence specifically in muskrat and musk deer, reducing the risk of false positives that might arise from a limited set of species. Considered the quality of the species' genome and the number of one-to-one orthologous genes in the species, we selected 12 species for our analysis. We will sequenced more musk secretion species genome in our further study.

Comment 2-16:

16. line439: The TEX15 as a testis-specific protein in the text suggests that it may not be directly related to the species-specific trait of musk secretion in the musk gland. Some more apt examples, focusing on genes that are more directly associated with the unique characteristics of the musk gland could provide more relevant insights into the molecular underpinnings of this trait.

Response 2-16:

Thanks for the helpful comments. As suggested, we reanalyzed the data. Thus, the candidate genes involved in the secreting progress from scRNA-seq expression were further logically analyzed whether the 3D genome

conformation of these loci changed. As a result, Figure 4 and Figure S9 were regenerated.

At the PEI (promoter-enhancer interaction) level, we found that there were two genes (SMPDL3A and NRCAM) with specific PEI in musk secretion stage, also showed a compartment transition from B to A and was a marker gene in Cluster 14 (Fig. 4e). There were more specific long-range interactions (> 25Kb) in musk secretion stage for these two genes (Fig. 4f). The gene SMPDL3A showed a relative higher expression in all clusters at the musk secretion stage (Fig. 4g). SMPDL3A (sphingomyelin phosphodiesterase acid-like 3A) is an enzyme induced by lipid metabolism through liver X receptor that degrades cGAMP, modulating the cGAS-STING pathway which is involved in immune responses and lipid sensing [68]. NRCAM (neuronal cell adhesion molecule) primarily associated with neural development, its expression also promotes malignant cell transformation, cell motility, and metastatic disease [69]. In addition, MAP3K1, NODAL and Slc38a2 also showed significantly more PEIs and contacted with more enhancers during the musk secretion stage (Fig S9). MAP3K1 is a key component of the protein kinase signal transduction cascade and plays a crucial role in cellular signaling pathways [70]. NODAL maintains stem cell pluripotency and promotes directed differentiation [71]. Slc38a2 encodes an amino acid transport protein that facilitates cellular uptake of amino acids [72]. These results suggest that two important functions of the musk gland during the *musk s*ecretion stage are lipid metabolism and cell specialization, which indicate that synthesis and secretion activity were very active at this stage. Our results demonstrated that candidate loci can be analyzed in future studies of musk secretion mechanisms.

Comment 2-17:

17. The manuscript shows p-values in all of the pathway enrichment figures, yet the methods section states that p-value correction for multiple testing was done. Additionally, line 431 mentions using p-values to indicate significance. The authors need to clarify this discrepancy regarding multiple testing correction.

It is important for the authors to specify whether the p-values displayed in the figures and mentioned in the text are raw or have been corrected for multiple comparisons. This transparency is essential to ensure that the reported significance levels accurately reflect the analyses' reliability, considering the potential for false positives when multiple tests are performed. Clearly explaining these details is crucial for the study's credibility and reproducibility.

Response 2-17:

Thanks for the comments. As suggested, we have revised the p-value correction methods in our revised manuscript. It is corrected for multiple testing by the Benjamini-Hochberg method as described in our methods part.

Comment 2-18:

18. line452: It would be necessary to provide further details on the three adaptive convergent genes, such as NOP2, DST, and FAM160A1. For instance, creating a visual representation like figure, could help illustrate the situation within the context of convergent evolution.

Response 2-18:

Thanks for the constructive comments. As suggested, we generated a figure to illustrate the situation within the context of convergent evolution (Figure S3).

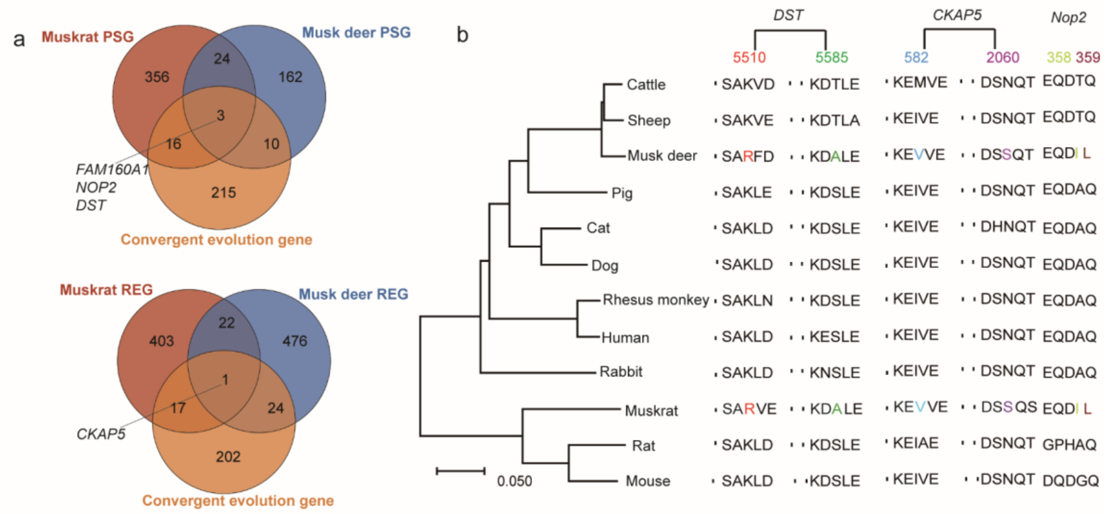

Fig S3. Overlapped genes between PSGs (REGs) and convergent evolution genes (a) and sequence alignment highlighting sites that evolved in 3

representative genes (b).

Comment 2-19:

19. The authors should explore whether any of the convergently positively selected genes or convergent amino acid replacement genes are related to musk secretion in the musk gland. Investigating whether these genes are specifically expressed in musk gland tissue or display species-specific expression can yield valuable insights into their relevance to the unique traits of the musk gland. These analyses could help to establish a connection between these genes and the adaptive characteristics associated with musk gland function.

Response 2-19:

Thanks for the constructive comments. As suggested, we analyzed whether the convergently positively selected genes or convergent amino acid replacement genes are related to musk secretion in the musk gland. Because there are only 10 musk gland tissue specific genes, there was no overlap with the convergently positively selected genes. Among these 10 genes, *KRT80* and *MPIG6B* genes were also PSGs in muskrat. These two genes were also limited studied. Keratin 80 (KRT80) is an intermediate filament protein that contributes to the structural integrity of epithelial cells. The megakaryocyte and platelet inhibitory receptor gene G6P (MPIG6B) regulates platelets production, aggregation, and activation.

Comment 2-20:

20. In conclusion, the most important findings of this study should be clarified, and the description of the relevant methods will not be repeated.

Response 2-20:

Thanks for the comments. We rewritten the most important findings of this study in both abstract and introduction:

The study identified particular genes, such as *SMPDL3A* and *NRCAM*, that

exhibit unique patterns of genetic interactions and changes in their genomic neighborhoods, suggesting they may play a key role in controlling the musk secretion process. The research concludes that the evolutionary adaptation for musk production in both the muskrat and the musk deer is likely driven by robust lipid metabolism and specialized cell functions, which underscores the intricate nature of the musk gland. This finding emphasizes the necessity for continued investigation to uncover the full functional impact of genetic variations that are specific to the musk secretion process.

The repeated methods were revised in our new manuscript.

Comment 2-21:

Minor suggestions:

1. Acronyms: Ensure that all acronyms are spelled out full name in the first time when they appear in the text for better understanding.

2. line691 and line698 are same.

3. Methods descriptions should also be listed in order of article logic and importance. For example, you should first write "sample collection" section and so on.

Response 2-21:

Thanks for the constructive comments. As suggested, all acronyms are spelled out full name in the first time when they appear in the text for better understanding; the repeated methods were removed; we first write "sample collection".

Reviewer #3

Comment 3-1:

Natural musk is mainly secreted by musk gland located between the navel and genitals of mature male forest musk deer. But the molecular mechanism of musk-secretion is still wait to be uncovered, hindered by the lack of

comprehensive multi-omics analyses and respective platform. In this study, the authors successfully generated high quality 2.48 Gb and 2.83 Gb for muskrat and musk deer genomes with contig N50 values of 60.53 and 69.45Mb, which anchored onto 28 and 30 chromosomes, respectively. To explore the genes specifically expressed in musk gland and their functions, we used muskrat as a model animal to conduct further analyses, as muskrat tissue samples are accessible in contrast to the endangered musk deer by 168 muskrat transcriptomes. And the further single cell RNA sequencing and Hi-C analysis indicated that enhanced expression during the musk secretion stage of muskrat is related to the biological process "regulation of secretion". Overall, these results show that active lipid metabolism may underlie the adaptation evolution of musk secretion. Those findings, alongside the provided database, facilitate a deeper understanding of the molecular mechanism underlying the unique phenomenon of musk secretion, and may provide insights for the mating behavior and breeding of muskrat and musk deer.

The findings are meaningful, the database is very important. I think it could be accepted to be published after a little polish.

Response 3-1:

We are delighted to receive your comments. We sincerely appreciate the thoughtful and constructive comments from you and another two anonymous reviewers, in improving our manuscript.
As suggested, we mainly focused the analyses on the genes with both expression and 3D genomic changes. In addition, in our revised manuscript, 1) we regenerated all figures, tables and supplementary files; 2) added and revised relative results, methods and discussion; 3) we polish our manuscript with a professional English language editing company. All comments with our point-by-point responses to each reviewer are listed. We sincerely appreciate your assistance in improving our manuscript.
