# Supplementary material for: Multiomics analysis provides insights into musk secretion in muskrat and musk deer
Source: Gigascience. 2025 Feb 26;14:giaf006. doi: 10.1093/gigascience/giaf006 (PMC11878540; doi:10.1093/gigascience/giaf006)
Supplement: giaf006_GIGA-D-24-00205_Revision_2 [file giaf006_giga-d-24-00205_revision_2.pdf]

GigaScience

Multi-omics analysis provides insights into musk secretion in muskrat and musk deer

--Manuscript Draft--

**Manuscript Number:**	GIGA-D-24-00205R2
**Full Title:**	Multi-omics analysis provides insights into musk secretion in muskrat and musk deer
**Article Type:**	Research
**Funding Information:**	National Natural Science Foundation of China (81973428) — Dr Hang Jie<br>National Natural Science Foundation of China (82274046) — Dr Hang Jie<br>the Fundamental Research Funds of Chongqing (2022JK017) — Dr Hang Jie<br>the Beijing Nova Program (Z211100002121022，20230484446) — Mr. Shilin Tian<br>National Natural Science Foundation of China (32272859) — Mr. Zhengrong Yuan
**Abstract:**	Background<br>Musk, secreted by the musk gland of adult male musk-secreting mammals, holds significant pharmaceutical and cosmetic potential. However, understanding the molecular mechanisms of musk secretion remain limited, largely due to the lack of comprehensive multi-omics analyses and available platforms for relevant species, such as muskrat (Ondatra zibethicus Linnaeus) and Chinese forest musk deer (Moschus berezovskii Flerov).<br>Results<br>We generated chromosome-level genome assemblies for the two species muskrat (Ondatra zibethicus Linnaeus) and musk deer (Moschus berezovskii Flerov), along with 168 transcriptomes from various muskrat tissues. Comparative analysis with eleven other vertebrate genomes revealed genes and amino acid sites with signs of adaptive convergent evolution, primarily linked to lipid metabolism, cell cycle regulation, protein binding, and immunity. Single-cell RNA sequencing in muskrat musk glands identified increased acinar/glandular epithelial cells during secretion, highlighting the role of lipometabolism in gland development and evolution. Additionally, we developed MuskDB (http://muskdb.cn/home/), a freely accessible multi-omics database platform for musk-secreting mammals.<br>Conclusions<br>The study concludes that the evolution of musk secretion in muskrats and musk deer is likely driven by lipid metabolism and cell specialization. This underscores the complexity of the musk gland and calls for further investigation into musk secretion-specific genetic variants.
**Corresponding Author:**	Diyan Li<br>Chengdu University<br>Chengdu, CHINA
**Corresponding Author Secondary Information:**	
**Corresponding Author's Institution:**	Chengdu University
**Corresponding Author's Secondary Institution:**	
**First Author:**	Tao Wang
**First Author Secondary Information:**	
**Order of Authors:**	Tao Wang<br>Maosen Yang

Powered by Editorial Manager® and ProduXion Manager® from Aries Systems Corporation

	Xin Shi
	Shilin Tian
	Yan Li
	Wenqian Xie
	Zhengting Zou
	Dong Leng
	Ming Zhang
	Chengli Zheng
	Chungang Feng
	Bo Zeng
	Xiaolan Fan
	Huimin Qiu
	Jing Li
	Guijun Zhao
	Diyan Li
	Zhengrong Yuan
	Hang Jie
**Order of Authors Secondary Information:**	
**Response to Reviewers:**	Dear editor,<br>We sincerely thank you for allowing us to revise our manuscript entitled 'Multi-omics analysis provides insights into musk secretion in muskrat and musk deer' (original manuscript No. GIGA-D-24-00205) with substantial improvements and resolve all the concerns raised by the reviewers. 1) We revised our manuscript carefully and updated the database; 2) 'Better Papers Faster' was asked for our current revised manuscripts for editing service to check for grammar and polish the writing again.<br>Below we provide our point-to-point responses, and hope that you and the reviewers are satisfied many thanks for your consideration of our manuscript for possible publication in GigaScience.<br>We look forward to hearing a positive response from you.<br><br>Best regards,<br>Zhengrong Yuan, Diyan Li and Hang Jie<br><br>Detailed responses to reviewers<br>All comments provided by reviewers are listed.<br>____<br>Reviewer 1<br>Comment #1<br>I would like to thank the authors for carefully considering reviewers' comments. I believe the manuscript has substantially improved after the revision. I think this paper has provided rich multi-omics data resource for related studies, including high-quality genomes, RNA-seq, scRNA-seq, Hi-C and others. Selection analysis is only one part of this paper, so the "selection" in the title may be not appropriate. I think the title should be "Multi-omics analysis provides insights into musk secretion in muskrat and musk deer" or something like this. About data Availability, I did not see any statements about the public database where the datasets generated in this study were deposited. Prerequisites of acceptance must include data availability. I have no further comments on biology-related analysis. However I am not satisfied with the wording, just as the first-round review. Please note and revise the wording carefully again, especially singular and plural forms (e.g. Line512 contacts vs contact), Past tense and present tense (e.g. line514, is/showed; line527, analyzes). The authors said they have polished the English wording. However still massive but simple typos were found.<br>Response #1

Powered by Editorial Manager® and ProduXion Manager® from Aries Systems Corporation

	1)Thanks for the constructive comments, as suggested, we revised the title to “Multi-omics analysis provides insights into musk secretion in muskrat and musk deer”;<br>2)Line510, contacts vs contact: “contacts” —>”contact”<br>3)Line511, is/showed: We changed “when gene Synpo2 is entered, the results showed the contact profile of this gene in two stages of secretion and non-secretion” to “When the gene Synpo2 is entered, the results display the contact profile of this gene at the stages of secretion and non-secretion”.<br>4)Line524, “comprehensively analyzes” was changed to “comprehensively analysis of the Hi-C”.<br>5)‘Better Papers Faster’ was asked for our current revised manuscripts for editing service to check for grammar, and polish the writing again;<br><br>Comment #2<br>Line 35: "muskrats" should be "muskrat".<br>Response #2<br>Line 36: "muskrats" was changed to "muskrat".<br><br>Comment #3<br>Line 38-39: genomes can not be generated by sequencing... "We generated chromosome-level genome assemblies for the two species muskrat and musk deer, along with 168 transcriptomes from various muskrat tissues" will be better. You have spelled out the full Latin names above.<br>Response #3<br>Thanks for the constructive comment. We have made the modifications as suggested (Line 39-40).<br><br>Comment #4<br>Line 43: too general. The statement should be quantitative.<br>Response #4<br>Thanks for the comment. We have revised the sentence to “Single-cell RNA sequencing in muskrat musk glands identified increased acinar/glandular epithelial cells during secretion, highlighting the role of lipometabolism in gland development and evolution”.<br><br>Comment #5<br>Line 45: About the Database. The URL is bad. Nobody remembers the IP address of a database. I visited this database and found some functions required user registering and login, e.g. Download module and Tools module. Jbrowse only provided gene and TE element tracks. The authors have generated so many omic datasets, why not display them in the Jbrowse? Hic or HiC or Hi-C, please keep it unified.<br>Response #5<br>Thanks for the helpful comment.<br>(1)We have applied for the record of our database to China government. The website is: http://muskdb.cn/home/ . Please check again. And we will continually add more data of the other musk secretion animals’ data in our future study.<br>(2)All user registering and login are all canceled, please check the database again.<br><br>Comment #6<br>Line 48-49: move to result part.<br>Response #6<br>We have deleted " Genes such as SMPDL3A and NRCAM exhibited specific PEIs and compartment transitions, suggesting a role in musk secretion." from the conclusions. Because the specific expressions are already detailed in the results, here we simply want to emphasize their importance.<br><br>Comment #7<br>Line 107: remove "(only male secret musk)".<br>Response #7<br>Change has been made as suggested.<br><br>Comment #8<br>Line112: ONT coverage or all sequencing data coverage?<br>Response #8<br>It is all sequencing data coverage.

Comment #9
Line 116-117: generated 2.48-Gb and 2.83-Gb assemblies
Response #9
We changed “2.48 Gb and 2.83 Gb” to “2.48-Gb and 2.83-Gb”

Comment #10
Line 118: 69.45 Mb. No space. which were anchored
Response #10
“69.45Mb” —> “69.45 Mb”

Comment #11
Line 123-124: 1,048- and 3.56-fold for the muskrat and musk deer, compared to the published sequences, respectively ?
Response #11
Thanks for the comment, sorry for our carelessness.
Yes, it is “1,048- and 3.56-fold for the muskrat and musk deer, compared to the published sequences, respectively”.

Comment #12
Line 163: and convergence gene set?
Response #12
Yes, it is convergence gene set. Change has been made as suggested.

Comment #13
Line169-170: no chromatin structure changed genes were mentioned.
Response #13
Thanks for the comment, we have removed chromatin structure information in the sentence. Yes, this part is only PSGs.

Comment #14
Line184-185: above analysis was PSG and REG, no expression analysis.
Response #14
Thanks for the comment, we have removed this sentence.

Comment #15
Line212: why "PSGs (REGs)", rather than "PSGs, REGs"
Response #15
Thanks for the helpful comment, "PSGs (REGs)" was changed to "PSGs, REGs".

Comment #16
Line 213: Fig.1d show three genes overlapped by the three gene sets. No CKAP5.
Response #16
The results of CKAP5 is shown in Supplementary Fig. S3.

Comment #17
Line 238: change "in the transcriptome of muskrat among" to "from".
Response #17
As suggested, "in the transcriptome of muskrat among" was changed to "from" (Line 236).

Comment #18
Line 343: REGs.
Response #18
Line 340, 182, “REG” was changed to “REGs”.

Comment #19
Line 344: cell division
Response #19
“Cell division” was changed to “cell division”, Line 342.

Comment #20
Line 345: GPC4 and COL6A3, no italic
Response #20

Powered by Editorial Manager® and ProduXion Manager® from Aries Systems Corporation

	Line 202, 204, 217, 272, 346, 349, 351, 391, 394, 471, 474, 478: The format for gene names has been corrected to italicize them.<br><br>Comment #21<br>Line 352: PSGs. Still too many typos. I will not point them out one by one.<br>Response #21<br>Thanks for the helpful comment, we revised the manuscript again. “PSGS” was revised to “PSGs”.
**Additional Information:**	
**Question**	**Response**
Are you submitting this manuscript to a special series or article collection?	No
**Experimental design and statistics**<br><br>Full details of the experimental design and statistical methods used should be given in the Methods section, as detailed in our Minimum Standards Reporting Checklist. Information essential to interpreting the data presented should be made available in the figure legends.<br><br>Have you included all the information requested in your manuscript?	Yes
**Resources**<br><br>A description of all resources used, including antibodies, cell lines, animals and software tools, with enough information to allow them to be uniquely identified, should be included in the Methods section. Authors are strongly encouraged to cite Research Resource Identifiers (RRIDs) for antibodies, model organisms and tools, where possible.<br><br>Have you included the information requested as detailed in our Minimum Standards Reporting Checklist?	Yes
**Availability of data and materials**<br><br>All datasets and code on which the conclusions of the paper rely must be either included in your submission or	Yes

Powered by Editorial Manager® and ProduXion Manager® from Aries Systems Corporation

deposited in publicly available repositories (where available and ethically appropriate), referencing such data using a unique identifier in the references and in the “Availability of Data and Materials” section of your manuscript.<br><br>Have you have met the above requirement as detailed in our Minimum Standards Reporting Checklist?	

Powered by Editorial Manager® and ProduXion Manager® from Aries Systems Corporation

Manuscript Click here to access/download;Manuscript;Revised MS-Li.docx

Multi-omics analysis provides insights into musk secretion in muskrat and musk deer

Tao Wang[1], Maosen Yang[2,3], Xin Shi[4], Shilin Tian[5], Yan Li[6], Wenqian Xie[7], Zhengting Zou[8], Dong Leng[9], Ming Zhang[9], Chengli Zheng[4], Chungang Feng[10], Bo Zeng[9], Xiaolan Fan[9], Huimin Qiu[11], Jing Li[11], Guijun Zhao[3], Zhengrong Yuan[7], Diyan Li[3*] and Hang Jie[2*]

[1] School of Basic Medical Sciences, Chengdu University, Chengdu, 610106, China
[2] Jinfo Mountain Forestry Ecosystem of Chongqing Observation and Research Station, Chongqing Institute of medicinal plant cultivation, Chongqing University of Chinese Medicine, Chongqing 402760, China
[3] School of Pharmacy, Chengdu University, Chengdu 610106, China
[4] Sichuan Institute of Musk Deer Breeding, Sichuan Institute for Drug Control, Chengdu 611845, China
[5] College of Life Sciences, Wuhan University, Wuhan 430072, China
[6] Chengdu Research Base of Giant Panda Breeding, Chengdu 611081, China
[7] College of Biological Sciences and Technology, Beijing Forestry University, Beijing 100083, China
[8] Key Laboratory of Zoological Systematics and Evolution, Institute of Zoology, Chinese Academy of Sciences, Beijing 100101, China
[9] College of Animal Science and Technology, Sichuan Agricultural University, Chengdu 611130, China
[10] College of Animal Science and Technology, Nanjing Agricultural University, Nanjing 210095 China
[11] College of Agriculture, Kunming University, Kunming 650214, China

Tao Wang, Maosen Yang, and Xin Shi contribute equally to this work.
[*] For correspondence: Diyan Li and Hang Jie. Email: lidiyan860714@163.com, jiehangisgood@126.com

ORCID iDs:
Tao Wang [0000-0002-3790-560X]; Xin Shi [0000-0001-7806-2920]; Shilin Tian [0000-0001-8958-1806]; Zhengting Zou [0000-0003-1716-5090]; Ming Zhang [0000-0003-2728-128X]; Chengli Zheng [0009-0008-9677-5076]; Chungang Feng [0000-0002-7031-4211]; Guijun Zhao [0000-0001-7710-3372]; Diyan Li [0000-0001-7490-3550]; Zhengrong Yuan [0000-0002-5175-0675]; Hang Jie [0000-0003-3491-8971];

Abstract
Background
Musk, secreted by the musk gland of adult male musk-secreting mammals, holds significant pharmaceutical and cosmetic potential. However, understanding the molecular mechanisms of musk secretion remain limited, largely due to the lack of comprehensive multi-omics analyses and available platforms for relevant species, such

as muskrat (*Ondatra zibethicus* Linnaeus) and Chinese forest musk deer (*Moschus berezovskii* Flerov).

Results

We generated chromosome-level genome assemblies for the two species muskrat (*Ondatra zibethicus* Linnaeus) and musk deer (*Moschus berezovskii* Flerov), along with 168 transcriptomes from various muskrat tissues. Comparative analysis with eleven other vertebrate genomes revealed genes and amino acid sites with signs of adaptive convergent evolution, primarily linked to lipid metabolism, cell cycle regulation, protein binding, and immunity. Single-cell RNA sequencing in muskrat musk glands identified increased acinar/glandular epithelial cells during secretion, highlighting the role of lipometabolism in gland development and evolution. Additionally, we developed MuskDB (http://muskdb.cn/home/), a freely accessible multi-omics database platform for musk-secreting mammals.

Conclusions

The study concludes that the evolution of musk secretion in muskrats and musk deer is likely driven by lipid metabolism and cell specialization. This underscores the complexity of the musk gland and calls for further investigation into musk secretion-specific genetic variants.

Introduction

Natural musk (Moschus) is mainly secreted by the musk gland located between the navel and genitals of mature male forest musk deer (*Moschus berezovskii* Flerov), an endangered artiodactyl species native to southern and central China and northernmost Vietnam [1]. Natural musk has long been an important component of traditional Chinese medicine and was used for resuscitation, blood circulation, collateral drainage, detumescence, and pain relief [2]. In addition, the muskrat (*Ondatra zibethicus* Linnaeus), a semiaquatic rodent native to North America [3] and Canada but has been introduced to Europe, Asia, South America, and Australia, has similar musk gland and secret musk likewise. The musk secreted by forest musk deer and muskrat produces a specific fragrance, whose chemical composition may be involved in chemical communication, potentially encoding information about sexual maturity and attraction [4]. The chemical composition analysis of musk showed that it contained active macrocyclic ketone components such as muscone and normuscone [5]. Muskrat musk also contains macrocyclic ketone compounds such as muscone and normuscone [6]. This class of substances is thought to be necessary for exerting drug effects. Our previous research indicated that musk of muskrat and musk deer have up to 272 identical metabolites, including organic compounds such as amino acids, fatty acids, ketones, aldehydes, and steroids [7].

Compared with other musk-secreting mammals (i.e., other musk deer species), there has been more captive breeding practice for the forest musk deer and the muskrat. The high-quality genome sequences of these two species and comparative analyses with the other mammalian genomes can potentially shed light on their genome diversity and the genetic components underlying musk secretion, which may have experienced

convergent adaptation during the long process of evolution. However, there are no convergent evolutionary studies on musk-producing animals to elucidate the related mechanism of musk secretion. In the process of evolution, it is crucial to identify which genes are subject to positive and convergent evolution. Additionally, identifying the genes that are differentially expressed in different cells of the musk gland during musk secretion is critical. The regulation of the molecular process of musk secretion by these genes from chromatin conformation remains unknown.

Here, we sequenced the genomes of a male muskrat and a male musk deer. In addition, to characterize the transcriptomic variability with respect to known tissue-specific physiological activities and identify key genes underlying the musk-secreting phenotype, we sequenced 84 RNA-seq libraries and 84 small RNA-seq libraries of 13 various muskrat organs. To accurately depict cell composition and transcriptomic changes in the musk gland of muskrat between musk secretion and non-secretion stages, we further used a single-cell RNA (scRNA) approach to dissect the transcriptional differences. We also examined the potential chromatin architecture dynamics underlying the phenotype by sequencing three and four Hi-C libraries for the musk gland, respectively, in the secretion and non-secretion stages (**Supplementary Table S1**). Integrated with these multi-omics data, the study identified particular genes, such as *SMPDL3A* and *NRCAM*, that exhibit unique patterns of genetic interactions and changes in their genomic neighborhoods, suggesting they may play key roles in controlling the musk secretion process. The research concludes that the evolutionary adaptation for musk production in both the muskrat and the musk deer is likely driven by robust lipid metabolism and specialized cell functions, underscoring the musk gland's intricate nature. This finding emphasizes the necessity for continued investigation to uncover the full functional impact of genetic variations specific to the musk secretion process.

Results and discussion

Genome assembly of two musk-secreting mammals

We sequenced the genomes of two male musk-secreting mammals [*Ondatra zibethicus* (muskrat) (2 years of age) and *Moschus berezovskii Flerov* (forest musk deer) (2.5 years of age)] via integration of Oxford Nanopore Technologies (ONT) long reads, high-throughput chromosome conformation capture (Hi-C) data and BGI T7 paired-end sequences (**Supplementary Table S1**) to over 235.25-fold (~583.41 Gb) and 246.02-fold (~696.23 Gb) coverage, respectively. We assembled the two chromosome-level genomes by applying an improved assembly method that utilizes Hi-C interaction pairs to cluster ONT long sequences with potential linkages and avoid any erroneous overlap caused by long-distance repetitive sequences during string graph assembly [8] (**see methods**). We successfully generated 2.48-Gb and 2.83-Gb for muskrat and musk deer genomes with contig N50 values of 60.53 and 69.45 Mb, which anchored onto 28 and 30 chromosomes, respectively (**Supplementary Table 1, Supplementary Table S2; Fig. 1a**). The 28 anchored chromosomes in muskrat were confirmed by karyotype analysis (**Supplementary Fig. S1a**). In particular, five chromosome sequences have

reached the gap-free level in the muskrat genome (**Supplementary Table S3**), and our two assemblies have improved the N50 length of the contig by 1,048- and 3.56-fold for the muskrat and musk deer compared to the published sequences, respectively (**Supplementary Fig. S1b-c**). Our assembled genomes exhibit excellent completeness, as evidenced by the coverage of > 99% paired-end reads across > 99% of the genome, and recovery of averaged 96.95% of BUSCOs (Benchmarking Universal Single-Copy Orthologs) [9] in 9,226 conserved mammalian genes from the mammalia_odb10 database (**Supplementary Table S4**). Furthermore, we used a reference-free and *k*-mer-based approach and estimated a high assembly quality value (QV) of more than 44, exceeding the Vertebrate Genome Project (VGP) standard of QV40 [10, 11]. Subsequently, we predicted 1013.31 Mb (40.85%) and 1539.41 Mb (54.33%) transposable elements (TEs) for muskrat and musk deer, respectively (**Supplementary Table S5**). By combining homology- and *ab initio*-based methods, aided by evidence of transcription, we identified 23,260 and 24,375 protein-coding genes in the muskrat and musk deer genomes, respectively (**Supplementary Table S5, Supplementary Fig. S1d-e**).

Table 1. Global summary of two assemblies for muskrat and musk deer

Genomic features	Muskrat	Musk deer
Assembled genome size (Gb)	2.48	2.83
Percentage of anchoring (%)	97.19	98.79
Contig Number	561	1,173
Contig N50 (Mb)	60.53	69.45
GC content (%)	41.69	42.14
Repeat ratio (%)	40.85	54.33
Predicted number of protein coding genes	23,260	24,375
QV	44.48	44.09
BUSCOs (%)	96.89	96.28

Next, we explored gene family expansion and contraction in two musk secretion species. As a result, we determined that 181 gene families underwent an expansion, and 134 underwent a contraction for the musk deer. Muskrat shows comparable numbers of gene family contraction (166) and expansion (161) events (**Fig. 1b**). GO enrichment analyses of contracted genes indicated that these two species were both involved in pathways like “Olfactory Signaling Pathway,” “Olfactory transduction,” “Signaling by GPCR,” “B cell receptor signaling pathway,” “Tight junction Immunoregulatory interactions between a Lymphoid and a non-Lymphoid cell,” “Natural killer cell-mediated cytotoxicity” and “Autoimmune thyroid disease” (**Supplementary Fig. S2a**). Gene families underwent an expansion in these two species and were involved in biological processes like “Cell Cycle,” “Meiotic synapsis,” “Mitotic Anaphase,” and “Estrogen-dependent gene expression” (P < 0.05) (all Fisher’s exact test)

(**Supplementary Table S7-8**).

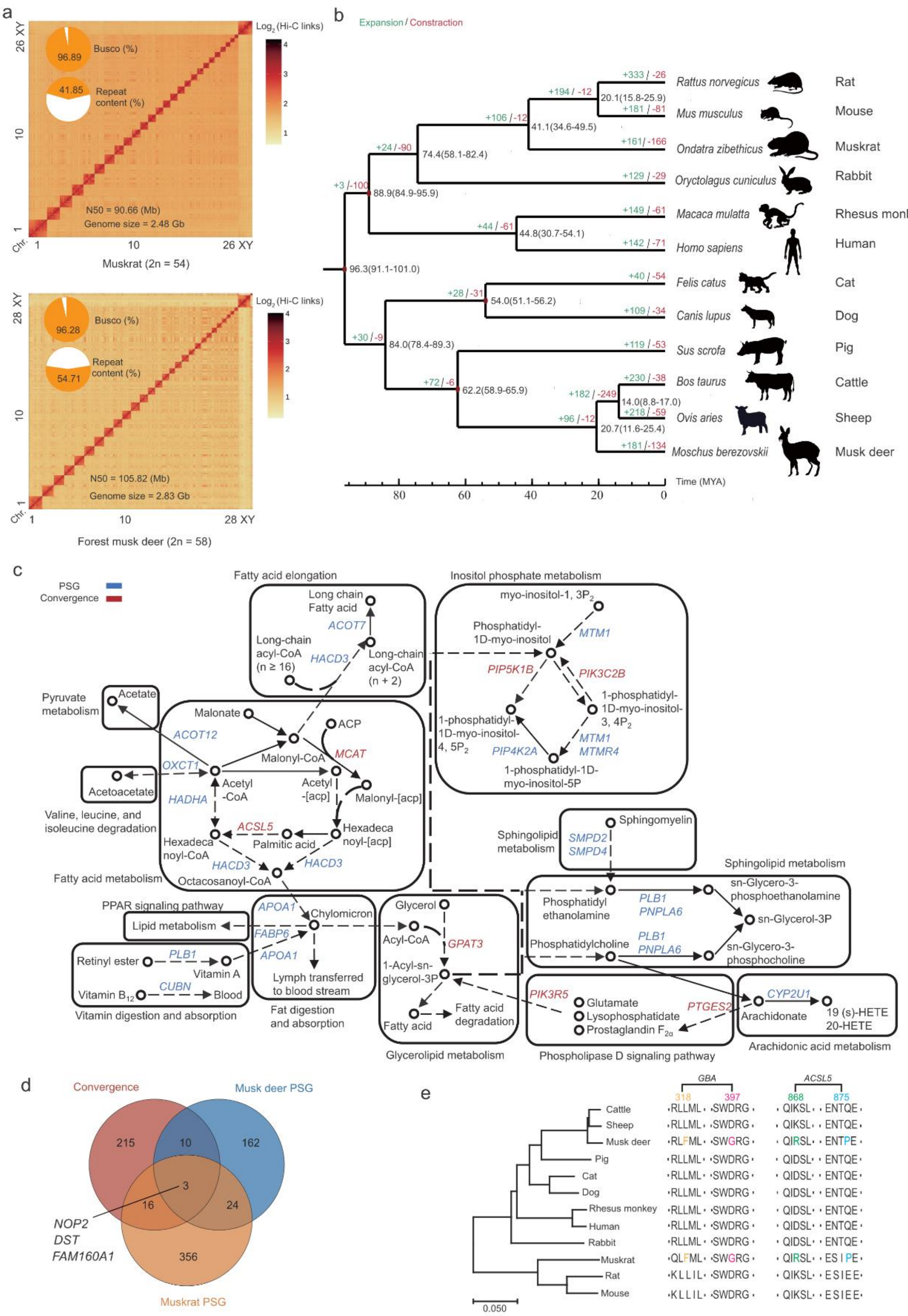

Figure 1: Genome assembly and gene family evolution in muskrat and musk deer. (a) Hi-C heatmaps for muskrat and forest musk deer. Pie charts represent the proportion of conserved BUSCO gene sets and repeat content. Contig N50 and assembled genome size are shown. (b) Divergence times and expansion and contraction of gene families in muskrat and musk deer genomes. Numbers on the nodes represent divergence times, with the error range shown in

parentheses. The numbers of gene families that expanded (green) or contracted (red) in each lineage after speciation are shown on the corresponding branch. (c) Enrichment for PSGs and convergence genes that functions in the metabolism of lipids (only partial pathways were shown). The PSGs are shown in blue, and the convergence genes are shown in red. (d) The Venn diagram shows the number of PSGs and REGs in muskrat and musk deer. (e) The sequence alignment shows sites of convergence gene in *GBA* and *ACSL5*.

Positive selection and rapid evolution genes in muskrat and musk deer were mainly involved in the metabolism of lipids and epithelial regulation

To explore the function of positively selected genes (PSGs) and rapidly evolving genes (REGs) in muskrat and musk deer during evolution. We next analyzed 7,409 gene trees based on one-to-one orthologs, each constrained to the reconstructed species phylogeny. By applying branch tests and branch-site tests in PAML [12] to the corresponding branches, we identified 443 rapidly evolving genes (REGs) and 399 positively selected genes (PSGs) for muskrats, 523 REGs, and 199 PSGs for musk deer, respectively **(Supplementary Table S6)**. Interestingly, the gene *Hdac1* was in GO term "epidermal cell differentiation," which is enriched by gene family expansion in the forest musk deer and muskrat, coinciding with the musk gland-specific gene. These respective sets of genes were mainly involved in the "Metabolism of proteins" and "Metabolism of lipids" (**Supplementary Table S9-10**). We also identified that PSGs like *ANAPC4*, *CDC16*, and *RBL2* were involved in the cell cycle pathway (**Supplementary Fig. S2b**). Muskrat PSGs (*HBEGF*, *PIGR*, *PLCE1*, *NCK2*) were enriched in the "Epidermal growth factor receptor signaling pathway", and REGs (*TGFBR3*, *BMP4*, *STRAP*, *ATF2*, *CDC73*) were enriched in "Negative regulation of epithelial cell proliferation". In addition, we deduced the convergent sites in musk secretion species (muskrat and musk deer) at each node for all the 7,409 single-copy orthologs based on the phylogenetic tree (**Fig 1b**) using two methods (JTT-Fgene [13, 14] and CCS [15]). As a result, total of 244 genes were identified under the JTT-Fgene model (FDR < 0.05, Poisson test), whereas 457 genes were identified by the CCS method [15]. 209 genes were detected as convergent evolution for muskrat and musk deer by both two methods (JTT-Fgene and CCS). We performed functional gene enrichment analyses for convergent evolution genes, and found that the gene sets were also significantly enriched for "Metabolism of proteins" ($P = 0.045$, Fisher's exact test), "Metabolism of lipids and lipoproteins" ($P = 0.035$, Fisher's exact test).

RDH8 is a convergent evolution gene involved in "The canonical retinoid cycle in rods (twilight vision)" in muskrat and musk deer. Although olfactory receptor genes are contracted in these two species, several vision-related genes were PSGs or convergent evolution genes, indicating they have a sensory trade-off otherwise observed in arboreal species [16] and giraffes [17], which is consistent with the fact that both species are

timid and sensitive. *TEX15*, a testis-specific protein, is required for TE silencing. A previous study indicated that *TEX15*, a new essential epigenetic regulator, may function as a nuclear effector of MILI to silence TEs by DNA methylation [18]. Our study suggested that the *TEX15* gene is an outlier with seven unique amino acid convergent substitutions for muskrat and musk deer (**Supplementary Fig. S2c**). This indicated that the *TEX15* gene not only plays a role in male germ cells, but it might also have an essential role in forming the male characteristic organ of a musk-secreting species.

For the “Metabolism of lipids and lipoproteins” pathway, we identified 10 convergent evolution genes (*SLC44A2, GBA, PTGES2, GPAT3, PIK3R5, ACSL5, MED12, ACBD6, PIP5K1B, BDH2*) in this pathway (*P* value = 0.035, Fisher’s exact test) in muskrat and musk deer (**Fig. 1c**). We also checked the overlapped genes between PSGs, REGs and convergent evolution genes, and found *NOP2*, *DST*, *FAM160A1* and *CKAP5* were shared between them (**Fig. 1d, Supplementary Fig. S3**). These genes have been less studied, such as FAM160A1, a member of the UPF0518 family of proteins, each containing a conserved retinoic acid-induced 16 (RAI16)-like domain with unknown biological function [19]. *CKAP5* enables the formation of persistent actin bundles on dynamic microtubules [20]. Among these convergent evolution genes, *ACSL5* and *GBA* have two amino acid substitutions (**Fig. 1e**). The protein encoded by the *ACSL5* gene is an isozyme of the long-chain fatty-acid-coenzyme A ligase family, which catalyzes the formation of fatty acyl-CoAs from long-chain fatty acids (C16–C20). Fatty acyl-CoAs are then used in lipid synthesis or β-oxidation mediated pathways [21]. The *GBA1* gene encodes the lysosomal enzyme beta-glucocerebrosidase (GCase) that degrades glucosylceramide, and is pivotal in glycosphingolipid substrate metabolism [22]. In addition, choline is essential for synthesizing phospholipids [23], and the gene *SLC44A2* participated in the process. These results indicated a rapid evolution of lipid metabolism in these two species. In addition, some convergent evolution genes that also have roles in “Metabolism of lipids” ($P = 0.0002$) and “Cell Cycle, Mitotic” ($P = 0.006$) were also detected (**Supplementary Fig. S2a**). This might be because the male musk gland has a cyclic change; in the musk secretion stage, the musk gland is atrophic, and in the stage of musk non-secretion, the glands become larger, accompanied by cell proliferation and differentiation.

Tissue-specific expression of genes in the musk gland

To explore the genes specifically expressed in the musk gland and their functions, we used a muskrat as a model animal to conduct further analyses, as muskrat tissue samples are accessible in contrast to the endangered musk deer. We first constructed 84 RNA-seq libraries and 84 small RNA-seq libraries to explore tissue-specific expression patterns from 13 tissues (two from entoderm [liver and lung], eight from mesoderm [testis, heart, spleen, kidney, muscle, fat, uterus, and ovary], and three from ectoderm

[brain, eyeball, and musk gland]) (**Fig. 2a**), with at least six biological replicates for each stage. We then updated the annotation of distinct transcript types, including lncRNAs and miRNAs (**Supplementary Fig. S4a**), representing a core atlas dataset of *de novo* assembled transcripts. There are 10 and 3 gland-tissue-specific expressed lncRNAs and miRNAs were detected, respectively (**Supplementary Fig. S4b**).

After filtering the low expression levels genes with transcripts per million (TPM) < 1 in at least 50% samples in each analyzed tissue, we evaluated the expression levels of 14,861 (63.89%) muskrat genes and mainly looked for tissue-specific expression patterns. The results showed that the transcriptional profiles of each tissue type are highly reproducible among biological replicates (Spearman's $r > 0.80$) (**Fig. 2b**). Ovary, musk gland, and uterus also clustered into obviously separate respective groups. Meanwhile, brain and eyeball tissues clustered together (**Fig. 2c**). More than 60% of genes were expressed in each tissue for muskrat (**Supplementary Fig. S4c**), but expression levels of different genes are skewed. In most tissues, the expression of highly expressed 1,000 genes takes up an average expression of more than 50%, especially for muscle (**Fig. 2d**). We also observed dissimilarities between the gene expression level distribution across tissues. The most abundant transcripts (the top 1,000, as ranked by expression levels) in a tissue, accounted for greater than half of the total transcribed muscle (~71.71%), liver (~68.76%), and heart (~61.34%), whereas testis (~37.70%) had a more uniform distribution (**Fig. 2e, Supplementary Fig. S4d**). Testis showed the highest number of differentially expressed genes compared with other tissues (**Supplementary Fig. S4e**).

In terms of protein-coding gene expression, there are 3 (uterus), 35 (testis), 27 (spleen), 6 (ovary), 26 (muscle), 9 (lung), 47 (liver), 23 (kidney), 19 (heart), 10 (gland), 8 (fat), 38 (eyeball), and 112 (brain) tissue-specific genes were detected (**Supplementary Table S11**). The tissue-specific genes were commonly enriched in distinct cellular functions. For example, the specifically expressed genes for the musk gland were mainly involved in "epidermis development" and those for testis in "male gamete generation" and "meiotic nuclear division" (**Supplementary Fig. S5**). Notably, among the genes specifically expressed in musk gland (**Fig. 2f**), *KRT80* and *MPIG6B* genes were also PSGs in muskrat. Keratin 80 (*KRT80*) is an intermediate filament protein that contributes to the structural integrity of epithelial cells [24]. The megakaryocyte and platelet inhibitory receptor gene G6P (*MPIG6B*) regulates platelet production, aggregation, and activation [25]. In addition, we also found that some musk gland-specific genes are related to lipid metabolism, such as *TRPV3* and *LIPM* (**Fig. 2f**). Transient receptor potential (TRP) channels are polymodal sensors that convert a multitude of environmental cues into cellular signaling events essential for physiology [26]. *TRPV3* is activated by warm temperatures and numerous chemicals, including plant extracts, lipid metabolites, and synthetic small molecules such as 2-

aminoethoxydiphenyl borate (2-APB) [27, 28]. In mice, *LIPM* (lipase) also has a restricted tissue expression in the epidermal tissue [29], supporting the unique and active function of *LIPM* in musk gland function.

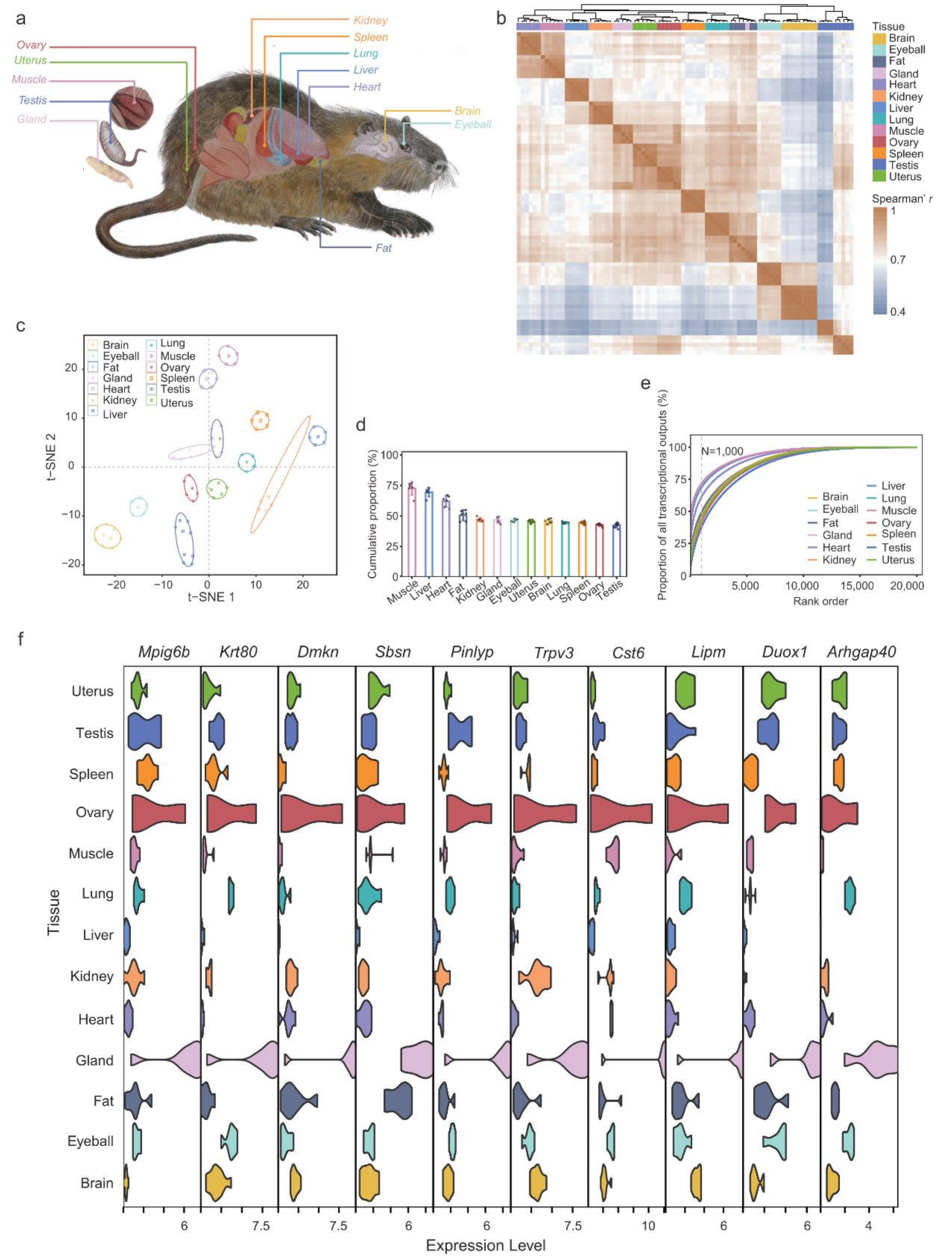

Figure 2: Characteristics of the muskrat BodyMap transcriptome. (a) Samples derived from 13 tissues were used for muskrat transcriptome reconstruction. (b) Hierarchical clustering and Spearman' *r* heatmap of samples using expression values (TPM). (c) t - distributed stochastic neighbor embedding (t-SNE) clustering of samples using expression values (TPM). The ellipses indicate the samples of the same tissue with similar transcriptional profiles, constructed at a

probability of 0.95 (*n* = 19,800). (d) The cumulative expression proportion of the top 1,000 highly expressed genes in all samples. (e) Abundance distribution of transcripts across 13 tissues. The x-axis indicates the proportion of transcripts sorted from highest to lowest expression, with the vertical dashed line indicating the top 1,000 of the highest abundance transcripts. The y-axis indicates the accumulated fraction of transcripts relative to the total transcripts. Colored lines represent mean values across different tissues. (f) The genes specifically expressed in musk gland.

Single-cell reconstruction of musk secretion remodeling in the muskrat adult musk gland

To depict transcriptomic changes during musk secretion at the single-cell level, we further measured the transcriptional differences between representative secretion and non-secretion stages in the musk gland by 10× Genomics scRNA-seq system (**Fig. 3a**). After quality filtering, the transcriptome profiles of 19,398 cells were available for cell-type characterization (12,128, and 7,270 cells for musk secretion and non-secretion stages, respectively) (**Supplementary Table S1**). To explore the cell types of these musk glands, we performed the uniform manifold approximation and projection (UMAP) analysis and identified 23 cell clusters (**Fig. 3b, c**). We surveyed the expression patterns of the top 50 most variable genes (**Supplementary Fig. S6a**), which could cluster these cells into 13 known cell types (**Supplementary Table S12**).

We found the vast majority of collected cells (49.53% and 59.16%) possess characteristics typical of fibroblasts (clusters 1, 2, 3, 4, 5, 11, and 18) with higher expression of *IGFBP3* [30, 31], *DCN* [32], and *C3* [33] (**Supplementary Fig. S6b**). Clusters 0, 10, 16, and 21 were identified as macrophages with higher expression of *C1qa*, *C1qb*, *C1qc*, *Ctss*, *Cd14,* and *Cd68* [34]; cluster 21 with higher expression of *Coro1a* [34] and *Cd74* [35, 36]. Cluster 17 was identified as mastocytes with expressed *Alox5*, *Cpa3*, *Kit,* and *Srgn* [37]gene markers. Cluster 22 expressed *Ccnb2*, *Hmgb2*, *Hmgb3*, *Mcm6*, *Ube2c,* and *Uhrf1* [38] mesenchymal progenitor cell (MPCS) markers. In addition, a recent study [39] suggested that proliferative marker genes *Top2a*, *Mki67,* and *Birc5* were markers for cluster 22 in our study. Neutrophil granulocytes (cluster 12) highly expressed *Adam8*, *Arg2*, *Anxa1,* and *C5ar1* [35]. Endothelial cells (clusters 7 and 8) expressed markers of *Plvap*, *Cav1*, *Cav2*, *Emcn*, *Gpihbp1*, *Pecam1,* and *Tm4sf1* [34]. Myoepithelial cells (cluster 6) expressed gene markers of *Acta2*, *Myh11*, *Myl9*, *Mylk*, *Tpm,* and *De* [40, 41]. T cell (cluster 9) expressed *Rapgef6*, *Ltb*, *Rpl12*, *Rplp1*, *Rps16,* and *Rps23* [35] , and *CD3* gene markers [42-45]. Basal epithelial cells (cluster 13) expressed *Ccnd2*, *Krt14* and *Krt17* markers [46]. Acinar cells/glandular epithelial cells (GEC) (cluster 14) expressed *Cited4*, *Epcam*, *Crabp2,* and *phyh2* gene markers. Smooth muscle cells (cluster 15) highly expressed *Des*, *Myh11*, *Acta2*, and *Tpm2* [40, 47]. In addition, the *PPP1R14A* gene highly expressed in cluster 15 could inhibit the myosin phosphatase, lead to increased phosphorylation of myosin, and enhance smooth muscle contraction. Cluster 20 highly expressed *SOX1*, *S100* [48], *MPZ* [49], *NCAM* [50-52], *SCN7A* and *CRYAB*. *SCN7A* is one of the many voltage-gated sodium channel proteins. *CRYAB* is highly expressed in many neurological diseases, and the protein

encoded by the *S100B* gene might play a role in Ca^{2+} flux stimulation and promoting astrocyte hyperplasia. The chromosomal rearrangement or expression change of S100B is associated with neurological diseases such as Alzheimer's disease, Down's syndrome, and epilepsy. Thus, cluster 20 cells were defined as Swann cells. Cluster 19 cells with highly expressed *Alas2*, *Bpgm,* and *Mkrn1* were identified as erythrocyte precursor cells [34].

Next, we focus on the cell clusters with increased cell abundance in the musk secretion stage (**Supplementary Table S13**), which included cluster 12, cluster 14, cluster 17, and cluster 21. Intriguingly, the DEGs in cluster 14 (**Supplementary Table S14, Supplementary Fig. S6c**) were involved in the "Regulation of hormone levels," "lipid biosynthetic process," and "organic acid transport" pathways, which are related to musk secretion (**Supplementary Fig. S6d**). In addition, for the DEGs in cell cluster 14, we also determined that five genes overlapped with REGs, including those associated with carboxylic acid transport (*SLC26A2*), transcription factor activity (*ZNF317*), cell division (*NCKAP51*) and collagen-containing extracellular matrix (*GPC4* and *COL6A3*). It is worth mentioning that *NCKAP51* is also a REG of forest musk deer. Studies in closely related species have suggested that it targets mir-2425-5p to regulate the proliferation and differentiation of bovine myogenic satellite cells [53]. *GPC4* is differentially expressed in dental epithelial and mesenchymal cells [54], also expressed in renal epithelial cells to regulate epithelial branching morphogenesis [55]. *COL6A3* encodes collagen type VI and is usually expressed in tumor epithelial cells to promote invasion and metastasis [56]. Two PSGs, cyclin (*WEE1*) and integrin ligand (*NPNT*), coincided with the differential genes of GEC. *WEE1* can regulate cell division by mediating the G2/M phase progression of epithelial cells [57]. *NPNT* was found to be highly expressed in epithelial and mesenchymal cells of the tooth germ and regulated the differentiation of Sox2+ cells in dental epithelial cells through the EGFR-PI3K-Akt signaling pathway [58]. These genes provide evolutionary evidence for the epithelial-mesenchymal transition of the muskrat musk gland during musk secretion and non-secretion.

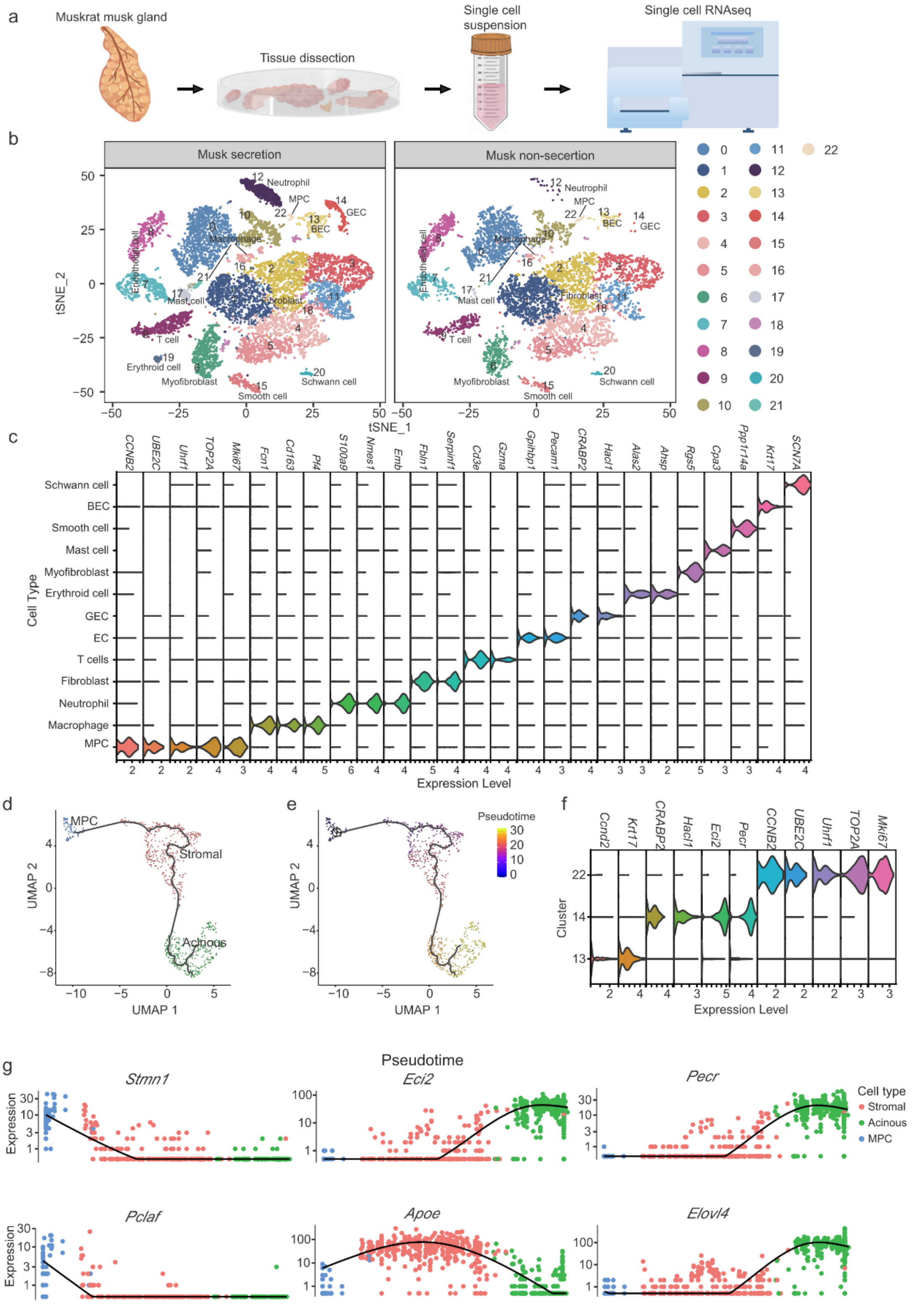

Figure 3: Musk gland single-cell transcriptome map of muskrats. (a) Schematic representation of muskrat musk gland tissue preparation for single-cell transcriptome analysis. (b) tSNE and UMAP cluster map revealing 23 specific clusters representing the major musk gland cell types. (c) Violin plots show the expression of representative differentially expressed genes for each cluster. (d-e). (d) UMAP visualization of the muskrat mesenchymal progenitor cell (cluster 22), basal cell (cluster 13), and acinar cells/glandular epithelial cells (cluster 14). Cells are color-coded by cluster. (e) Cells are color-coded by pseudotime reconstruction traces. (f) Violin plot of genes selected as population

markers for each of the 13, 14, and 22 clusters. (g) Genes that displayed divergent expression patterns during acinar cells/glandular epithelial cells' generation in muskrat.

Pseudotime reconstruction traces of the origin and specification of acinar cells/glandular epithelial cells

Our scRNA results showed that acinar cells/glandular epithelial cells (cluster 14) have an increased number of cells in the musk secretion stage (with a relative abundance of 2.76% and 0.12% in musk secretion and non-secretion stages, respectively), and this cell type is related to musk secretion. Thus, we further explored the origin and differentiation of the cells of this cluster. Acinar cells/glandular epithelial cells (cluster 14) expressed *Cited4*, *Epcam*, *Crabp2,* and *Phyh2* gene markers [34]. We used cell lineage trajectory analysis to elucidate the origin and differentiation of the acinar cells/glandular epithelial cells in muskrat. Pseudotime analysis based on transcript profiling enabled a precise reconstruction of acinar cells/glandular epithelial cells in the male muskrat gland.

The high concordance of scRNA status between cluster13 and cluster14 indicated that muskrat acinar cells/glandular epithelial cells derive directly from the basal cells, and the basal cells derive directly from the mesenchymal progenitor cell (**Fig. 3d, e**). This scenario on the biological origin of glandular epithelial cells was consistent with the scRNA of mouse mammary epithelial cells [59]. The markers for acinar cells/glandular epithelial cells (*CRABP2*, *Hacl1*, *Eci2*, and *Pecr*) of cluster 14, found by pseudotime reconstruction, displayed high and specific expressions compared with the other two clusters (**Fig. 3f, g**). These genes were mainly involved in lipometabolic functions. For example, *CRABP2* is involved in the metabolism and transportation of retinoic acid from the cytosol to the RARs (retinoic acid receptors) located in the nucleus [60]. *Hacl1* is an enzyme that catalyzes the hydrolysis of long-chain fatty acids [61]. Enoyl-CoA delta-isomerase 2 (*ECI2*) is a protein that catalyzes the isomerization of unsaturated fatty acid intermediates during beta-oxidation, a process that breaks down fatty acids to produce energy [62]. Peroxisomal trans-2-enoyl-CoA reductase (*PECR*) is a protein that plays a role in the metabolism of fatty acids, specifically by reducing unsaturated and polyunsaturated fatty acids to their saturated forms in peroxisomes [63]. A previous study showed that muskrat musk contained fatty acids (29.32%) by gas chromatography-mass spectrometry, which are the main components of musk [64]. Combined, these results indicated that the function of lipometabolism is important in the development and evolution of the musk gland.

Dynamic changes in compartmentalization, TAD, and PEI for musk secretion

In the eukaryotic cell nucleus, genomic DNA is highly folded and spatially organized into a hierarchy of 3D structures, including chromosome territories, compartments, topologically associating domains (TADs), and long-range interactions [65], which play

important roles in transcriptional regulation [66]. To elucidate the multiscale regulatory rewiring of chromatin architecture during musk secretion, we used in situ Hi-C to map chromatin contacts for musk glands between the secretion and non-secretion stages. We generated a total of ~2.77 billion valid contacts (~692.44 million [M] contacts per sample (**Supplementary Table S15**) and reached a maximum resolution of 5 kb by merging the intrachromosomal contacts of the replicates at each stage) (**Supplementary Table S16**). Most (~54.29%) contacts occurred within chromosomes, exhibited high reproducibility among the biological replicates, and consisted dominantly (~57.82%) of long-range interactions (≥20 kb) (**Supplementary Fig. S7a-d**). All samples showed a strong decrease in contact probability with increased distance between loci (**Supplementary Fig. S7e**). All samples showed similar A/B compartment patterns; ~44.4% and ~48.0% of the whole genome were Compartment A bins for musk secretion and non-secretion periods, respectively (**Supplementary Fig. S7f**). Compartment A was positively correlated with Guanine-Cytosine content (Spearman's $r > 0.60$, $P < 2.20 \times 10^{-16}$) (**Supplementary Fig. S7g-h**) and has a high gene density (**Supplementary Fig. S7i**). We then constructed genome-wide inter-chromosomal contact maps by dividing the genome into 500-kb regions; it was revealed that the muskrat chromosomes have a similar likelihood to mutually contact each other during development: micro- and macrochromosomes tended to be self-associated, small and gene-rich chromosomes preferentially contacted with each other more frequently (**Fig. 4a**). A total of 2,969 and 3,438 TADs were subsequently detected in musk secretion and non-secretion stages in the musk gland, with a median size of ~500 and 575 kb respectively (**Supplementary Fig. S7j**). We observe that only 54% (2,256) of the positioning of TADs remains stable between the two stages (**Supplementary Fig. S7k**). We also compiled an extensive genome-wide catalog of PEIs in musk gland tissue at a 5 kb resolution. The median sizes were ~100 and ~60 kb (54.67% and 63.11% PEIs existed primarily in TADs) for musk secretion and non-secretion stages respectively (**Supplementary Fig. S8a-c**). We observed that ~87.98% of enhancers interacted with a more distant promoter instead of those closer by (**Supplementary Fig. S8d**). This spatial proximity data highlights the complexity of PEIs [67].

Next, we compared the 3D genome differences in the musk gland between the two stages. At the sub-chromosome level, we identified substantial number of regions showing compartmental switching in the musk gland between two stages (~153.6 Mb, or ~6.2% of the genome) (**Fig. 4b**). In these regions, most switching was from A to B (120.80 Mb, embedded with 781 genes), which indicated that these regions were more closed in the musk secretion stage compared with the non-secretion stage. The rest were transient switches, from B to A (32.8 Mb, embedded with 164 genes) (**Fig. 4b, c**). Because these active chromatin regions are of potential functional significance, we further checked the genes in areas subject to B-to-A switching events. They were primarily involved in "epithelial cell differentiation," "nephron development," "epidermis development," "cytoplasmic translation," "extracellular matrix organization," "negative regulation of endopeptidase activity," "cell morphogenesis," "protein activation cascade," "epithelial cell development," "intracellular steroid

hormone receptor signaling pathway" and "regulation of membrane potential" processes (**Fig. 4d**).

At the TAD structure level, many changes in chromatin structure occur. We used the insulation score (IS) to evaluate the overall extent of changes in chromatin conformation between the two stages. For the TAD with increased IS in the musk secretion stage, the content genes were involved in the "Regulation of proteolysis," "Response to radiation," "Regulation of lipid metabolic process," and "Transcription coregulator activity" pathways (**Supplementary Fig. S9a**), which indicated that the active function of regulation of lipid metabolism in the musk secretion stage. Furthermore, the PSGs or convergent evolution genes (**Supplementary Fig. S9b**) involved in TAD changing were also related to the metabolism of lipids and epithelial regulation pathways, such as *NAGS* catalyzing the production of N-acetylglutamate (NAG). This vital substance regulates urea synthesis [68]. *CEP250* plays a crucial role in differentiating spermatogonia and meiotic spermatocytes [69]. *CASP8AP2* plays a role in regulating cell proliferation, apoptosis, and gene expression [70].

At the PEI (promoter-enhancer interaction) level, we determined that there were two genes (*SMPDL3A* and *NRCAM*) with specific PEI in musk secretion stage, also showed a compartment transition from B to A and was a marker gene in Cluster 14 (**Fig. 4e**). There were more specific long-range interactions (> 25Kb) in musk secretion stage for these two genes (**Fig. 4f**). The gene *SMPDL3A* showed a relative higher expression in all clusters at the musk secretion stage (**Fig. 4g**). *SMPDL3A* (sphingomyelin phosphodiesterase acid-like 3A) is an enzyme induced by lipid metabolism through liver X receptor that degrades cGAMP, modulating the cGAS-STING pathway which is involved in immune responses and lipid sensing [71]. *NRCAM* (neuronal cell adhesion molecule) primarily associated with neural development, its expression also promotes malignant cell transformation, cell motility, and metastatic disease [72]. In addition, *MAP3K1*, *NODAL,* and *Slc38a2* also showed significantly more PEIs and were contacted with more enhancers during the musk secretion stage (**Supplementary Fig. S10**). *MAP3K1* is a critical component of the protein kinase signal transduction cascade and plays a crucial role in cellular signaling pathways [73]. *NODAL* maintains stem cell pluripotency and promotes directed differentiation [74]. *Slc38a2* encodes an amino acid transport protein facilitating cellular uptake of amino acids [75]. These results suggest that two essential functions of the musk gland during the musk secretion stage are lipid metabolism and cell specialization, which indicate that synthesis and secretion activity were very active at this stage. Our results demonstrated that candidate loci can be analyzed in future studies of musk secretion mechanisms.

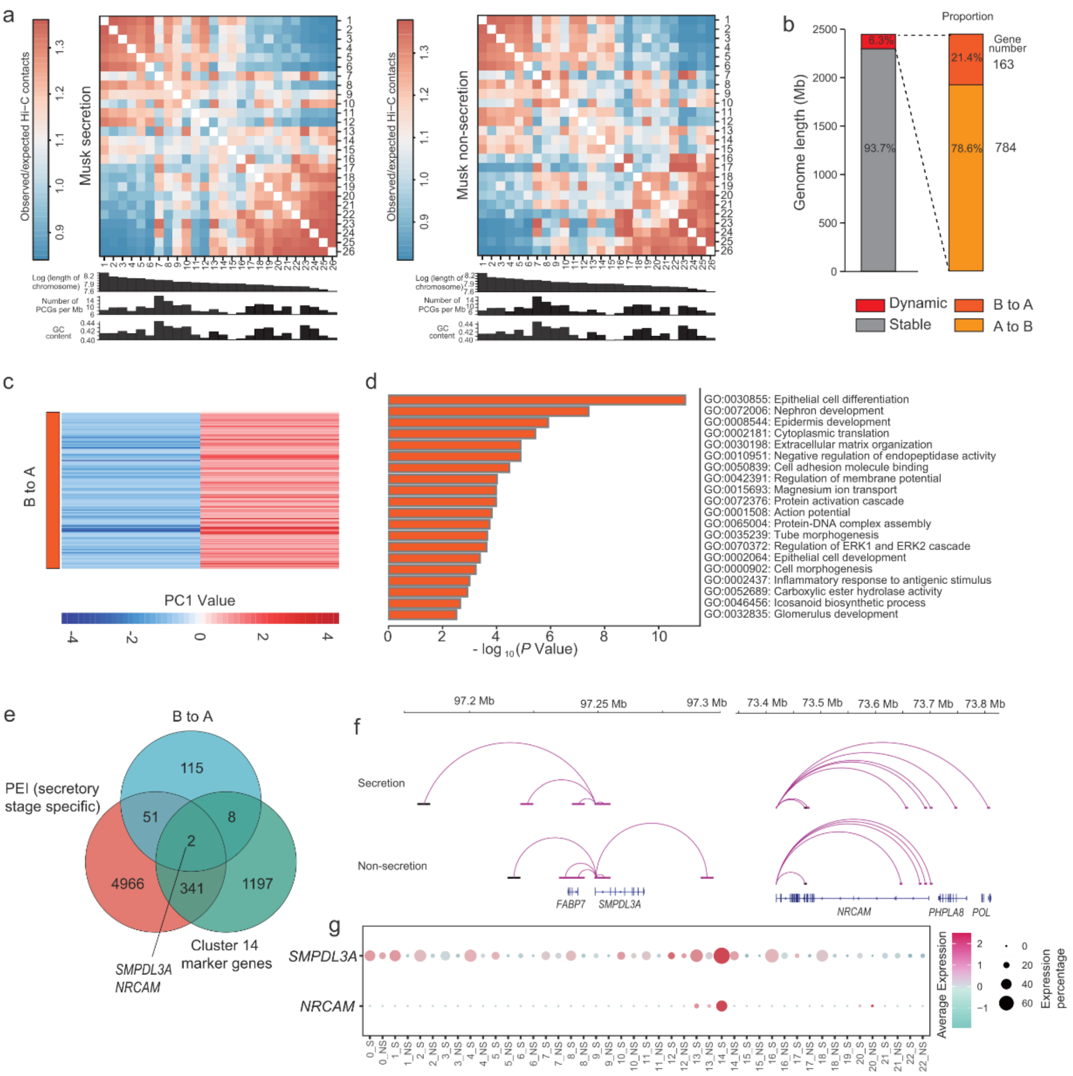

Figure 4: Global chromatin interaction patterns in musk gland of musk secretion and non-secretion stages. (a) Observed/expected contact matrices between chromosome pairs. Also shown are the length, gene density, and GC content of each chromosome. (b) Genomic lengths and proportions of stable and dynamic compartments. Dynamic compartments are classified into two types of transitions (A to B, and B to A). (c) Heatmap of the PC1 values for the compartment B to A switching regions. (d) The most enriched GO-BP terms for genes within B to A switch regions. (e) Veen diagram showing overlapping genes with compartment B to A, PEI specific to musk secretion stage, and marker genes in Cluster 14. (f) Promoter-enhancer interactions (PEIs) rewired in the musk gland of musk secretion and non-secretion stages of *SMPDL3*A and *NRCAM*. (g) Gene expression of *SMPDL3*A and *NRCAM* in each cluster at musk secretion and non-secretion stages.

The MuskDB platform

According to the sequencing data described above, we generated a database (MuskDB). MuskDB contains 47,635 gene entries from the genomes of two species, with information on 831 biological pathways,103 bulk RNA transcriptomes, two single-cell transcriptomes, and three Hi-C datasets. The platform also holds three Hi-C data sets (secretion stage in July and non-secretion stage in October for the muskrat musk gland

and blood sample of musk deer), including 343,392 promoter and enhancer interactions. On the homepage, the tools include “Blast,” “Sequence Fetch,” “Gene Sequence Extraction,” “Transposable Elements,” “Gene Synteny Viewer,” “Phylogenetic Tree,” “Gene Expression,” “Single Cell Expression” and “Hic Search” (**Fig. 5a**). MuskDB provides comprehensive information on muskrat and musk deer genes, including their annotation, location and expression. The heat maps in ‘Gene Expression’ and ‘Single Cell Expression’ show the expression of genes respectively in 13 tissues (**Fig. 5b**) and 13 different cell types (**Fig. 5c**). In ‘Hic Search,’ users could enter a gene name or genome region to show the contact information of this gene or in this region. For example, when the gene *Synpo2* is entered, the results display the contact profile of this gene at the stages of secretion and non-secretion (**Fig. 5d**).

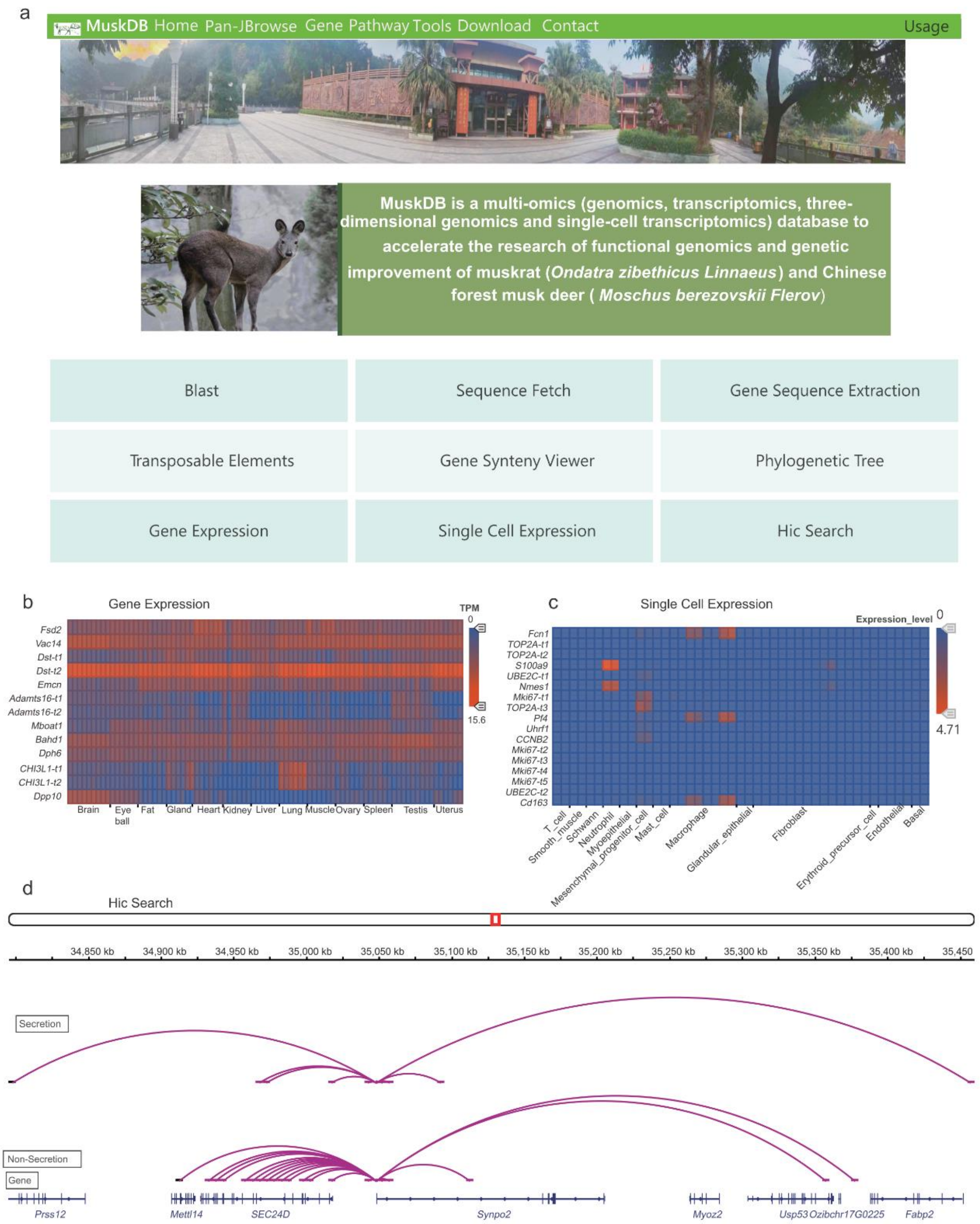

Figure 5: Overview of MuskDB and its application in musk secretion animal's functional genomics. (a) MuskDB homepage contents show available online tools. (b) The expression pattern of genes in different tissues is shown by MuskDB. (c) The expression pattern of genes in various cell clusters is shown by MuskDB. (d) Hi-C contacts of gene *Synpo2* in musk secretion and non-secretion stage shown by MuskDB.

Conclusion

This study provided an open-source, web-accessible, user-friendly multi-omics database platform of musk secretion animals, including two high-quality genome

assemblies of musk secretion species (muskrat and musk deer), a BodyMap transcriptome of muskrat, and comprehensively analysis of the Hi-C, RNA-seq, and scRNA-seq between musk secretion and non-secretion stages of muskrat *in vivo*. GO terms of stage-specific signature genes of musk glands were identified by scRNA-seq and emphasized significant functional differences between the musk secretion and non-secretion stages. Because of their unique evolutionary adaptations, the muskrat and musk deer have always been a topic of interest in animal evolution and physiology. Our high-quality genome assembly provides more precise comparative insights into the genetic basis of their biological features. We identified genetic changes underlying adaptations of both species to the musk secretion, notably related to the adaptation of lipid metabolism, cell cycle regulation, and sensory perception. Our single-cell RNA sequencing results may potentially facilitate the development of new strategies for the organoid culturing of the musk gland. These results show that active lipid metabolism may underlie the adaptation evolution of musk secretion. However, because of the complexity of the musk gland, more research on the functional consequences of musk secretion-specific genetic variants is perspectively needed.

Materials and methods

Methods

Sample collection

For genome sequencing, blood from a male muskrat and a male forest musk deer were collected from the Chongqing Institute of Medicinal Plant Cultivation. To fully investigate the muskrat transcriptome, we used 84 samples from 13 tissues (3 to 6 samples from each of the 13 tissues were collected). The healthy muskrats were isoflurane euthanasia; we adjusted the isoflurane flow rate or concentration to 5% or greater and continued isoflurane exposure until one minute after the muskrat's breathing stopped. The required tissues were then collected using standard anatomical techniques, and excess blood vessels and fat were removed in phosphate buffer solution and then flash-frozen in liquid nitrogen and stored in a -80°C refrigerator for subsequent studies.

Genome sequencing

Blood high-quality DNA was extracted from 20 mL of muskrat and forest musk deer blood using a DNeasy Blood & Tissue Kit (Qiagen, Valencia, USA) according to the manufacturer's instructions. Some of this DNA was then used to construct Nanopore libraries, which were sequenced using GridION X5 sequencers (Oxford Nanopore Technologies, Oxford, UK), and the remainder of the DNA was used to construct re-sequencing (NGS) libraries with an insert size of 400 bp, which were sequenced using the BGI T7 platform. The musk gland samples at musk secretion and non-secretion stages were used to construct Hi-C libraries, which were subsequently sequenced using

the Illumina NovaSeq 6000 platform (Illumina; RRID:SCR_016387).

Genome assembly and assessment

We used an optimized four-step genome assembly strategy [8] to generate complete assemblies of muskrat and musk deer genomes. First, we used the high-quality Nanopore sequences and applied a 'correct-then-assemble' strategy using NextDenovo (v2.5.0; RRID:SCR_025033) [76] to assemble the initial contigs. To correct the initial contigs, we utilized the software NextPolish (v1.4.1; RRID:SCR_025232), employing high-quality T7 paired-end reads and Nanopore long reads, and applied the recommended algorithm modules "best." in Nextpolish [77]. The specific parameters and pipelines used in the assembly process are available at Zenodo [78]. Thus, we yielded the ContigV1 assembly. Second, we generated unique mapped pairs by aligning the Hi-C read pairs to ContigV1 using Bowtie2 software (RRID:SCR_016368) [79] with a single-ended model. We then discarded invalid self-ligated and unligated fragments using the HiCUP pipeline (version 0.8.0; RRID:SCR_005569) [80]. We obtained the valid interaction pairs and calculated linkage frequency among all contigs using an agglomerative hierarchical clustering algorithm. The linked contigs were clustered based on the Hi-C signal density, indicating potential homologous chromosome associations. Third, we realigned the Nanopore reads to ContigV1 using package Minimap2 (RRID:SCR_018550) [81]. Suboptimal alignment reads were removed, and mapped reads of each contig group were extracted. Local assembly was performed for each classified mapped read to avoid false overlap relationships caused by repetitive sequences during assembly [82]. All contigs were corrected again using appropriate parameters, similar to the first step. Fourth, chromosome-scale genomes were anchored using linkage information, restriction enzyme site, and string graph formulation with the ALLHiC algorithm (RRID:SCR_022750) [83]. Any placement and orientation errors displaying distinct chromatin interaction patterns were manually adjusted.

To assess the quality of genome assemblies, we used the Merqury package to evaluate assembly precision by measuring QV values [10]. BUSCO analysis (version 5.2.1; RRID:SCR_015008) [9] was used to assess the assembly completeness by searching against 9,226 conserved mammalian genes from the mammalia_odb10 database. Additionally, T7 paired-end reads were aligned to the assembled genome using BWA software (RRID:SCR_010910) [84] to calculate the alignment ratio and coverage depth, for assessing the assembly completeness.

Transposable element (TE) annotation

We predicted the genome TEs by combining homology searching and *ab initio* prediction methods. We performed the homology searching by applying the RepeatMasker (RRID:SCR_012954) [85] and RepeatProteinMask packages to compare the genome against the Repbase TE library. In parallel, we performed *ab initio*

prediction by constructing a reference repeat library using the results from PILER (RRID:SCR_017333) [86], LTR_FINDER (RRID:SCR_015247) [87], and RepeatScout (RRID:SCR_014653) [88]. Then, we searched the genome against this library using RepeatMasker. In addition, we employed the Tandem Repeats Finder package (RRID:SCR_022065) [89] with specific parameters ("2 7 7 80 10 50 2000 -d –h") to predict tandem repeats in the genome.

Protein-coding gene prediction

We predicted protein-coding gene models via integration of homology- and *ab initio*-based methods, with additional evidence from transcription data. In the homology-based approach, we used protein repertoires from model mammalian species such as *Homo sapiens* (GCA_000001405.28), *Mus musculus* (GCA_000001635.8), *Equus caballus* (GCA_002863925.1), *Canis lupus familiaris* (GCA_000002285.2), *Rattus norvegicus* (GCA_015227675.2), *Sus scrofa* (GCA_000003025.6) and *Bos taurus* (GCA_002263795.2) were used as queries. These queries were searched against the target genome using the TBLASTN algorithm (RRID:SCR_011822) [90]. The resulting BLAST hits were conjoined using the Solar (Sorting Out Local Alignment Results) to obtain a comprehensive set of alignments. Next, the gene structures within each BLAST hit were determined using the GeneWise pipeline (RRID:SCR_015054) [91], allowing us to define gene models with high accuracy and specificity. Subsequently, we obtained transcriptomic data for muskrats and musk deer from the NCBI database, with accession numbers listed in **Supplementary Table S1**. The RNA-seq data were aligned to the genome using the Tophat software (RRID:SCR_013035) [92], enabling the identification of potential exonic regions and splicing junctions. Gene models, represented by the Cufflinks-set, were assembled from the mapped reads using Cufflinks (RRID:SCR_014597) [93]. In the *ab initio* method, we aligned the assembled transcripts with the assembled genome using the Program to Assemble Spliced Alignment (PASA) (RRID:SCR_014656) [94]. This allowed us to assemble the transcript alignments into gene structure models used as the training set for Augustus (RRID:SCR_008417) [95], SNAP (RRID:SCR_007936) [96], and GlimmerHMM (RRID:SCR_002654) [97] pipelines. With these training sets, we conducted ab initio prediction of coding regions in the repeat-masked genome using Augustus, GlimmerHMM, and SNAP. Furthermore, we employed GeneID (RRID:SCR_021639) [98] and GeneScan (RRID:SCR_023400) [99] to directly generate predicted gene models in the repeat-masked genome. After completing the aforementioned methods, the generated gene models were integrated using EVidenceModeler (RRID:SCR_014659). We assigned weights to each type of evidence as follows: Homology-set > Cufflinks-set > Augustus > GeneID = SNAP = GlimmerHMM = GeneScan. Furthermore, we used PASA2 to update the gene models, incorporating untranslated regions and information on alternative splicing variations.

To annotate the protein-coding genes, we searched for functional motifs, domains, and information on the possible biological processes of the genes in established databases such as SwissProt (RRID:SCR_021164) [100], the NR database (from NCBI), and the KEGG (Kyoto Encyclopedia of Genes and Genomes) (RRID:SCR_012773) [101].

Identification of one-to-one orthologous genes

In addition to the gene sets generated from our two assembled genomes, we also downloaded gene sets from 10 other mammalian genomes, including human, macaque, mouse, horse, cattle, sheep, pig, cat, dog, and rabbit, from the Ensembl database (RRID:SCR_002344). We used these 12 gene sets to identify orthologous genes. To accomplish this, we first selected the longest translation to represent each gene and filtered our genes with fewer than 50 amino acids. Then, we performed an all-against-all BLASTP (RRID:SCR_001010) comparison with an E-value threshold of 1e-7 to determine similarities between genes across the 12 species. We extracted alignment pairs from each pair of genomes while restricting a maximum of five hits per protein sequence. These alignment pairs were used as input for the MCScanX algorithm (RRID:SCR_022067) [102], which helped detect collinear blocks of coding genes and identify orthologous gene pairs with high confidence. We specifically focused on one-to-one orthologous genes between pairs of mammalian species.

After integrating a matrix of orthologous genes for the 12 mammalian species, we ensured each orthologous cluster included all the species. Subsequently, we performed multiple sequence alignment for these one-to-one orthologs using PRANK (v.170427; RRID:SCR_017228) [103] . We applied the Gblocks package (v0.91b) (RRID:SCR_015945) [104] to minimize the impact of alignment errors and divergent regions. Alignments shorter than 90 nucleotides were discarded to maintain quality. Through this process, we identified 7,409 one-to-one orthologs among the 12 species.

Phylogeny construction and divergence time estimation

We initially used MODELTEST [105] to analyze the codon alignments of one-to-one orthologs and determined that the general time-reversible (GTR) substitution model was the most suitable for the observed data. Therefore, we conducted a phylogenetic tree for the 12 mammals using the maximum likelihood method implemented in the RaxML package (RRID:SCR_006086) [106]. The best-fitting substitution model "GTR+GAMMAX" was utilized, and 1,000 bootstrap replicates were performed to assess the robustness of the tree topology. To estimate divergence times, we employed the MCMCTree program from the PAML package (version 4.9; RRID:SCR_014932) [12].

Gene family clustering, expansion, and contraction analysis

We utilized the OrthoFinder package (v2.3.1; RRID:SCR_017118) [107] to identify gene families by detecting orthogroups and paralogous genes based on the results of

the all-against-all BLASTP analysis (see also the method "Identification of one-to-one orthologous genes"). The expansion and contraction of gene families were evaluated by comparing cluster sizes between the ancestral species and each of the 12 mammalian species. This analysis used the Café program (RRID:SCR_005983) [108], which employs a probabilistic graphical model. Using conditional likelihoods as test statistics, we calculated *P-values* for each lineage and set a threshold of $P < 0.05$ to determine gene families that exhibited significant expansion or contraction.

Identification of positively selected genes (PSGs) and rapidly evolving genes (REGs)

We employed the CodeML program in the PAML package (version 4.9; RRID:SCR_014932) [12] to identify PSGs and REGs based on the 7,409 orthologous genes. For PSGs, we utilized the free-ratio branch-site mode (model = 1) as an alternative model, assuming positive selection on the foreground branch. The null model allowed sites to undergo purifying selection or evolve neutrally. For REGs, we utilized the branch model, specifically the one-ratio model (model = 0) as the null model assuming the same evolutionary rate for all branches, and the two-ratio model (model = 2) as an alternative model allowing different evolutionary rates for the foreground branch. The likelihood ratio test (LRT) method was used to detect differences between the nested models, and *P-values* were computed based on $\chi 2$ statistics. Multiple testing was corrected using the false discovery rate (FDR) method.

Convergent evolution among musk secretion animals

To test for convergence among musk secretion animals (muskrat and musk deer), we used 12 mammals from Fig. 1b. Based on the phylogenetic tree, we used two methods to detect the convergent amino acid substitutions for each node of the 7,409 single-copy orthologs: 1) method of Zhang and Kumar [13, 14], a site was assumed as a convergent site if amino acids of a focused node at that site are the same but different with their most recent ancestral amino acids. Amino acid sequences of internal nodes for all the 7,409 single-copy orthologs were reconstructed by CODEML in PAML. For each gene, the number of observed convergent site was compared with the neutral expectations derived from the JTT-fgene model, and the Poisson test was then used to evaluate the difference. 2) CCS method [15], the convergent signal is identified when all two musk secretion species (muskrat and musk deer) share the same derived character at a conservative site.

Functional enrichment analysis

The gene set enrichment analyses, including Gene Ontology (GO), KEGG pathway, and Reactome analyses, were performed using the KOBAS 3.0 software (RRID:SCR_006350) [109, 110], with human homologs as references. The statistical significance of enrichment was assessed using the binomial distribution test, and the P-values were adjusted for multiple testing using the Benjamini method.

Muskrat transcriptome reconstruction

Total RNA was extracted from each sample using RNAiso Plus reagent (TaKaRa, #9108) according to the manufacturer's instructions. We estimated the integrity and quality of the total RNA using a Bioanalyzer 2100 system (Agilent Technologies, Palo Alto, CA, USA; RRID:SCR_018043) and an RNA 6000 Nano kit. Eighty-four poly-A RNA-seq libraries were constructed. LncRNAs were then sequenced using the NOVAseq-6000 platform with a paired-end sequencing length of 150 bp (PE150) at Shenggong Bioengineering Co., LTD (Shanghai, China). MicroRNA was sequenced by the NEXTSEQ550 platform (RRID:SCR_016384) with a single-end sequencing length of 75 bp (SE75) at Shenggong Bioengineering Co., LTD (Shanghai, China). In total, we then generated a total of 1.37 Tb high-quality RNA-seq data (~16.33 Gb sequences per sample) and 27.13 Gb high-quality miRNA data (~0.32 Gb sequences per sample) (**Supplementary Table S1**).

Clean reads were obtained after quality control filtering. Using FASTX-Toolkit software (RRID:SCR_005534) to remove the clean and low-quality reads (quality value less than 30 bases accounted for more than 20%). The lncRNA sequenced reads were aligned to our self-assembled muskrat genome by the STAR alignment tool (version 2.6.0; RRID:SCR_005622), with, on average, ~97% (~57.06 million) of aligned reads for each library. Read counts were quantified using featureCounts (version 2.0.1; RRID:SCR_012919). Gene-level transcript abundance was estimated as transcripts per million (TPM).

For miRNA analysis, human, mouse, and rat miRNA and miRNA precursor sequences were downloaded from miRBase (Version 22.1; RRID:SCR_003152). The software miRdeep2 (version 0.1.2; RRID:SCR_010829) was used to first index the muskrat genome, and then the data we had initially processed were converted into the format required by the software and compared with the muskrat genome. Finally, the sequences were compared with known miRNA sequences and miRNA precursor sequences. Combined with the position of genome alignment, the matching degree was calculated to form the minimum free energy of stem-ring miRNA, to identify whether it is a muskrat miRNA. The prediction of new miRNA is similar. Mature miRNAs of muskrat relatives, such as humans, mice, and rats, were selected as a reference, and mirdeep2.pl of the software was used for prediction. The screening criteria for new miRNA should meet the miRDeep2 Score25 and have a secondary structure with p-value < 0.05 as candidate miRNA. Then, the information of new miRNA predicted by all samples should be counted, and the candidate new miRNA predicted by at least two samples should be considered new miRNA. The number of miRNA reads between samples was counted by TPM (Tags per million), and its expression was calculated.

Gene transcriptional profiling across tissues

We calculated the tissue specificity of gene abundance reflected by the tau score (τ)

(ranging from 0 to 1, with 1 for highly tissue-specific genes and 0 for ubiquitously transcribed genes) for each gene with scaled TPM values [111]. For each tissue, we averaged all replicates and then calculated τ to account for unequal numbers of replicates among tissues. We used $\tau \geq 0.75$ as the cut-off for tissue-specific genes. We calculated the abundance distribution (i.e., transcriptome complexity) of distinct transcripts across tissues, reflected as the fraction of total RNAs contributed by the most highly expressed genes. Differential gene expression analysis was performed using edgeR (version 3.40.2; RRID:SCR_012802) [112], with a Benjamini &. Hochberg adjusted P value ≤ 0.01 and $\log_2$(fold change) ≥ 1 as cut-offs for statistical significance.

Single-cell preparation

After harvesting, musk gland tissues were washed in ice-cold RPMI1640 and dissociated using Demonstrated_Protocol_Adult_Mouse_Nuclei_Isolation_RevA. (10× Genomics Catalog No.CG000393 Rev A) from Miltenyi Biotec as instructions. DNase treatment was optional according to the viscosity of the homogenate. Cell count and viability were estimated using a fluorescence Cell Analyzer (Countstar® Rigel S2) with AO/PI reagent after the removal of erythrocytes (Miltenyi 130-094-183). Then, debris and dead cell removal was performed or not (Miltenyi 130-109-398/130-090-101). Finally, fresh cells were washed twice in the RPMI1640 and then resuspended at 1×10^6 cells per mL in 1×PBS and 0.04% bovine serum albumin.

Single-cell RNA-seq library construction and sequencing

ScRNA libraries were prepared using Chromium Next GEM Single Cell 3′ Reagent Kits v3.1 (10× Genomics). Briefly, the appropriate number of cells were mixed with reverse transcription reagent and then loaded to the sample well in Chromium Next GEM Chip G. Subsequently, Gel Beads and Partitioning Oil were dispensed into corresponding wells separately in the chip. After emulsion droplet generation, reverse transcription was performed at 53 °C for 45 minutes and inactivated at 85 °C for 5 minutes. Next, cDNA was purified from a broken droplet and amplified in the PCR reaction. The amplified cDNA product was then cleaned, fragmented, end-repaired, A-tailed, and ligated to the sequencing adaptor. Finally, the indexed PCR was performed to amplify the DNA representing the 3’ polyA part of expressing genes, which also contained a Cell Bar code and a Unique Molecular Index. The indexed sequencing libraries were cleaned with SPRI beads, quantified by quantitative PCR (KAPA Biosystems KK4824), and then sequenced on Illumina NovaSeq 6000 with PE150 read length.

Single-cell RNA sequencing data quality control

Fastp (v0.20.1; RRID:SCR_016962) [113] was used to trim primer sequence and low-quality bases of raw reads and collect the basic statistics. The specific parameters could be summarized as below: (1) A 4 bp sliding window was moved from the front (5') to tail. Once the mean quality of the bases in the window was below 10, the bases, along

with the subsequent bases, would be dropped, the leading N bases were also trimmed (--cut_front --cut_front_window_size 4 --cut_front_mean_quality 10); (2) A 1 bp sliding window was moved from tail (3') to front. The bases in the window were dropped if its mean quality was below 3, the trailing N bases were also trimmed, similar to the Trimmomatic TRAILING method (--cut_tail --cut_tail_window_size 1--cut_tail_mean_quality 3); (3) The auto adapter was detected for PE data (detect_adapter_for_pe); (4) The trimmed Reads shorter than 60 bp were discarded (--length_required 60). The cleaned reads after trimming were used in the following steps.

Processing the single-cell RNA sequencing data

The Cell Ranger (RRID:SCR_017344) [114] Single-Cell Software Suite performed sample demultiplexing, barcode processing, and single-cell 3' gene counting. The single-cell data from the two stages were analyzed jointly.

The clustering and visualization were finished by Seurat (RRID:SCR_007322) [115], with the following steps: (1) Data normalization. LogNormalize, a global-scaling normalization method, was employed to normalize the expression. The expression measurement of one transcript was divided by those of all the transcripts of the cell and multiplied by a scale factor (10,000 by default), and then the result was logarithmically transformed. (2) Detection of highly variable features. FindVariableFeatures was used to get 2,000 features per dataset. (3) Scaling. A linear transformation ('scaling'), a standard pre-processing step before dimensional reduction techniques, was applied. (4) Dimensional reduction. PCA on the scaled data was performed, and the first 15 principal components were used in the following steps. (5) Clustering. A graph-based approach was applied to cluster the cells. (6) tSNE/UMAP. The non-linear dimensional reduction technique was used to visualize and explore these datasets. (7) Cluster markers. FindAllMarkers with the default parameters except "logfc.threshold=1" was used to find markers that determined the cell clusters via the differential expression, and the top 9 markers were visualized.

Pseudotime analysis

In R, utilizing the Monocle3 software package (v1.3.1; RRID:SCR_018685) (Cao et al., 2019), pseudotime trajectories for all 13, 14, and 22 subpopulations in the musk gland were constructed. This technique sorts individual cells along their developmental paths based on how closely their gene expression patterns match those of other sequenced cells, effectively mapping out the dynamics of cellular changes [116, 117]. Furthermore, the method evaluates how genes work together in space by using Moran's Index to measure their co-expression, which helps to establish a timeline of gene expression changes.

Identification of differentially expressed genes (DEGs) in Cluster 14 cells

DEGs in the Cluster 14 cells of musk secretion and non-secretion stages were identified using the FindMarkers function in Seurat. Genes meeting the thresholds ($|\log_2 FC| > 1$, P-adjusted < 0.05, min.pct = 0.25) were considered as DEGs.

The results were visualized using dot plots.

Hi-C data of musk gland tissue

Hi-C data processing

We utilized Juicer (RRID:SCR_017226), an efficient open-source tool [118], to process the Hi-C datasets. Initially, we aligned the high-quality Hi-C reads to the genome using the BWA-mem module. Subsequently, we eliminated abnormal, duplicate, and low-quality alignments (MAPQ < 30). We constructed a normalized contact matrix using the KR algorithm at different resolutions, including 5Kb, 25Kb, 100Kb, 500Kb, and 1Mb.

Resolution evaluation of the Hi-C matrix

To determine the optimal resolution for our Hi-C matrix, we divided the genome into window sizes ranging from 1Kb to 1Mb. For each bin, we counted the number of *cis* contacts, defined as any contact where one read mapped within that bin, and calculated the percentage of bins with contacts greater than 1000. We identified the minimum window size with a percentage greater than 80 as the optimal resolution for our Hi-C matrix.

Identification of compartment A/B at the resolution of 100 Kb and 25 Kb

At a resolution of 100 Kb, compartment A/B analysis was performed as previously described. Briefly, a Pearson correlation matrix was generated using the 'cor' function in R. The first three principal components were obtained by applying the 'prcomp' function in R to the correlation matrix. Bins at 100 Kb with positive Spearman's correlation between PC1 values and gene density were classified as compartment A, while bins with negative correlation were classified as compartment B.

For compartment A/B identification at a resolution of 25 Kb, the A-B index value was used as previously described, representing the comparative likelihood of a sequence interacting with A or B at a resolution of 100 Kb. Bins at 25 Kb with positive values (indicating a greater association with A at 100 Kb) were identified as A compartments, while bins with negative values (indicating a greater association with B at 100 Kb) were identified as B compartments.

Identification of inter-chromosome interaction pattern

The patterns of inter-chromosome interactions were calculated following the previous protocol [119]. In brief, the observed number of contacts was normalized against the expected contacts in each inter-chromosome pair.

Identification of TAD

We identified TADs from the normalized contact matrix at a resolution of 25 Kb. We used the directionality index (DI) score and a Hidden Markov Model (HMM) algorithm implemented in the TADtool software with default parameters [120] to assign TAD boundaries.

Promoter-enhancer interaction (PEI) analysis

We first combined the clean data of biological replicates and constructed the normalized contact matrix at 5 Kb resolution. We then used PSYCHIC software to generate raw PEIs [121]. We then filtered low-confidence PEIs with interaction distances lower than 10 Kb or FDR greater than 0.001.

Availability of Source Code and Requirements

Project name: Paper_scripts_muskrat_muskdeer

Project homepage: https://github.com/YMSen/Paper_scripts_muskrat_muskdeer

License: MIT license

Softwareheritage PID:

swh:1:snp:9702a4beb9ada324e96485cc8e7b985b40319d72;origin=https://github.com/YMSen/Paper_scripts_muskrat_muskdeer [122].

Data Availability

The sequencing data for this project have been deposited in the NCBI (National Center for Biotechnology Information) and can be accessed with BioProject No. PRJNA996334, PRJNA992196, PRJNA992194, PRJNA985369, PRJNA985371, PRJNA992197, PRJNA1132163 and PRJNA1197571. Musk deer transcriptomic sequencing data were re-used from BioProject PRJNA289641, PRJNA928235, PRJNA790817, PRJNA291827, PRJNA289642. All supporting data and materials are available in the *GigaScience* GigaDB database [123-125].

Ethics Statement

All animal research was conducted according to the Regulations for the Administration of Affairs Concerning Experimental Animals (Ministry of Science and Technology, China, revised in March 2017) and approved by the Animal Ethical and Welfare Committee (AEWC) of Chengdu University under permit No. YXY-2022630272.

Competing Interests

The authors declare that they have no conflict of interest.

Funding

This research was funded by the National Natural Science Foundation of China (81973428, 82274046 to H.J., and 32272859 to Z.Y.), the Fundamental Research Funds of Chongqing (2022JK017) to H.J., and the Beijing Nova Program (Z211100002121022 and 20230484446) to S.T.

Additional Files

Supplementary Fig. S1

Karyotype and genome assembly of muskrats.

Supplementary Fig. S2

Terms for expanded and contracted genes in muskrat and musk deer

Supplementary Fig. S3

Sequence alignment highlighting sites that evolved in *DST*, *CKAP5*, and *NoP2*.

Supplementary Fig. S4

Gene expression from 13 tissues in muskrat.

Supplementary Fig. S5

Significantly enriched GO terms in tissue-specific expressed genes for each tissue. Tissue specificity of gene abundance was reflected by the tau score (τ).

Supplementary Fig. S6

Transcriptome map of muskrat analyzed.

Supplementary Fig. S7

Hi-C data quality and global chromatin interaction patterns during two representative musk gland stages.

Supplementary Fig. S8

Basic features of PEIs.

Supplementary Fig. S9

PSGs and REGs involved in cell cycle and sequence alignment highlighting sites that evolved in parallel in *Casp8ap2*, *NAGS*, and *Cep250*.

Supplementary Fig. S10

Promoter-enhancer interactions (PEIs) rewired in the musk gland of musk secretion and non-secretion stages.

Supplementary Table S1

Sequencing data summary.

Supplementary Table S2

Summary for the chromosome-scale genomes of muskrat and musk.

Supplementary Table S3

Overview of chromosome-scale genomes of muskrat and musk deer.

Supplementary Table S4

Summary of assembly quality evaluation.

Supplementary Table S5

Summary of the TEs and statistics of gene structure in muskrat and musk deer genomes.

Supplementary Table S6

PSGs, REGs and convergent evolution gene in muskrat and musk deer genomes.

Supplementary Table S7

The significantly enriched terms for expanded genes in muskrat and musk deer.

Supplementary Table S8

The significantly enriched terms for contracted genes in muskrat and musk deer.

Supplementary Table S9

The significantly enriched terms for rapid evolution genes in muskrat.

Supplementary Table S10

The significantly enriched terms for positive selected and rapid evolution genes in musk deer.

Supplementary Table S11

Tissue-specific genes in each tissue.

Supplementary Table S12

Marker genes of each cell type.

Supplementary Table S13

Number of cells of each cell type in two musk secretion stages.

Supplementary Table S14

DEGs in comparison between musk secretion and non-secretion stages detected in cluster 14.

Supplementary Table S15

Hi-C data summary.

Supplementary Table S16

Hi-C matrix resolution evaluation by Cis contacts.

Authors' Contributions

Tao Wang: Writing - Original Draft, Writing - Review & Editing. **Maosen Yang:** Formal analysis, Software, Visualization. **Xin Shi:** Formal analysis, Data Curation, Visualization. **Shilin Tian:** Formal analysis, Funding acquisition, Software. **Yan Li:** Formal analysis, Methodology. **Wenqian Xie:** Formal analysis. **Zhengting Zou:** Review & Editing. **Dong Leng:** Formal analysis, Visualization. **Ming Zhang:** Data Curation, Resources. **Chengli Zheng:** Data Curation, Resources. **Chungang Feng:** Writing - Review & Editing. **Bo Zeng:** Investigation. **Xiaolan Fan:** Data Curation, Resources. **Huimin Qiu:** Formal analysis. **Jing Li:** Software. **Guijun Zhao:** Supervision. **Zhengrong Yuan:** Funding acquisition, Writing - Review & Editing. **Diyan Li:** Conceptualization, Methodology, Validation, Writing - Original Draft. **Hang Jie:** Funding acquisition, Writing - Review & Editing, Resources.

References

1. He L, Wang WX, Li LH, Liu BQ, Liu G, Liu SQ, et al. Effects of crowding and sex on fecal cortisol levels of captive forest musk deer. Biol Res. 2014;47 1:48. doi:10.1186/0717-6287-47-48.
2. Lv S, Lei Z, Yan G, Shah SA, Ahmed S and Sun T. Chemical compositions and pharmacological activities of natural musk (Moschus) and artificial musk: A review. Journal of Ethnopharmacology. 2022;284:114799. doi:https://doi.org/10.1016/j.jep.2021.114799.
3. Mychajliw AM and Harrison RG. Genetics reveal the origin and timing of a cryptic insular introduction of muskrats in North America. PLoS One. 2014;9 10:e111856. doi:10.1371/journal.pone.0111856.
4. Sokolov VE, Kagan MZ, Vasilieva VS, Prihodko VI and Zinkevich EP. Musk deer (Moschus moschiferus): Reinvestigation of main lipid components from preputial gland secretion. J Chem Ecol. 1987;13 1:71-83. doi:10.1007/BF01020352.

5. Li D, Chen B, Zhang L, Gaur U, Ma T, Jie H, et al. The musk chemical composition and microbiota of Chinese forest musk deer males. Sci Rep. 2016;6:18975. doi:10.1038/srep18975.
6. Li Y, Zhang T, Fan M, Zhou J, Yang S, Zhang M, et al. Comparison of amino acid profiles and metabolic gene expression in muskrat scented glands in secretion and non-secretion season. Sci Rep. 2017;7:41158. doi:10.1038/srep41158.
7. Shi X, Zeng D, Zhao G, Zhang C, Feng X, Zheng C, et al. Correlation Analysis between Muskrat (Ondatra zibethicus) Musk and Traditional Musk. Animals : an open access journal from MDPI. 2023;13 10 doi:10.3390/ani13101678.
8. Tian S, Zeng J, Jiao H, Zhang D, Zhang L, Lei CQ, et al. Comparative analyses of bat genomes identify distinct evolution of immunity in Old World fruit bats. Sci Adv. 2023;9 18:eadd0141. doi:10.1126/sciadv.add0141.
9. Simao FA, Waterhouse RM, Ioannidis P, Kriventseva EV and Zdobnov EM. BUSCO: assessing genome assembly and annotation completeness with single-copy orthologs. Bioinformatics. 2015;31 19:3210-2. doi:10.1093/bioinformatics/btv351.
10. Rhie A, Walenz BP, Koren S and Phillippy AM. Merqury: reference-free quality, completeness, and phasing assessment for genome assemblies. Genome Biol. 2020;21 1:245. doi:10.1186/s13059-020-02134-9.
11. Editorial NB. A reference standard for genome biology. Nat Biotechnol. 2018;36 12:1121. doi:10.1038/nbt.4318.
12. Yang Z. PAML 4: phylogenetic analysis by maximum likelihood. Mol Biol Evol. 2007;24 8:1586-91. doi:10.1093/molbev/msm088.
13. Zhang J and Kumar S. Detection of convergent and parallel evolution at the amino acid sequence level. Mol Biol Evol. 1997;14 5:527-36. doi:10.1093/oxfordjournals.molbev.a025789.
14. Zou Z and Zhang J. Are Convergent and Parallel Amino Acid Substitutions in Protein Evolution More Prevalent Than Neutral Expectations? Mol Biol Evol. 2015;32 8:2085-96. doi:10.1093/molbev/msv091.
15. Xu S, He Z, Guo Z, Zhang Z, Wyckoff GJ, Greenberg A, et al. Genome-Wide Convergence during Evolution of Mangroves from Woody Plants. Mol Biol Evol. 2017;34 4:1008-15. doi:10.1093/molbev/msw277.
16. Nummela S, Pihlström H, Puolamäki K, Fortelius M, Hemilä S and Reuter T. Exploring the mammalian sensory space: co-operations and trade-offs among senses. J Comp Physiol A Neuroethol Sens Neural Behav Physiol. 2013;199 12:1077-92. doi:10.1007/s00359-013-0846-2.
17. Liu C, Gao J, Cui X, Li Z, Chen L, Yuan Y, et al. A towering genome: Experimentally validated adaptations to high blood pressure and extreme stature in the giraffe. Sci Adv. 2021;7 12 doi:10.1126/sciadv.abe9459.
18. Yang F, Lan Y, Pandey RR, Homolka D, Berger SL, Pillai RS, et al. TEX15 associates with MILI and silences transposable elements in male germ cells. Genes & development. 2020;34 11-12:745-50. doi:10.1101/gad.335489.119.
19. Chan JY, Ng AYJ, Cheng CL, Nairismägi M-L, Venkatesh B, Cheah DMZ, et al. Whole exome sequencing identifies recessive germline mutations in FAM160A1 in familial NK/T cell lymphoma. Blood Cancer Journal. 2018;8 11:111. doi:10.1038/s41408-018-0149-5.

20. Sabo J, Dujava Zdimalova M, Slater PG, Dostal V, Herynek S, Libusova L, et al. CKAP5 enables formation of persistent actin bundles templated by dynamically instable microtubules. Current Biology. 2024;34 2:260-72.e7. doi:https://doi.org/10.1016/j.cub.2023.11.031.
21. Luo Q, Das A, Oldoni F, Wu P, Wang J, Luo F, et al. Role of ACSL5 in fatty acid metabolism. Heliyon. 2023;9 2:e13316. doi:10.1016/j.heliyon.2023.e13316.
22. Polinski NK, Martinez TN, Gorodinsky A, Gareus R, Sasner M, Herberth M, et al. Decreased glucocerebrosidase activity and substrate accumulation of glycosphingolipids in a novel GBA1 D409V knock-in mouse model. PloS one. 2021;16 6:e0252325. doi:10.1371/journal.pone.0252325.
23. Hirai K, Watanabe S, Nishijima N, Shibata K, Hase A, Yamanaka T, et al. Molecular and Functional Analysis of Choline Transporters and Antitumor Effects of Choline Transporter-Like Protein 1 Inhibitors in Human Pancreatic Cancer Cells. International journal of molecular sciences. 2020;21 15 doi:10.3390/ijms21155190.
24. Li C, Liu X, Liu Y, Liu X, Wang R, Liao J, et al. Keratin 80 promotes migration and invasion of colorectal carcinoma by interacting with PRKDC via activating the AKT pathway. Cell Death Dis. 2018;9 10:1009. doi:10.1038/s41419-018-1030-y.
25. Attar LJ, Alelaimat A, Alshorman A and Aladily TN. MPIG6B Gene-Related Myelofibrosis: A Rare Inherited Disease That Is Frequently Described in Arab Population. Avicenna J Med. 2024;14 1:69-72. doi:10.1055/s-0044-1779697.
26. Clapham DE. TRP channels as cellular sensors. Nature. 2003;426 6966:517-24. doi:10.1038/nature02196.
27. Deng Z, Maksaev G, Rau M, Xie Z, Hu H, Fitzpatrick JAJ, et al. Gating of human TRPV3 in a lipid bilayer. Nature structural & molecular biology. 2020;27 7:635-44. doi:10.1038/s41594-020-0428-2.
28. Xu H, Delling M, Jun JC and Clapham DE. Oregano, thyme and clove-derived flavors and skin sensitizers activate specific TRP channels. Nat Neurosci. 2006;9 5:628-35. doi:10.1038/nn1692.
29. Holmes RS, Cox LA and VandeBerg JL. Comparative studies of mammalian acid lipases: Evidence for a new gene family in mouse and rat (Lipo). Comp Biochem Physiol Part D Genomics Proteomics. 2010;5 3:217-26. doi:10.1016/j.cbd.2010.05.004.
30. Ding JF, Sun H, Song K, Zhou Y, Tu B, Shi KH, et al. IGFBP3 epigenetic promotion induced by METTL3 boosts cardiac fibroblast activation and fibrosis. European journal of pharmacology. 2023;942:175494. doi:10.1016/j.ejphar.2023.175494.
31. Wu JJ, Zhu S, Tang YF, Gu F, Liu JX and Sun HZ. Microbiota-host crosstalk in the newborn and adult rumen at single-cell resolution. BMC biology. 2022;20 1:280. doi:10.1186/s12915-022-01490-1.
32. Fan C, Liao M, Xie L, Huang L, Lv S, Cai S, et al. Single-Cell Transcriptome Integration Analysis Reveals the Correlation Between Mesenchymal Stromal Cells and Fibroblasts. Frontiers in genetics. 2022;13:798331. doi:10.3389/fgene.2022.798331.
33. Afzali B and Kemper C. Fibroblast tissue priming-not so nice to C you! Immunity. 2021;54 5:847-50. doi:10.1016/j.immuni.2021.04.010.
34. Farmer DT, Nathan S, Finley JK, Shengyang Yu K, Emmerson E, Byrnes LE, et al. Defining epithelial cell dynamics and lineage relationships in the developing lacrimal gland.

Development (Cambridge, England). 2017;144 13:2517-28. doi:10.1242/dev.150789.

35. Park J, Shrestha R, Qiu C, Kondo A, Huang S, Werth M, et al. Single-cell transcriptomics of the mouse kidney reveals potential cellular targets of kidney disease. Science (New York, NY). 2018;360 6390:758-63. doi:10.1126/science.aar2131.

36. Gladka MM, Molenaar B, de Ruiter H, van der Elst S, Tsui H, Versteeg D, et al. Single-Cell Sequencing of the Healthy and Diseased Heart Reveals Cytoskeleton-Associated Protein 4 as a New Modulator of Fibroblasts Activation. Circulation. 2018;138 2:166-80. doi:10.1161/circulationaha.117.030742.

37. Tusi BK, Wolock SL, Weinreb C, Hwang Y, Hidalgo D, Zilionis R, et al. Population snapshots predict early haematopoietic and erythroid hierarchies. Nature. 2018;555 7694:54-60. doi:10.1038/nature25741.

38. Xie T, Wang Y, Deng N, Huang G, Taghavifar F, Geng Y, et al. Single-Cell Deconvolution of Fibroblast Heterogeneity in Mouse Pulmonary Fibrosis. Cell reports. 2018;22 13:3625-40. doi:10.1016/j.celrep.2018.03.010.

39. Castle JC, Loewer M, Boegel S, de Graaf J, Bender C, Tadmor AD, et al. Immunomic, genomic and transcriptomic characterization of CT26 colorectal carcinoma. BMC genomics. 2014;15 1:190. doi:10.1186/1471-2164-15-190.

40. Guo M, Wang H, Potter SS, Whitsett JA and Xu Y. SINCERA: A Pipeline for Single-Cell RNA-Seq Profiling Analysis. PLoS computational biology. 2015;11 11:e1004575. doi:10.1371/journal.pcbi.1004575.

41. Ichikawa T, Sugiura H, Koarai A, Yanagisawa S, Kanda M, Hayata A, et al. Peroxynitrite augments fibroblast-mediated tissue remodeling via myofibroblast differentiation. American journal of physiology Lung cellular and molecular physiology. 2008;295 5:L800-8. doi:10.1152/ajplung.90264.2008.

42. Yeh MY, Shih YL, Chung HY, Chou J, Lu HF, Liu CH, et al. Chitosan promotes immune responses, ameliorates glutamic oxaloacetic transaminase and glutamic pyruvic transaminase, but enhances lactate dehydrogenase levels in normal mice in vivo. Experimental and therapeutic medicine. 2016;11 4:1300-6. doi:10.3892/etm.2016.3057.

43. Chueh FS, Lin JJ, Lin JP, Yu FS, Lin JH, Ma YS, et al. Crude extract of Polygonum cuspidatum promotes immune responses in leukemic mice through enhancing phagocytosis of macrophage and natural killer cell activities in vivo. In vivo (Athens, Greece). 2015;29 2:255-61. https://iv.iiarjournals.org/content/29/2/255.short

44. Chueh FS, Lin JJ, Lin JH, Weng SW, Huang YP and Chung JG. Crude extract of Polygonum cuspidatum stimulates immune responses in normal mice by increasing the percentage of Mac-3-positive cells and enhancing macrophage phagocytic activity and natural killer cell cytotoxicity. Molecular medicine reports. 2015;11 1:127-32. doi:10.3892/mmr.2014.2739.

45. Lin CC, Kuo CL, Lee MH, Hsu SC, Huang AC, Tang NY, et al. Extract of Hedyotis diffusa Willd influences murine leukemia WEHI-3 cells in vivo as well as promoting T- and B-cell proliferation in leukemic mice. In vivo (Athens, Greece). 2011;25 4:633-40. https://iv.iiarjournals.org/content/25/4/633.short.

46. Plasschaert LW, Žilionis R, Choo-Wing R, Savova V, Knehr J, Roma G, et al. A single-cell atlas of the airway epithelium reveals the CFTR-rich pulmonary ionocyte. Nature. 2018;560 7718:377-81. doi:10.1038/s41586-018-0394-6.

47. Sharifiaghdas F, Naji M, Sarhangnejad R, Rajabi-Zeleti S, Mirzadeh H, Zandi M, et al.

Comparing supportive properties of poly lactic-co-glycolic acid (PLGA), PLGA/collagen and human amniotic membrane for human urothelial and smooth muscle cells engineering. Urology journal. 2014;11 3:1620-8. https://doi.org/10.22037/uj.v11i3.2120

48. Demir IE, Boldis A, Pfitzinger PL, Teller S, Brunner E, Klose N, et al. Investigation of Schwann cells at neoplastic cell sites before the onset of cancer invasion. Journal of the National Cancer Institute. 2014;106 8 doi:10.1093/jnci/dju184.

49. Saiki T, Nakamura N, Miyabe M, Ito M, Minato T, Sango K, et al. The Effects of Insulin on Immortalized Rat Schwann Cells, IFRS1. International journal of molecular sciences. 2021;22 11 doi:10.3390/ijms22115505.

50. Deborde S, Omelchenko T, Lyubchik A, Zhou Y, He S, McNamara WF, et al. Schwann cells induce cancer cell dispersion and invasion. The Journal of clinical investigation. 2016;126 4:1538-54. doi:10.1172/jci82658.

51. Dezawa M and Adachi-Usami E. Role of Schwann cells in retinal ganglion cell axon regeneration. Progress in retinal and eye research. 2000;19 2:171-204. doi:10.1016/s1350-9462(99)00010-5.

52. Azam SH and Pecot CV. Cancer's got nerve: Schwann cells drive perineural invasion. The Journal of clinical investigation. 2016;126 4:1242-4. doi:10.1172/jci86801.

53. Tong HL, Jiang RY, Zhang WW and Yan YQ. MiR-2425-5p targets RAD9A and MYOG to regulate the proliferation and differentiation of bovine skeletal muscle-derived satellite cells. Scientific reports. 2017;7 1:418. doi:10.1038/s41598-017-00470-8.

54. Chen J, Sun T, You Y, Lin B, Wu B and Wu J. Genome-wide identification of potential odontogenic genes involved in the dental epithelium-mesenchymal interaction during early odontogenesis. BMC genomics. 2023;24 1:163. doi:10.1186/s12864-023-09140-8.

55. Karihaloo A, Kale S, Rosenblum ND and Cantley LG. Hepatocyte growth factor-mediated renal epithelial branching morphogenesis is regulated by glypican-4 expression. Molecular and cellular biology. 2004;24 19:8745-52. doi:10.1128/mcb.24.19.8745-8752.2004.

56. Ho CM, Chang TH, Yen TL, Hong KJ and Huang SH. Collagen type VI regulates the CDK4/6-p-Rb signaling pathway and promotes ovarian cancer invasiveness, stemness, and metastasis. American journal of cancer research. 2021;11 3:668-90. https://pmc.ncbi.nlm.nih.gov/articles/PMC7994167/.

57. Donker L, Houtekamer R, Vliem M, Sipieter F, Canever H, Gómez-González M, et al. A mechanical G2 checkpoint controls epithelial cell division through E-cadherin-mediated regulation of Wee1-Cdk1. Cell reports. 2022;41 2:111475. doi:10.1016/j.celrep.2022.111475.

58. Arai C, Yoshizaki K, Miyazaki K, Saito K, Yamada A, Han X, et al. Nephronectin plays critical roles in Sox2 expression and proliferation in dental epithelial stem cells via EGF-like repeat domains. Scientific reports. 2017;7:45181. doi:10.1038/srep45181.

59. Han Y, Villarreal-Ponce A, Gutierrez G, Jr., Nguyen Q, Sun P, Wu T, et al. Coordinate control of basal epithelial cell fate and stem cell maintenance by core EMT transcription factor Zeb1. Cell reports. 2022;38 2:110240. doi:10.1016/j.celrep.2021.110240.

60. Lixa C, Clarkson MW, Iqbal A, Moon TM, Almeida FCL, Peti W, et al. Retinoic Acid Binding Leads to CRABP2 Rigidification and Dimerization. Biochemistry. 2019;58 41:4183-94. doi:10.1021/acs.biochem.9b00672.

61. Casteels M, Sniekers M, Fraccascia P, Mannaerts GP and Van Veldhoven PP. The role of 2-

hydroxyacyl-CoA lyase, a thiamin pyrophosphate-dependent enzyme, in the peroxisomal metabolism of 3-methyl-branched fatty acids and 2-hydroxy straight-chain fatty acids. Biochemical Society transactions. 2007;35 Pt 5:876-80. doi:10.1042/bst0350876.

62. Itkonen HM, Brown M, Urbanucci A, Tredwell G, Ho Lau C, Barfeld S, et al. Lipid degradation promotes prostate cancer cell survival. Oncotarget. 2017;8 24:38264-75. doi:10.18632/oncotarget.16123.

63. Piórkowska K, Tyra M, Ropka-Molik K and Podbielska A. Evolution of peroxisomal trans-2-enoyl-CoA reductase (PECR) as candidate gene for meat quality. Livestock Science. 2017;201:85-91. doi:https://doi.org/10.1016/j.livsci.2017.05.004.

64. Zhang M, Yang S, Shi M, Zhang S, Zhang T, Li Y, et al. Regulatory Roles of Peroxisomal Metabolic Pathways Involved in Musk Secretion in Muskrats. J Membr Biol. 2019;252 1:61-75. doi:10.1007/s00232-018-0057-4.

65. Lieberman-Aiden E, van Berkum NL, Williams L, Imakaev M, Ragoczy T, Telling A, et al. Comprehensive mapping of long-range interactions reveals folding principles of the human genome. Science. 2009;326 5950:289-93. doi:10.1126/science.1181369.

66. Schoenfelder S and Fraser P. Long-range enhancer-promoter contacts in gene expression control. Nature reviews Genetics. 2019;20 8:437-55. doi:10.1038/s41576-019-0128-0.

67. Li D, Ning C, Zhang J, Wang Y, Tang Q, Kui H, et al. Dynamic transcriptome and chromatin architecture in granulosa cells during chicken folliculogenesis. Nature communications. 2022;13 1:131. doi:10.1038/s41467-021-27800-9.

68. Al Kaabi EH and El-Hattab AW. N-acetylglutamate synthase deficiency: Novel mutation associated with neonatal presentation and literature review of molecular and phenotypic spectra. Mol Genet Metab Rep. 2016;8:94-8. doi:10.1016/j.ymgmr.2016.08.004.

69. Floriot S, Bellutti L, Castille J, Moison P, Messiaen S, Passet B, et al. CEP250 is Required for Maintaining Centrosome Cohesion in the Germline and Fertility in Male Mice. Front Cell Dev Biol. 2021;9:754054. doi:10.3389/fcell.2021.754054.

70. Li ZG, Wu MY and Jia HT. [Research Progress on Expression Regulation, Function and Clinical Significance of CASP8AP2 Gene]. Zhongguo Shi Yan Xue Ye Xue Za Zhi. 2015;23 2:557-61. doi:10.7534/j.issn.1009-2137.2015.02.051.

71. Shin H and Chung H. SMPDL3A links cholesterol metabolism to the cGAS-STING pathway. Immunity. 2023;56 11:2459-61. doi:https://doi.org/10.1016/j.immuni.2023.10.015.

72. Zhou L, He L, Liu CH, Qiu H, Zheng L, Sample KM, et al. Liver cancer stem cell dissemination and metastasis: uncovering the role of NRCAM in hepatocellular carcinoma. J Exp Clin Cancer Res. 2023;42 1:311. doi:10.1186/s13046-023-02893-w.

73. Suddason T and Gallagher E. A RING to rule them all? Insights into the Map3k1 PHD motif provide a new mechanistic understanding into the diverse roles of Map3k1. Cell Death Differ. 2015;22 4:540-8. doi:10.1038/cdd.2014.239.

74. Mulas C, Kalkan T and Smith A. NODAL Secures Pluripotency upon Embryonic Stem Cell Progression from the Ground State. Stem Cell Reports. 2017;9 1:77-91. doi:https://doi.org/10.1016/j.stemcr.2017.05.033.

75. Gauthier-Coles G, Bröer A, McLeod MD, George AJ, Hannan RD and Bröer S. Identification and characterization of a novel SNAT2 (SLC38A2) inhibitor reveals synergy with glucose transport inhibition in cancer cells. Front Pharmacol. 2022;13:963066.

doi:10.3389/fphar.2022.963066.
76. Hu J, Wang Z, Sun Z, Hu B, Ayoola AO, Liang F, et al. NextDenovo: an efficient error correction and accurate assembly tool for noisy long reads. Genome Biol. 2024;25 1:107. doi:10.1186/s13059-024-03252-4.
77. Hu J, Fan J, Sun Z and Liu S. NextPolish: a fast and efficient genome polishing tool for long-read assembly. Bioinformatics. 2020;36 7:2253-5. doi:10.1093/bioinformatics/btz891.
78. Tian S, Comparative genomics of horseshoe bats. https://doi.org/10.5281/zenodo.13690583
79. Langmead B and Salzberg SL. Fast gapped-read alignment with Bowtie 2. Nat Methods. 2012;9 4:357-9. doi:10.1038/nmeth.1923.
80. Wingett S, Ewels P, Furlan-Magaril M, Nagano T, Schoenfelder S, Fraser P, et al. HiCUP: pipeline for mapping and processing Hi-C data. F1000Res. 2015;4:1310. doi:10.12688/f1000research.7334.1.
81. Li H. Minimap2: pairwise alignment for nucleotide sequences. Bioinformatics. 2018;34 18:3094-100. doi:10.1093/bioinformatics/bty191.
82. Myers EW. The fragment assembly string graph. Bioinformatics. 2005;21 Suppl 2:ii79-85. doi:10.1093/bioinformatics/bti1114.
83. Zhang X, Zhang S, Zhao Q, Ming R and Tang H. Assembly of allele-aware, chromosomal-scale autopolyploid genomes based on Hi-C data. Nat Plants. 2019;5 8:833-45. doi:10.1038/s41477-019-0487-8.
84. Li H and Durbin R. Fast and accurate long-read alignment with Burrows-Wheeler transform. Bioinformatics. 2010;26 5:589-95. doi:10.1093/bioinformatics/btp698.
85. Bergman CM and Quesneville H. Discovering and detecting transposable elements in genome sequences. Brief Bioinform. 2007;8 6:382-92. doi:10.1093/bib/bbm048.
86. Edgar RC and Myers EW. PILER: identification and classification of genomic repeats. Bioinformatics. 2005;21 Suppl 1:i152-8. doi:10.1093/bioinformatics/bti1003.
87. Xu Z and Wang H. LTR_FINDER: an efficient tool for the prediction of full-length LTR retrotransposons. Nucleic Acids Res. 2007;35 Web Server issue:W265-8. doi:10.1093/nar/gkm286.
88. Price AL, Jones NC and Pevzner PA. De novo identification of repeat families in large genomes. Bioinformatics. 2005;21 Suppl 1:i351-8. doi:10.1093/bioinformatics/bti1018.
89. Benson G. Tandem repeats finder: a program to analyze DNA sequences. Nucleic Acids Res. 1999;27 2:573-80. doi:10.1093/nar/27.2.573.
90. Mount DW. Using the Basic Local Alignment Search Tool (BLAST). CSH Protoc. 2007;2007:pdb top17. doi:10.1101/pdb.top17.
91. Birney E, Clamp M and Durbin R. GeneWise and Genomewise. Genome Res. 2004;14 5:988-95. doi:10.1101/gr.1865504.
92. Kim D, Pertea G, Trapnell C, Pimentel H, Kelley R and Salzberg SL. TopHat2: accurate alignment of transcriptomes in the presence of insertions, deletions and gene fusions. Genome Biol. 2013;14 4:R36. doi:10.1186/gb-2013-14-4-r36.
93. Trapnell C, Roberts A, Goff L, Pertea G, Kim D, Kelley DR, et al. Differential gene and transcript expression analysis of RNA-seq experiments with TopHat and Cufflinks. Nat Protoc. 2012;7 3:562-78. doi:10.1038/nprot.2012.016.
94. Haas BJ, Delcher AL, Mount SM, Wortman JR, Smith Jr RK, Hannick LI, et al. Improving

the Arabidopsis genome annotation using maximal transcript alignment assemblies. Nucleic acids research. 2003;31 19:5654-66. doi: 10.1093/nar/gkg770.

95. Stanke M and Waack S. Gene prediction with a hidden Markov model and a new intron submodel. Bioinformatics. 2003;19 Suppl 2:ii215-25. doi:10.1093/bioinformatics/btg1080.

96. Korf I. Gene finding in novel genomes. BMC Bioinformatics. 2004;5:59. doi:10.1186/1471-2105-5-59.

97. Majoros WH, Pertea M and Salzberg SL. TigrScan and GlimmerHMM: two open source ab initio eukaryotic gene-finders. Bioinformatics. 2004;20 16:2878-9. doi:10.1093/bioinformatics/bth315.

98. Guigo R. Assembling genes from predicted exons in linear time with dynamic programming. J Comput Biol. 1998;5 4:681-702. doi:10.1089/cmb.1998.5.681.

99. Burge C and Karlin S. Prediction of complete gene structures in human genomic DNA. J Mol Biol. 1997;268 1:78-94. doi:10.1006/jmbi.1997.0951.

100. UniProt Consortium T. UniProt: the universal protein knowledgebase. Nucleic Acids Res. 2018;46 5:2699. doi:10.1093/nar/gky092.

101. Kanehisa M, Goto S, Sato Y, Kawashima M, Furumichi M and Tanabe M. Data, information, knowledge and principle: back to metabolism in KEGG. Nucleic Acids Res. 2014;42 Database issue:D199-205. doi:10.1093/nar/gkt1076.

102. Wang Y, Tang H, Debarry JD, Tan X, Li J, Wang X, et al. MCScanX: a toolkit for detection and evolutionary analysis of gene synteny and collinearity. Nucleic Acids Res. 2012;40 7:e49. doi:10.1093/nar/gkr1293.

103. Veidenberg A, Medlar A and Loytynoja A. Wasabi: An Integrated Platform for Evolutionary Sequence Analysis and Data Visualization. Mol Biol Evol. 2016;33 4:1126-30. doi:10.1093/molbev/msv333.

104. Talavera G and Castresana J. Improvement of phylogenies after removing divergent and ambiguously aligned blocks from protein sequence alignments. Syst Biol. 2007;56 4:564-77. doi:10.1080/10635150701472164.

105. Posada D and Crandall KA. MODELTEST: testing the model of DNA substitution. Bioinformatics. 1998;14 9:817-8. doi:10.1093/bioinformatics/14.9.817.

106. Stamatakis A. RAxML version 8: a tool for phylogenetic analysis and post-analysis of large phylogenies. Bioinformatics. 2014;30 9:1312-3. doi:10.1093/bioinformatics/btu033.

107. Li L, Stoeckert CJ, Jr. and Roos DS. OrthoMCL: identification of ortholog groups for eukaryotic genomes. Genome Res. 2003;13 9:2178-89. doi:10.1101/gr.1224503.

108. De Bie T, Cristianini N, Demuth JP and Hahn MW. CAFE: a computational tool for the study of gene family evolution. Bioinformatics. 2006;22 10:1269-71. doi:10.1093/bioinformatics/btl097.

109. Xie C, Mao X, Huang J, Ding Y, Wu J, Dong S, et al. KOBAS 2.0: a web server for annotation and identification of enriched pathways and diseases. Nucleic Acids Res. 2011;39 Web Server issue:W316-22. doi:10.1093/nar/gkr483.

110. Mao X, Cai T, Olyarchuk JG and Wei L. Automated genome annotation and pathway identification using the KEGG Orthology (KO) as a controlled vocabulary. Bioinformatics. 2005;21 19:3787-93. doi: 10.1093/bioinformatics/bti430.

111. Yanai I, Benjamin H, Shmoish M, Chalifa-Caspi V, Shklar M, Ophir R, et al. Genome-wide midrange transcription profiles reveal expression level relationships in human tissue

specification. Bioinformatics. 2005;21 5:650-9. doi:10.1093/bioinformatics/bti042.

112. Robinson MD, McCarthy DJ and Smyth GK. edgeR: a Bioconductor package for differential expression analysis of digital gene expression data. Bioinformatics. 2010;26 1:139-40. doi:10.1093/bioinformatics/btp616.

113. Chen S, Zhou Y, Chen Y and Gu J. fastp: an ultra-fast all-in-one FASTQ preprocessor. Bioinformatics. 2018;34 17:i884-i90. doi:10.1093/bioinformatics/bty560.

114. Zheng GX, Terry JM, Belgrader P, Ryvkin P, Bent ZW, Wilson R, et al. Massively parallel digital transcriptional profiling of single cells. Nature communications. 2017;8:14049. doi:10.1038/ncomms14049.

115. Butler A, Hoffman P, Smibert P, Papalexi E and Satija R. Integrating single-cell transcriptomic data across different conditions, technologies, and species. Nat Biotechnol. 2018;36 5:411-20. doi:10.1038/nbt.4096.

116. Wang T, Leng D, Cai Z, Chen B, Li J, Kui H, et al. Insights into left-right asymmetric development of chicken ovary at the single-cell level. J Genet Genomics. 2024; doi:10.1016/j.jgg.2024.08.002.

117. Leng D, Zeng B, Wang T, Chen B-L, Li D-Y and Li Z-J. Single nucleus/cell RNA-seq of the chicken hypothalamic-pituitary-ovarian axis offers new insights into the molecular regulatory mechanisms of ovarian development. Zoological Research. 2024;45 5:1088-107. doi:10.24272/j.issn.2095-8137.2024.037.

118. Durand NC, Shamim MS, Machol I, Rao SS, Huntley MH, Lander ES, et al. Juicer Provides a One-Click System for Analyzing Loop-Resolution Hi-C Experiments. Cell systems. 2016;3 1:95-8. doi:10.1016/j.cels.2016.07.002.

119. Battulin N, Fishman VS, Mazur AM, Pomaznoy M, Khabarova AA, Afonnikov DA, et al. Comparison of the 3D organization of sperm and fibroblast genomes using the Hi-C approach. Genome biology. 2015;16 1:77. doi:10.1186/s13059-015-0642-0.

120. Kruse K, Hug CB, Hernandez-Rodriguez B and Vaquerizas JM. TADtool: visual parameter identification for TAD-calling algorithms. Bioinformatics. 2016;32 20:3190-2. doi:10.1093/bioinformatics/btw368.

121. Ron G, Globerson Y, Moran D and Kaplan T. Promoter-enhancer interactions identified from Hi-C data using probabilistic models and hierarchical topological domains. Nature communications. 2017;8 1:2237. doi:10.1038/s41467-017-02386-3.

122. Wang T, YangM, Shi X et al.. Scripts for paper "Multi-omics analysis provides insights into musk secretion in muskrat and musk deer". [Computer software]. Software Heritage, https://archive.softwareheritage.org/swh:1:snp:9702a4beb9ada324e96485cc8e7b985b40319d72;origin=https://github.com/YMSen/Paper_scripts_muskrat_muskdeer

123. Wang T; Yang M; Shi X; Tian S; Li Y; Xie W; Zou Z; Leng D; Zhang M; Zheng C; Feng C; Zeng B; Fan X; Qiu H; Li J; Zhao G; Yuan Z; Li D; Jie H. Supporting data for "Multi-omics analysis provides insights into musk secretion in muskrat and musk deer" GigaScience Database 2024. https://doi.org/10.5524/102628

124. Wang T; Yang M; Shi X; Tian S; Li Y; Xie W; Zou Z; Leng D; Zhang M; Zheng C; Feng C; Zeng B; Fan X; Qiu H; Li J; Zhao G; Yuan Z; Li D; Jie H. Genome assembly of the muskrat Ondatra zibethicus GigaScience Database 2024. https://doi.org/10.5524/102630

125. Wang T; Yang M; Shi X; Tian S; Li Y; Xie W; Zou Z; Leng D; Zhang M; Zheng C; Feng C; Zeng B; Fan X; Qiu H; Li J; Zhao G; Yuan Z; Li D; Jie H. Genome assembly of the musk deer

Moschus berezovskii Flerov GigaScience Database 2024. https://doi.org/10.5524/102631

Figure 1

Click here to access/download;Figure;Figure1.pdf

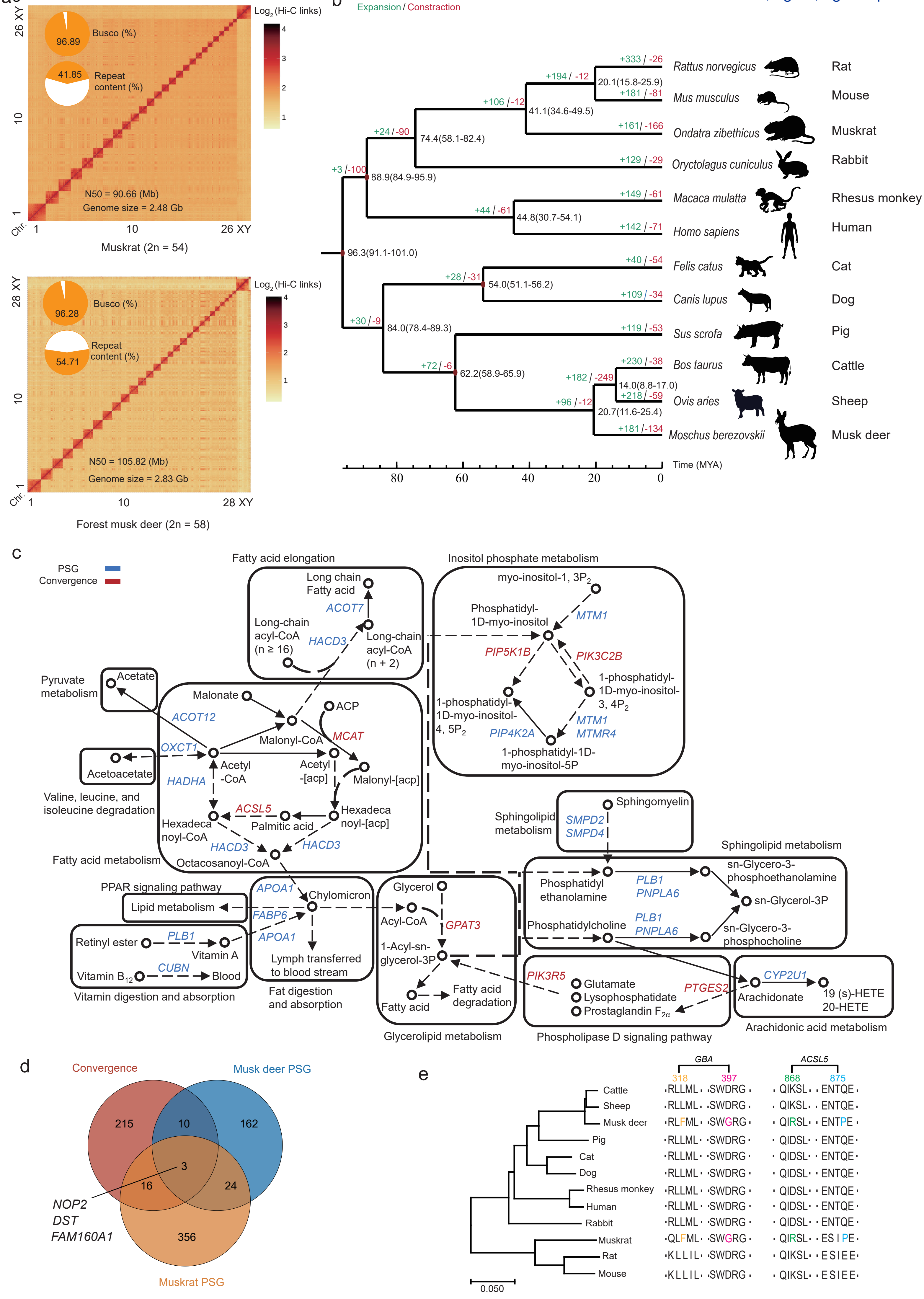

Figure 2

Click here to access/download;Figure;Figure2.pdf

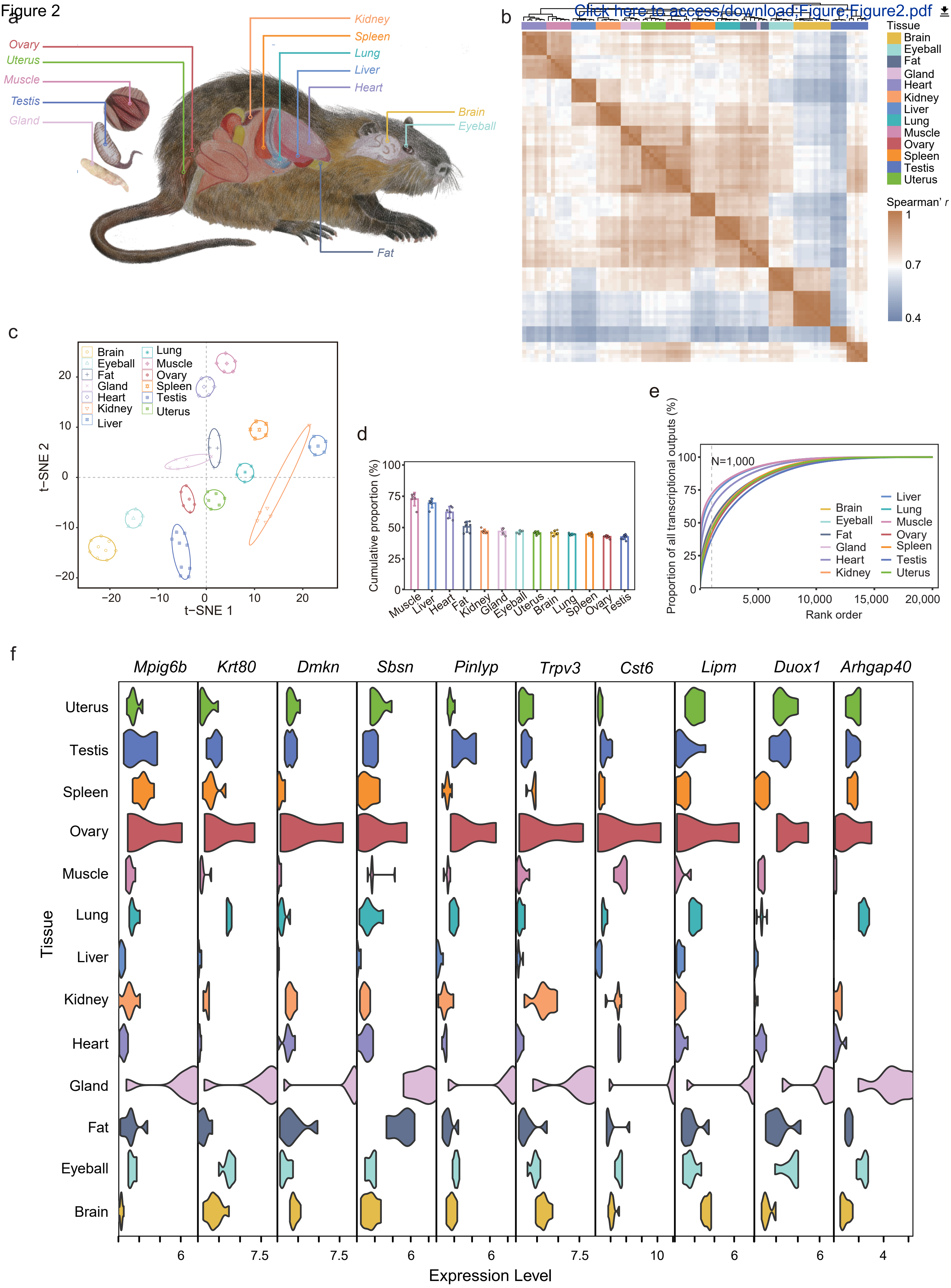

Figure 3

Click here to access/download;Figure;Figure3.pdf

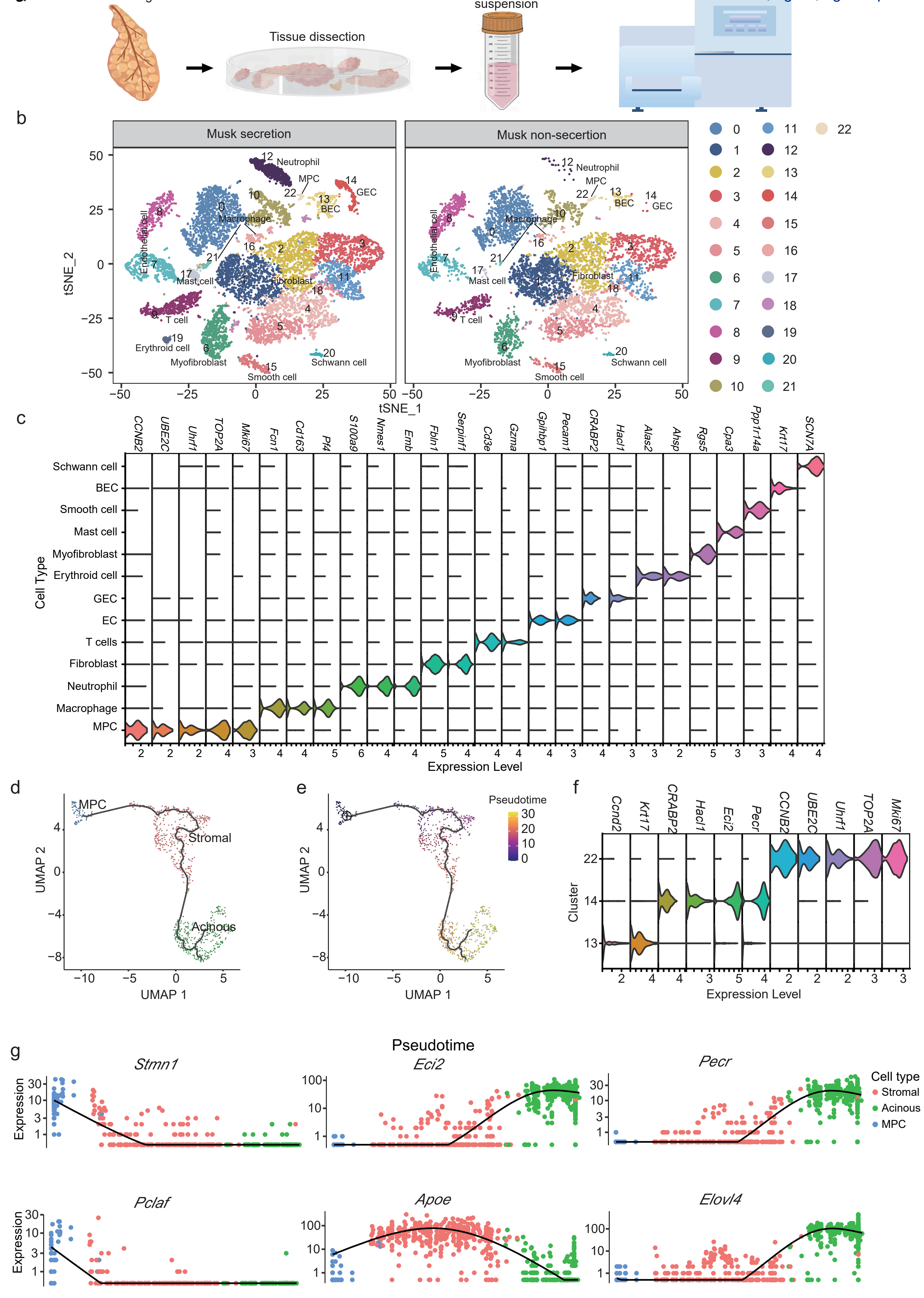

Click here to access/download;Figure;Figure4.pdf

Figure 4

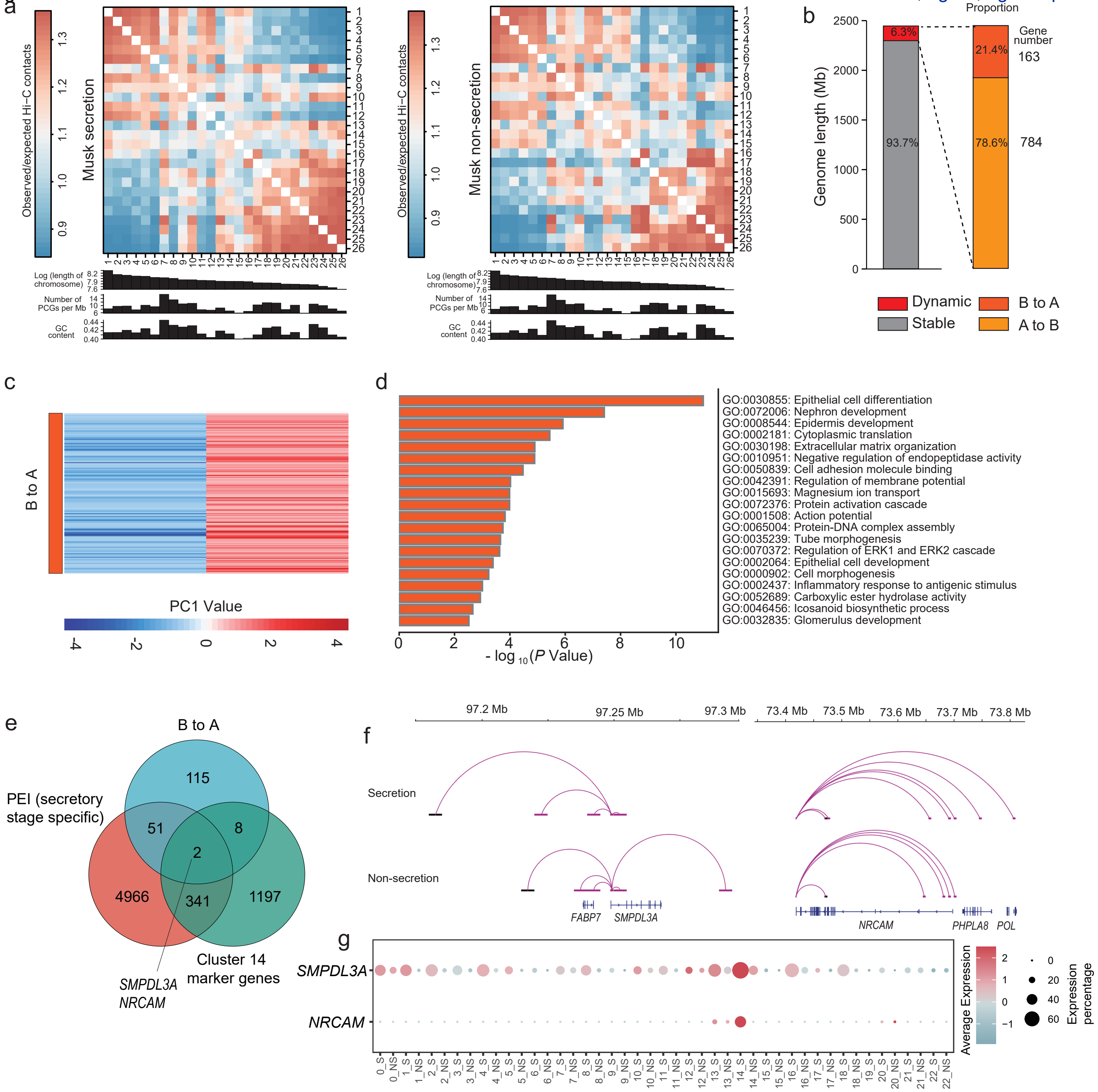

Figure 5

Click here to access/download;Figure;Figure5.pdf

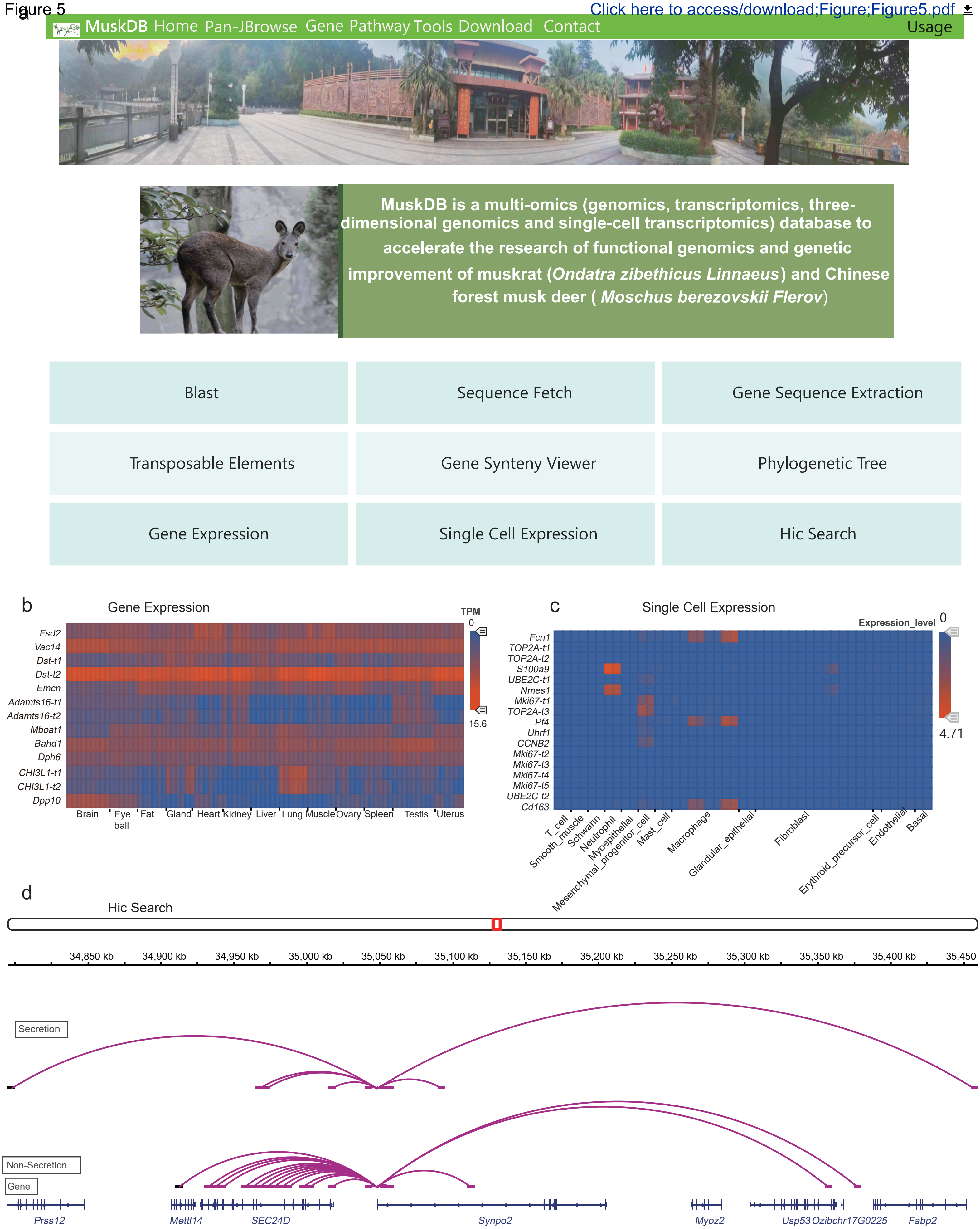

Click here to access/download
Supplementary Material
Additional file1.xlsx

Click here to access/download
Supplementary Material
Surpporting information.docx
