# Supplementary material for: Multiomics analysis provides insights into musk secretion in muskrat and musk deer
Source: Gigascience. 2025 Feb 26;14:giaf006. doi: 10.1093/gigascience/giaf006 (PMC11878540; doi:10.1093/gigascience/giaf006)
Supplement: giaf006_Response_to_Reviewer_Comments_Original_Submission [file giaf006_response_to_reviewer_comments_original_submission.pdf]

Author's Response To Reviewer Comments

Dear editor,
We sincerely thank you for allowing us to revise our manuscript entitled ‘Multi-omics analyses identify distinct patterns of selection in musk secretion animals’ (original manuscript No. GIGA-D-24-00205) with substantial improvements and resolve all the concerns raised by the reviewers. 1) We revised our manuscript carefully, reanalyzed the data, especially the convergent evolution genes; 2) ‘Better Papers Faster’ was asked for our originally submitted and the current revised manuscripts for editing service to check for grammar, and polish the writing; 3) all figures are recognized.
Below we provide our point-to-point responses, and hope that you and the reviewers are satisfied many thanks for your consideration of our manuscript for possible publication in GigaScience.
We look forward to hearing a positive response from you.

Best regards,
Zhengrong Yuan, Diyan Li and Hang Jie

Detailed responses to reviewers
All comments provided by reviewers are in gray italics, and our responses are in black. Important revisions in the manuscript are marked in red.

Reviewer 1
Comment 1-1:
Wang et al. utilized multi-omics data to investigate the mechanism underlying musk secreting, by sequencing the genomes of two musk-secreting mammals (muskrat and musk deer), 188 RNA-seq libraries of muskrat organs, scRNA-seq, and HiC data. The size of dataset generated here is large and such omics data would provide great resources for related studies. However, I have several concerns.
Major:
1.The data presented in this study is impressive, but no strong connections among different data analysis sections (genome assembly, bulk RNA-seq analysis, scRNA-seq, HiC and selection analysis). The authors should carefully re-organize and connect these sections by logic. Five figures are recommended. Suggestions on section organization: PSG and REG analysis should be moved after genome assembly part. The PEI (Line373) and compartment/TAD analysis (Line296) should be merged as one section.
Response 1-1:
Thanks for your positive and helpful comments.
(1) As suggested, only five figures were displayed in our revised manuscript.
(2) PSG and REG analysis was moved after genome assembly part.
(3) The PEI and compartment/TAD analysis were merged as one section.

Comment 1-2:
This study also sequenced 84 small RNA-seq libraries (line 78/line 145). But no analysis about small RNA was found in this manuscript. The small RNA-seq is miRNA-seq (Line 720)? I also noticed the sequencing of lncRNA-seq (Line 718). Too confusing.
Response 1-2:
Thanks for the comments, yes, we also sequenced 84 small RNA-seq libraries (it is miRNA). We only did basic analysis of miRNA (Figure S4b), including mapping, expression and t-SNE clustering analysis. But we did not do differential expression and function enrichment analysis among different tissues. For lncRNA-seq libraries, we mostly focused on analysis the expression profile of mRNAs. The lncRNA analysis was also similar to miRNA, only including mapping, expression and t-SNE clustering analysis (Figure S4a).

Figure S4. Gene expression from 13 tissues in muskrat. (a) t - distributed stochastic neighbor embedding (t-SNE) clustering of samples using lncRNA (left panel) and miRNA (right panel) expression. (b) The lncRNAs (left panel) and miRNAs (right panel) specifically expressed in musk gland. (c) The proportion of expressed genes of each sample. Each dot represents a sample in every tissue. (d) Abundance distribution of transcripts across tissues. The x-axis indicates the proportion of transcripts sorted from highest to lowest abundance, with the vertical dashed line indicating the top 1,000 of highest abundance transcripts.

The y-axis indicates the accumulated fraction of transcripts relative to the total transcripts. Colored lines represent mean values across tissues. (e) Heatmap of DEGs (differentially expressed genes) numbers in pairwise comparisons among the 13 tissues. DEGs were identified using the threshold of |log2fold change| > 1 and corrected (P < 0.01).

Comment 1-3:
The material&method part was poorly organized and written and I can not follow. For example, in the part named "Expression analysis of mRNA, lncRNA and miRNA"(Line863), the contents were the experimental details of RNA-seq with no expression analysis methods. Also this experimental details repeated the previous contents (Line704-724) but with inconsistent description.
Response 1-3:
Thanks for your constructive comments. Sorry for the repeated "Expression analysis" methods. We deleted it and carefully revised the expression analysis method to the subsection of "Muskrat transcriptome reconstruction".

Comment 1-4:
Many typos. The authors should check and revise carefully.
Response 1-4:
Thanks for the helpful comments. Sorry for our carelessness. In our revised manuscript, we have checked and revised carefully.

Comment 1-5:
Line94-96 The sequencing depth and quality should be mentioned here. Also only OZ sequencing data information was found in Table S1.
Response 1-5:
Thanks for your suggestions. We describe the sequencing depth of the two musk-secreting mammals (muskrat and musk deer): briefly, muskrat and forest musk deer had 235.25-fold and 246.02-fold sequencing depths, respectively. The quality sequencing is also shown in Table S1.

Comment 1-6:
Line 122-125 Simplify the statement, especially the use of software (CAFE, OrthoFinder..) which should be in Method.
Response 1-6:
We are sincerely grateful for your comments. We Simplify the statement, especially the use of software, as they are already described in the Methods. We also checked the results section for similar errors and made the same modifications, with the modification process marked in red.

Comment 1-7:
What function modules are the expanded or contracted gene families enriched in ? Or the numbers here mean nothing.
Response 1-7:
Thanks for the constructive comments. Enrichment analysis of expanded and contracted genes in the two musk-secreting mammals were performed using KEGG pathway, Reactome and Go ontology. The Top 10 enrichment pathways are shown in Fig S2a and the original results of enrichment analysis are shown in Table S7-S8.
GO enrichment analyses of contracted genes showed that these two species were both involved in pathways like "Olfactory Signaling Pathway", "Olfactory transduction", "Signaling by GPCR", "B cell receptor signaling pathway", "Tight junction Immunoregulatory interactions between a Lymphoid and a non-Lymphoid cell", "Natural killer cell mediated cytotoxicity" and "Autoimmune thyroid disease" (Fig. S2a). Gene families underwent an expansion in these two species were involved in biological processes like "Cell Cycle", "Meiotic synapsis", "Mitotic Anaphase" and "Estrogen-dependent gene expression" (P < 0.05) (all Fisher's exact test) (Table S7, Table S8).

Comment 1-8:
Line 131 The legend of Figure 1 should be revised, maybe "Genome assembly and gene family evolution in muskrat and musk deer"?. Other Figure legends should be corrected.

Response 1-8:
Thanks, as suggested, we have modified the legend of Figure 1 to: Genome assembly and gene family evolution in muskrat and musk deer. Other Figure legends were also corrected.

Comment 1-9:
Line 171-172 PSGs and REGs should be explained here or re-organized.
Response 1-9:
Thanks for the comment. The PSGs and REGs were re-organized to "Positive selection and rapid evolution genes in muskrat and musk deer were mainly involved in metabolism of lipids and epithelial regulation" part.

Comment 1-10:
Line 186-196: Each sub-figure should have a sub-legend, rather than mix them together, e.g. (b) and (c).
Response 1-10:
As suggested, we revised each sub-figure have a sub-legend (such as new Figure 2, Figure 3 and Figure 4 and so on).

Comment 1-11:
Line 187: "tissues *and* were used". Line 190: in the line"constructed at a probability of 0.95. (n = 19,800). (d)The cumulative", there should be no dot (.) after 0.95, "n" should be italic, also no space before "The". I hope the authors check and revise such typos carefully. In the following, I will not point them one by one.
Response 1-11:
Thanks for the comments. Changes have been made accordingly.

Comment 1-12:
Line 200: "We" to ", we". Line 219: Neutrophil.
Response 1-12:
Changes have been made as suggested.

Comment 1-13:
Line 238: total cell numbers of secretion (12128) and non-secretion (7270) stages are different, direct comparison of cell numbers in each cluster to infer the cell cluster related to secreting is not reasonable. The cell abundance of each cluster should be normalized firstly and them compared between the two stages. State it clearly here, cell number or cell abundance/proportion.
Response 1-13:
Thanks for the constructive suggestion.
Yes, for the comparison between two stages, we firstly normalized firstly cell abundance of each cluster in each stage. We have revised the statement in the revised manuscript.

Comment 1-14:
Line 270-271: It should be "Figure 4a". This sentence should be removed.
Response 1-14:
Change was made. As suggested, the sentence was removed in our revised manuscript.

Comment 1-15:
Line 296: In the part of HiC analysis, the authors investigated compartment and TAD dynamics between the secreting and non-secreting stages. Despite the observed massive difference, this part looks isolated from previous expression analysis. Logically the author analyzed the expression changes and inferred some candidate genes involved in the secreting progress from both bulk and scRNA-seq, the following step should be examining whether the conformation of these loci (e.g. CRABP2, Hacl1, Eci2, and Pecr) has changed in the secreting process, like from inactive compartment B to active compartment A, TAD fusion and fission. Although the authors described some cases (e.g. ROS1, SOX9, NUS1, NPNT), no expression data (Bulk, scRNA-seq) are provided. Similarly, the PEI analysis is weak. The author merged the HiC data and analyzed the interactions between promoters and enhancers, regardless of methodological issues, the meaningful PEIs should be those showing changes from non-secreting to secreting stage, like increased interaction strength.

Response 1-15:
Thanks for the helpful suggestions. As suggested, we reanalyzed the data. Thus, the candidate genes involved in the secreting progress from scRNA-seq expression were further logically analyzed whether the 3D genome conformation of these loci changed. As a result, Figure 4 and Figure S9 were regenerated.
At the PEI (promoter-enhancer interaction) level, we found that there were two genes (SMPDL3A and NRCAM) with specific PEI in musk secretion stage, also showed a compartment transition from B to A and was a marker gene in Cluster 14 (Fig. 4e). There were more specific long-range interactions (> 25Kb) in musk secretion stage for these two genes (Fig. 4f). The gene SMPDL3A showed a relative higher expression in all clusters at the musk secretion stage (Fig. 4g). SMPDL3A (sphingomyelin phosphodiesterase acid-like 3A) is an enzyme induced by lipid metabolism through liver X receptor that degrades cGAMP, modulating the cGAS-STING pathway which is involved in immune responses and lipid sensing [68]. NRCAM (neuronal cell adhesion molecule) primarily associated with neural development, its expression also promotes malignant cell transformation, cell motility, and metastatic disease [69]. In addition, MAP3K1, NODAL and Slc38a2 also showed significantly more PEIs and contacted with more enhancers during the musk secretion stage (Fig S10). MAP3K1 is a key component of the protein kinase signal transduction cascade and plays a crucial role in cellular signaling pathways [70]. NODAL maintains stem cell pluripotency and promotes directed differentiation [71]. Slc38a2 encodes an amino acid transport protein that facilitates cellular uptake of amino acids [72]. These results suggest that two important functions of the musk gland during the musk secretion stage are lipid metabolism and cell specialization, which indicate that synthesis and secretion activity were very active at this stage. Our results demonstrated that candidate loci can be analyzed in future studies of musk secretion mechanisms.

Figure S10. Promoter-enhancer interactions (PEIs) rewired in the musk gland of musk secretion and non-secretion stages.

Figure 4: Global chromatin interaction patterns in musk gland of musk secretion and non-secretion stages.

Comment 1-16:
Line 346: there is no statement in the manuscript referring to Fig 5f.
Response 1-16:
Thanks for the comment, as suggested, we reanalyzed our data and present meaningful genes in our revised manuscript and cited the figures. See comment 1-15.

Comment 1-17:
Line 411: This part should be moved following the genome assembly part.
Response 1-17:
As suggested, this part was moved following the genome assembly part.

Comment 1-18:
Line471: The paragraph about five genes in REG and DEGs in cell cluster 14 should be moved to the scRNA-seq part.
Response 1-18:
Thanks for the helpful comment, as suggested, we moved this paragraph to the scRNA-seq part.

Comment 1-19:
Line 500: Database is useful but there are too many figures in the manuscript, and I would suggest shortening this part into one or two sentences in the conclusion section, and moving the figure in the supplementary material.
Response 1-19:
Thanks for the helpful comment. As suggested, there are only five figures in our revised manuscript, including this figure. And we also shortening this part.

Reviewer #2:
The manuscript titled "Multi-omics analyses identify distinct patterns of selection in musk secretion animals" by Wang et al. explored the molecular mechanisms of musk secretion using high-quality genome, Hi-C, RNA-seq, and scRNA-seq data. Additionally, they provide an open database platform (MuskDB),

which is an important resource. Although the topic is interesting, some limitations in the text description and data analysis were found in this paper. After these concerns have been revised and clarified, I suggest that this manuscript could be accepted for publication.
Thank you for your positive comments. In our revised manuscript, we reorganized the text, figures and supplementary files. And logically, analyzed 3D genome reorganization of candidate genes with showing their expression profile in musk gland tissue and different cell types.

Comment 2-1:
1. In the part of abstract, the logical flow of the results differs significantly from the overall structure of the article, which can be confusing. Additionally, while the authors described their work using multi-omics analysis, just mentioned different pathways identified by each omics approach. Did the analysis revealed any common pathways or genes that are supported by multiple lines of evidence?
In addition, the application of musk in biomedicine should be mentioned to enhance the research significance of this paper.
Response 2-1:
Thanks for the helpful suggestions. In our revised manuscript, we reanalyzed our data. And presented candidate genes by multiple lines of evidence. The abstract was also rewritten.
In addition, as suggested, the application of musk in biomedicine was added: Natural musk has long been an important component of traditional Chinese medicine, and was used as resuscitation, blood circulation, collateral drainage, detumescence and pain relief [2].

Comment 2-2:
2. In introduction, it is suggested to propose the clear scientific questions and hypotheses. What are the key points of this study? Please highlights of these discoveries.
Response 2-2:
As suggested, in introduction, we propose the clear scientific questions: However, there are no convergent evolutionary studies on musk-producing animals to elucidate the related mechanism of musk secretion. In the process of evolution, which genes are subject to positive and convergent evolution? Which genes are differentially expressed in different cells of the musk gland during the period of musk secretion? And how the molecular process of musk secretion regulated by these genes from chromatin conformation is unknown.
The key points of this study were also rewritten: Notably, genes like SMPDL3A and NRCAM showed specific PEIs and compartment transitions, indicating their potential role in musk secretion regulation. The study concludes that the adaptation evolution of musk secretion in muskrat and musk deer is likely underpinned by active lipid metabolism and cell specialization, highlighting the complexity of the musk gland and the need for further research into the functional implications of musk secretion-specific genetic variants.

Comment 2-3:
3. The authors assembled the genomes of musk deer and muskrat using ONT, Hi-C, and NGS sequencing data. Given the relatively high error rate associated with ONT sequencing, appropriate correction methods should be applied. Additionally, the specific parameters used in the assembly process were not detailed. Please added in the methods section to provide a reference for these parameters.
Response 2-3:
Thank you for the constructive comments. As suggested, we added the specific detailed parameters used in the assembly process: For the correction of the initial contigs, we utilized the software NextPolish (v1.4.1), employing high-quality T7 paired-end reads and Nanopore long reads, and applied the recommended algorithm modules "best." in Nextpolish.
The specific parameters and pipelines used in the assembly process are available at Zenodo (https://doi.org/10.5281/zenodo.13690583).

Comment 2-4:
4. Figure S1 only shows the results for muskrats; I suggest including an additional figure to display the assembly results for musk deer. Moreover, both newly assembled genomes should be compared with existing reference genomes to further demonstrate the high quality of these assemblies, like collinearity comparison, et al.
Response 2-4:
As suggested, we compared with existing musk deer reference genomes to further demonstrate the high

quality of our assemblies.
Our two assemblies have improved N50 length of the contig for the muskrat by 1,048- and 3.56-fold compared to the published sequences [9], respectively (Fig. S1b), and musk deer by 1,048- and 3.56-fold compared to the published sequences (Fig. S1c).

Figure S1: (b) Muskrat genome assembly in this study compared with two previous studies. (d) Musk deer genome assembly in this study compared with two previous studies.

Comment 2-5:
5. Which functions are the expanded and contracted gene families associated with? This should be detailed in a supplementary table.
Response 2-5:
Thanks for the constructive comments. Enrichment analysis of expanded and contracted genes in the two musk-secreting mammals were performed using KEGG pathway, Reactome and Go ontology. The Top 10 enrichment pathways are shown in Fig S2a and the original results of enrichment analysis are shown in Table S7-S8.
GO enrichment analyses of contracted genes showed that these two species were both involved in pathways like “Olfactory Signaling Pathway”, “Olfactory transduction”, “Signaling by GPCR”, “B cell receptor signaling pathway”, “Tight junction Immunoregulatory interactions between a Lymphoid and a non-Lymphoid cell”, “Natural killer cell mediated cytotoxicity” and “Autoimmune thyroid disease” (Fig. S2a). Gene families underwent an expansion in these two species were involved in biological processes like “Cell Cycle”, “Meiotic synapsis”, “Mitotic Anaphase” and “Estrogen-dependent gene expression” ($P < 0.05$) (all Fisher’s exact test) (Table S7, Table S8).

Comment 2-6:
6. Fig. 2e-d: The authors have depicted the gene expression level distribution. However, a tissue-specific expression profile might provide more insightful information.
Response 2-6:
Thanks for the helpful comments, we agree that a tissue-specific expression profile provide more insightful information. We provided the tissue-specific expression genes enrichment analysis results in Figure S4 for each tissue.

Fig. S4 Significantly enriched GO terms in tissue-specific expressed genes for each tissue. Tissue specificity of gene abundance was reflected by the tau score (τ).

Comment 2-7:
7. Line 168: The number of tissue-specific expressed genes for each tissue should be clearly described for clarity.
Response 2-7:
As suggested, we added the numbers of tissue-specific expressed genes for each tissue.
In terms of expression, there are 3 (uterus), 35 (testis), 27 (spleen), 6 (ovary), 26 (muscle), 9 (lung), 47 (liver), 23 (kidney), 19 (heart), 10 (gland), 8 (fat), 38 (eyeball), and 112 (brain) tissue-specific genes were detected.

Comment 2-8:
8. Line 171-174: It is unclear whether the PSG and REG described in this section are also tissue-specific expressed genes related to the musk gland. These PSGs and REGs should overlapped with musk-gland tissue-specific genes, and then be analyzed for possible functions.
Response 2-8:
Thanks for the helpful comments, the genes here was not musk-gland tissue-specific genes. Thus, we moved these genes’ results to “Positive selection and rapid evolution genes in muskrat and musk deer were mainly involved in metabolism of lipids and epithelial regulation” part.

Comment 2-9:
9. If the authors later focus more on the biological function of the Acinar cells/glandular epithelial cells cluster, it would be beneficial to present the marker genes for this specific cell type using immunofluorescence staining.

Response 2-9:
Thanks for the helpful comments, the using immunofluorescence staining is very useful for present the marker genes. We need to collect sample from different musk secretion stage, this need time, we hope we could present this in our further studies.

Comment 2-10:
10. The overall methodological description in the single-cell transcriptomics section is overly simplistic. It is unclear whether the single-cell data from the two stages were analyzed jointly or separately. Additionally, it is not specified whether the pseudotime analysis was conducted using the secretion stage, the non-secretion stage, or both. These aspects need clarification to ensure a more comprehensive understanding of the analysis workflow. Providing detailed information on these points will help readers better understand the study's design and the interpretability of the results.
Response 2-10:
Thanks for the helpful comments, as suggested, we expanded methodological description in the single-cell transcriptomics section. 1) the single-cell data from the two stages were analyzed jointly; 2) pseudotime analysis was conducted using both stage cells.
Pseudotime Analysis
In R, utilizing the Monocle3 software package v1.3.1 (Cao et al., 2019), pseudotime trajectories for all 13, 14 and 22 subpopulations in the musk gland were constructed. This technique sorts individual cells along their developmental paths based on how closely their gene expression patterns match those of other sequenced cells, effectively mapping out the dynamics of cellular changes [110, 111]. Furthermore, the method evaluates how genes work together in space by using Moran's Index to measure their co-expression, which helps to establish a timeline of gene expression changes.
Identification of differentially expressed genes (DEGs) in Cluster 14 cells
DEGs in the Cluster 14 cells of musk secretion and non-secretion stages were identified using the FindMarkers function in Seurat. Genes meeting the thresholds ($|log2FC| > 1$, P-adjusted < 0.05, min.pct = 0.25) were considered as DEGs. The results were visualized using dot plots.

Comment 2-11:
11. The number of differentially expressed genes (DEGs) both in RNA-seq and scRNA-seq should be clearly specified, and detailed information should be presented in a supplementary table. Additionally, a more stringent threshold should be applied for identifying DEGs: abs(log2FC)>1 and corrected (P < 0.05). Furthermore, it is important to determine whether the tissue-specific expressed genes in the musk gland are differentially expressed between the secretion and non-secretion stages.
Response 2-11:
Thanks for the helpful comments. The number of differentially expressed genes (DEGs) in RNA-seq were presented in Figure S3e a more stringent threshold (abs(log2FC)>1 and corrected (P < 0.01)).
As suggested, a supplementary table was provided for these tissue specific genes and DEGs for scRNA-seq (Table S11 and Table S14).

Figure S3e Heatmap of DEGs (differentially expressed genes) numbers in pairwise comparisons among the 13 tissues. DEGs were identified using the threshold of abs(log2FC)>1 and corrected (P < 0.01).
Comment 2-12:
12. Line 391-393: The authors explored the changes in PEIs during the secretion and non-secretion periods, but they did not further analyze the impact of these changes on the expression levels of the corresponding genes. Including such an analysis could provide valuable insights into the regulatory effects of PEI changes on gene expression and their potential biological implications.
Response 2-12:
Thanks for the constructive comments. As suggested, we reanalyzed the data. Thus, the candidate genes involved in the secreting progress from scRNA-seq expression were further logically analyzed whether the 3D genome conformation of these loci changed. As a result, Figure 4 and Figure S9 were regenerated. The expression of the genes showed a higher expression in musk secretion stage.

Figure 4. (e) Veen diagram showing overlapping genes with compartment B to A, PEI specific to musk secretion stage, and marker genes in Cluster 14. (f) Promoter-enhancer interactions (PEIs) rewired in the musk gland of musk secretion and non-secretion stages of SMPDL3A and NRCAM. (g) Gene expression of SMPDL3A and NRCAM in each cluster at musk secretion and non-secretion stages.

Comment 2-13:
13. line 417-419: The authors have provided limited information about the criteria used for selecting positively selected genes (PSGs) and rapidly evolving genes (REGs). It is important to clearly specify the threshold settings for these selections. Additionally, the genes identified through this process should be presented in a supplementary table to ensure transparency and allow for further analysis. Moreover, the authors could leverage the species-specific PSGs and REGs to identify sets of convergently positively selected genes and convergently rapidly evolving genes. This approach could yield insights into shared evolutionary pressures or functional adaptations across different species. Including these analyses would enhance the depth and comprehensiveness of the study.
Response 2-13:
Thanks for the comments. For PSGs, we utilized the free-ratio branch-site mode (model = 1) as an alternative model, assuming positive selection on the foreground branch. The null model allowed sites to undergo purifying selection or evolve neutrally. For REGs, we utilized the branch model, specifically the one-ratio model (model = 0) as the null model assuming the same evolutionary rate for all branches, and the two-ratio model (model = 2) as an alternative model allowing different evolutionary rates for the foreground branch. The likelihood ratio test (LRT) method was used to detect differences between the nested models, and P-values were computed based on $\chi 2$ statistics. Multiple testing was corrected using the false discovery rate (FDR) method.
As suggested, we provided the PSGs, REGs and convergently genes (Table S6). Convergently positively selected genes and convergently rapidly evolving genes were also detected (Fig S3).

Fig S3. Overlapped genes between PSGs (REGs) and convergent evolution genes (a) and sequence alignment highlighting sites that evolved in 3 representative genes (b).

Comment 2-14:
14. line428: Identifying convergent amino acid substitution sites and genes can indeed be influenced by various confounding factors, potentially leading to a high rate of false positives. Implementing a multi-method approach will enhance the validity and robustness of the study's conclusions. To improve the accuracy of these identifications, employing multiple methods is advisable. Methods such as "conv_cal" and "CCS" are effective for screening convergent amino acid substitutions. By using a combination of these methods, researchers can cross-verify findings, minimize false positives, and increase confidence in identifying true instances of convergent evolution.
Response 2-14:
As suggested, we used "CCS" methods to screen convergent amino acid substitutions.
Convergent Evolution among musk secretion animals
To test for convergence among musk secretion animals (muskrat and musk deer), we used 12 mammals from Fig. 1b. Based on the phylogenetic tree, we used two methods to detect the convergent amino acid substitutions for each node of the 7,409 single-copy orthologs: 1) method of Zhang and Kumar (Zhang JZ, Kumar S. 1997. Detection of convergent and parallel evolution at the amino acid sequence level. Mol Biol Evol. 14(5):527–536; Zou Z, Zhang J. 2015. Are convergent and parallel amino acid substitutions in protein evolutionmore prevalent than neutral expectations?Mol Biol Evol. 32(8):2085–2096.). A site was assumed as a convergent site if amino acids of a focused node at that site are the same but different with their most recent ancestral amino acids. Amino acid sequences of internal nodes for all the 7,409 single-copy orthologs were reconstructed by CODEML in PAML. For each gene, the number of observed convergent site was compared with the neutral expectations derived from the JTT-fgene model, and the Poisson test was then used to evaluate the difference.
2) CCS method (Xu S, He Z, Guo Zet al. Genome-wide convergence during evolution of mangroves from woody plants. Mol Biol Evol2017; 34: 1008–15.), the convergent signal is identified when all two musk secretion species (muskrat and musk deer) share the same derived character at a conservative site.

Comment 2-15:
15. line411: To ensure robust conclusions, it would be beneficial to include a broader range of species in the analysis. This expanded dataset would help determine the identified sites exhibit true convergence specifically in muskrat and musk deer, reducing the risk of false positives that might arise from a limited set of species.
Response 2-15:

Thanks for the helpful comments. We agree that broader range of species in the analysis will help determine the identified sites exhibit true convergence specifically in muskrat and musk deer, reducing the risk of false positives that might arise from a limited set of species. Considered the quality of the species' genome and the number of one-to-one orthologous genes in the species, we selected 12 species for our analysis. We will sequenced more musk secretion species genome in our further study.

Comment 2-16:
16. line439: The TEX15 as a testis-specific protein in the text suggests that it may not be directly related to the species-specific trait of musk secretion in the musk gland. Some more apt examples, focusing on genes that are more directly associated with the unique characteristics of the musk gland could provide more relevant insights into the molecular underpinnings of this trait.
Response 2-16:
Thanks for the helpful comments. As suggested, we reanalyzed the data. Thus, the candidate genes involved in the secreting progress from scRNA-seq expression were further logically analyzed whether the 3D genome conformation of these loci changed. As a result, Figure 4 and Figure S9 were regenerated.
At the PEI (promoter-enhancer interaction) level, we found that there were two genes (SMPDL3A and NRCAM) with specific PEI in musk secretion stage, also showed a compartment transition from B to A and was a marker gene in Cluster 14 (Fig. 4e). There were more specific long-range interactions (> 25Kb) in musk secretion stage for these two genes (Fig. 4f). The gene SMPDL3A showed a relative higher expression in all clusters at the musk secretion stage (Fig. 4g). SMPDL3A (sphingomyelin phosphodiesterase acid-like 3A) is an enzyme induced by lipid metabolism through liver X receptor that degrades cGAMP, modulating the cGAS-STING pathway which is involved in immune responses and lipid sensing [68]. NRCAM (neuronal cell adhesion molecule) primarily associated with neural development, its expression also promotes malignant cell transformation, cell motility, and metastatic disease [69]. In addition, MAP3K1, NODAL and Slc38a2 also showed significantly more PEIs and contacted with more enhancers during the musk secretion stage (Fig S9). MAP3K1 is a key component of the protein kinase signal transduction cascade and plays a crucial role in cellular signaling pathways [70]. NODAL maintains stem cell pluripotency and promotes directed differentiation [71]. Slc38a2 encodes an amino acid transport protein that facilitates cellular uptake of amino acids [72]. These results suggest that two important functions of the musk gland during the musk secretion stage are lipid metabolism and cell specialization, which indicate that synthesis and secretion activity were very active at this stage. Our results demonstrated that candidate loci can be analyzed in future studies of musk secretion mechanisms.

Comment 2-17:
17. The manuscript shows p-values in all of the pathway enrichment figures, yet the methods section states that p-value correction for multiple testing was done. Additionally, line 431 mentions using p-values to indicate significance. The authors need to clarify this discrepancy regarding multiple testing correction. It is important for the authors to specify whether the p-values displayed in the figures and mentioned in the text are raw or have been corrected for multiple comparisons. This transparency is essential to ensure that the reported significance levels accurately reflect the analyses' reliability, considering the potential for false positives when multiple tests are performed. Clearly explaining these details is crucial for the study's credibility and reproducibility.
Response 2-17:
Thanks for the comments. As suggested, we have revised the p-value correction methods in our revised manuscript. It is corrected for multiple testing by the Benjamini-Hochberg method as described in our methods part.

Comment 2-18:
18. line452: It would be necessary to provide further details on the three adaptive convergent genes, such as NOP2, DST, and FAM160A1. For instance, creating a visual representation like figure, could help illustrate the situation within the context of convergent evolution.
Response 2-18:
Thanks for the constructive comments. As suggested, we generated a figure to illustrate the situation within the context of convergent evolution (Figure S3).

Fig S3. Overlapped genes between PSGs (REGs) and convergent evolution genes (a) and sequence alignment highlighting sites that evolved in 3 representative genes (b).

Comment 2-19:
19. The authors should explore whether any of the convergently positively selected genes or convergent amino acid replacement genes are related to musk secretion in the musk gland. Investigating whether these genes are specifically expressed in musk gland tissue or display species-specific expression can yield valuable insights into their relevance to the unique traits of the musk gland. These analyses could help to establish a connection between these genes and the adaptive characteristics associated with musk gland function.
Response 2-19:
Thanks for the constructive comments. As suggested, we analyzed whether the convergently positively selected genes or convergent amino acid replacement genes are related to musk secretion in the musk gland. Because there are only 10 musk gland tissue specific genes, there was no overlap with the convergently positively selected genes. Among these 10 genes, KRT80 and MPIG6B genes were also PSGs in muskrat. These two genes were also limited studied. Keratin 80 (KRT80) is an intermediate filament protein that contributes to the structural integrity of epithelial cells. The megakaryocyte and platelet inhibitory receptor gene G6P (MPIG6B) regulates platelets production, aggregation, and activation.

Comment 2-20:
20. In conclusion, the most important findings of this study should be clarified, and the description of the relevant methods will not be repeated.
Response 2-20:
Thanks for the comments. We rewritten the most important findings of this study in both abstract and introduction:
The study identified particular genes, such as SMPDL3A and NRCAM, that exhibit unique patterns of genetic interactions and changes in their genomic neighborhoods, suggesting they may play a key role in controlling the musk secretion process. The research concludes that the evolutionary adaptation for musk production in both the muskrat and the musk deer is likely driven by robust lipid metabolism and specialized cell functions, which underscores the intricate nature of the musk gland. This finding emphasizes the necessity for continued investigation to uncover the full functional impact of genetic variations that are specific to the musk secretion process.
The repeated methods were revised in our new manuscript.

Comment 2-21:
Minor suggestions:
1. Acronyms: Ensure that all acronyms are spelled out full name in the first time when they appear in the text for better understanding.
2. line691 and line698 are same.
3. Methods descriptions should also be listed in order of article logic and importance. For example, you should first write "sample collection" section and so on.
Response 2-21:
Thanks for the constructive comments. As suggested, all acronyms are spelled out full name in the first time when they appear in the text for better understanding; the repeated methods were removed; we first write "sample collection".

Reviewer #3
Comment 3-1:
Natural musk is mainly secreted by musk gland located between the navel and genitals of mature male forest musk deer. But the molecular mechanism of musk-secretion is still wait to be uncovered, hindered by the lack of comprehensive multi-omics analyses and respective platform. In this study, the authors successfully generated high quality 2.48 Gb and 2.83 Gb for muskrat and musk deer genomes with contig N50 values of 60.53 and 69.45Mb, which anchored onto 28 and 30 chromosomes, respectively. To explore the genes specifically expressed in musk gland and their functions, we used muskrat as a model animal to conduct further analyses, as muskrat tissue samples are accessible in contrast to the endangered musk deer by 168 muskrat transcriptomes. And the further single cell RNA sequencing and Hi-C analysis indicated that enhanced expression during the musk secretion stage of muskrat is related to the biological process "regulation of secretion". Overall, these results show that active lipid metabolism may underlie the adaptation evolution of musk secretion. Those findings, alongside the provided database, facilitate a deeper

understanding of the molecular mechanism underlying the unique phenomenon of musk secretion, and may provide insights for the mating behavior and breeding of muskrat and musk deer.
The findings are meaningful, the database is very important. I think it could be accepted to be published after a little polish.
Response 3-1:
We are delighted to receive your comments. We sincerely appreciate the thoughtful and constructive comments from you and another two anonymous reviewers, in improving our manuscript.
As suggested, we mainly focused the analyses on the genes with both expression and 3D genomic changes. In addition, in our revised manuscript, 1) we regenerated all figures, tables and supplementary files; 2) added and revised relative results, methods and discussion; 3) we polish our manuscript with a professional English language editing company. All comments with our point-by-point responses to each reviewer are listed. We sincerely appreciate your assistance in improving our manuscript.
