# Supplementary material for: Multiomics analysis provides insights into musk secretion in muskrat and musk deer
Source: Gigascience. 2025 Feb 26;14:giaf006. doi: 10.1093/gigascience/giaf006 (PMC11878540; doi:10.1093/gigascience/giaf006)
Supplement: giaf006_Response_to_Reviewer_Comments_Revision_1 [file giaf006_response_to_reviewer_comments_revision_1.pdf]

Author's Response To Reviewer Comments

Dear editor,
We sincerely thank you for allowing us to revise our manuscript entitled ‘Multi-omics analysis provides insights into musk secretion in muskrat and musk deer’ (original manuscript No. GIGA-D-24-00205) with substantial improvements and resolve all the concerns raised by the reviewers. 1) We revised our manuscript carefully and updated the database; 2) ‘Better Papers Faster’ was asked for our current revised manuscripts for editing service to check for grammar and polish the writing again.
Below we provide our point-to-point responses, and hope that you and the reviewers are satisfied many thanks for your consideration of our manuscript for possible publication in GigaScience.
We look forward to hearing a positive response from you.

Best regards,
Zhengrong Yuan, Diyan Li and Hang Jie

Detailed responses to reviewers
All comments provided by reviewers are listed.

Reviewer 1
Comment #1
I would like to thank the authors for carefully considering reviewers' comments. I believe the manuscript has substantially improved after the revision. I think this paper has provided rich multi-omics data resource for related studies, including high-quality genomes, RNA-seq, scRNA-seq, Hi-C and others. Selection analysis is only one part of this paper, so the "selection" in the title may be not appropriate. I think the title should be "Multi-omics analysis provides insights into musk secretion in muskrat and musk deer" or something like this. About data Availability, I did not see any statements about the public database where the datasets generated in this study were deposited. Prerequisites of acceptance must include data availability. I have no further comments on biology-related analysis. However I am not satisfied with the wording, just as the first-round review. Please note and revise the wording carefully again, especially singular and plural forms (e.g. Line512 contacts vs contact), Past tense and present tense (e.g. line514, is/showed; line527, analyzes). The authors said they have polished the English wording. However still massive but simple typos were found.
Response #1
1) Thanks for the constructive comments, as suggested, we revised the title to “Multi-omics analysis provides insights into musk secretion in muskrat and musk deer”;
2) Line510, contacts vs contact: “contacts” —>”contact”
3) Line511, is/showed: We changed “when gene Synpo2 is entered, the results showed the contact profile of this gene in two stages of secretion and non-secretion” to “When the gene Synpo2 is entered, the results display the contact profile of this gene at the stages of secretion and non-secretion”.
4) Line524, “comprehensively analyzes” was changed to “comprehensively analysis of the Hi-C”.
5) ‘Better Papers Faster’ was asked for our current revised manuscripts for editing service to check for grammar, and polish the writing again;

Comment #2
Line 35: "muskrats" should be "muskrat".
Response #2
Line 36: "muskrats" was changed to "muskrat".

Comment #3
Line 38-39: genomes can not be generated by sequencing... "We generated chromosome-level genome assemblies for the two species muskrat and musk deer, along with 168 transcriptomes from various muskrat tissues" will be better. You have spelled out the full Latin names above.
Response #3
Thanks for the constructive comment. We have made the modifications as suggested (Line 39-40).

Comment #4
Line 43: too general. The statement should be quantitative.
Response #4
Thanks for the comment. We have revised the sentence to “Single-cell RNA sequencing in muskrat musk glands identified increased acinar/glandular epithelial cells during secretion, highlighting the role of lipometabolism in gland development and evolution”.

Comment #5
Line 45: About the Database. The URL is bad. Nobody remembers the IP address of a database. I visited this database and found some functions required user registering and login, e.g. Download module and Tools module. Jbrowse only provided gene and TE element tracks. The authors have generated so many omic datasets, why not display them in the Jbrowse? Hic or HiC or Hi-C, please keep it unified.
Response #5
Thanks for the helpful comment.
(1) We have applied for the record of our database to China government. The website is: http://muskdb.cn/home/ . Please check again. And we will continually add more data of the other musk secretion animals’ data in our future study.
(2) All user registering and login are all canceled, please check the database again.

Comment #6
Line 48-49: move to result part.
Response #6
We have deleted " Genes such as SMPDL3A and NRCAM exhibited specific PEIs and compartment transitions, suggesting a role in musk secretion." from the conclusions. Because the specific expressions are already detailed in the results, here we simply want to emphasize their importance.

Comment #7
Line 107: remove "(only male secret musk)".
Response #7
Change has been made as suggested.

Comment #8
Line112: ONT coverage or all sequencing data coverage?
Response #8
It is all sequencing data coverage.

Comment #9
Line 116-117: generated 2.48-Gb and 2.83-Gb assemblies
Response #9
We changed “2.48 Gb and 2.83 Gb” to “2.48-Gb and 2.83-Gb”

Comment #10
Line 118: 69.45 Mb. No space. which were anchored
Response #10
“69.45Mb” —> “69.45 Mb”

Comment #11
Line 123-124: 1,048- and 3.56-fold for the muskrat and musk deer, compared to the published sequences, respectively ?
Response #11
Thanks for the comment, sorry for our carelessness.
Yes, it is “1,048- and 3.56-fold for the muskrat and musk deer, compared to the published sequences, respectively”.

Comment #12
Line 163: and convergence gene set?
Response #12

Yes, it is convergence gene set. Change has been made as suggested.

Comment #13
Line169-170: no chromatin structure changed genes were mentioned.
Response #13
Thanks for the comment, we have removed chromatin structure information in the sentence. Yes, this part is only PSGs.

Comment #14
Line184-185: above analysis was PSG and REG, no expression analysis.
Response #14
Thanks for the comment, we have removed this sentence.

Comment #15
Line212: why "PSGs (REGs)", rather than "PSGs, REGs"
Response #15
Thanks for the helpful comment, "PSGs (REGs)" was changed to "PSGs, REGs".

Comment #16
Line 213: Fig.1d show three genes overlapped by the three gene sets. No CKAP5.
Response #16
The results of CKAP5 is shown in Supplementary Fig. S3.

Comment #17
Line 238: change "in the transcriptome of muskrat among" to "from".
Response #17
As suggested, "in the transcriptome of muskrat among" was changed to "from" (Line 236).

Comment #18
Line 343: REGs.
Response #18
Line 340, 182, “REG” was changed to “REGs”.

Comment #19
Line 344: cell division
Response #19
“Cell division” was changed to “cell division”, Line 342.

Comment #20
Line 345: GPC4 and COL6A3, no italic
Response #20
Line 202, 204, 217, 272, 346, 349, 351, 391, 394, 471, 474, 478: The format for gene names has been corrected to italicize them.

Comment #21
Line 352: PSGs. Still too many typos. I will not point them out one by one.
Response #21
Thanks for the helpful comment, we revised the manuscript again. “PSGS” was revised to “PSGs”.
