# Supplementary material for: Multiomics analysis provides insights into musk secretion in muskrat and musk deer
Source: Gigascience. 2025 Feb 26;14:giaf006. doi: 10.1093/gigascience/giaf006 (PMC11878540; doi:10.1093/gigascience/giaf006)
Supplement: giaf006_Reviewer_1_Report_Original_Submission — Dongya Wu -- 8/14/2024 [file giaf006_reviewer_1_report_original_submission.pdf]

**Reviewer Report**

**Title: Multi-omics analysis provides insights into musk secretion in muskrat and musk deer**

**Version: Original Submission** **Date:** 8/14/2024

**Reviewer name: Dongya Wu**

**Reviewer Comments to Author:**

Wang et al. utilized multi-omics data to investigate the mechanism underlying musk secreting, by sequencing the genomes of two musk-secreting mammals (muskrat and musk deer), 188 RNA-seq libraries of muskrat organs, scRNA-seq, and HiC data. The size of dataset generated here is large and such omics data would provide great resources for related studies. However, I have several concerns.
Major:
1.The data presented in this study is impressive, but no strong connections among different data analysis sections (genome assembly, bulk RNA-seq analysis, scRNA-seq, HiC and selection analysis). The authors should carefully re-organize and connect these sections by logic. Five figures are recommended. Suggestions on section organization: PSG and REG analysis should be moved after genome assembly part. The PEI (Line373) and compartment/TAD analysis (Line296) should be merged as one section.
2.This study also sequenced 84 small RNA-seq libraries (line 78/line 145). But no analysis about small RNA was found in this manuscript. The small RNA-seq is miRNA-seq (Line 720)? I also noticed the sequencing of lncRNA-seq (Line 718). Too confusing.
3.The material&method part was poorly organized and written and I can not follow. For example, in the part named "Expression analysis of mRNA, lncRNA and miRNA"(Line863), the contents were the experimental details of RNA-seq with no expression analysis methods. Also this experimental details repeated the previous contents (Line704-724) but with inconsistent description.
4.Many typos. The authors should check and revise carefully.
Some details:
Line94-96 The sequencing depth and quality should be mentioned here. Also only OZ sequencing data information was found in Table S1.
Line 122-125 Simplify the statement, especially the use of software (CAFE, OrthoFinder..) which should be in Method. What function modules are the expanded or contracted gene families enriched in ? Or the numbers here mean nothing.
Line 131 The legend of Figure 1 should be revised, maybe "Genome assembly and gene family evolution in muskrat and musk deer"?. Other Figure legends should be corrected.
Line 171-172 PSGs and REGs should be explained here or re-organized.
Line 186-196: Each sub-figure should have a sub-legend, rather than mix them together, e.g. (b) and (c).
Line 187: "tissues *and* were used". Line 190: in the line"constructed at a probability of 0.95. (n = 19,800). (d)The cumulative", there should be no dot (.) after 0.95, "n" should be italic, also no space before "The". I hope the authors check and revise such typos carefully. In the following, I will not point them one by one.
Line 200: "We" to ", we".

Line 219: Neutrophil.
Line 238: total cell numbers of secretion (12128) and non-secretion (7270) stages are different, direct comparison of cell numbers in each cluster to infer the cell cluster related to secreting is not reasonable. The cell abundance of each cluster should be normalized firstly and them compared between the two stages. State it clearly here, cell number or cell abundance/proportion.
Line 270-271: It should be "Figure 4a". This sentence should be removed.
Line 296: In the part of HiC analysis, the authors investigated compartment and TAD dynamics between the secreting and non-secreting stages. Despite the observed massive difference, this part looks isolated from previous expression analysis. Logically the author analyzed the expression changes and inferred some candidate genes involved in the secreting progress from both bulk and scRNA-seq, the following step should be examining whether the conformation of these loci (e.g. CRABP2, Hacl1, Eci2, and Pecr) has changed in the secreting process, like from inactive compartment B to active compartment A, TAD fusion and fission. Although the authors described some cases (e.g. ROS1, SOX9, NUS1, NPNT), no expression data (Bulk, scRNA-seq) are provided. Similarly, the PEI analysis is weak. The author merged the HiC data and analyzed the interactions between promoters and enhancers, regardless of methodological issues, the meaningful PEIs should be those showing changes from non-secreting to secreting stage, like increased interaction strength.
Line 346: there is no statement in the manuscript referring to Fig 5f.
Line 411: This part should be moved following the genome assembly part.
Line471: The paragraph about five genes in REG and DEGs in cell cluster 14 should be moved to the scRNA-seq part.
Line 500: Database is useful but there are too many figures in the manuscript, and I would suggest shortening this part into one or two sentences in the conclusion section, and moving the figure in the supplementary material.

**Methods**

Are the methods appropriate to the aims of the study, are they well described, and are necessary controls included? Choose an item.

**Conclusions**

Are the conclusions adequately supported by the data shown? Choose an item.

**Reporting Standards**

Does the manuscript adhere to the journal’s guidelines on [minimum standards of reporting?](#) Choose an item.

Choose an item.

**Statistics**

Are you able to assess all statistics in the manuscript, including the appropriateness of statistical tests used? Choose an item.

**Quality of Written English**

Please indicate the quality of language in the manuscript: Choose an item.

**Declaration of Competing Interests**

Please complete a declaration of competing interests, considering the following questions:

- Have you in the past five years received reimbursements, fees, funding, or salary from an organisation that may in any way gain or lose financially from the publication of this manuscript, either now or in the future?
- Do you hold any stocks or shares in an organisation that may in any way gain or lose financially from the publication of this manuscript, either now or in the future?
- Do you hold or are you currently applying for any patents relating to the content of the manuscript?
- Have you received reimbursements, fees, funding, or salary from an organization that holds or has applied for patents relating to the content of the manuscript?
- Do you have any other financial competing interests?
- Do you have any non-financial competing interests in relation to this paper?

If you can answer no to all of the above, write 'I declare that I have no competing interests' below. If your reply is yes to any, please give details below.

I declare that I have no competing interests

I agree to the open peer review policy of the journal. I understand that my name will be included on my report to the authors and, if the manuscript is accepted for publication, my named report including any attachments I upload will be posted on the website along with the authors' responses. I agree for my report to be made available under an Open Access Creative Commons CC-BY license (http://creativecommons.org/licenses/by/4.0/). I understand that any comments which I do not wish to be included in my named report can be included as confidential comments to the editors, which will not be published.

Choose an item.

To further support our reviewers, we have joined with Publons, where you can gain additional credit to further highlight your hard work (see: https://publons.com/journal/530/gigascience). On publication of this paper, your review will be automatically added to Publons, you can then choose whether or not to claim your Publons credit. I understand this statement.

Yes Choose an item.
