# Supplementary material for: Multiomics analysis provides insights into musk secretion in muskrat and musk deer
Source: Gigascience. 2025 Feb 26;14:giaf006. doi: 10.1093/gigascience/giaf006 (PMC11878540; doi:10.1093/gigascience/giaf006)
Supplement: giaf006_Reviewer_1_Report_Revision_1 — Dongya Wu -- 11/4/2024 [file giaf006_reviewer_1_report_revision_1.pdf]

Reviewer Report

Title: Multi-omics analysis provides insights into musk secretion in muskrat and musk deer

Version: Revision 1 **Date:** 11/4/2024

Reviewer name: Dongya Wu

Reviewer Comments to Author:

GIGA-D-24-00205
I would like to thank the authors for carefully considering reviewers' comments. I believe the manuscript has substantially improved after the revision. I think this paper has provided rich multi-omics data resource for related studies, including high-quality genomes, RNA-seq, scRNA-seq, Hi-C and others. Selection analysis is only one part of this paper, so the "selection" in the title may be not appropriate. I think the title should be "Multi-omics analysis provides insights into musk secretion in muskrat and musk deer" or something like this. About data Availability, I did not see any statements about the public database where the datasets generated in this study were deposited. Prerequisites of acceptance must include data availability. I have no further comments on biology-related analysis. However I am not satisfied with the wording, just as the first-round review. Please note and revise the wording carefully again, especially singular and plural forms (e.g. Line512 contacts vs contact), Past tense and present tense (e.g. line514, is/showed; line527, analyzes). The authors said they have polished the English wording. However still massive but simple typos were found.
Line 35: "muskrats" should be "muskrat"
Line 38-39: genomes can not be generated by sequencing... "We generated chromosome-level genome assemblies for the two species muskrat and musk deer, along with 168 transcriptomes from various muskrat tissues" will be better. You have spelled out the full Latin names above.
Line 43: too general. The statement should be quantitative.
Line 45: About the Database. The URL is bad. Nobody remembers the IP address of a database. I visited this database and found some functions required user registering and login, e.g. Download module and Tools module. Jbrowse only provided gene and TE element tracks. The authors have generated so many omic datasets, why not display them in the Jbrowse? Hic or HiC or Hi-C, please keep it unified.
Line 48-49: move to result part.
Line 107: remove "(only male secret musk)".
Line112: ONT coverage or all sequencing data coverage?
Line 116-117: generated 2.48-Gb and 2.83-Gb assemblies
Line 118: 69.45 Mb. No space. which were anchored
Line 123-124: 1,048- and 3.56-fold for the muskrat and musk deer, compared to the published sequences, respectively ?
Line 163: and convergence gene set?
Line169-170: no chromatin structure changed genes were mentioned.
Line184-185: above analysis was PSG and REG, no expression analysis.
Line212: why "PSGs (REGs)", rather than "PSGs, REGs"

Line 213: Fig.1d show three genes overlapped by the three gene sets. No CKAP5.
Line 238: change "in the transcriptome of muskrat among" to "from".
Line 343: REGs.
Line 344: cell division
Line 345: GPC4 and COL6A3,no italic
Line 352: PSGs. Still too many typos. I will not point them out one by one.

Methods

Are the methods appropriate to the aims of the study, are they well described, and are necessary controls included? Choose an item.

Conclusions

Are the conclusions adequately supported by the data shown? Choose an item.

Reporting Standards

Does the manuscript adhere to the journal's guidelines on minimum standards of reporting? Choose an item.

Choose an item.

Statistics

Are you able to assess all statistics in the manuscript, including the appropriateness of statistical tests used? Choose an item.

Quality of Written English

Please indicate the quality of language in the manuscript: Choose an item.

Declaration of Competing Interests

Please complete a declaration of competing interests, considering the following questions:

- Have you in the past five years received reimbursements, fees, funding, or salary from an organisation that may in any way gain or lose financially from the publication of this manuscript, either now or in the future?
- Do you hold any stocks or shares in an organisation that may in any way gain or lose financially from the publication of this manuscript, either now or in the future?
- Do you hold or are you currently applying for any patents relating to the content of the manuscript?
- Have you received reimbursements, fees, funding, or salary from an organization that holds or has applied for patents relating to the content of the manuscript?

- Do you have any other financial competing interests?
- Do you have any non-financial competing interests in relation to this paper?

If you can answer no to all of the above, write 'I declare that I have no competing interests' below. If your reply is yes to any, please give details below.

I declare that I have no competing interests

I agree to the open peer review policy of the journal. I understand that my name will be included on my report to the authors and, if the manuscript is accepted for publication, my named report including any attachments I upload will be posted on the website along with the authors' responses. I agree for my report to be made available under an Open Access Creative Commons CC-BY license (http://creativecommons.org/licenses/by/4.0/). I understand that any comments which I do not wish to be included in my named report can be included as confidential comments to the editors, which will not be published.

Choose an item.

To further support our reviewers, we have joined with Publons, where you can gain additional credit to further highlight your hard work (see: https://publons.com/journal/530/gigascience). On publication of this paper, your review will be automatically added to Publons, you can then choose whether or not to claim your Publons credit. I understand this statement.

Yes Choose an item.
