# Supplementary material for: Multiomics analysis provides insights into musk secretion in muskrat and musk deer
Source: Gigascience. 2025 Feb 26;14:giaf006. doi: 10.1093/gigascience/giaf006 (PMC11878540; doi:10.1093/gigascience/giaf006)
Supplement: giaf006_Reviewer_2_Report_Original_Submission — Yu Wang -- 8/19/2024 [file giaf006_reviewer_2_report_original_submission.pdf]

Reviewer Report

Title: Multi-omics analysis provides insights into musk secretion in muskrat and musk deer

Version: Original Submission **Date:** 8/19/2024

Reviewer name: Yu Wang

Reviewer Comments to Author:

Comments:
The manuscript titled "Multi-omics analyses identify distinct patterns of selection in musk secretion animals" by Wang et al. explored the molecular mechanisms of musk secretion using high-quality genome, Hi-C, RNA-seq, and scRNA-seq data. Additionally, they provide an open database platform (MuskDB), which is an important resource. Although the topic is interesting, some limitations in the text description and data analysis were found in this paper. After these concerns have been revised and clarified, I suggest that this manuscript could be accepted for publication.
1. In the part of abstract, the logical flow of the results differs significantly from the overall structure of the article, which can be confusing. Additionally, while the authors described their work using multi-omics analysis, just mentioned different pathways identified by each omics approach. Did the analysis revealed any common pathways or genes that are supported by multiple lines of evidence?
In addition, the application of musk in biomedicine should be mentioned to enhance the research significance of this paper.
2. In introduction, it is suggested to propose the clear scientific questions and hypotheses. What are the key points of this study? Please highlights of these discoveries.
3. The authors assembled the genomes of musk deer and muskrat using ONT, Hi-C, and NGS sequencing data. Given the relatively high error rate associated with ONT sequencing, appropriate correction methods should be applied. Additionally, the specific parameters used in the assembly process were not detailed. Please added in the methods section to provide a reference for these parameters.
4. Figure S1 only shows the results for muskrats; I suggest including an additional figure to display the assembly results for musk deer. Moreover, both newly assembled genomes should be compared with existing reference genomes to further demonstrate the high quality of these assemblies, like collinearity comparison, et al.
5. Which functions are the expanded and contracted gene families associated with? This should be detailed in a supplementary table.
6. Fig. 2e-d: The authors have depicted the gene expression level distribution. However, a tissue-specific expression profile might provide more insightful information.
7. Line 168: The number of tissue-specific expressed genes for each tissue should be clearly described for clarity.
8. Line 171-174: It is unclear whether the PSG and REG described in this section are also tissue-specific expressed genes related to the musk gland. These PSGs and REGs should overlapped with musk-gland tissue-specific genes, and then be analyzed for possible functions.
9. If the authors later focus more on the biological function of the Acinar cells/glandular epithelial cells

cluster, it would be beneficial to present the marker genes for this specific cell type using immunofluorescence staining.

10. The overall methodological description in the single-cell transcriptomics section is overly simplistic. It is unclear whether the single-cell data from the two stages were analyzed jointly or separately. Additionally, it is not specified whether the pseudotime analysis was conducted using the secretion stage, the non-secretion stage, or both. These aspects need clarification to ensure a more comprehensive understanding of the analysis workflow. Providing detailed information on these points will help readers better understand the study's design and the interpretability of the results.

11. The number of differentially expressed genes (DEGs) both in RNA-seq and scRNA-seq should be clearly specified, and detailed information should be presented in a supplementary table. Additionally, a more stringent threshold should be applied for identifying DEGs: abs(log2FC)>1 and corrected ($P < 0.05$). Furthermore, it is important to determine whether the tissue-specific expressed genes in the musk gland are differentially expressed between the secretion and non-secretion stages.

12. Line 391-393: The authors explored the changes in PEIs during the secretion and non-secretion periods, but they did not further analyze the impact of these changes on the expression levels of the corresponding genes. Including such an analysis could provide valuable insights into the regulatory effects of PEI changes on gene expression and their potential biological implications.

13. line 417-419: The authors have provided limited information about the criteria used for selecting positively selected genes (PSGs) and rapidly evolving genes (REGs). It is important to clearly specify the threshold settings for these selections. Additionally, the genes identified through this process should be presented in a supplementary table to ensure transparency and allow for further analysis. Moreover, the authors could leverage the species-specific PSGs and REGs to identify sets of convergently positively selected genes and convergently rapidly evolving genes. This approach could yield insights into shared evolutionary pressures or functional adaptations across different species. Including these analyses would enhance the depth and comprehensiveness of the study.

14. line428: Identifying convergent amino acid substitution sites and genes can indeed be influenced by various confounding factors, potentially leading to a high rate of false positives. Implementing a multi-method approach will enhance the validity and robustness of the study's conclusions. To improve the accuracy of these identifications, employing multiple methods is advisable. Methods such as "conv_cal" and "CCS" are effective for screening convergent amino acid substitutions. By using a combination of these methods, researchers can cross-verify findings, minimize false positives, and increase confidence in identifying true instances of convergent evolution.

15. line411: To ensure robust conclusions, it would be beneficial to include a broader range of species in the analysis. This expanded dataset would help determine the identified sites exhibit true convergence specifically in muskrat and musk deer, reducing the risk of false positives that might arise from a limited set of species.

16. line439: The TEX15 as a testis-specific protein in the text suggests that it may not be directly related to the species-specific trait of musk secretion in the musk gland. Some more apt examples, focusing on genes that are more directly associated with the unique characteristics of the musk gland could provide more relevant insights into the molecular underpinnings of this trait.

17. The manuscript shows p-values in all of the pathway enrichment figures, yet the methods section states that p-value correction for multiple testing was done. Additionally, line 431 mentions using p-

values to indicate significance. The authors need to clarify this discrepancy regarding multiple testing correction.
It is important for the authors to specify whether the p-values displayed in the figures and mentioned in the text are raw or have been corrected for multiple comparisons. This transparency is essential to ensure that the reported significance levels accurately reflect the analyses' reliability, considering the potential for false positives when multiple tests are performed. Clearly explaining these details is crucial for the study's credibility and reproducibility.
18. line452: It would be necessary to provide further details on the three adaptive convergent genes, such as NOP2, DST, and FAM160A1. For instance, creating a visual representation like figure, could help illustrate the situation within the context of convergent evolution.
19. The authors should explore whether any of the convergently positively selected genes or convergent amino acid replacement genes are related to musk secretion in the musk gland. Investigating whether these genes are specifically expressed in musk gland tissue or display species-specific expression can yield valuable insights into their relevance to the unique traits of the musk gland. These analyses could help to establish a connection between these genes and the adaptive characteristics associated with musk gland function.
20. In conclusion, the most important findings of this study should be clarified, and the description of the relevant methods will not be repeated.
Minor suggestions:
1. Acronyms: Ensure that all acronyms are spelled out full name in the first time when they appear in the text for better understanding.
2. line691 and line698 are same.
3. Methods descriptions should also be listed in order of article logic and importance. For example, you should first write "sample collection" section and so on.

Methods

Are the methods appropriate to the aims of the study, are they well described, and are necessary controls included? Choose an item.

Conclusions

Are the conclusions adequately supported by the data shown? Choose an item.

Reporting Standards

Does the manuscript adhere to the journal’s guidelines on minimum standards of reporting? Choose an item.

Choose an item.

Statistics

Are you able to assess all statistics in the manuscript, including the appropriateness of statistical tests used? Choose an item.

Quality of Written English

Please indicate the quality of language in the manuscript: Choose an item.

Declaration of Competing Interests

Please complete a declaration of competing interests, considering the following questions:

- Have you in the past five years received reimbursements, fees, funding, or salary from an organisation that may in any way gain or lose financially from the publication of this manuscript, either now or in the future?
- Do you hold any stocks or shares in an organisation that may in any way gain or lose financially from the publication of this manuscript, either now or in the future?
- Do you hold or are you currently applying for any patents relating to the content of the manuscript?
- Have you received reimbursements, fees, funding, or salary from an organization that holds or has applied for patents relating to the content of the manuscript?
- Do you have any other financial competing interests?
- Do you have any non-financial competing interests in relation to this paper?

If you can answer no to all of the above, write 'I declare that I have no competing interests' below. If your reply is yes to any, please give details below.

I declare that I have no competing interests

I agree to the open peer review policy of the journal. I understand that my name will be included on my report to the authors and, if the manuscript is accepted for publication, my named report including any attachments I upload will be posted on the website along with the authors' responses. I agree for my report to be made available under an Open Access Creative Commons CC-BY license (http://creativecommons.org/licenses/by/4.0/). I understand that any comments which I do not wish to be included in my named report can be included as confidential comments to the editors, which will not be published.

Choose an item.

To further support our reviewers, we have joined with Publons, where you can gain additional credit to further highlight your hard work (see: https://publons.com/journal/530/gigascience). On publication of this paper, your review will be automatically added to Publons, you can then choose whether or not to claim your Publons credit. I understand this statement.

Yes Choose an item.
