# Supplementary material for: Multiomics analysis provides insights into musk secretion in muskrat and musk deer
Source: Gigascience. 2025 Feb 26;14:giaf006. doi: 10.1093/gigascience/giaf006 (PMC11878540; doi:10.1093/gigascience/giaf006)
Supplement: giaf006_Reviewer_3_Report_Original_Submission — Qingyou Liu -- 8/22/2024 [file giaf006_reviewer_3_report_original_submission.pdf]

Reviewer Report

Title: Multi-omics analysis provides insights into musk secretion in muskrat and musk deer

Version: Original Submission **Date:** 8/22/2024

Reviewer name: Qingyou Liu

Reviewer Comments to Author:

Natural musk is mainly secreted by musk gland located between the navel and genitals of mature male forest musk deer. But the molecular mechanism of musk-secretion is still wait to be uncovered, hindered by the lack of comprehensive multi-omics analyses and respective platform. In this study, the authors successfully generated high quality 2.48 Gb and 2.83 Gb for muskrat and musk deer genomes with contig N50 values of 60.53 and 69.45Mb, which anchored onto 28 and 30 chromosomes, respectively. To explore the genes specifically expressed in musk gland and their functions, we used muskrat as a model animal to conduct further analyses, as muskrat tissue samples are accessible in contrast to the endangered musk deer by 168 muskrat transcriptomes. And the further single cell RNA sequencing and Hi-C analysis indicated that enhanced expression during the musk secretion stage of muskrat is related to the biological process "regulation of secretion". Overall, these results show that active lipid metabolism may underlie the adaptation evolution of musk secretion. Those findings, alongside the provided database, facilitate a deeper understanding of the molecular mechanism underlying the unique phenomenon of musk secretion, and may provide insights for the mating behavior and breeding of muskrat and musk deer.
The findings are meaningful, the database is very important. I think it could be accepted to be published after a little polish.

Methods

Are the methods appropriate to the aims of the study, are they well described, and are necessary controls included? Choose an item.

Conclusions

Are the conclusions adequately supported by the data shown? Choose an item.

Reporting Standards

Does the manuscript adhere to the journal's guidelines on minimum standards of reporting? Choose an item.

Choose an item.

Statistics

Are you able to assess all statistics in the manuscript, including the appropriateness of statistical tests used? Choose an item.

Quality of Written English

Please indicate the quality of language in the manuscript: Choose an item.

Declaration of Competing Interests

Please complete a declaration of competing interests, considering the following questions:

- Have you in the past five years received reimbursements, fees, funding, or salary from an organisation that may in any way gain or lose financially from the publication of this manuscript, either now or in the future?
- Do you hold any stocks or shares in an organisation that may in any way gain or lose financially from the publication of this manuscript, either now or in the future?
- Do you hold or are you currently applying for any patents relating to the content of the manuscript?
- Have you received reimbursements, fees, funding, or salary from an organization that holds or has applied for patents relating to the content of the manuscript?
- Do you have any other financial competing interests?
- Do you have any non-financial competing interests in relation to this paper?

If you can answer no to all of the above, write 'I declare that I have no competing interests' below. If your reply is yes to any, please give details below.

I declare that I have no competing interests.

I agree to the open peer review policy of the journal. I understand that my name will be included on my report to the authors and, if the manuscript is accepted for publication, my named report including any attachments I upload will be posted on the website along with the authors' responses. I agree for my report to be made available under an Open Access Creative Commons CC-BY license (http://creativecommons.org/licenses/by/4.0/). I understand that any comments which I do not wish to be included in my named report can be included as confidential comments to the editors, which will not be published.

Choose an item.

To further support our reviewers, we have joined with Publons, where you can gain additional credit to further highlight your hard work (see: https://publons.com/journal/530/gigascience). On publication of this paper, your review will be automatically added to Publons, you can then choose whether or not to claim your Publons credit. I understand this statement.

Yes Choose an item.
